# Chemoenzymatic Synthesis of Asymmetric *N*‑Glycans in Aqueous Solution via Sulfate and Phosphate Orthogonal Protection

**DOI:** 10.1021/jacs.6c06320

**Published:** 2026-06-22

**Authors:** Pengxi Chen, Ruofan Li, Yi-Fang Zeng, Tzu-Hao Tseng, Larissa Krasnova, Chi-Huey Wong

**Affiliations:** Department of Chemistry, 4356 The Scripps Research Institute, 10550 North Torrey Pines Road, La Jolla, California 92037, United States

## Abstract

Glycoproteins often exist in nature as heterogeneous glycoforms, making it difficult to obtain pure homogeneous samples for studying the roles of specific *N*-glycans within a glycoprotein. Despite various chemical and enzymatic methods developed for the synthesis of *N*-glycans, access to highly diverse *N*-glycans remains a challenge. Herein, we report a concise strategy for the synthesis of multiantennary *N*-glycans from designed common core structures with phosphate and sulfate protecting groups, which can be selectively deprotected by respective phosphatase and sulfatase, thereby enabling enzymatic elongation to install a desired glycan chain at a specific antenna. We also explored the directing effect of galactose sulfation for enzymatic fucosylation of the neighboring GlcNAc, further expanding the structural diversity of multiantennary *N*-glycans. Starting from sulfate and phosphate-terminated core structures, this synthetic strategy in aqueous solution provides an efficient and practical route for the enzymatic assembly of a diverse array of complex *N*-glycans for biological study.

## Graphical abstract

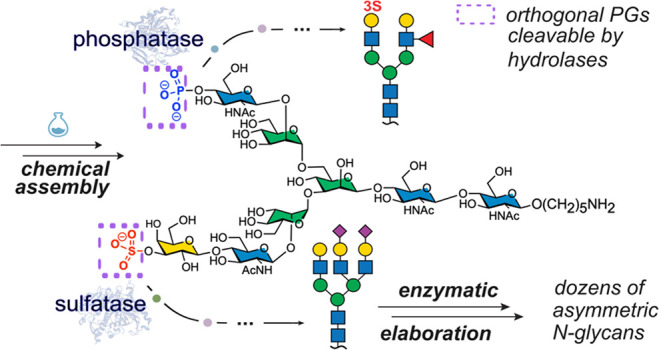

## Introduction

*N-*Glycosylation is one of the most common and complex protein modifications that affects protein structure, properties, and function.^1–5^ However, it has been a challenge to study the impact of glycosylation in biology and disease progression due to the difficulty in obtaining homogeneous, structurally well-defined glycoproteins and *N*-glycans from biological sources. Even though the enzymatic synthesis of *N*-glycans has been extensively studied,^4,5^ the high complexity of the process and substrate specificity have precluded scientists from performing total enzymatic synthesis of *N*-glycans in an efficient and practical manner. Alternatively, total chemical synthesis of *N*-glycans and *N*-linked glycopeptides has been reported.^1–3^ However, the tedious manipulations of protecting groups often limit access to structural diversity, hindering the study of the impact of *N*-glycosylation on glycoprotein structure and function.

Our recent reviews on the advancements of *N*-glycan synthesis,^4,5^ including the modular chemoenzymatic methods developed by us,^6–8^ suggest that despite substantial progress, there is still a lack of a general and efficient method to access such highly diverse and complex structures, particularly those found on human glycoproteins, which are estimated to exceed 20 thousand structures. A major challenge in chemical synthesis of *N*-glycans is the need for complex protection and deprotection strategies, as well as extensive isolation and purification procedures to achieve the desired stereoselectivity and antenna differentiation.

To extend access to structurally diverse asymmetric *N*-glycans beyond specialized chemistry laboratories, several biochemistry-centered strategies based on enzymatic antenna differentiation have been reported (Figure 1),^4,9–12^ Moreover, extensive studies of glycosyltransferases (GTs) from various organisms, have provided numerous options for the enzymatic assembly of structurally diverse *N*-glycans.^13–18^ However, enzymatic glycosylation, such as that used to install LacNAc (*N-*acetylglucosamine) repeats, is difficult to control and yields heterogeneous product mixtures. Thus, differentiation of antennae in the synthesis of multiantennary *N*-glycans remains a critical challenge for accessing *N*-glycans with increased structural complexity and well-defined glycosidic sequence, and several strategies have been deployed to tackle this problem.

**Figure 1:**
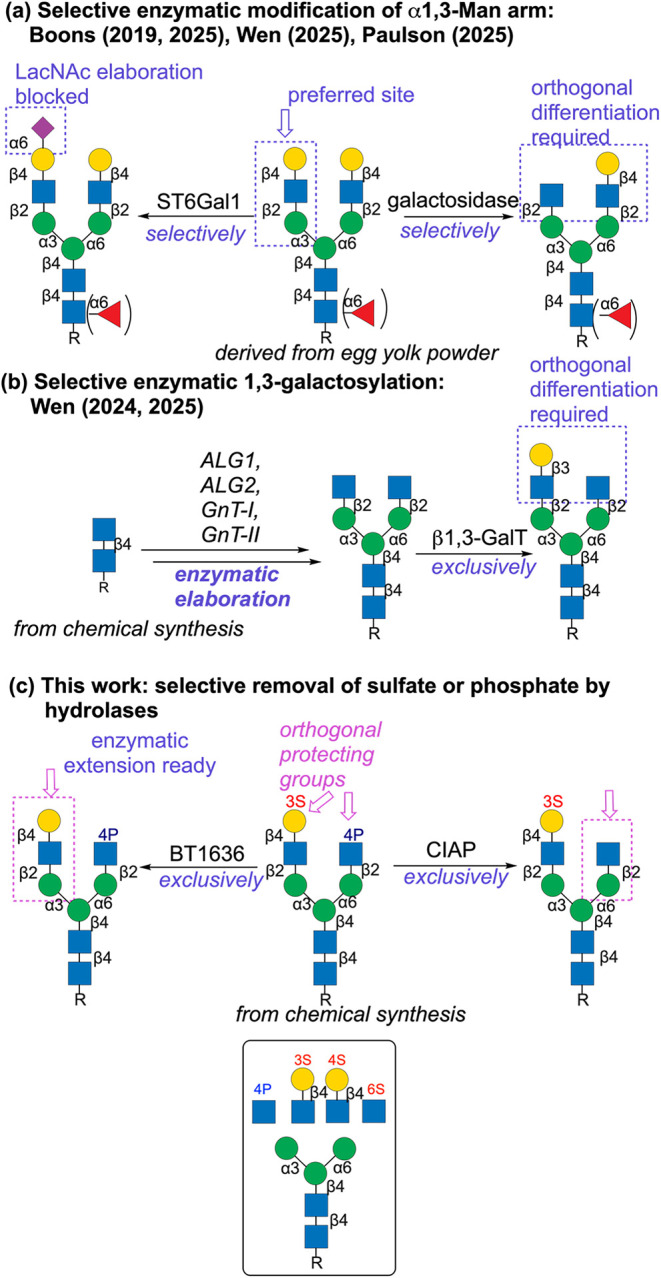
Strategies for chemoenzymatic synthesis of asymmetric *N*-glycans. Enzymatic modification of the α1,3-Man arm by (a) galactosidase and 2,6-sialyltransferase and (b) β1,3-galactosyltransferase. (c) Use of sulfate and phosphate orthogonal protecting groups for selective enzymatic deprotection and elongation (our strategy). The boxed structure shows applicable sulfate and phosphate groups as orthogonal protecting groups. R = -(CH_2_)_5_NH_2_, linker for microarray printing.

In 2017, Boons and co-workers reported on the use of unnatural sugar connections in *N*-glycans as orthogonal protecting groups to assemble asymmetric bi-, tri-, and tetra-antennary *N*-glycans.^19^ The same group later reported a “stop-and-go” strategy for differentiating *N*-glycan antennae using a GlcNTFA residue (*N*-trifluoroacetyl glucosamine) as a switch for the pending enzymatic modifications.^20^

In parallel, several alternative strategies with enzymatic differentiation of the α1,3-Man arm from the α1,6-Man arm on G0 or G2 *N*-glycan cores have been developed. These methods rely on *N*-glycan cores obtained either from egg yolk or via chemoenzymatic synthesis (Figure 1a and 1b);^21–24^ however, these strategies require carefully arranged enzymatic steps to achieve full differentiation of the two arms, making these approaches inconvenient for generating a broad array of structures, especially for variations on the α1,3-Man arm.

As a consequence, antenna-selective enzymatic assembly of asymmetric *N*-glycans remains limited in scope, restricting access to more structurally complex *N*-glycans with well-defined glycosidic sequences. For this purpose, aqueous-compatible protecting groups can be a transformative tool for guiding enzymatic modification of branched carbohydrates. Phosphoryl groups have been used as directing groups in the synthesis of peptides,^25^ oligonucleotides,^26^ and carbohydrates.^27^ However, the heavy hydroxylation of the latter requires multiple transient directing groups for differentiation. Herein, we developed a conceptually simple and practical strategy for the synthesis of asymmetric *N*-glycans. Our approach leverages orthogonal protecting groups (sulfate and phosphate, Figure 1c) that can be selectively removed by corresponding enzymes in aqueous media, enabling controlled enzymatic elongation at the selected antennae. With the availability of core intermediates **1** and **19** in Scheme 2 (*vide infra*), our method eliminates the labor-intensive chemical steps, making it user-friendly and readily implemented in biochemistry-focused laboratories. In addition, we observed that fucosylation could be guided by the sulfation pattern of the LacNAc motif, thereby expanding our library to sulfated and fucosylated *N*-glycans.

## Results and Discussion

In our initial search for appropriate protecting groups, the acyl group was considered a suitable candidate.^6^ However, we observed the undesired acyl migration in the partially protected trisaccharide model compound in an aqueous solution (see Figure S1, Supporting Information). A similar observation has been reported for sialyl and galactopyranosyl scaffolds, even with bulky acyl groups, in alkaline media.^28,29^ Such migration would complicate the reaction system, generating mixtures of acyl-migrated byproducts that can interfere with subsequent glycosylation and render this type of protecting group not suitable for our approach.

In the search for better alternatives, naturally occurring posttranslational modifications of sugars drew our attention. Specifically, sulfate and phosphate groups appeared to be attractive candidates due to their ubiquitous presence in natural glycans as stable functionalities and their feasible removal by specific hydrolases. Notably, phosphoryl migration was reported at pH 1.0 but not at pH 7.0 or 9.0, the typical pH range used for GT-mediated *N*-glycan modification.^30^ The sulfate group stability was verified by NMR as presented in Figure S2, Supporting Information.

### Assessment of Sulfate and Phosphate Groups in a Monosaccharide Model Study

To keep chemical synthesis concise, we first planned to install sulfate and phosphate groups on GlcNAc. As shown in Entry 1, Table 1, a widely used calf intestinal alkaline phosphatase (CIAP) hydrolyzed 3*O*-phosphate on GlcNAc with great efficacy. As CIAP cleaves phosphate monoesters with wide structural diversity, it was expected to hydrolyze phosphate at other positions of GlcNAc. With C4-OH being the galactosylation site for the type II LacNAc motif, we planned to cap C4-OH of GlcNAc with phosphate as a protecting group.

**Table 1:**
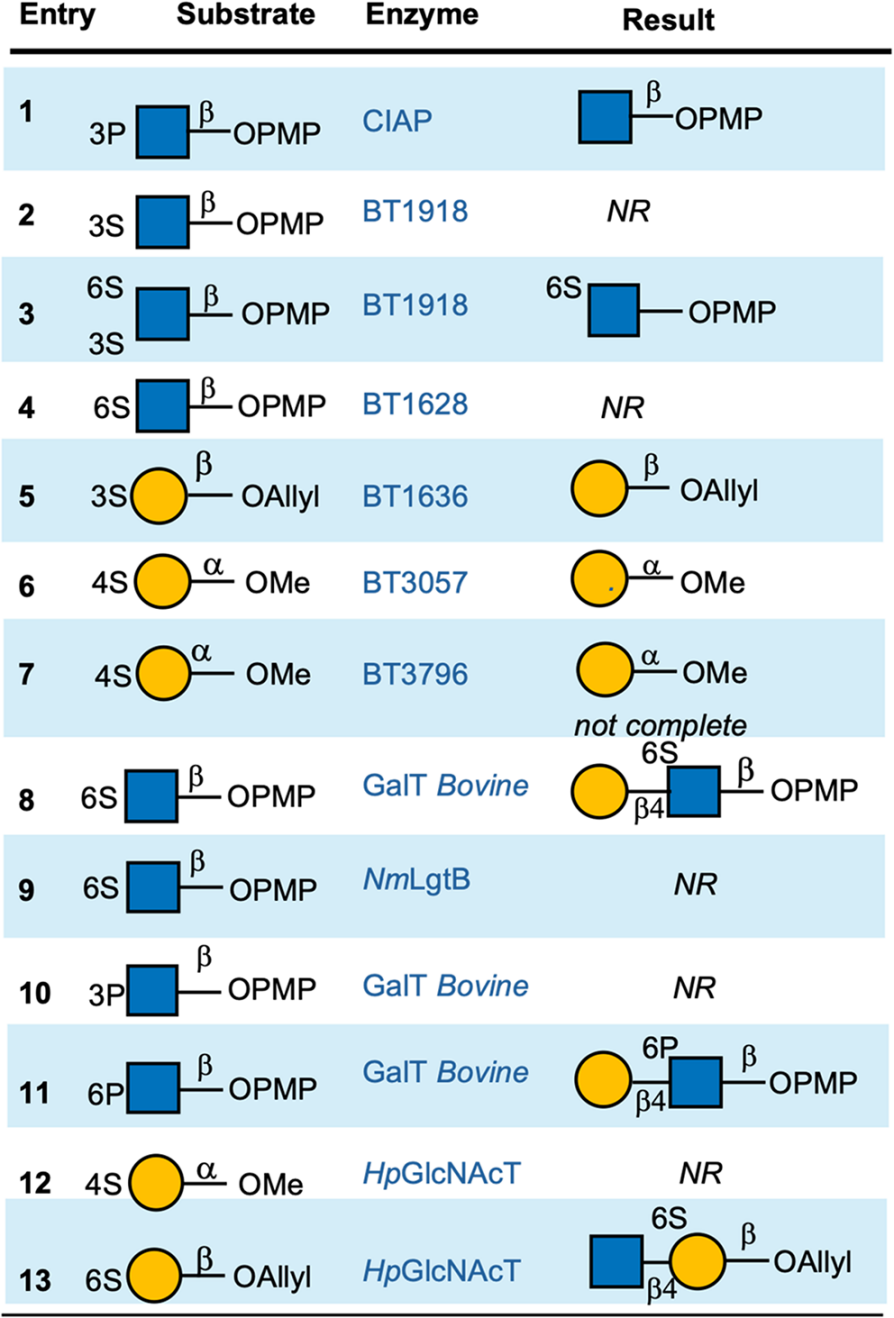
Recognition of Phosphorylated or Sulfated Galactosides and *N*-Acetylglucosaminosides by Hydrolases and Glycosyltransferases (GTs)^a^

a NR = no reaction. PMP: *p-*methylphenyl; enzymes: CIAP: calf intestinal alkaline phosphatase; BT1628, BT1918, BT1636, BT3057, BT3796: sugar sulfatases from *B. thetaiotaomicron* *;* *Hp*GlcNAcT: *H. pylori* β1,3-*N*-acetylglucosaminyltransferase; GalT *Bovine*: bovine β1,4-galactosyltransferase; *Nm*LgtB: *N. meniningitidis* β1,4-galactosyltransferase.

Identifying a suitable sulfatase, however, was not as straightforward and required extensive research. First, we tested several commercially available sulfatases (*i.e*., arylsulfatases from abalone entrails, *Patella vulgata*, *Helix pomatia*, and *Aerobacter aerogenes*), which were reported to cleave sulfate groups of carbohydrates. However, none of them showed detectable activity toward 3S or 6S-GlcNAc. Then, we switched our attention to human gut microbes, which possess a powerful arsenal of carbohydrate-active enzymes (CAZymes) recognizing a wide range of complex carbohydrates, including sulfated glycans.^31–36^ Several sulfatases from the gut microbe *Bacteroides thetaiotaomicron*, previously reported to hydrolyze sulfated GlcNAc, were expressed and tested (Entries 2—4).^37^ Among these enzymes, BT1918 exhibited the reported hydrolytic activity, cleaving 3*O*-sulfate from 3S,6S-GlcNAc, but not from 3S-GlcNAc (Entries 4 and 2), indicating its exquisite substrate specificity.

In contrast to the reported reactivity toward 6*O*-sulfated GlcNAc, sulfatase BT1628 failed to hydrolyze the substrate in our hands (Entry 3). Unlike the phosphate groups that could be efficiently removed by CIAP, no matter where they are located on the carbohydrate backbone, the sulfate groups on oligosaccharides (e.g., glycosaminoglycans) usually require specific sulfatases for their hydrolysis.^38^ Therefore, it might be difficult to identify a sulfatase that matches a sulfate group arbitrarily positioned on a sugar motif, such as the aforementioned 3S-GlcNAc and 6S-GlcNAc, as well as rarely seen 4S-GlcNAc. Consequently, we turned our attention to sulfated galactose, as naturally occurring galactosides exhibit more diverse sulfation patterns (*e.g.*, mucin *O*-glycans) and might provide desired solutions. To our delight, the sulfatases from the above-mentioned gut microbe *B. thetaiotaomicron* worked very well on 3S- and 4S-galactoses (Entries 5 and 6). BT1636 cleaved the 3*O*-sulfate group on galactose cleanly, and BT3057 worked for 4S-galactose. Moreover, sulfation on either 3*O*- or 4*O*-position of a galactose prevents its 3*O*-glycosylation mediated by *N*-acetylglucosaminyltransferase (*Hp*GlcNAcT, Entry **12**). Therefore, 3S-Gal with matching sulfatase BT1636 as the deprotecting enzyme was selected as a promising candidate for our chemoenzymatic strategy.

In parallel, we also tested the promiscuity of several GTs on phosphorylated and sulfated sugars (Entries 8—13), and the results provided useful insights for the subsequent design of the enzymatic route. For example, 6S-GlcNAc was recognized by bovine β1–4GalT1 (Entry 8), but not *Neisseria meniningitidis* β1,4-GalT *(Nm*LgtB, Entry 9).^18^ For phosphorylated GlcNAcs, bovine β4GalT1 accepted 6P-GlcNAc as a substrate (Entry 11), but not 3P-GlcNAc (Entry 10). Moreover, we found that 4S-Gal was not a substrate for *Helicobacter pylori* β1,3-GlcNAcT (*Hp*GlcNAcT, Entry 12), but 6S-Gal permitted 3*O*-glycosylation facilitated by the same enzyme (Entry 13). These findings showed that, in addition to 3S-Gal, the 6S-GlcNAc, 3P-GlcNAc, and 4S-Gal also exhibit the desired protecting group orthogonality for our strategy.

Next, we extended the substrate scope of BT1636 and CIAP to tetrasaccharide **2** (Scheme 1), a substrate that closely represents a biantennary *N*-glycan and carries both sulfate and phosphate groups on different antennae. As anticipated, the desulfated product **3** and dephosphorylated product **4** were obtained efficiently after the treatment with BT1636 and CIAP, respectively. With this success, we were confident that the strategy could be implemented into the chemoenzymatic synthesis of biantennary *N*-glycans. Parenthetically, tetrasaccharide **2**, with an asymmetric GlcNAc_2_Man core, could serve as an intermediate toward *O*-mannose glycans of M2 type.

**Scheme 1:**
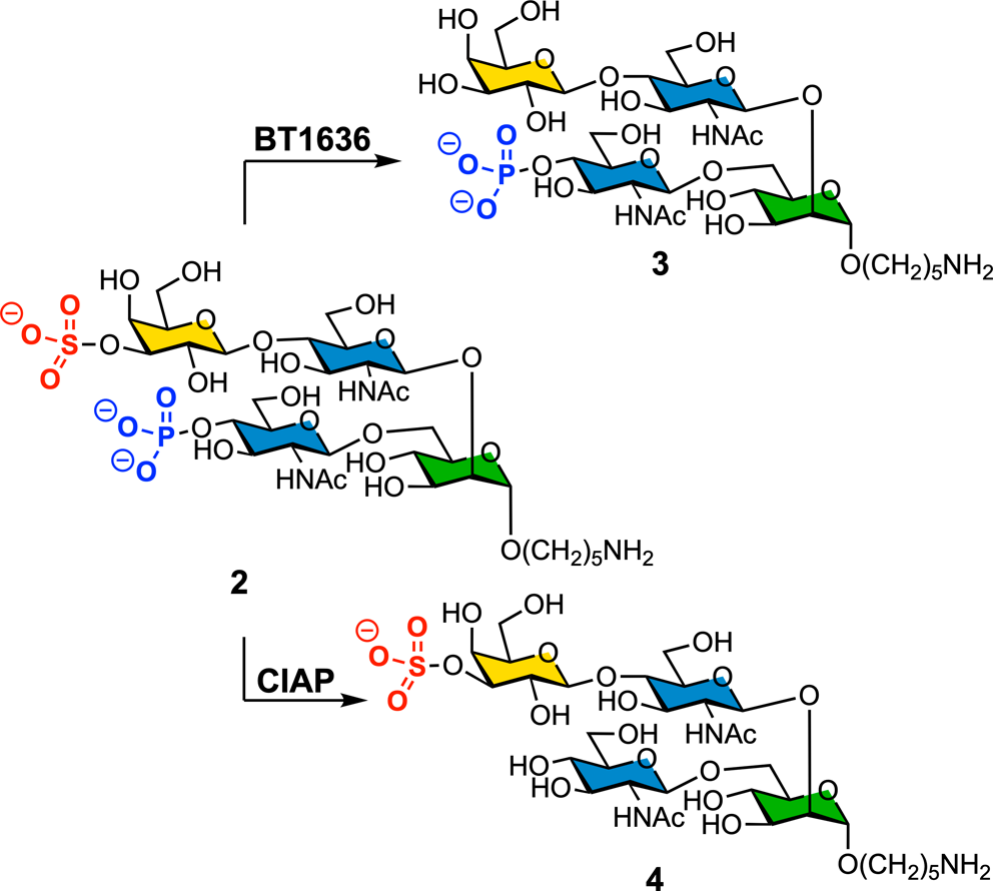
BT1636 and CIAP Processing of Bi-Antennary Tetrasaccharide **2**

### Chemical Synthesis of Biantennary *N*-Glycans Bearing Sulfate and Phosphate Groups

Based on our screening, we validated 3S- and 4S-Gal, as well as 4P-GlcNAc, as orthogonally protected terminal residues for *N*-glycan. An advanced biantennary intermediate **13** was designed accordingly (Scheme 2), bearing 3S-Gal and 4P-GlcNAc terminals for differentiating the α1,3- and α1,6-Man arms.

**Scheme 2:**
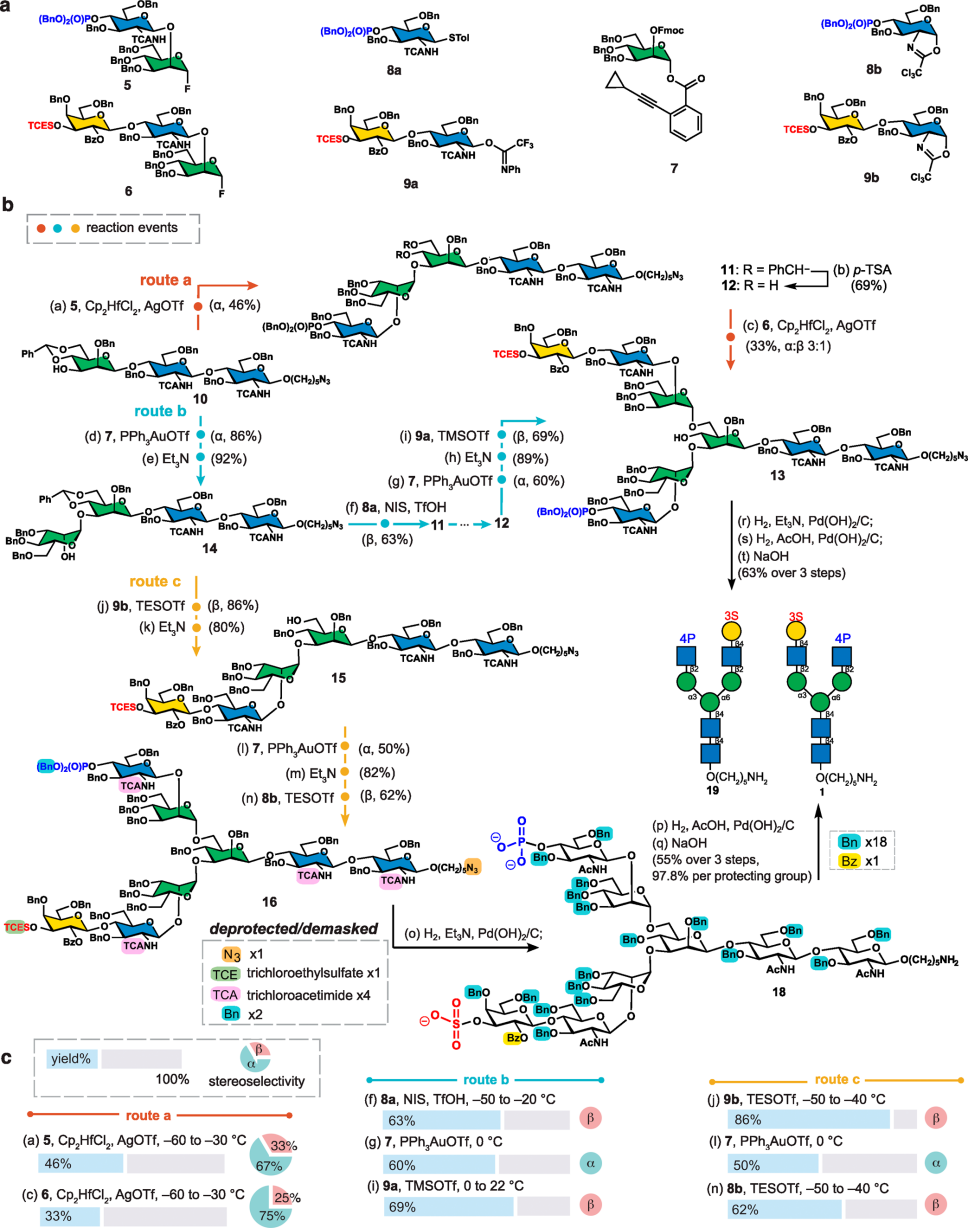
Assembly of Bi-Antennary *N*-glycans **1** and **19**, Capped with Sulfate and Phosphate Groups for Enzymatic Differentiation: (a) Glycosylation Donors; (b) Preparation of **1** and **19**; (c) Comparison of Yield and Stereoselectivity of Glycosylation Reactions in Routes a–c

The traditional introduction of the sulfate group into carbohydrates is based on the late-stage sulfation by SO_3_ derivatizing reagents (e.g., SO_3_·Py and SO_3_·Et_3_N). Limitations of this approach include tedious protecting group manipulations, difficulties in handling the highly polar sulfates during synthesis and purification, and low reaction efficacy, especially for multiple sulfation sites at a late stage. To overcome these drawbacks, Taylor et al. (2010) developed an efficient methodology that uses 2,2,2-trichloroethyl (TCE) as a protecting group for the sulfate ester.^39^ TCE sulfate (TCES) embedded sugar donors exhibit good glycosylation reactivity and could be easily removed under mild conditions with Zn(0)-ammonium formate or Pd(0)/C-ammonium formate. With this methodology, the protection-free sulfate group of **19** could be smoothly obtained from the TCE sulfate precursor **13**, whose sulfate group could originate from donors **6** and **9a** (*vide infra*). To keep TCES intact during downstream chemical transformations, protecting groups of **13** were meticulously selected. For example, *N*-acetylamide of GlcNAc was masked as trichloroacetamide (TCA), because unlike other GlcNAc protecting groups, demasking of GlcNTCA can be accomplished under very mild conditions without affecting TCES.

We then proceeded to the assembly of building blocks into glycan **19**. Adapting the disconnection strategy from our previous work on *N*-glycan synthesis,^6,7^ disaccharide **5** and trisaccharide **6** were proposed to serve as glycosylation donors to construct two Man-Man linkages (i.e., **10** → **11** → **13**, **Route a.** See our previous paper for the preparation of the precursor of **10**.^8^). However, Mukaiyama-Suzuki glycosylation (i.e., using **5** and **6** as donors, Cp_2_HfCl_2_–AgOTf as promoter)^40–43^ only delivered products **11** and **13**, respectively, with poor stereoselectivity,^44^ which was then largely improved with a stereoselective glycosylation using an *o*-alkynylbenzoate donor equipped with a 2*O*-directing group.^19,45^ As depicted in **Route b** (i.e., **10** → **14** → **13**), donor **7** with Fmoc as a directing group was used to react with trisaccharide **10**, affording exclusive α-selectivity on the Man(1–3)­Man linkage. This step was followed by Fmoc removal (Et_3_N) to produce **14**, whose subsequent glycosylation with thioglycoside **8a** completed the assembly of the GnT-I arm as in **11** in 45% overall yield. Hydrolysis of the benzylidene group of the latter (*p*-TSA, MeCN) afforded diol **12** (69%), which was subjected to the sequential glycosylation reactions with **7**, Fmoc deprotection, and glycosylation reaction with **9a**, furnishing octasaccharide **13** in 37% overall yield, with exclusive α configuration at Man(1–6)­Man linkage. Although the stereoselectivity was improved using this approach, the regioselectivity remained unresolved due to the close reactivities of C4-OH and C6-OH on the central mannose of **12** (see Supporting Information for details).

To improve synthetic efficiency and block the undesired 4*O*-glycosylation, we switched our synthetic target to **1** and redesigned our synthetic route (Scheme 2, **10** → **15** → **1**, **Route c**). The reason for having 3S-Gal on the GnT-I arm in glycan **1** as opposed to **19** was guided by the need to carry out a regioselective benzylidene opening, which is compatible with the TCES group but not a benzyl phosphate, making the GnT-I arm the appropriate site for 3S-Gal installation. As such, hexasaccharide **15** was prepared from **14**, and the deprotected C6-OH was then unambiguously glycosylated with glycosylation donor **7**.

However, the glycosylation with either thioglycoside (**8a**) or trifluoroacetimidate (**9a**) donor was not as clean as we expected. Both reactions produced significant amounts of oxazolines **8b** and **9b**, respectively, along with unidentified polar byproducts, which diminished the yield, largely due to decomposition of the substrate. When oxazoline **9b** was used as a glycosyl donor, we noticed that **14** was smoothly glycosylated at a much lower temperature of −45 °C, resulting in a cleaner reaction (See Figure S3, Supporting Information). A similar observation was made for the glycosylation with oxazoline donor **8b**, whose activation temperature (below −40 °C) was significantly lower than that of **8a** (above −30 °C),^46–48^ resulting in a cleaner glycosylation. The formation of trichloroacetyloxazolines as glycosylation byproducts and their use as active glycosyl donors have been previously reported.^49–51^ Eventually, the fully protected precursor of **1** was prepared from **14** via sequential glycosylation reactions with donors **9b**, **7**, and **8b**, accompanied by proper manipulations of protecting groups, in 24% overall yield for five steps.

### Tackling the Challenging Hydrodechlorination of *N*-TCAs for Global Deprotection

To this end, both fully protected octasaccharides were subjected to global deprotection, a formidable task of removing/demasking 19/20 benzyl groups, 4 trichloroacetyl groups, 1 trichloroethyl group, 1 azido group, and a benzoate ester. Even though the trichloroacetyl group has been widely used as a masking group for *N*-acetylamide in carbohydrate synthesis, the demasking process is notorious for the stubborn, incomplete dechlorination, especially when multiple TCAs are present in a single molecule. Two methods have been developed to tackle this problem: (1) radical-mediated hydrodechlorination using Bu_3_SnH or Zn (0) species, and (2) alkaline hydrolysis followed by *N*-acetylation. Both methods require excess of reagents, prolonged reaction times, and sophisticated monitoring, yet provide subpar yields.^44,49,52–54^ Therefore, a more practical and robust method is needed for the complex substrates, such as octasaccharide **13** (cf. Scheme 2).

Retrospectively, for the monosaccharides from Table 1, the *N*-acetyl group of GlcNAc derivatives was masked with TCA and demasked via hydrogenolysis using Pd (OH)_2_/C. At first, the hydrodechlorination toward 3P-GlcNAc-OPMP (Entry 1, Table 1) was fast for the first two chlorine atoms of TCA, but sluggish for the last one. The reaction was incomplete even after 2 days, when all benzyl ethers were cleaved. In comparison, the global deprotection toward 3S-GlcNAc-PMP (Entry 2, Table 1) using Et_3_N as an additive, following Ingram’s procedure for TCES demasking (2010),^55,56^ resulted in a complete and rapid dichlorination of both TCES and *N*-TCA moieties, followed by debenzylation to afford the fully deprotected product after overnight treatment. We proposed that Et_3_N base not only neutralized HCl generated *in situ* but also accelerated hydrogenolytic dechlorination. When the procedure was applied to furnish octasaccharides **1** and **19**, the hydrodechlorination of the TCE-sulfate and four *N*-TCAs, with a total of 15 chlorine atoms, was rapidly accomplished, along with the reduction of the azido group and partial debenzylation of phosphate (as revealed by ^1^H NMR). After the Et_3_N-promoted hydrogenolysis and demasking of *N*-TCAs were accomplished in 12 h, monitored by TLC and NMR, the reaction mixture was filtered and concentrated to remove excess Et_3_N. Then, an acidic hydrogenolysis [Pd­(OH)_2_/C, AcOH, H_2_(balloon)] to cleave all benzyl ethers was performed smoothly, followed by hydrolysis of benzoates on the Gal residues, to afford the final asymmetric octasaccharides **1** and **19** as pure products via purification by size-exclusive chromatography (P-2 gel, dH_2_O).

As such, the deprotection/demasking of 20 benzyl groups, 4 trichloroacetyl groups, 1 trichloroethyl group, and 1 azido group was accomplished in a simple two-step hydrogenolysis, avoiding the common formation of byproducts from the saturation of the benzene ring in the global deprotection of complex carbohydrates. The complete hydrodechlorination of *N*-TCAs was especially remarkable with the advantages of mild reaction conditions, short reaction time, and simple purification (i.e., ambient temperature, simple base as additive, overnight for 4 *N*-TCAs, with no need for HPLC). A similar accelerating effect of Et_3_N on hydrodechlorination of aromatic chlorides was reported by Sajiki and co-workers (2006).^57^ They showed that Et_3_N, as well as several lipophilic organic bases [e.g., DBU and 2,6-di-*tert*-butyl-4-methylpyridine (DTBMP)], promoted hydrodechlorination of 4-chlorobiphenyl, otherwise unproductive under neutral Pd(0)/C hydrogenolysis. Combined with our results, using a base as an additive for hydrogenolysis could be a promising solution to tackle the challenging Pd (0)/C-mediated hydrodechlorination of the TCA group in carbohydrate synthesis.

### Enzymatic Differentiation and Elaboration of Orthogonally Protected *N*-Glycans

The established practical chemical synthesis led to a platform for the enzymatic assembly of asymmetric *N*-glycans. As depicted in Scheme 3, intermediate **19** was first treated with CIAP to remove the phosphate group selectively, affording **21**, which was accepted as substrate by the highly conserved GnT-IV to give the triantennary *N*-glycan **22**, expanding the structural complexity of our strategy. Enzymes GnT-IV and V were reported to tolerate a few structural modifications on GlcNAc on the α1,3- and α1,6-Man arms, respectively, but do not tolerate the addition of a β1,4-galactoside to the other arm. The recognition of **21** and **25** (*vide infra*) by GnT-IV and V highlights the versatility of sulfate in the enzymatic elaboration of *N*-glycans. The β1,4-galactosylation on **22** mediated by bovine β4GalT (bovine) and the following α2,3-sialylation by Vs16 (*Vibrio sp.* JT-FAJ-16)^13,58^ afforded **23** and **24** in quantitative yields. In parallel, glycan **1** was processed using a similar sequence (See Supporting Information for details). Deprotection of **1** with CIAP delivered the dephosphorylated product **25**, which was treated with GnT-V, to install a branch of GlcNAc on the C6 position of the Man-α1,6-Man arm to give triantennary *N*-glycan **26**. Proceeding through the similar sequence of galactosylation and sialylation, **26** was elaborated into **27** and **28**, serving as isomeric glycans of **23** and **24**, and rendering valuable structure variations for studying the influence of different sugar arrangements on their interaction with biomolecules. Finally, the robustness of sulfatase BT1636 was signified by the conversion of **28** to **29**, where the sulfate was removed smoothly despite the structural complexity and congestion of the triantennary substrate carrying multiple negative charges.

**Scheme 3:**
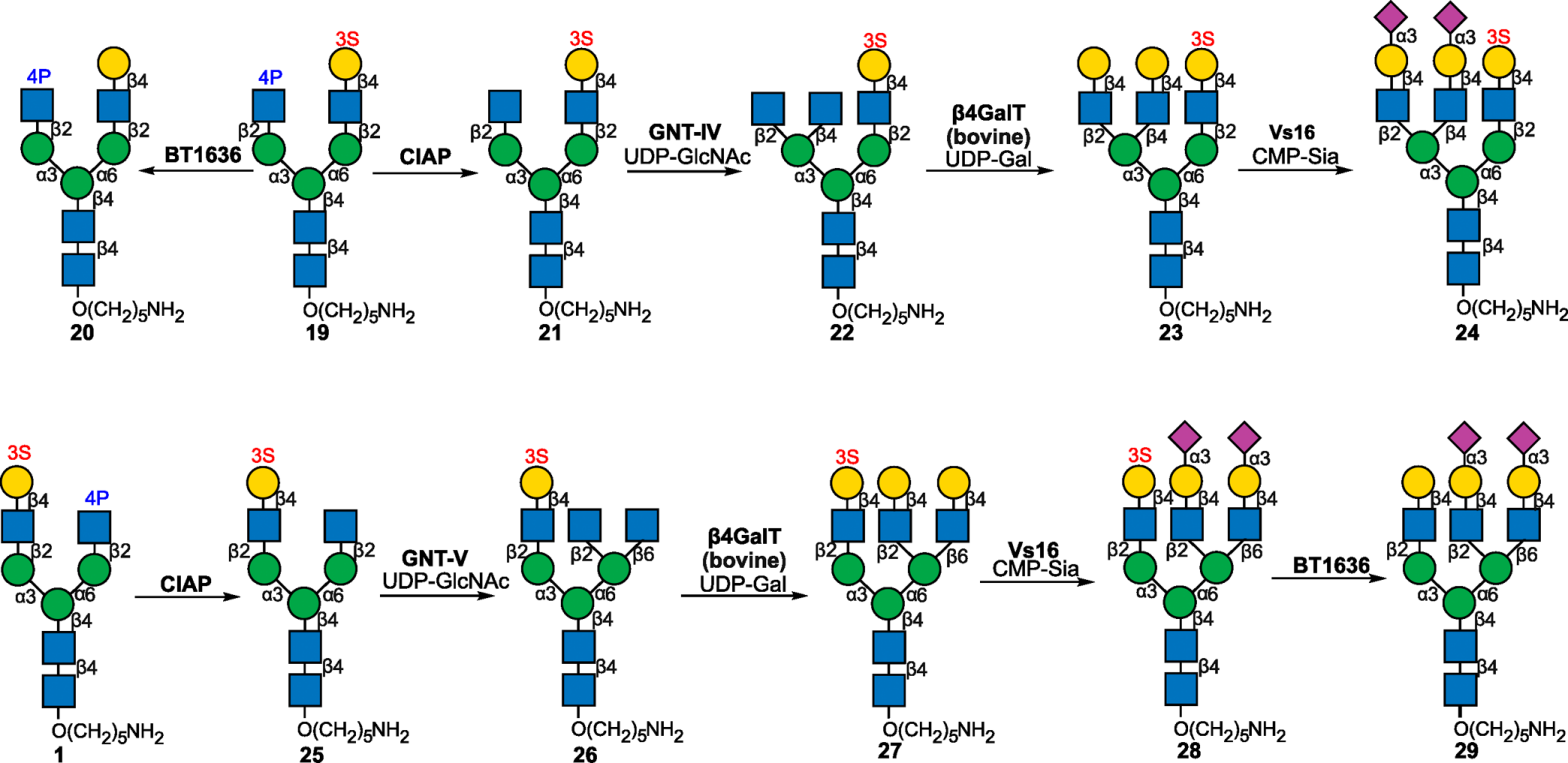
Enzymatic Elaboration of Bi-Antennary *N*-Glycans **19** and **1**, Leading to Structurally Diverse Asymmetric *N*-Glycans

In addition to the above-described enzymatic syntheses initiated by the selective deprotection of the phosphate group on **1** and **19**, selective desulfation by BT1636 was also conducted on **19** to afford the desulfated product **20**, enabling the enzymatic synthesis complementary to the CIAP-differentiated sequence.

### Selective Fucosylation on LacNAc Motifs

To enhance the structural complexity and diversity of our strategy, sulfate-directed fucosylation on GlcNAc was studied. We first verified the selective fucosylation of 3′-sulfate and unmodified LacNAc motifs. Based on the previously reported specificity of human α1,3-fucosyltransferases (FuTs) over nonsubstituted, 3′-sialyl, and 3′-sulfate LacNAcs, complementary selectivity could be achieved with selected FuTs.^59^ Thus, we tested the substrate preference of FuT-5, 6, and FuT-9 on 3′-sulfated, 3′-phosphorylated, and plain LacNAc structures (Scheme 4a). Competing reactions were carried out between trisaccharides **30a** and **31**, as well as **30b** and **31.** The mass difference of the resulting reaction mixture, as determined by LCMS, was used to assign the fucosylated products. From this experiment, we observed that FuT-9 prefers nonmodified LacNAc **31** over 3′S-LacNAc **30a** and 3′P-LacNAc **30b**, giving **33** as the only fucosylated product when 1.1 equiv of GDP-fucose were used. The FuT-5 and FuT-6 showed complementary preference for FuT-9, affording fucosylation product **32a**/**b** with control of GDP-fucose equivalence. Notably, FuT-5 caused the decomposition of GDP-fucose during the overnight reaction,^21^ and therefore FuT-6 was used next to mediate a clean conversion. Interestingly, despite structural discrepancies, 3′P-LacNAc regulates these FuTs in the same way as the sulfated substrate does and therefore could also be utilized to direct selective fucosylation as needed. Based on these results, *N*-glycan **34**, which was prepared *in situ* from **25** (see Supporting Information for more details), was treated with FuT-6 and FuT-9 to deliver fucosylated products **35** and **36**, respectively, in a site-selective manner.

**Scheme 4:**
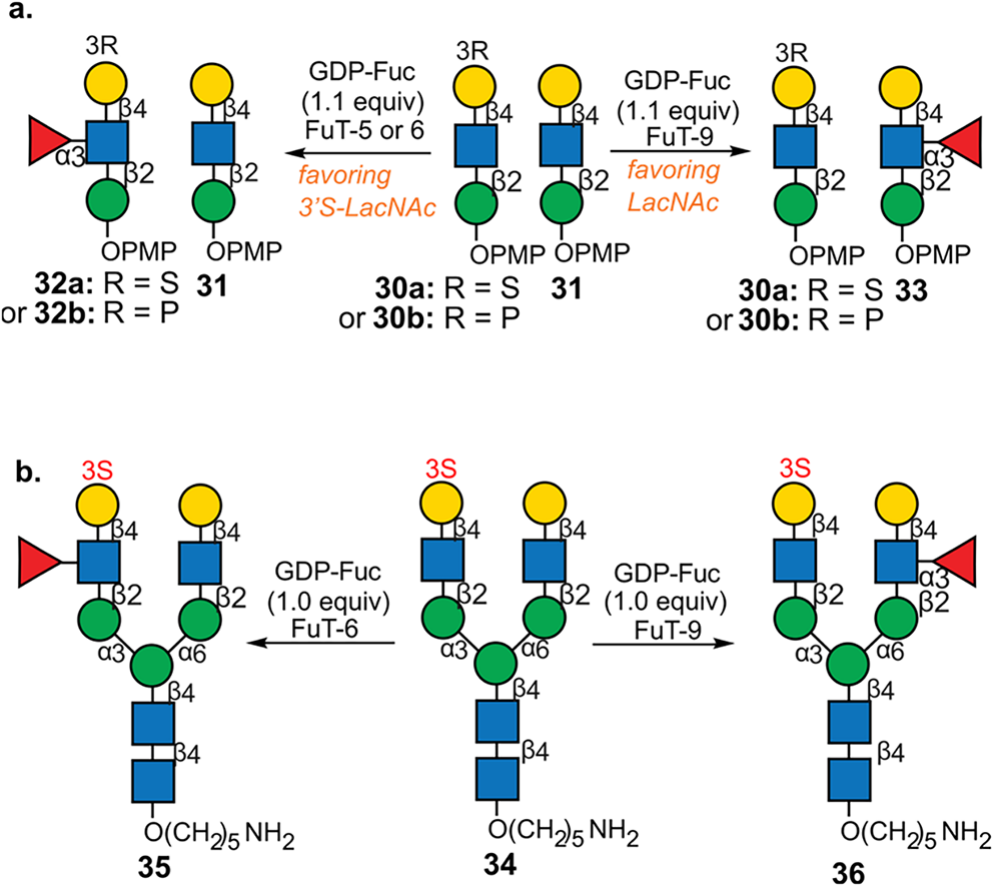
(a) Competition Studies between 3′-Sulfated LaNAc **30a** or Phosphorylated LacNAc **30b** and Non-Modified LacNAc **31** for Determining the Preferences of Fucosyltransferases FuT-5, 6, and 9; (b) Selective Fucosylation of **34** by FuT-6 and FuT-9

Full assignments of fucosylated glycans **35** and **36** were achieved through careful systematic analysis of ^1^H, ^13^C, HSQC, H2BC, and HMBC spectra, guided by previously reported assignments of SCT.^60,61^ The fucosylation sites in the reactions described above were confirmed by 2D NMR spectroscopic analysis. The glycosyl substituents on sugars exert an α-glycosylation effect on the attached carbon, causing a ^13^C signal shift of 6–10 ppm to lower field, and a β-glycosylation effect on the nucleus adjacent to the glycosyl substituents with a ^13^C shift of 0–3 ppm to upfield.^60,61^ Based on these spectroscopic rules, once ^13^C chemical shifts of C3 and C4 of both GlcNAc (3) and (4) (numbering shown in Figure 2a) were assigned, the chemical shift differences were used to determine the fucosylation site. The 3-fucosylation causes a downfield shift of C3 of the fucosylated GlcNAc and an upfield shift of C4 compared to the unfucosylated GlcNAc.

**Figure 2:**
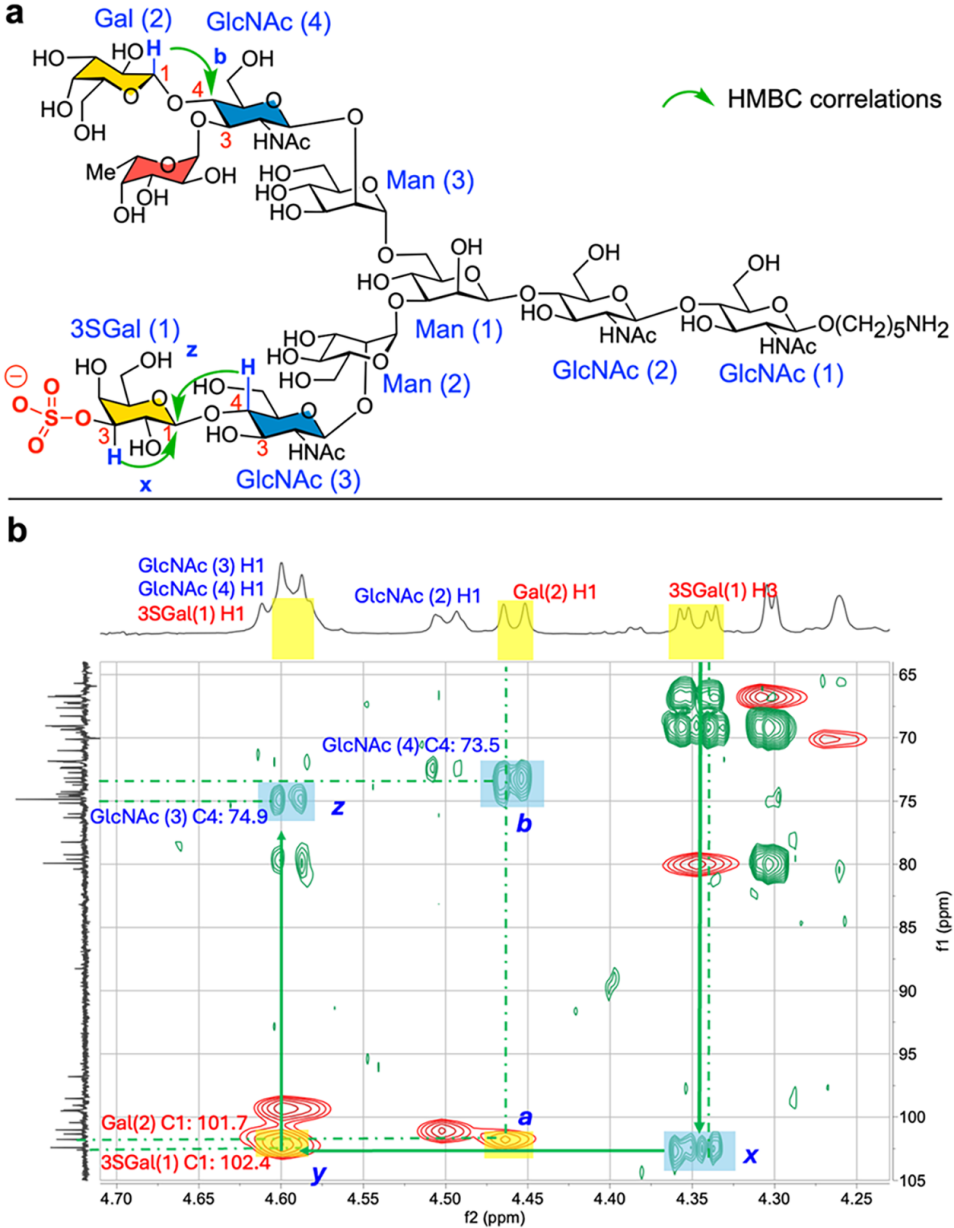
Elucidation of the fucosylation site of **36**: (a) numbering of **36** and HMCB correlations indicated by green arrows; (b) assignment of signature cross-peaks on the intercepted superimposed HMBC (green cross-peaks) and HSQC (red cross-peaks) spectra.

Figure 2b shows the excerpt region of the super imposed HMBC and HSQC spectra of **36**. The H3 of 3S-Gal (i.e., Gal 1) can be easily identified as a signature downfield doublet of doublets (4.34 ppm), which correlates cross-peak ***x*** in HMBC via three intramolecular bonds, to 3S-Gal C1 (102.4 ppm). The HSQC cross-peak of the latter can be identified as ***y*** (4.59 ppm, 102.4 ppm). Tracing vertically from ***y*** reaches HMBC cross-peak ***z*** (4.59 ppm, 74.9 ppm), presenting the correlation between Gal (1) H1 and GlcNAc (3) C4. The assignment of H1, C1 (4.46 ppm, 101.7 ppm) of Gal (2) (see Supporting Information for the assignment) enables the identification of GlcNAc (4) C4 at 73.5 ppm (i.e., from *a* to *b*), which shifts upfield compared to that of GlcNAc (3) C4 with Δ^13^C = −1.4 ppm. The shift difference indicates a β-glycosylation effect from the fucosyl substituent at GlcNAc (4) C3. Thus, the fucosylation site of **36** was confirmed, and the fucose position of **35** was verified via the same NMR analysis.

To our knowledge, the observed directed fucosylations of **34** is the first example of the application of human FuTs on 3′S-LacNAc in glycan synthesis. This approach could be further extended to glycans **23**, **24**, **27**, and **28** with sulfate or sialic acid as directing groups to furnish triantennary glycans possessing sulfate, sialic acid, and fucose moieties, adding valuable structure diversity to the glycan library.

## Conclusion

In this report, we established a conceptually simple yet powerful chemoenzymatic method for the synthesis of asymmetric multiantennary *N*-glycans, which could be practically adopted by biology-centered laboratories. Our strategy takes advantage of the (bio)­orthogonal sulfate and phosphate protecting groups, as well as optimized synthesis of the core glycan modules and their assembly. The key chemical steps for the synthesis of core intermediates include: (1) use of an *o*-alkynylbenzoate donor with directing group at C2 position for constructing Man-Man linkages with higher efficacy and exclusive α-selectivity; (2) use of sulfate 2,2,2-trichloroethyl (TCE) monoester as a masked, chemically manipulatable sulfate precursor; (3) careful protecting strategy design that not only rendered the base-labile phosphate and sulfate groups intact but also enabled the selective removal of five different types of protecting groups (25 total groups) in the molecule; and (4) a feasible hydrogenolytic dechlorination protocol that allows a single-step unmask of trichloroacetamide (TCA) to acetimide (NAc), a long-standing conundrum in carbohydrate chemistry.

The developed strategy for assembling asymmetric complex-type *N*-glycans enables direct and independent modification of either arm and is powered by the orthogonal sulfate and phosphate groups installed at 3*O*-Gal and 4*O*-GlcNAc, respectively, each paired with a highly selective hydrolase. Using this strategy, we streamlined the synthesis of dozens of *N*-glycans, including triantennary structures, and variants bearing naturally occurring 3S-Gal motif, thereby generating a broad panel of glycans with structural diversity not accessible by previously reported methods. The materials generated during our study provide valuable tools for probing biologically relevant questions.

Finally, we validated the utility of our strategy through the enzymatic synthesis of a series of complex-type *N*-glycans, including variants bearing the 3S-Gal modification. Based on this unique functionality, we demonstrated that the sulfation on the LacNAc motif could act as a switch for the pending fucosylation, depending on the fucosyltransferases used. Such observation might unveil the underlying differentiation mechanism in the biosynthesis of highly complex *N*-glycans. 3S-Gal can not only block fucosylation but also selectively direct fucosylation on the same antenna by using FuT-9 and FuT-6, respectively.

In perspective, by leveraging the simplicity of chemical installation of sulfate groups and the exquisite specificity of carbohydrate sulfatases, capable of distinguishing among sulfate positions on the same sugar, chemists could turn sulfate groups at different positions of sugars into orthogonal protecting groups, drastically reducing the burden of chemical synthesis of asymmetric multiantennary *N*-glycans. With the availability of glycan cores bearing the orthogonal handles for selective enzymatic deprotection and differential antennae elaboration, the assembly of asymmetric di-, tri-, and tetra-antennary *N*-glycans could become a routine procedure in biology-centered groups. We anticipate that the efficiency of chemical synthesis of the core intermediates, combined with the versatility of the subsequent enzymatic steps, will make our strategy broadly useful and widely adopted within the glycoscience community.

## Supplementary Material



## Notes

Experimental section; characterization data; NMR spectra; ^1^H NMR spectra of allyl-b-3-*O*-sulfo-galactoside (S1d) over 44.5 h show no migration; TCL record for reaction between S1 and 8b; and TCL records for preparing S2 from 14 and 9a/9b (PDF)
