# Supplementary material for: Chemoenzymatic Synthesis of Asymmetric N‑Glycans in Aqueous Solution via Sulfate and Phosphate Orthogonal Protection
Source: J Am Chem Soc. 2026 Jun 22;148(25):26446–56. doi: 10.1021/jacs.6c06320 (PMC13339134; doi:10.1021/jacs.6c06320)

Chemoenzymatic Synthesis of Asymmetric N-Glycans in Aqueous Solution via Sulfate and Phosphate Orthogonal Protection

Pengxi Chen, Ruofan Li, Yi-Fang Zeng, Tzu-Hao Tseng, Larissa Krasnova, Chi-Huey Wong*

Chemistry Department, The Scripps Research Institute, 10550 North Torrey Pines Road,

La Jolla, CA 92037, United States

Table of Contents

General Methods **7**
Supplemental Figures **8**
NMR Studies of O-acetylated and O-sulfated glycans 8
Figure S1. ^{1}H NMR spectra of S1a over 120 min. 8
Figure S2. ^{1}H NMR spectra of allyl-b-3-O-sulfo-galactoside (S1d) over 44.5 h show no migration. 9
TCL records of glycosylation reactions 10
Figure S3. TCL record for reaction between 14 and 8a. 10
Figure S4. TCL record for reaction between S1 and 8b. 10
Figure S5. TCL records for preparing S2 from 14 and 9a/9b. 11
1. 12
Experimental Procedures 12
General procedure I: preparation of glycosyl fluorides 12
General procedure II: glycosylation of thioglycosides, promoted by NIS and TMSOTf/TfOH 12
General procedure III: glycosylation of glycosyl fluorides promoted by Cp_2HfCl_2/ AgOTf 12
General procedure IV: glycosylation of ortho-alkynyl benzoates promoted by $Ph_3PAuOTf$ 13
General procedure V: glycosylation of oxazolines promoted by TESOTf 13
General procedure VI: removal of Fmoc group 13
General procedure VII: global deprotection of octasaccharides 13 and 16 13
Scheme S1. Synthesis of S9 15
Synthesis of S6 15
Synthesis of S7 16
Scheme S2. Synthesis of S9 from S8 17
Synthesis of S13 18
Synthesis towards 16 19
Synthesis of 10 19
Scheme S3. Synthesis of 5 20
Synthesis of 8a 20
Synthesis of S17 21
Synthesis of 5 22
Scheme S4. Synthesis of 6 24
Synthesis of **S18** 24
Synthesis of **S19** and **S20** 25
Synthesis of S21 26
Synthesis of 6 27
Scheme S5. Synthesis of 15 from 8 and 9 29
Scheme S6. Synthesis of 11 from 14 and 8a 29
Synthesis of 12 31
Synthesis of 13 32
Scheme S7. Synthesis of 14 33
Synthesis of S23 and S24 35
Scheme S8. Synthesis of 9a 37
Synthesis of S25 37
Synthesis of S26 39
Synthesis of 9a 40
Synthesis of 13 41
Synthesis of 19 42

Scheme S9. Synthesis of 16 from 14 via sequential glycosylation reactions with donors 9b, 7, and 8b 43
Synthesis of 9b 43
Synthesis of S2 45
Synthesis of 15 46
Synthesis of S27 47
Synthesis of S1 49
Synthesis of 8b 50
Synthesis of 16 51
Synthesis of 1 53
2. 54
Preparation of Enzymes 54
Table S1. Enzymes used in this work. 54
Commercial Sources of Sugar Donors and Enzymes 54
Experimental Procedures 54
General procedure VIII: enzymatic hydrolysis of phosphate 54
General procedure IX: enzymatic hydrolysis of sulfate 55
General procedure X: enzymatic ®1,3-N-acetylglucosaminyltransferase 55
General procedure XI: enzymatic ®1,4-galaccosylation 55
General procedure XII: enzymatic Synthesis of 〈2,3- and 〈2,6-sialylation 55
Enzymatic synthesis of 20 56
Enzymatic synthesis of 21 56
Enzymatic synthesis of 22 57
Enzymatic synthesis of 23 57
Enzymatic synthesis of 24 58
Enzymatic synthesis of 25 58
Enzymatic synthesis of 26 59
Enzymatic synthesis of 27 59
Enzymatic synthesis of 28 60
Enzymatic synthesis of 29 61
Enzymatic synthesis of 34 61
Enzymatic synthesis of 35 and 36 from 34 via three-steps synthesis 62
3. 65
4. 66
5. 72
S13 ^{1}H NMR spectrum 73
S13 DEPTQ135 ^{13}C NMR spectrum 74
S13 HSQC spectrum 75
5 ^{1}H NMR spectrum 76
5 DEPTQ135 ^{13}C NMR spectrum 77
5 HSQC spectrum 78
5 HSQC spectrum with splitting in F2 phase 79
5 ^{19}F NMR spectrum 80
5 ^{31}P NMR spectrum 81
6 ^{1}H spectrum 82
6 DEPTQ135 ^{13}C NMR spectrum 83
8a ^{1}H NMR spectrum 84

8a DEPTQ135 ^{13}C NMR spectrum 85
8a HSQC spectrum 86
10 ^{1}H NMR spectrum 87
10 DEPTQ 135 ^{13}C NMR spectrum 88
10 HSQC spectrum 89
11 ^{1}H NMR spectrum 90
11 DEPTQ135 ^{13}C NMR spectrum 91
11 HSQC spectrum 92
11 HSQC with splitting in F2 phase 93
12 ^{1}H NMR spectrum 94
12 DEPTQ135 ^{13}C NMR spectrum 95
12 HSQC spectrum 96
12 HSQC spectrum with splitting via F2 phase 97
13 ^{1}H spectrum 98
13 HSQC spectrum 99
14 ^{1}H NMR spectrum 100
14 DEPTQ135 ^{13}C NMR spectrum 101
14 HSQC spectrum 102
14 HSQC spectrum with splitting via F2 phase 103
S23 ^{1}H NMR spectrum 104
S23 DEPTQ135 ^{13}C NMR spectrum 105
S23 HSQC spectrum 106
S23 HSQC spectrum with splitting via F2 phase 107
S24 ^{1}H NMR spectrum 108
S24 DEPTQ135 ^{13}C NMR spectrum 109
S24 HSQC spectrum 110
S24 HSQC spectrum with splitting via F2 phase 111
9a ^{1}H NMR spectrum 112
9a HSQC spectrum 113
S17 ^{1}H NMR spectrum 114
S17 DEPTQ135 ^{13}C NMR spectrum 115
S17 ^{31}P spectrum 116
S17 HSQC spectrum 117
S18 ^{1}H NMR spectrum 118
S18 DEPTQ135 ^{13}C NMR spectrum 119
S18 HSQC spectrum 120
S19 ^{1}H spectrum 121
S19 DEPTQ135 ^{13}C NMR spectrum 122
S19 HSQC spectrum 123
S21 ^{1}H spectrum 124
S21 DEPTQ135 ^{13}C NMR spectrum 125
S21 HSQC spectrum 126
19 ^{1}H spectrum 127
19 DEPTQ135 ^{13}C NMR spectrum 128
19 HSQC spectrum 129
S25 ^{1}H spectrum 130
S25 ^{13}C NMR spectrum 131
S25 HSQC spectrum 132

S26 ^{1}H spectrum 133
S26 DEPTQ135 ^{13}C NMR spectrum 134
S26 HSQC spectrum 135
9b ^{1}H spectrum 136
9b DEPTQ135 ^{13}C NMR spectrum 137
9b HSQC spectrum 138
S2 ^{1}H spectrum 139
S2 HSQC spectrum 140
15 ^{1}H spectrum 141
15 HSQC spectrum 142
S27 ^{1}H spectrum 143
S27 DEPTQ135 ^{13}C NMR spectrum 144
S27 HSQC spectrum 145
S1 ^{1}H spectrum 146
S1 HSQC spectrum 147
8b ^{1}H spectrum 148
16 ^{1}H spectrum 149
16 DEPTQ135 ^{13}C NMR spectrum 150
16 HSQC spectrum 151
16 HSQC spectrum with splitting via F2 phase 152
1 ^{1}H spectrum 153
1 DEPTQ135 ^{13}C NMR spectrum 154
1 HSQC spectrum 155
1 HSQC spectrum with splitting via F2 phase 156
1 superimposed HSQC spectra 157
1 COSY spectrum 158
1 TOCSY-HSQC spectrum 159
1 HMBC spectrum 160
21 ^{1}H spectrum 161
21 HSQC spectrum 162
22 ^{1}H NMR spectrum 163
22 HSQC spectrum 164
25 ^{1}H NMR spectrum 165
25 DEPTQ135 ^{13}C NMR spectrum 166
25 HSQC spectrum 167
25 HSQC spectrum with splitting via F2 phase 168
26 ^{1}H NMR spectrum 169
26 HSQC spectrum 170
27 ^{1}H NMR spectrum 171
27 HSQC spectrum 172
29 ^{1}H NMR spectrum 173
29 HSQC spectrum 174
34 ^{1}H NMR spectrum 175
34 HSQC spectrum 176
35 ^{1}H NMR spectrum 177
35 DEPTQ135 ^{13}C NMR spectrum 178
35 TOCSY spectrum 179
35 COSY spectrum 180

35 HSQC spectrum 181
35 HMBC spectrum 182
35 H2BC spectrum 183
36 ^{1}H NMR spectrum 184
36 DEPTQ135 ^{13}C NMR spectrum 185
36 HSQC spectrum 186
36 HSQC spectrum with splitting via F2 phase 187
36 HMBC spectrum 188
36 TOCSY spectrum 189
36 H2BC spectrum 190
36 HMBC spectrum 8
36 TOCSY spectrum 9
36 H2BC spectrum 80

General Methods

All the organic reactions were performed under inert atmosphere (nitrogen or argon) using anhydrous solvents unless otherwise noted. All chemicals were purchased as reagent grade and used without further purification. Anhydrous dichloromethane (CH_2Cl_2), acetonitrile (CH_3CN), toluene, methanol (MeOH), tetrahydrofuran (THF), *tert*-butanol (*t*-BuOH), *n*-butanol (*n*-BuOH) were purchased from commercial sources and used without further distillation.

All the chemical reactions were monitored by analytical thin-layer chromatography (TLC) using silica gel 60 F254 plates and visualized under UV (254 nm) and/or by acidic ceric ammonium molybdate (CAM), *p*-anisaldehyde, or $KMnO_4$. Enzymatic reactions were monitored by TLC or LC/MS (Agilent).

Flash chromatography was performed on silica gel (Fischer Chemical or Silicycle Inc.) of 40-63 µm particle size. Size exclusive chromatography (SEC) was performed on P2 gel (Bio-rad, fine, 45-90 µm) or Sephadex G-25 (Cytiva, fine).

NMR spectra were recorded with samples in 5mm NMR tubes at 25 °C on NMR spectrometers of Bruker Avance NEO 399 (5 mm BBFO probe), AVIII 400 (5 mm BBFO probe), Avance NEO 500 (5 mm BBFO probe), AVIII HD 600 [5 mm CPQCI (H-C-N-P) CryoProbe and 5 mm CPDCH (C-H) CryoProbe], and AVIII 700 (CPTCI Cryoprobe). NMR data are presented as follows: chemical shift, splitting patterns, coupling constant. Splitting patterns are described using the following abbreviations: s, singlet; brs, broad singlet; d, doublet; t, triplet; q, quartet; quint, quintet; hept, heptet; dd, doublet of doublets; m, multiplet. Coupling constant are reported in Hertz (Hz). All ^{1}H chemical shifts were calibrated using solvent residue peaks of $CDCl_3$ (δ = 7.26 ppm, s), MeOD (δ = 3.31 ppm, quint) and D_2O (δ = 4.79 ppm, s).

High resolution ESI mass spectra were recorded on an Agilent 6230B time-of-flight LC-TOF spectrometer.

Supplemental Figures

NMR Studies of *O*-acetylated and *O*-sulfated glycans

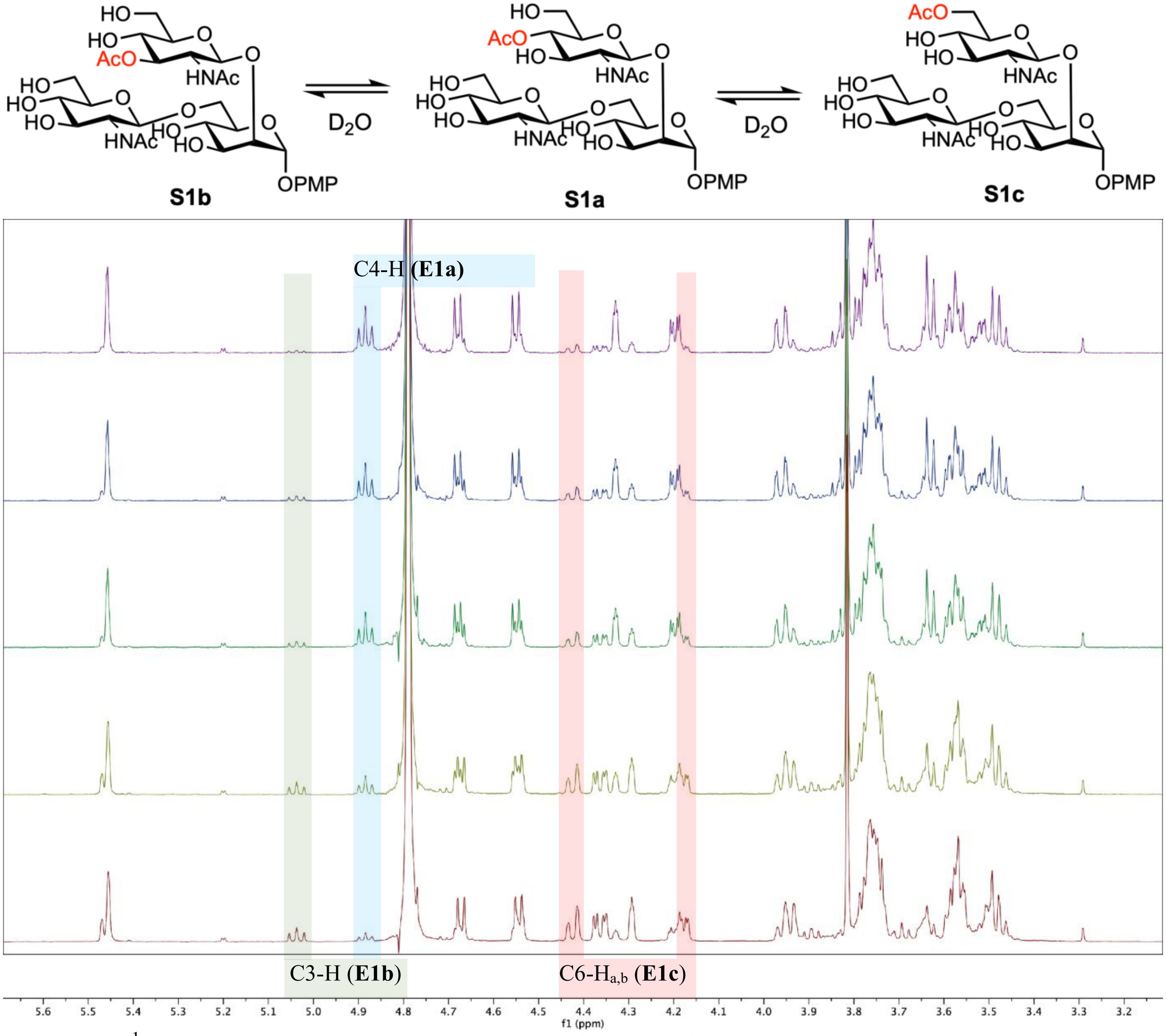

Figure S1. ^{1}H NMR spectra of S1a over 120 min. The stacked NMR spectra show the migration of acetate to form a mixture consisting of 3'*O*-acetate (**S1b**), 4'*O*-acetate (**S1a**), and 6'*O*-acetate (**S1c**): from top to bottom: t = 5, 15, 20, 60, and 120 min (25 mM PBS in D_2O, *p*D = 7.9, 22 °C).

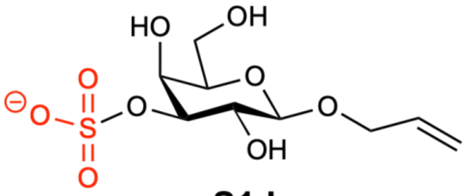

S1d

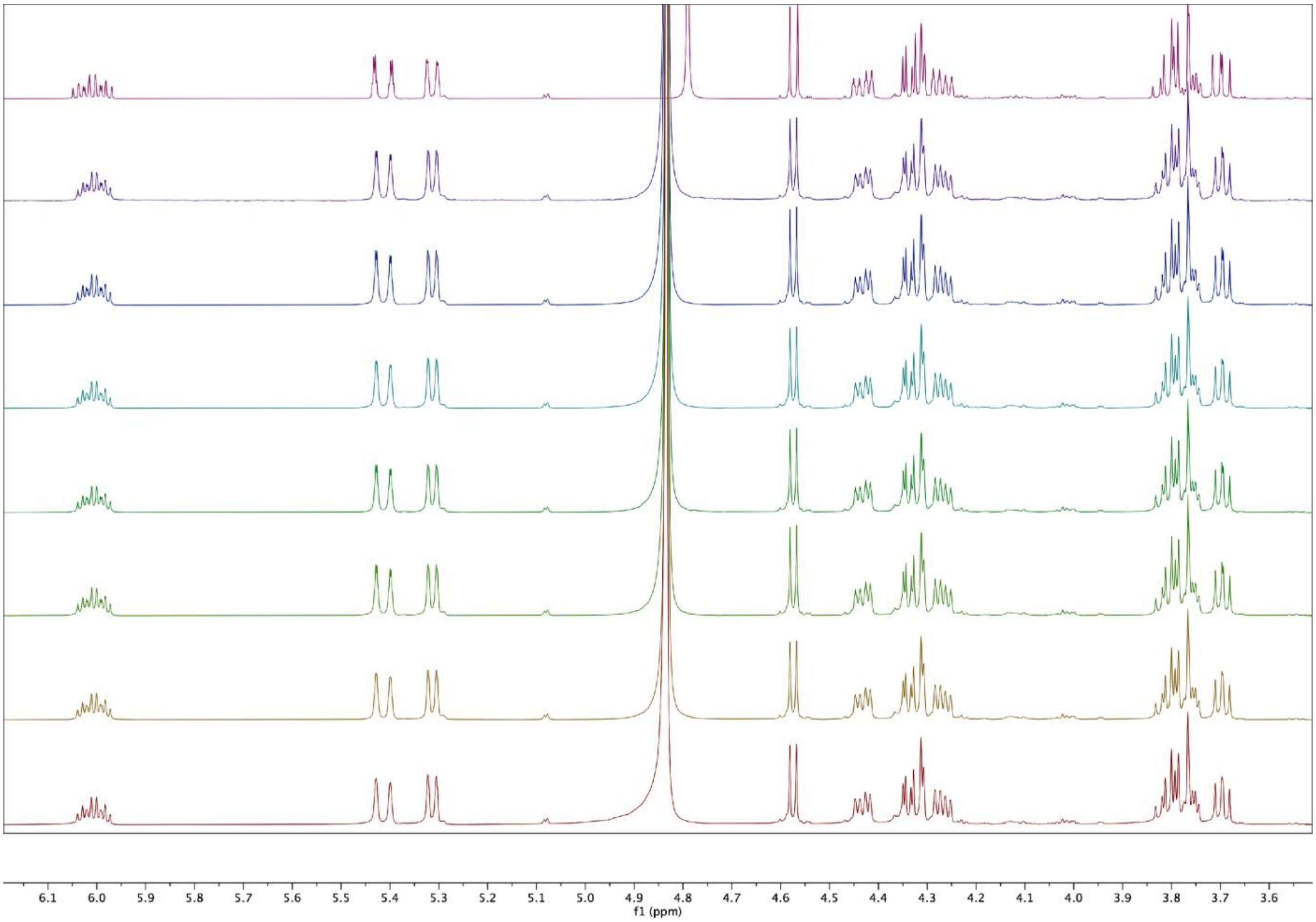

Figure S2. ^{1}H NMR spectra of allyl-b-3-*O*-sulfo-galactoside (S1d) over 44.5 h show no migration. From top to bottom: *t* = 15 min, 50 min, 2 h, 4 h, 8 h, 16 h, and 44.5 h (25 mM PBS in D_2O, *p*D = 7.9, 22 °C

TCL records of glycosylation reactions

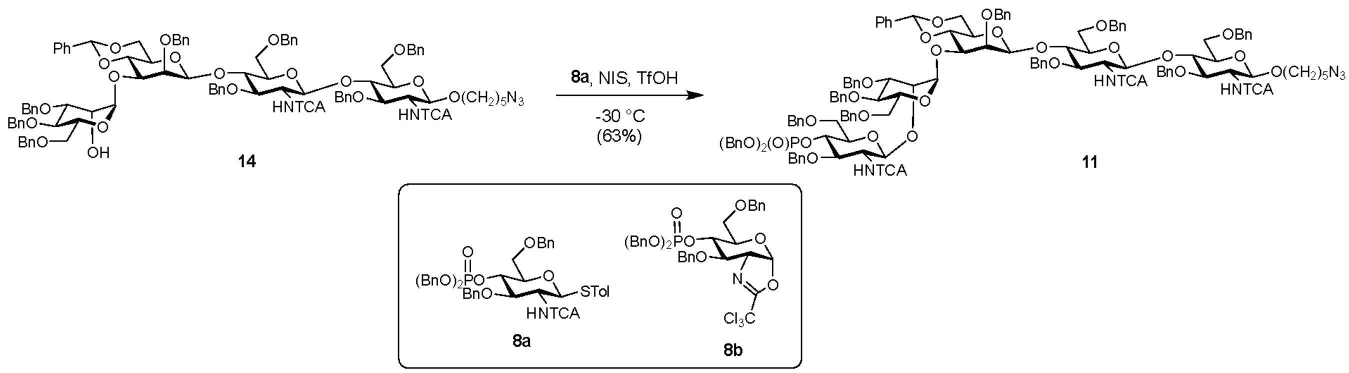

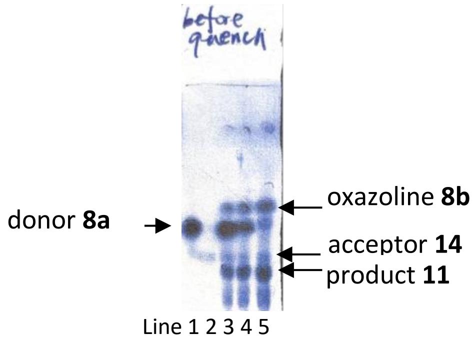

developing solvent: hexane/acetone 2/1.
Line 1: donor **8a**
Line 2: acceptor **14**
Line 3: co-spot of **14**, **8a**, and reaction mixture
Line 4: co-spot of **14** and reaction mixture
Line 5: reaction mixture

Figure S3. TCL record for reaction between 14 and 8a.

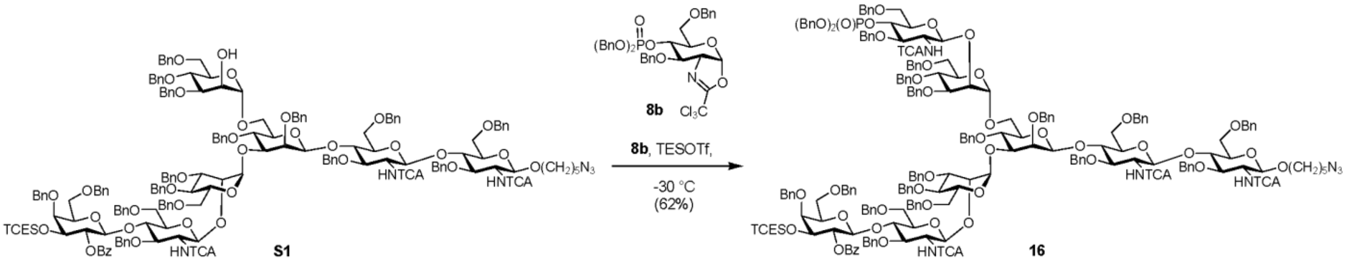

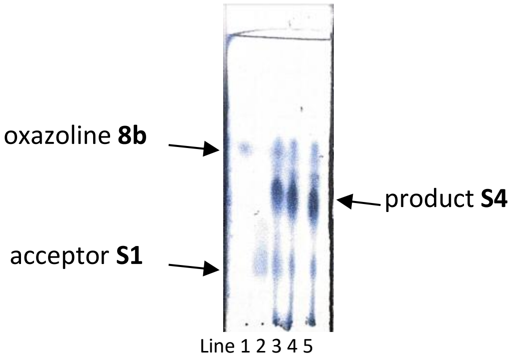

developing solvent: toluene/EtOAc 5/1.
Line 1: donor **8b**
Line 2: acceptor **S1**
Line 3: co-spot of **8b**, **S1**, and reaction mixture
Lines 4, 5: reaction mixture

Figure S4. TCL record for reaction between S1 and 8b.

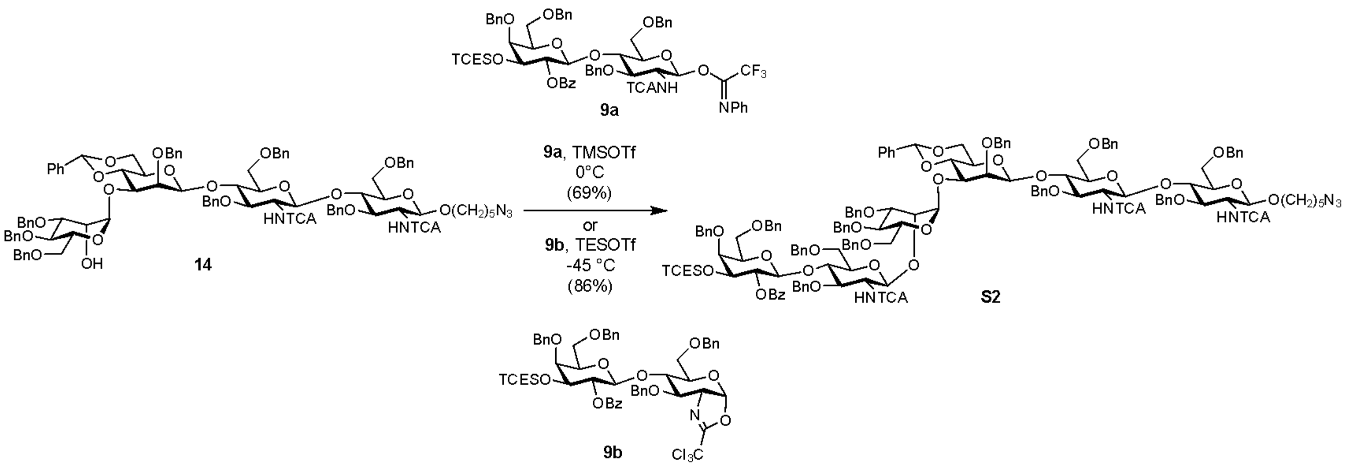

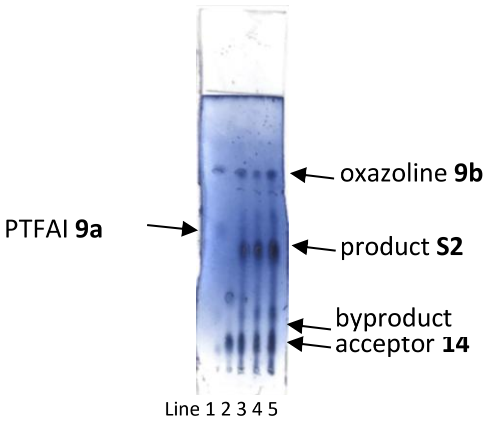

Reaction of **14** and **9a**
developing solvent: toluene/EtOAc 5/1

Line 1*: donors **9a** and **9b**, acceptor **14**
Line 2: acceptor **14**
Line 3: co-spot of **9a**, **14**, and reaction mixture
Lines 4, 5: reaction mixture

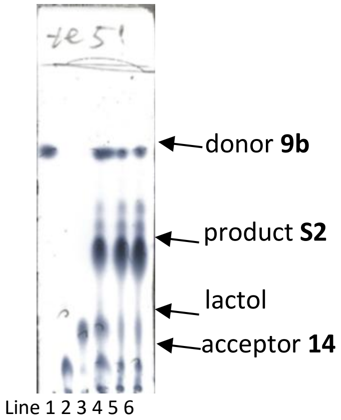

Reaction of **14** and **9b**
developing solvent: toluene/EtOAc 5/1

Line 1: donor **9b**
Line 2: acceptor **14**
Line 3: lactol (hydrolysis product of **9b**)
Line 4: co-spot of **9b**, **14**, and reaction mixture
Lines 5, 6: reaction mixture

Figure S5. TCL records for preparing S2 from 14 and 9a/9b.

1. Chemical Synthesis

Experimental Procedures

General procedure I: preparation of glycosyl fluorides

To a mixture of the substrate and 4Å MS (100 mg per 1 mL solvent) in CH_2Cl_2 (0.1 M) at −50 °C was added DAST (2.0 equiv.). The reaction mixture was allowed to stir at the same temperature for 30 min and then filtered through Celite®. The filtrate was washed with saturated $NaHCO_3$ (aq.), dried over anhydrous Na_2SO_4 and concentrated under vacuum. The residue was purified on a short silica gel column with hexanes/EtOAc as eluent to afford desired glycosyl fluoride.

General procedure II: glycosylation of thioglycosides, promoted by NIS and TMSOTf/TfOH

A mixture of thioglycoside donor (1.2 – 2.0 equiv., depending on the reaction), acceptor (1.0 equiv.), and flame-dried 4Å molecular sieves (100 mg/mL) in CH_2Cl_2 were stirred at −50 °C for 5 min before NIS (1.2 equiv to donor) and TfOH (0.1 – 0.2 equiv., depending on the reaction) were added. The reaction mixture was stirred at proper temperature for 30 min – 1 hour (depending on the reaction) before it was lowered to below −70 °C and quenched by sequential addition of Et_3N, $NaHCO_3$ (sat. aq.) and $Na_2S_2O_3$ (s). The cooling bath was removed, and the resulting mixture was stirred until its yellow color faded to white. The resulting mixture was filtered to remove 4Å molecular sieves, diluted with CH_2Cl_2 and H_2O and extracted with CH_2Cl_2. The organic phases were combined, dried over Na_2SO_4, filtered, and concentrated under vacuum. The resulting residue was purified by flash chromatography (silica gel) to afford the glycosylation product.

General procedure III: glycosylation of glycosyl fluorides promoted by Cp_2HfCl_2/ AgOTf

To a mixture of Cp_2HfCl_2 (3.5 equiv.), AgOTf (5.0 equiv.) and 4Å MS (1.5 g per 1.0 mmol Hf, flame dried) with vigorous stirring was added toluene (*ca.* 0.07 M Hf) at room temperature. The suspension was stirred for another 3 – 8 hrs before it was cooled to −60 °C, at which point a solution of saccharyl fluoride donor (1.2 equiv.) and acceptor (1.0 equiv.) in PhMe was slowly added into the suspension. The

reaction mixture was slowly warmed up to −30 °C over 1 hr and then quenched by saturated aqueous $NaHCO_3$ and diluted with EtOAc. The resulting suspension was filtered through Celite® and the organic layer of the filtrate was separated. The aqueous layer was extracted with EtOAc and the organic layers were combined, dried over Na_2SO_4, and concentrated under vacuum. The residue was purified by flash chromatography (silica gel) to afford the desired glycosylation product.

General procedure IV: glycosylation of ortho-alkynyl benzoates promoted by $Ph_3PAuOTf$

To a flame-dried flask under argon were sequentially added AgOTf (1.0 equiv.) and Ph_3PAuCl (1.0 equiv.) and the white mixture was stirred under vacuum for 15 min before CH_2Cl_2 (4.0 mL per 0.2 mmol catalyst) was added under argon. The flask was then covered with aluminum foil and the suspension continued stirring at room temperature for another 2 hrs. The resulting slightly purplish suspension was allowed to sediment for 15 min and the supernatant, which contained *ca.* 0.05 M $Ph_3PAuOTf$, was ready to be used for the pending Au(I)-promoted glycosylation reactions.

A mixture of *o*-alkynyl benzoate donor (1.2 to 3.0 equiv., depending on the reaction), acceptor (1.0 equiv.) and flame-dried 4Å molecular sieves (100 mg/mL) in CH_2Cl_2 (final concentration of acceptor: 0.02 M) were stirred at 0 °C for 30 min before a solution of $Ph_3PAuOTf$ (ca. 0.05 M in CH_2Cl_2, 0.2 to 1.0 equiv., depending on the reaction) was added. The resulting mixture was allowed to stir at 0 °C for another 30 min and then directly subjected onto a flash column (silica gel). The desired product was purified using proper eluent.

General procedure V: glycosylation of oxazolines promoted by TESOTf

To a mixture of oxazoline (1.2 equiv.), acceptor (1.0 equiv.), flame-dried 4Å molecular sieves (100 mg/mL) in toluene (acceptor concentration 0.03M) at −50 °C was added TESOTf (0.2 equiv). The reaction mixture was stirred at −50 °C – −4 0°C for 40 min before a mixture of MeOH/Et_3N 1/1 and $NaHCO_3$ (sat.) added at below −70 °C and then warmed to 22 °C. The resulting mixture was filtered to remove 4Å molecular sieves, diluted with CH_2Cl_2 and H_2O and extracted with CH_2Cl_2. The organic phases were combined, dried over Na_2SO_4, filtered, and concentrated under vacuum. The residue was purified by flash chromatography (silica gel) to afford the glycosylation product.

General procedure VI: removal of Fmoc group

A solution of Fmoc carbonate substrate (1.0 equiv.) in CH_2Cl_2:Et_3N (10:1, v/v, final concentration of the substrate: 30-40 mg/mL) at room temperature was stirred for 12 hrs before all the volatiles were removed under vacuum. The residue was then purified by flash chromatography using proper eluents to afford desired alcohol.

General procedure VII: global deprotection of octasaccharides 13 and 16

Amberlite IR-120 (H) ion resin was pre-washed with H_2O until the eluent became colorless.

The solution of the substrate in THF/H_2O/*t*-BuOH (6/3/1, *v*/*v*/*v*, substrate concentration *ca*. 20 mg/mL) was degassed and backfilled with argon for three times before 20% $Pd(OH)_2$/C (100% *w/w*) was added. The reaction system was then switched to a hydrogen atmosphere using a H_2 balloon and was added Et_3N (30 qeuiv.). The reaction mixture was stirred at 22 °C for 12 hrs, filtered and concentrated under vacuum to remove Et_3N to give partially debenzylated crude product. The resulting crude was again dissolved in THF/H_2O/*t*-BuOH/AcOH (6/3/1/0.2 *v*/*v*/*v*/*v*, substrate concentration *ca.* 15 mg/mL) and subjected to hydrogenolysis with 20% $Pd(OH)_2$/C (100% *w/w*) and H_2 balloon. The reaction was stirred for another 24 hrs and was then filtered and concentrated under vacuum to give a glass-like crude, which was redissolved in H_2O (substrate concentration *ca.* 15 mg/mL) and treated with aq. 1N NaOH (final concentration *ca*. 0.2 M) at 22 °C. The reaction mixture was neutralized by Amberlite IR-120 (H) upon completion and the resulting suspension was filtered, concentrated under vacuum and purified by P2 gel chromatography (dH_2O as eluent). The fraction containing the product was lyophilized to afford the product.

Scheme S1. Synthesis of S9

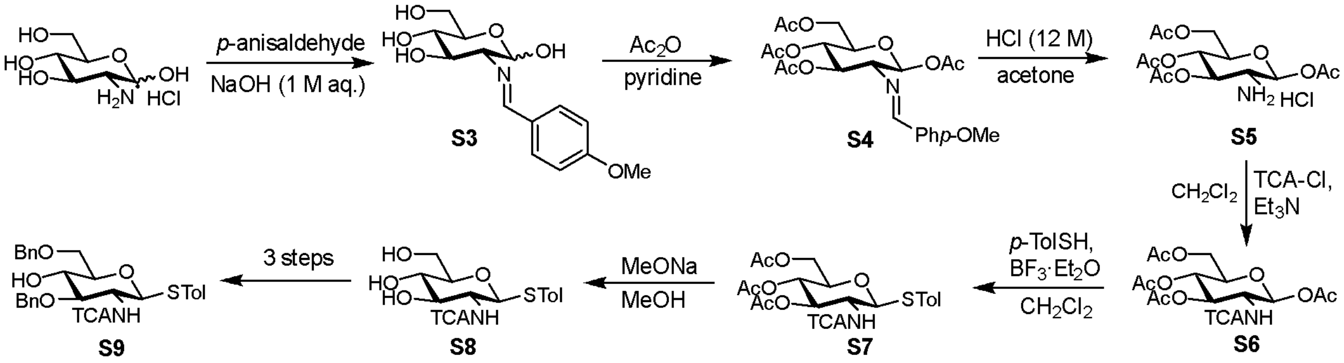

S3 to **S5** were prepared following the procedure in the literature.[1]

Synthesis of S6

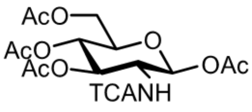

β-1,3,4,6-tetra-*O*-acetyl-2-(trichloroacetamido)-2-deoxy-D-glucopyranose (S6):

To the solution of **S5** (7.80 g, 20.3 mmol, 1.0 equiv.) in CH_2Cl_2 (150 mL) at 0 °C was added Et_3N (5.70 mL, 41.0 mmol, 2.0 equiv.) and trichloroacetyl chloride (2.9 mL, 25.6 mmol, 1.3 equiv.) and the reaction mixture was stirred for 40 min before $NaHCO_3$ (sat. aq.) was added. The resulting mixture was diluted with CH_2Cl_2 and H_2O and extracted with CH_2Cl_2. The organic phases were combined, dried over Na_2SO_4, filtered, and concentrated under vacuum. The resulting residue was recrystallized from hexane/EA to afford **S6** as white powders (8.74 g, 17.8 mmol, 89%).

NMR data of **S6** were in accordance with that in the literature.[1]

S6: ^{1}H NMR (500 MHz, MeOD-d4) δ 5.91 (d, *J* = 8.8 Hz, 1H), 5.39 (dd, *J* = 10.5, 9.1 Hz, 1H), 4.33 (dd, *J* = 12.6, 4.6 Hz, 1H), 4.14 (dd, *J* = 12.6, 2.3 Hz, 1H), 4.05 (ddd, *J* = 10.1, 4.5, 2.3 Hz, 1H), 3.66 (dd, *J* = 10.5, 8.8 Hz, 1H), 3.33 (p, *J* = 1.6 Hz, 1H), 2.22 (s, 3H), 2.12 (s, 3H), 2.05 (6H) ppm.

^{13}C NMR (126 MHz, MeOD-d4) δ 170.71, 170.52, 169.72, 168.65, 90.23, 72.59, 70.74, 67.82, 61.17, 52.95, 19.33, 19.24, 19.12, 19.07 ppm.

Synthesis of S7

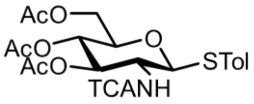

S7

***p*-Tolyl 2-(trichloroacetamido)-2-deoxy-3,4,6-tri-*O*-acetyl-1-thio-β-D-glucopyranoside (S7):**

To the mixture of **S6** (12.0 g, 24.4 mmol, 1.0 equiv.) and *p*-toluenethiol (3.6 g, 29.3 mmol, 1.2 equiv.) in CH_2Cl_2 (200 mL) at 22 °C was added BF_3 · Et_2O (6.1 mL, 38.3 mmol, 1.6 equiv.). The reaction mixture was stirred overnight before it was quenched by $NaHCO_3$ (sat. aq.). The resulting mixture was washed with 1N NaOH (aq.) until remained *p*-toluenethiol was removed from the organic phase. The organic phase was dried over anhydrous Na_2SO_4, filtered, and concentrated under vacuum to give crude **S7**, which was dissolved with minimum amount of CH_2Cl_2 and then precipitated from addition of hexane. **S7** was obtained as white powders (11.6 g, 21.0 mmol, 86%).

NMR data of **S7** were in accordance with that in the literature.[2]

Scheme S2. Synthesis of S9 from S8

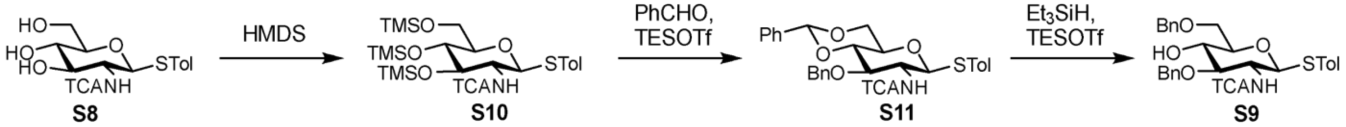

S9 was prepared from **S8** referring to the work by Wang and co-workers: [2]

***p*-Tolyl 2-(trichloroacetamido)-2-deoxy-3,6-di-*O*-benzyl-1-thio-β-D-glucopyranoside (S9)**

To a suspension of **S8** (1.17 g, 2.73 mmol, 1.0 equiv.) in CH_2Cl_2 (20 mL) at 22 °C were added with HMDS (1.1 mL, 5.5 mmol, 2.0 equiv.) and TMSOTf (50 µL, 0.28 mmol, 0.1 equiv.). The suspension gradually dissolved, and the reaction completed in 1 hour. The reaction mixture was washed with $NaHCO_3$ (sat. aq) and the organic phase was dried over anhydrous Na_2SO_4, filtered, and dried under vacuum to give the crude per-trimethylsilylated product **S10**, which was used without further purification.

To a solution of **S10** in CH_2Cl_2 (12 mL) at 0 °C was added benzaldehyde (830 µL, 8.20 mmol, 3.0 equiv.), Et_3SiH (525 µL, 3.30 mmol, 1.2 equiv.) and TMSOTf (50 µL, 0.81 mmol, 0.1 equiv.). White precipitation formed (presumably **S11**) and the mixture was stirred for 20 min and then MeCN (6 mL) was added, followed by Et_3SiH (2.20 mL, 13.7 mmol, 5.0 equiv.) and TMSOTf (50 µL, 0.81 mmol, 0.1 equiv.). The reaction mixture was stirred at 0 °C for 40 min. The reaction mixture was quenched with $NaHCO_3$ (sat. aq), extracted with CH_2Cl_2. The combined organic layers were dried over anhydrous Na_2SO_4, filtered, concentrated under vacuum to give a residue, which was dissolved with minimum amount of CH_2Cl_2 and then precipitated from addition of hexane. **S9** was obtained as white powders (1.3 g, 2.1 mmol, 76%).

S9: ^{1}H NMR (600 MHz, $CDCl_3$) δ 7.45 – 7.30 (m, 12H), 7.09 (d, *J* = 7.8 Hz, 2H), 6.86 (d, *J* = 7.8 Hz, 1H), 5.14 (d, *J* = 10.3 Hz, 1H), 4.93 – 4.70 (m, 2H), 4.70 – 4.54 (m, 2H), 4.01 (dd, *J* = 10.1, 8.6 Hz, 1H), 3.91 – 3.83 (m, 1H), 3.79 (dd, *J* = 10.3, 4.8 Hz, 1H), 3.71 (dd, *J* = 9.6, 8.5 Hz, 1H), 3.59 (dt, *J* = 9.5, 4.8 Hz, 1H), 3.46 (td, *J* = 10.1, 7.8 Hz, 1H), 2.34 (s, 3H) ppm.

^{13}C NMR (151 MHz, $CDCl_3$) δ 161.47, 138.73, 137.82, 137.66, 133.81, 129.86, 128.66, 128.52, 128.19, 128.12, 127.91, 127.78, 127.73, 84.70, 81.06, 77.91, 75.13, 73.36, 70.58, 56.58, 21.17 ppm.

Synthesis of S13

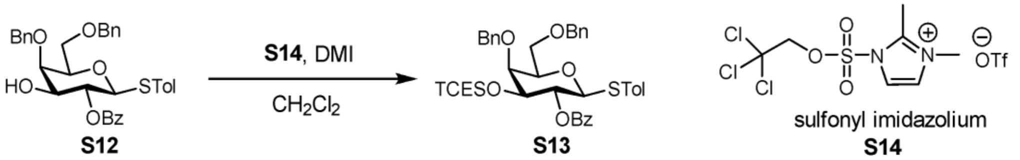

***p*-Tolyl 2-*O*-benzoyl-4,6-di-*O*-benzyl-3-*O*-(2,2,2-trichloroethoxysulfonyl)-1-thio-β-D-galactopyranoside (S13):**

The preparation of **S13** was referred to the known literature.[3] The sulfation reagent **S14** was prepared according to Ingram and Taylor.[4]

To the mixture of **S12** (193 mg, 0.34 mmol, 1.0 equiv.) and sulfonyl imidazolium **S14** (233 mg, 0.51 mmol, 1.5 equiv.) in CH_2Cl_2 (3 mL) under N_2 atmosphere was added a solution of 1,2-dimethylimidazole (DMI, 98 mg, 1.0 mmol, 3.0 equiv., 1 M in CH_2Cl_2) at 22 °C. The resulting mixture was stirred overnight before it was diluted with CH_2Cl_2 and partitioned between CH_2Cl_2 and H_2O. The combined organic layers were dried over anhydrous Na_2SO_4, filtered, and dried under vacuum. The resulting residue was purified on a short column (silica gel, hexane/EtOAc) to afford **S13** as white powders (231 mg, 0.29 mmol, 87%).

Note: CH_2Cl_2 was added to the eluent in the scenario when **S13** precipitated in the column.

S13: ^{1}H NMR (500 MHz, $CDCl_3$) δ = 8.11 – 8.05 (m, 2H), 7.64 – 7.57 (m, 1H), 7.47 (t, *J* = 7.9 Hz, 2H), 7.39 – 7.27 (m, 12H), 7.02 (d, *J* = 7.7 Hz, 2H), 5.67 (t, *J* = 9.8 Hz, 1H), 4.98 (dd, *J* = 9.8, 2.9 Hz, 1H), 4.89 (d, *J* = 11.6 Hz, 1H), 4.75 (dd, *J* = 9.8, 1.3 Hz, 1H), 4.61 (d, *J* = 11.6 Hz, 1H), 4.51 – 4.41 (m, 3H), 4.36 (d, *J* = 3.0 Hz, 1H), 4.29 (dd, *J* = 10.8, 1.3 Hz, 1H), 3.77 (t, *J* = 6.5 Hz, 1H), 3.69 (dd, *J* = 9.3, 5.6 Hz, 1H), 3.62 (dd, *J* = 9.4, 7.4 Hz, 1H), 2.31 (s, 3H) ppm.

^{13}C NMR (126 MHz, $CDCl_3$) δ = 165.1, 138.5, 137.8, 137.7, 133.8, 133.4, 130.2, 129.8, 128.8, 128.6, 128.5, 128.4, 128.1, 128.04, 127.96, 127.9, 92.3, 86.9, 85.9, 79.8, 77.2, 75.4, 74.5, 73.7, 68.2, 68.0, 21.3 ppm.

Synthesis towards 16

Synthesis of 10

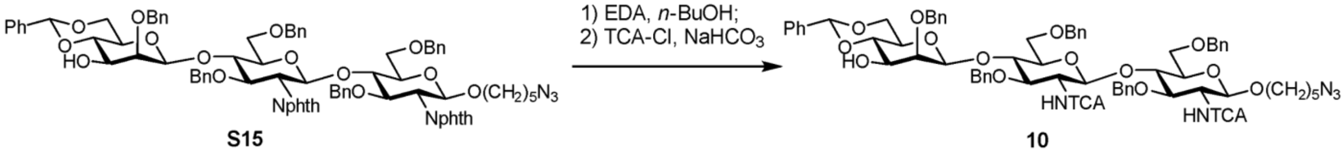

5-Azidopentyl 2-*O*-benzyl-4,6-benzylidene-β-D-mannopyranosyl-(1 -> 4)-3,6-di-*O*-benzyl-2-deoxy-2-(trichloroacetamido)-β-D-glucopyranosyl-(1 -> 4)-3,6-di-*O*-benzyl-2-deoxy-2-(trichloroacetamido)-β-D-glucopyranoside (10):

A solution of **S15**[3] (550 mg, 0.41 mmol) in *n*-BuOH/ethylenediamine (4:1, v/v, 25 mg/mL) was stirred at 90 °C for 18 hrs. The reaction mixture was cooled to room temperature, concentrated under vacuum to give a crude which was purified by flash chromatography to give the deprotected product as white powders (440 mg). The obtained intermediate was suspended in CH_2Cl_2/$NaHCO_3$ (1.05 M) (7.2 mL/2.4 mL). To the mixture was added trichloroacetyl chloride (135 μL, 1.2 mmol, 3.0 equiv.) at 0 °C and the reaction mixture was stirred for 1hr before it was diluted and extracted with CH_2Cl_2. The organic phases were combined, dried over Na_2SO_4, filtered, and concentrated under vacuum. The resulting residue was purified by flash chromatography (silica gel, toluene/EtOAc) to afford **10** (450 mg, 0.31 mmol, 76% over 2 steps).

10: ^{1}H NMR (600 MHz, $CDCl_3$) δ = 7.49 – 7.43 (m, 2H), 7.41 – 7.17 (m, 28H), 6.92 (d, *J* = 7.7 Hz, 1H), 6.64 (d, *J* = 7.9 Hz, 1H), 5.44 (s, 1H), 5.07 (d, *J* = 10.5 Hz, 1H), 4.99 (d, *J* = 11.5 Hz, 1H), 4.96 (d, *J* = 11.4 Hz, 1H), 4.82 (d, *J* = 8.3 Hz, 1H), 4.80 (d, *J* = 7.7 Hz, 1H), 4.72 (d, *J* = 12.0 Hz, 1H), 4.65 (d, *J* = 11.5 Hz, 1H), 4.63 – 4.57 (m, 2H), 4.55 – 4.50 (m, 2H), 4.46 (d, *J* = 10.4 Hz, 1H), 4.31 (d, *J* = 12.0 Hz, 1H), 4.17 (t, *J* = 8.1 Hz, 1H), 4.07 (dd, *J* = 10.6, 5.0 Hz, 1H), 4.04 – 3.97 (m, 2H), 3.87 (dt, *J* = 9.6, 6.2 Hz, 1H), 3.78 – 3.65 (m, 5H), 3.63 – 3.40 (m, 8H), 3.26 – 3.17 (m, 3H), 3.05 (td, *J* = 9.7, 4.9 Hz, 1H), 2.31 (br s, *J* = 8.6 Hz, 1H), 1.65 – 1.52 (m, 4H), 1.48 – 1.33 (m, 2H) ppm.

^{13}C NMR (151 MHz, $CDCl_3$) δ = 161.8, 161.7, 138.32, 138.28, 138.1, 138.0, 137.5, 137.2, 129.2, 128.7, 128.6, 128.5, 128.35, 128.32, 128.30, 128.22, 128.18, 128.05, 128.00, 127.93, 127.87, 127.7, 127.6, 126.3, 102.0, 101.6, 99.5, 98.3, 92.5, 79.1, 78.9, 78.2, 78.0, 77.9, 75.9, 75.3, 74.9, 74.73, 74.69, 74.4, 73.6, 73.2, 70.8, 69.5, 68.39, 68.37, 68.3, 66.9, 58.6, 57.4, 51.3, 29.0, 28.6, 23.2 ppm.

Scheme S3. Synthesis of 5

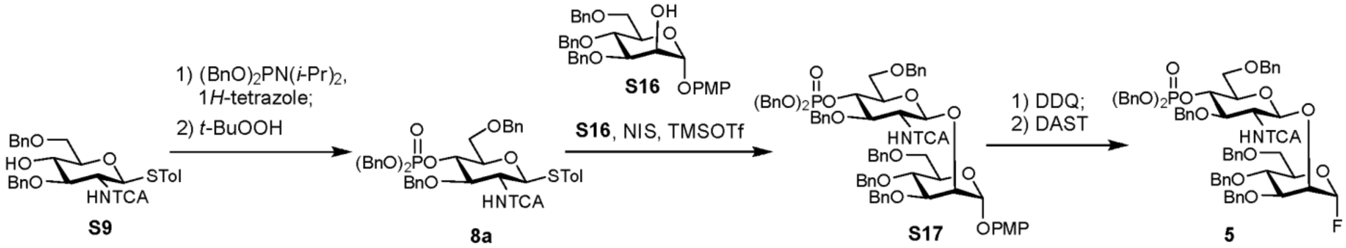

Synthesis of 8a

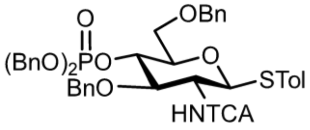

8a

***p*-Tolyl 4-*O*-dibenzylphosphoryl-3,6-di-*O*-benzyl-2-deoxy-2-(trichloroacetamido)-1-thio-β-D-glucopyranoside (8a):**

To a solution of **S9** (518 mg, 0.85 mmol, 1.0 equiv.) in CH_2Cl_2 (8 mL) at 0 °C were added dibenzyl *N*,*N*-diisopropylphosphoramidate (450 μL, 1.36 mmol, 1.6 equiv.) and 1*H*-tetrazole (149 mg, 2.13 mmol, 2.5 equiv.). The resulting mixture was stirred at this temperature for 1 hr for completion, upon which *tert*-butyl hydroperoxide (120 μL, 0.95 mmol, 1.25 equiv., 70%w/w in H_2O) was added. The reaction mixture was stirred and allowed to warm to 22 °C over 30 min before it was partitioned with CH_2Cl_2 and H_2O. The combined organic layers were concentrated at reduced pressure to give a crude which was purified by fast chromatography (silica gel, toluene/EtOAc) to give **8a** as a colorless oil (528 mg, 73%).

8a: ^{1}H NMR (600 MHz, $CDCl_3$) δ = 7.40 – 7.36 (m, 2H), 7.29 – 7.16 (m, 15H), 7.15 – 7.11 (m, 2H), 7.09 – 7.06 (m, 2H), 7.03 (d, *J* = 7.9 Hz, 1H), 6.98 (d, *J* = 7.9 Hz, 2H), 5.09 (d, *J* = 10.1 Hz, 1H), 4.87 – 4.81 (m, 3H), 4.80 – 4.72 (m, 2H), 4.56 (d, *J* = 10.4 Hz, 1H), 4.48 (d, *J* = 11.9 Hz, 1H), 4.46 – 4.38 (m, 2H), 4.19 (dd, *J* = 9.9, 8.7 Hz, 1H), 3.81 (dd, *J* = 10.8, 1.8 Hz, 1H), 3.71 – 3.61 (m, 2H), 3.50 (td, *J* = 10.0, 7.8 Hz, 1H), 2.26 (s, 3H) ppm.

^{13}C NMR (151 MHz, $CDCl_3$) δ = 161.7, 138.8, 138.5, 137.6, 135.74, 135.69, 135.67, 135.6, 133.8, 130.0, 128.67, 128.66, 128.62, 128.61, 128.5, 128.4, 128.1, 128.04, 128.02, 127.9, 127.8, 127.65, 127.60, 92.5, 84.5, 79.8 (d, *J* = 3.5 Hz), 78.8 (d, *J* = 4.3 Hz), 76.2 (d, *J* = 6.8 Hz), 74.6, 73.4, 69.8 (d, *J* = 5.5 Hz), 69.7 (d, *J* = 5.5 Hz), 68.9, 56.7, 21.3 ppm.

Synthesis of S17

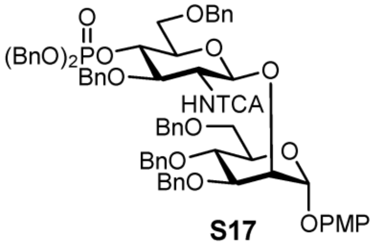

S17

4-Methoxyphenyl 4-*O*-dibenzylphosphoryl-3,6-di-*O*-benzyl-2-deoxy-2-(trichloroacetamido)-β-D-glucopyranosyl-(1 -> 2)-3,4,6-tri-*O*-benzyl-α-D-mannopyranoside (S17):

S17 was prepared *according to General procedure II* from **8a** (158 mg, 0.18 mmol, 1.4 equiv.) and **S16**[3] (72 mg, 0.13 mmol, 1.0 equiv.) as a colorless glassy film (84 mg, 0.17 mmol, 50%).

S17: ^{1}H NMR (600 MHz, $CDCl_3$) δ = 7.39 – 7.21 (m, 25H), 7.20 – 7.13 (m, 8H), 7.09 (t, *J* = 7.5 Hz, 2H), 7.01 – 6.97 (m, 2H), 6.80 – 6.77 (m, 2H), 5.44 (d, *J* = 2.0 Hz, 1H), 5.30 (d, *J* = 8.6 Hz, 1H), 4.96 – 4.90 (m, 3H), 4.88 – 4.82 (m, 4H), 4.73 – 4.66 (m, 2H), 4.63 – 4.58 (m, 2H), 4.58 – 4.50 (m, 2H), 4.47 – 4.41 (m, 3H), 4.38 (d, *J* = 12.0 Hz, 1H), 4.14 (dd, *J* = 9.5, 3.1 Hz, 1H), 4.02 (t, *J* = 9.6 Hz, 1H), 3.92 – 3.87 (m, 1H), 3.84 – 3.79 (m, 1H), 3.78 – 3.71 (m, 6H), 3.66 (dd, *J* = 11.1, 1.9 Hz, 1H), 3.44 (ddd, *J* = 10.3, 8.6, 6.5 Hz, 1H) ppm.

^{13}C NMR (151 MHz, $CDCl_3$) δ = 162.5, 155.1, 150.3, 138.50, 138.47, 138.21, 138.17, 137.3, 135.82, 135.76, 135.7, 129.0, 128.8, 128.7, 128.63, 128.59, 128.54, 128.50, 128.40, 128.37, 128.34, 128.32, 128.00, 127.98, 127.8, 127.74, 127.65, 127.61, 127.56, 127.5, 117.8, 114.7, 97.8, 96.6, 92.3, 78.8, 77.1 (d, *J* = 3.4 Hz), 76.6 (d, *J* = 6.7 Hz), 75.2, 75.0, 74.7 (d, *J* = 4.5 Hz), 74.4, 73.7, 73.5, 73.4, 72.6, 72.1, 69.63 (d, *J* = 5.3 Hz), 69.58 (d, *J* = 5.3 Hz), 69.1, 68.9, 58.6, 55.7 ppm; ^{31}P NMR (162 MHz, $CDCl_3$) δ = −1.8 ppm.

Synthesis of 5

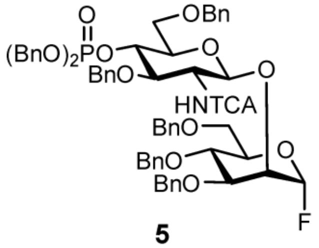

5

Synthesis of 4-*O*-dibenzylphosphoryl-3,6-di-*O*-benzyl-2-deoxy-2-(trichloroacetamido)-β-D-gluco-pyranosyl-(1 -> 2)-3,4,6-tri-*O*-benzyl-α-D-mannopyranosyl (lactol):

PMP removal in the preparation of **5** was referred to the literature.[5]

To a vigorously stirred mixture of **S17** in HFIP/H_2O (1.4 mL/150 µL, 9/1) was added DDQ (36 mg, 0.16 mmol, 1.2 equiv.) at 0 °C. The reaction mixture was kept at this temperature for 3 hrs before it was diluted with CH_2Cl_2 and washed with saturated $NaHCO_3$ solution and brine. The combined organic layers were dried over anhydrous Na_2SO_4, filtered, and concentrated to give a crude mixture, which was purified by flash chromatography (silica gel, hexane/EtOAc) to give the product as colorless oil.

4-*O*-dibenzylphosphoryl-3,6-di-*O*-benzyl-2-deoxy-2-(trichloroacetamido)-β-D-glucopyranosyl-(1 -> 2)-3,4,6-tri-*O*-benzyl-α-D-mannopyranosyl fluoride (5):

Fluoride **5** was prepared *according to General procedure I* from the obtained lactol (122 mg, 102 µmol, 1.0 equiv.) as a colorless glassy film (105 mg, 86%).

5: ^{1}H NMR (600 MHz, $CDCl_3$) δ 7.37 – 7.20 (m, 30H), 7.19 – 7.14 (m, 3H), 7.08 (t, *J* = 7.5 Hz, 2H), 5.60 (dd, *J* = 50.7, 2.2 Hz, 1H), 5.26 (d, *J* = 8.5 Hz, 1H), 4.95 (dd, *J* = 13.2, 7.7 Hz, 3H), 4.90 – 4.81 (m, 3H), 4.78 (d, *J* = 10.5 Hz, 1H), 4.69 – 4.59 (m, 4H), 4.55 (q, *J* = 9.1 Hz, 1H), 4.50 (td, *J* = 8.1, 4.6 Hz, 3H), 4.40 (d, *J* = 12.1 Hz, 1H), 4.35 (t, *J* = 2.7 Hz, 1H), 4.01 (t, *J* = 9.6 Hz, 1H), 3.93 (ddd, *J* = 11.9, 7.7, 3.4 Hz, 2H), 3.86 – 3.80 (m, 1H), 3.79 – 3.71 (m, 3H), 3.69 (dd, *J* = 11.1, 1.8 Hz, 1H), 3.41 (ddd, *J* = 10.2, 8.5, 6.5 Hz, 1H) ppm.

^{13}C NMR (151 MHz, $CDCl_3$) δ = 162.5, 138.28, 138.25, 138.17, 138.16, 137.0, 135.83, 135.78, 135.76, 135.7, 128.9, 128.8, 128.72, 128.70, 128.63, 128.60, 128.56, 128.51, 128.49, 128.45, 128.38, 128.1, 128.0, 127.88, 127.86, 127.81, 127.79, 127.74, 127.71, 127.68, 127.66, 106.9 (d, *J* = 222.3 Hz), 96.5, 92.2, 77.9, 77.1 (d, *J* = 3.6 Hz), 76.5 (d, *J* = 6.6 Hz), 75.2, 74.6 (d, *J* = 4.5 Hz), 74.5, 74.4, 74.3, 73.8, 73.6, 73.3, 70.6

(d, J = 36.1 Hz), 69.72, 69.69, 69.66, 69.6, 68.7, 68.5, 58.5 ppm; ^{19}F NMR (376 MHz, $CDCl_3$) δ = −137.0 ppm.

^{31}P NMR (162 MHz, $CDCl_3$) δ = −1.8 ppm.

Scheme S4. Synthesis of 6

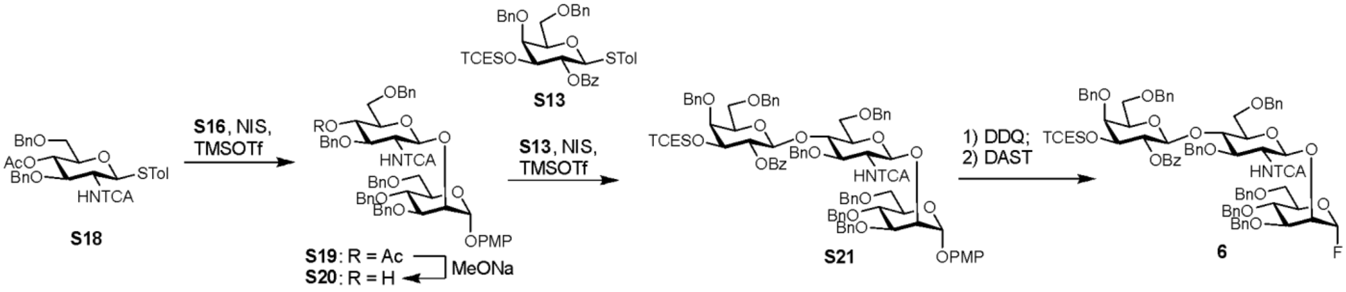

*Synthesis of **S18***

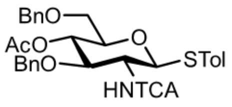

S18

***p*-Tolyl 4-*O*-acetyl-3,6-di-*O*-benzyl-2-deoxy-2-(trichloroacetamido)-1-thio-β-D-glucopyranoside (S18):**

To a solution of **S9** (878 mg) in CH_2Cl_2 (5 mL) at °C were added Et_3N (500 uL), Ac_2O (250 uL) and DMAP (15 mg). The resulting mixture was stirred at this temperature for 1 hr, quenched by the addition of MeOH (), and concentrated under vacuum to give a crude residue, which was purified by flash chromatography (silica gel, hexane/EtOAc) to give **S18** as a white powdery solid.

S18: ^{1}H NMR (600 MHz, $CDCl_3$) δ = 7.42 (d, *J* = 7.9 Hz, 2H), 7.37 – 7.32 (m, 4H), 7.32 – 7.24 (m, 4H), 7.22 – 7.18 (m, 2H), 7.06 (d, *J* = 7.9 Hz, 2H), 6.89 (d, *J* = 7.4 Hz, 1H), 5.26 (d, *J* = 10.2 Hz, 1H), 5.00 (t, *J* = 9.5 Hz, 1H), 4.62 (d, *J* = 11.0 Hz, 1H), 4.58 – 4.50 (m, 3H), 4.30 (t, *J* = 9.5 Hz, 1H), 3.69 (dt, *J* = 9.6, 4.4 Hz, 1H), 3.60 (d, *J* = 4.5 Hz, 2H), 3.37 (td, *J* = 10.1, 7.4 Hz, 1H), 2.32 (s, 3H), 1.87 (s, 3H) ppm.

^{13}C NMR (151 MHz, $CDCl_3$) δ = 169.8, 161.7, 139.0, 138.1, 137.5, 134.0, 130.1, 128.7, 128.5, 128.2, 128.02, 127.98, 127.8, 127.5, 83.9, 78.7, 77.9, 74.9, 73.7, 71.7, 69.7, 57.3, 21.3, 21.0 ppm.

*Synthesis of **S19** and **S20***

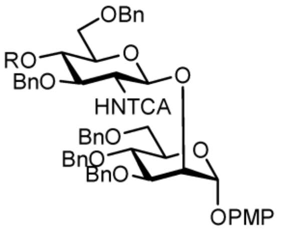

S19: R = Ac
S20: R = H

4-Methoxyphenyl 4-*O*-acetyl-3,6-di-*O*-benzyl-2-deoxy-2-(trichloroacetamido)-β-D-glucopyranosyl-(1 -> 2)-3,4,6-tri-*O*-benzyl-α-D-mannopyranoside (S19):

Based on *General procedure I*, a reaction of **S18** (331 mg, 0.51 mmol, 1.0 equiv.) and **S16** (281 mg, 0.51 mmol, 1.0 equiv.), NIS (172 mg, 0.77 mmol, 1.5 equiv.), TfOH (9 µL, 0.10 mmol, 0.2 equiv.), and CH_2Cl_2 (2 mL) afforded **S19** as a colorless glassy film.

S19: ^{1}H NMR (600 MHz, $CDCl_3$) δ = 7.40 (d, *J* = 6.2 Hz, 1H), 7.37 – 7.19 (m, 22H), 7.15 (t, *J* = 7.6 Hz, 2H), 7.01 – 6.97 (m, 2H), 6.81 – 6.76 (m, 2H), 5.44 (d, *J* = 2.2 Hz, 1H), 5.32 (d, *J* = 8.8 Hz, 1H), 5.07 (dd, *J* = 10.1, 9.0 Hz, 1H), 4.87 (d, *J* = 10.9 Hz, 1H), 4.84 (d, *J* = 10.4 Hz, 1H), 4.73 – 4.67 (m, 2H), 4.65 (d, *J* = 11.4 Hz, 1H), 4.60 (d, *J* = 12.0 Hz, 1H), 4.55 (d, *J* = 11.3 Hz, 1H), 4.52 (d, *J* = 11.0 Hz, 1H), 4.48 – 4.41 (m, 4H), 4.16 – 4.13 (m, 1H), 4.03 (t, *J* = 9.6 Hz, 1H), 3.89 (ddd, *J* = 9.8, 4.4, 1.9 Hz, 1H), 3.79 – 3.75 (m, 4H), 3.74 – 3.71 (m, 1H), 3.66 (dd, *J* = 11.0, 1.9 Hz, 1H), 3.58 – 3.51 (m, 2H), 3.46 – 3.40 (m, 1H), 1.87 (s, 3H) ppm.

^{13}C NMR (151 MHz, $CDCl_3$) δ = 169.9, 162.4, 155.1, 150.2, 138.5, 138.4, 138.1, 137.8, 137.2, 129.0, 128.8, 128.54, 128.51, 128.45, 128.41, 128.38, 127.9, 127.79, 127.76, 127.75, 127.7, 117.8, 114.7, 97.9, 96.6, 92.4, 78.8, 76.2, 75.2, 75.0, 74.7, 73.84, 73.76, 73.7, 73.5, 72.6, 72.0, 71.9, 69.6, 69.1, 58.9, 55.7, 20.9 ppm.

4-Methoxyphenyl 3,6-di-*O*-benzyl-2-deoxy-2-(trichloroacetamido)-β-D-glucopyranosyl-(1 -> 2)-3,4,6-tri-*O*-benzyl-α-D-mannopyranoside (S20):

To the solution of the so obtained **S19** in MeOH/CH_2Cl_2 (2 mL/1 mL) at 22 °C was added MeONa (0.01 mmol, 0.2 equiv., 5.4 M in MeOH). The resulting mixture was neutralized with Amberlite upon completion, filtered, and concentrated under vacuum to give **S20** as a colorless glassy film (360 mg, 0.21 mmol, 68% over 2 steps).

Synthesis of S21

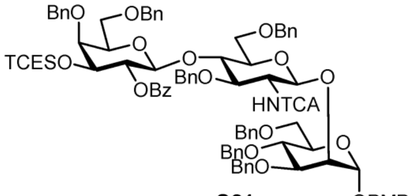

S21

4-Methoxyphenyl 2-*O*-benzoyl-4,6-di-*O*-benzyl-3-*O*-(2,2,2-trichloroethoxysulfonyl)-β-D-galacto-pyranosyl-(1 -> 4)-3,6-di-*O*-benzyl-2-deoxy-2-(trichloroacetamido)-β-D-glucopyranosyl-(1 -> 2)-3,4,6-tri-*O*-benzyl-α-D-mannopyranoside (S21):

Trisaccharide **S21** was prepared according to *General procedure II* from **S13** (261 mg, 0.40 mmol, 1.4 equiv.), **S20** (297 mg, 0.28 mmol, 1.0 equiv.), NIS (92 mg, 0.42 mmol, 1.5 equiv.), and TfOH (10 µL, 0.12 mmol, 0.3 equiv.) in CH_2Cl_2 (5 mL) as a colorless glassy film (349 mg, 0.21 mmol, 72%).

S21: ^{1}H NMR (600 MHz, $CDCl_3$) δ = 8.00 – 7.96 (m, 2H), 7.60 (t, *J* = 7.4 Hz, 1H), 7.45 (t, *J* = 7.7 Hz, 2H), 7.38 – 7.17 (m, 30H), 7.17 – 7.11 (m, 3H), 7.08 (t, *J* = 7.4 Hz, 2H), 6.94 (d, *J* = 9.0 Hz, 2H), 6.77 – 6.73 (m, 2H), 5.65 (dd, *J* = 10.2, 7.8 Hz, 1H), 5.37 (d, *J* = 2.1 Hz, 1H), 5.14 (d, *J* = 8.4 Hz, 1H), 5.01 (d, *J* = 10.4 Hz, 1H), 4.90 (d, *J* = 11.4 Hz, 1H), 4.80 (d, *J* = 10.9 Hz, 1H), 4.75 (dd, *J* = 10.2, 3.2 Hz, 1H), 4.72 (d, *J* = 10.5 Hz, 1H), 4.67 (d, *J* = 7.9 Hz, 1H), 4.64 – 4.57 (m, 3H), 4.54 (d, *J* = 11.9 Hz, 1H), 4.51 (d, *J* = 10.5 Hz, 1H), 4.49 – 4.44 (m, 2H), 4.43 – 4.33 (m, 3H), 4.31 – 4.25 (m, 4H), 4.19 (d, *J* = 12.1 Hz, 1H), 4.08 – 4.00 (m, 2H), 3.94 (t, *J* = 9.5 Hz, 1H), 3.84 (dd, *J* = 10.0, 4.2 Hz, 1H), 3.74 (s, 3H), 3.70 (dd, *J* = 11.1, 4.6 Hz, 1H), 3.66 – 3.60 (m, 2H), 3.54 – 3.47 (m, 2H), 3.46 – 3.40 (m, 2H), 3.33 (q, *J* = 8.5 Hz, 1H), 3.28 (d, *J* = 9.7 Hz, 1H) ppm.

^{13}C NMR (151 MHz, $CDCl_3$) δ = 164.8, 162.1, 155.1, 150.3, 138.8, 138.53, 138.51, 138.0, 137.83, 137.80, 137.6, 133.9, 130.0, 129.0, 128.9, 128.74, 128.68, 128.65, 128.55, 128.5, 128.4, 128.3, 128.2, 128.14, 128.12, 128.09, 128.05, 128.03, 127.99, 127.84, 127.76, 127.7, 127.6, 127.4, 117.8, 114.7, 99.9, 97.8, 97.3, 92.5, 92.4, 84.6, 79.8, 78.7, 76.63, 76.57, 75.7, 75.10, 75.07, 75.0, 74.9, 74.6, 73.8, 73.7, 73.4, 73.2, 73.1, 72.7, 72.6, 70.2, 69.2, 67.9, 67.4, 58.4, 55.7 ppm.

Synthesis of 6

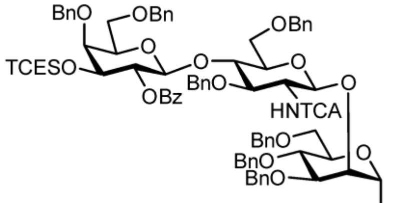

6

For PMP deprotection, the procedure from reference[5] was adapted.

2-*O*-benzoyl-4,6-di-*O*-benzyl-3-*O*-(2,2,2-trichloroethoxysulfonyl)-β-D-galacto-pyranosyl-(1 -> 4)-3,6-di-*O*-benzyl-2-deoxy-2-(trichloroacetamido)-β-D-glucopyranosyl-(1 -> 2)-3,4,6-tri-*O*-benzyl-α-D-mannopyranose (lactol):

To a mixture of **S21** (153 mg, 0.087 mmol) in CH_2Cl_2/HFIP/H_2O (a mixture in 8/5/0.5 v/v/v, 0.7 mL/0.44 mL/44 uL) at 22 °C was added PIFA (53 mg, 0.12 mmol, 1.4 equiv.). The resulting green reaction mixture was stirred at this temperature for 4 hrs and quenched with $NaHCO_3$ (sat.) and $Na_2S_2O_3$ (s) at 0 °C. The reaction mixture was extracted with CH_2Cl_2. The combined organic layers were concentrated under vacuum to give a crude, which was purified by flash chromatography (silica gel, hexane/EtOAc) to give 114 mg (0.069 mmol, 80%) pure lactol.

2-*O*-benzoyl-4,6-di-*O*-benzyl-3-*O*-(2,2,2-trichloroethoxysulfonyl)-β-D-galacto-pyranosyl-(1 -> 4)-3,6-di-*O*-benzyl-2-deoxy-2-(trichloroacetamido)-β-D-glucopyranosyl-(1 -> 2)-3,4,6-tri-*O*-benzyl-α-D-manno-pyranosyl fluoride (6):

Based on *General procedure I*, **6** was prepared from the so-obtained lactol (114 mg, 0.071 mmol, 1.0equiv.) with DAST (16 µL, 0.12 mmol, 1.8 equiv.) as a colorless glassy film (104 mg, 0.065 mmol, 92%).

6: ^{1}H NMR (600 MHz, $CDCl_3$) δ = 7.99 (dd, *J* = 8.2, 1.4 Hz, 2H), 7.64 – 7.60 (m, 1H), 7.48 (t, *J* = 7.8 Hz, 2H), 7.43 – 7.20 (m, 27H), 7.19 – 7.11 (m, 6H), 7.07 (t, *J* = 7.5 Hz, 2H), 5.64 (dd, *J* = 10.2, 7.8 Hz, 1H), 5.52 (dd, *J* = 50.9, 1.5 Hz, 1H), 5.08 (d, *J* = 8.4 Hz, 1H), 5.00 (d, *J* = 10.5 Hz, 1H), 4.90 (d, *J* = 11.4 Hz, 1H), 4.79 – 4.73 (m, 2H), 4.67 – 4.64 (m, 2H), 4.63 (d, *J* = 12.1 Hz, 1H), 4.60 – 4.56 (m, 2H), 4.54 (d, *J* = 10.6 Hz, 1H), 4.50 (d, *J* = 10.6 Hz, 1H), 4.49 – 4.45 (m, 2H), 4.43 (d, *J* = 10.9 Hz, 1H), 4.40 (d, *J* = 11.6 Hz, 1H), 4.32 (dd, *J* = 10.1, 8.5 Hz, 1H), 4.29 (d, *J* = 3.5 Hz, 1H), 4.28 – 4.24 (m, 3H), 4.20 (br t, *J* = 2.7

Hz, 1H), 4.03 (t, J = 9.0 Hz, 1H), 3.91 (t, J = 9.5 Hz, 1H), 3.88 – 3.81 (m, 2H), 3.72 – 3.67 (m, 1H), 3.62 (ddd, J = 13.9, 11.0, 2.5 Hz, 2H), 3.53 – 3.47 (m, 2H), 3.45 – 3.40 (m, 2H), 3.32 – 3.26 (m, 2H) ppm.

^{13}C NMR (151 MHz, $CDCl_3$) δ = 164.8, 162.1, 138.7, 138.21, 138.18, 137.84, 137.76, 137.3, 134.0, 130.0, 129.0, 128.8, 128.73, 128.69, 128.67, 128.6, 128.53, 128.50, 128.25, 128.21, 128.18, 128.12, 128.11, 128.05, 128.0, 127.9, 127.8, 127.5, 106.8 (d, J = 221.3 Hz), 99.9, 97.2, 92.4, 92.3, 84.5, 79.8, 77.8, 76.5, 76.4, 75.7, 75.2, 75.1, 74.8, 74.6, 74.3, 74.1, 73.7, 73.6, 73.2, 73.1, 71.2 (d, J = 35.5 Hz), 70.2, 68.6, 67.7, 67.4, 58.3 ppm.

Scheme S5. Synthesis of 15 from 8 and 9

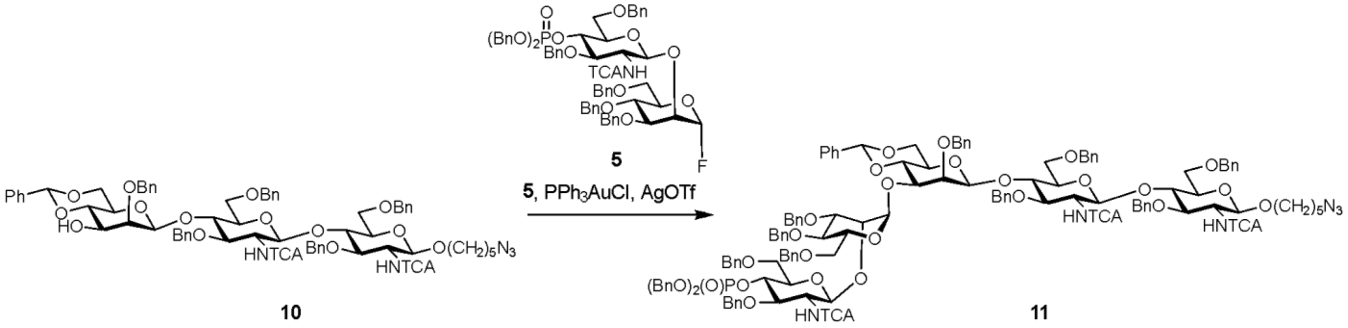

5-Azidopentyl 4-*O*-dibenzylphosphoryl-3,6-di-*O*-benzyl-2-deoxy-2-(trichloroacetamido)-β-D-glucopyranosyl-(1 -> 2)-3,4,6-tri-*O*-benzyl-α-D-mannopyranosyl-(1 -> 4)-2-*O*-benzyl-4,6-benzylidene-β-D-mannopyranosyl-(1 -> 4)-3,6-di-*O*-benzyl-2-deoxy-2-(trichloroacetamido)-β-D-glucopyranosyl-(1 -> 4)-3,6-di-*O*-benzyl-2-deoxy-2-(trichloroacetamido)-β-D-glucopyranoside (11):

Pentasaccharide **11** was prepared according to *General procedure III*: the promoter of Cp_2HfCl_2/AgOTf was prepared in toluene/CH_2Cl_2 1 mL/1 mL; **5** (45 mg, 38 μmol, 1.2 equiv.) and **10** (45 mg, 31 μmol, 1.0 equiv) were dissolved in 1.5 mL toluene for addition. ⟨ anomer **11** was obtained as a colorless glassy film (37 mg, 14 ⌠mol, 46%).

Scheme S6. Synthesis of 11 from 14 and 8a

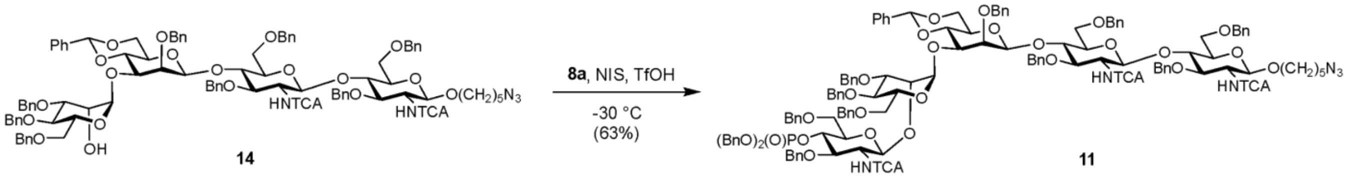

The preparation of **14** was described below in Scheme S7.

Pentasaccharide **11** was prepared *according to General procedure II* from **8a** (163 mg, 187 μmol, 1.6 equiv.), **14** (219 mg, 117 μmol, 1.0 equiv.), and NIS (47 mg, 0.21 mmol, 1.8 equiv.) in CH_2Cl_2 (2 mL) as a colorless glassy film (200 mg, 73.4 μmol, 63%).

11: ^{1}H NMR (500 MHz, $CDCl_3$) δ = 7.50 – 7.45 (m, 2H), 7.43 – 7.11 (m, 61H), 7.11 – 7.05 (m, 2H), 6.94 (d, *J* = 7.8 Hz, 1H), 6.65 – 6.59 (m, 2H), 5.26 (s, 1H), 5.13 (d, *J* = 1.9 Hz, 1H), 5.05 (d, *J* = 10.5 Hz, 1H), 4.95 (d, *J* = 11.4 Hz, 1H), 4.93 – 4.87 (m, 4H), 4.87 – 4.81 (m, 2H), 4.81 – 4.74 (m, 5H), 4.72 (d, *J* = 12.0 Hz, 1H), 4.68 (d, *J* = 11.1 Hz, 1H), 4.63 – 4.57 (m, 2H), 4.53 (d, *J* = 12.1 Hz, 1H), 4.50 (d, *J* = 8.3 Hz, 1H), 4.47 – 4.45 (m, 2H), 4.45 – 4.33 (m, 7H), 4.29 – 4.23 (m, 2H), 4.17 – 4.10 (m, 2H), 4.01 – 3.82 (m,

6H), 3.81 – 3.61 (m, 10H), 3.61 – 3.49 (m, 6H), 3.45 (dt, *J* = 9.7, 6.6 Hz, 1H), 3.38 – 3.31 (m, 2H), 3.23 (t, *J* = 6.9 Hz, 2H), 3.20 – 3.15 (m, 1H), 2.77 (td, *J* = 9.6, 4.9 Hz, 1H), 2.72 – 2.67 (m, 1H), 1.66 – 1.52 (m, 4H), 1.47 – 1.35 (m, 2H) ppm.

^{13}C NMR (151 MHz, $CDCl_3$) δ = 161.9, 161.8, 161.7, 138.7, 138.45, 138.43, 138.36, 138.3, 138.2, 137.9, 137.83, 137.81, 135.9, 135.85, 135.81, 130.2, 128.9, 128.8, 128.72, 128.67, 128.65, 128.58, 128.56, 128.48, 128.46, 128.40, 128.38, 128.15, 128.13, 128.07, 128.03, 127.99, 127.97, 127.95, 127.94, 127.86, 127.84, 127.79, 127.77, 127.72, 127.68, 127.6, 127.5, 126.8, 102.2, 101.0, 99.6, 98.6, 98.5, 97.2, 92.60, 92.58, 92.57, 78.9, 78.5, 78.1, 78.0, 77.9, 75.9, 75.8 (d, *J* = 5.0 Hz), 75.5, 75.4, 75.1, 75.0, 74.9, 74.8, 74.4, 73.8 (d, *J* = 4.4 Hz), 73.6, 73.5, 73.41, 73.39, 73.2, 72.4, 71.8, 71.2, 70.3, 69.7, 69.62, 69.59, 69.55, 69.0, 68.6, 68.5, 68.4, 66.7, 58.5, 57.5, 57.4, 51.4, 29.2, 28.7, 23.4 ppm.

Synthesis of 12

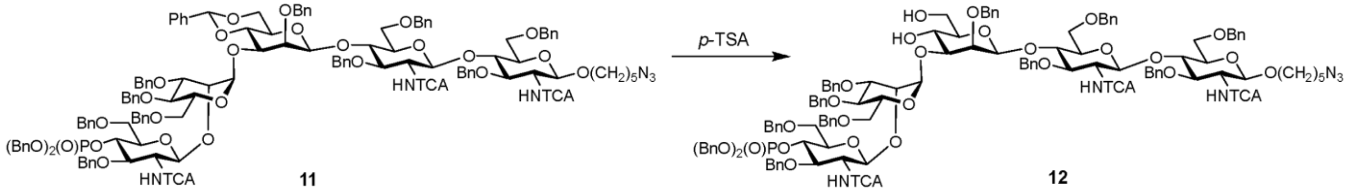

5-Azidopentyl 4-*O*-dibenzylphosphoryl-3,6-di-*O*-benzyl-2-deoxy-2-(trichloroacetamido)-β-D-glucopyranosyl-(1 -> 2)-3,4,6-tri-*O*-benzyl-α-D-mannopyranosyl-(1 -> 4)-2-*O*-benzyl-β-D-mannopyranosyl-(1 -> 4)-3,6-di-*O*-benzyl-2-deoxy-2-(trichloroacetamido)-β-D-glucopyranosyl-(1 -> 4)-3,6-di-*O*-benzyl-2-deoxy-2-(trichloroacetamido)-β-D-glucopyranoside (12):

To a solution of **11** (117 mg, 0.045 mmol, 1.0 equiv.) in MeCN (1 mL) at 22 °C was added *p*-toluenesulfonic acid (17 mg, 0.073 mmol, 1.6 equiv.). The reaction mixture was stirred at this temperature for 6 hrs before it was neutralized with Et_3N. The reaction mixture was concentrated under vacuum to give a crude mixture, which was purified by flash chromatography (silica gel, toluene/EtOAc) to give **12** as a colorless glassy film (97 mg, 0.031 mmol, 69%).

12: ^{1}H NMR (600 MHz, $CDCl_3$) δ = 7.40 (d, *J* = 7.7 Hz, 2H), 7.38 – 7.10 (m, 58H), 6.91 (d, *J* = 7.9 Hz, 1H), 6.52 (br d, *J* = 6.4 Hz, 1H), 5.36 (d, *J* = 2.7 Hz, 1H), 5.00 (d, *J* = 10.9 Hz, 1H), 4.97 – 4.66 (m, 15H), 4.65 – 4.56 (m, 3H), 4.55 – 4.27 (m, 12H), 4.25 (br t, *J* = 2.9 Hz, 1H), 4.12 (t, *J* = 8.2 Hz, 1H), 4.06 (t, *J* = 9.3 Hz, 1H), 3.96 (t, *J* = 8.5 Hz, 1H), 3.93 – 3.40 (m, 23H), 3.27 (dd, *J* = 11.7, 5.8 Hz, 1H), 3.23 (t, *J* = 6.9 Hz, 2H), 3.17 (dt, *J* = 9.6, 3.0 Hz, 1H), 2.98 – 2.92 (m, 1H), 1.91 (br s, 2H), 1.63 – 1.53 (m, 4H), 1.48 – 1.34 (m, 2H) ppm.

^{13}C NMR (151 MHz, $CDCl_3$) δ = 162.1, 161.84, 161.82, 139.0, 138.4, 138.33, 138.31, 138.1, 137.94, 137.92, 137.88, 137.71, 137.69, 135.65, 135.63, 135.60, 135.58, 128.72, 128.70, 128.66, 128.63, 128.58, 128.55, 128.51, 128.50, 128.46, 128.42, 128.37, 128.2, 128.09, 128.08, 128.01, 128.00, 127.91, 127.87, 127.81, 127.79, 127.72, 127.68, 127.6, 127.4, 127.1, 100.7, 99.7, 99.4, 98.7, 97.2, 92.64, 92.57, 80.8, 79.1, 78.3, 78.1, 78.0, 77.5, 76.02, 75.95 (d, *J* = 5.1 Hz), 75.94, 75.6, 74.9, 74.83, 74.76, 74.6, 74.5, 74.22 (d, *J* = 4.4 Hz), 73.7, 73.52, 73.50, 73.47, 73.4, 73.0, 72.9, 72.1, 69.9, 69.70, 69.66, 69.6, 68.9, 68.4, 68.3, 67.3, 62.6, 58.3, 57.5, 57.3, 51.4, 29.1, 28.7, 23.3 ppm.

Synthesis of 13

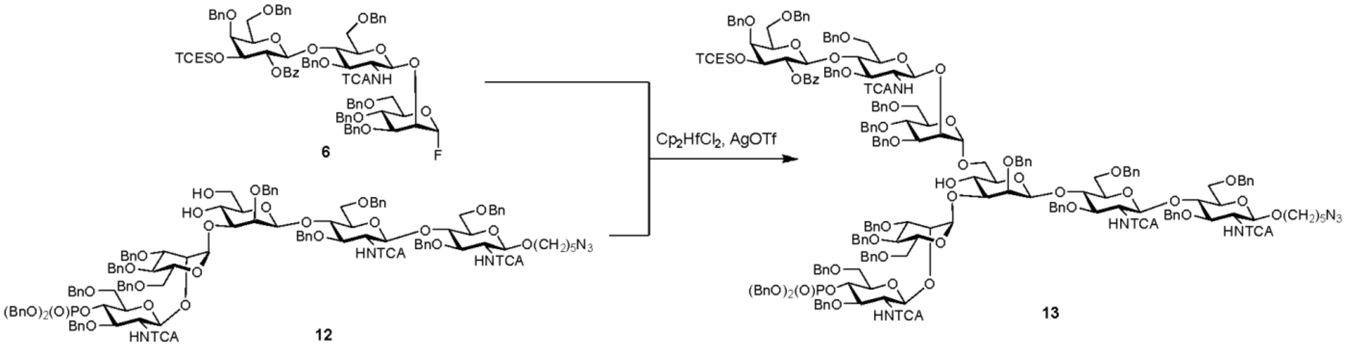

5-Azidopentyl 2-*O*-benzoyl-4,6-di-*O*-benzyl-3-*O*-(2,2,2-trichloroethoxysulfonyl)-β-D-galactopyranosyl-(1 -> 4)-3,6-di-*O*-benzyl-2-deoxy-2-(trichloroacetamido)-β-D-glucopyranosyl-(1 -> 2)-3,4,6-tri-*O*-benzyl-α-D-mannopyranosyl-(1 -> 6)-[4-*O*-dibenzylphosphoryl-3,6-di-*O*-benzyl-2-deoxy-2-(trichloroacet-amido)-β-D-glucopyranosyl-(1 -> 2)-3,4,6-tri-*O*-benzyl-α-D-mannopyranosyl-(1 -> 4)]-2-*O*-benzyl-β-D-mannopyranosyl-(1 -> 4)-3,6-di-*O*-benzyl-2-deoxy-2-(trichloroacetamido)-β-D-glucopyranosyl-(1 -> 4)-3,6-di-*O*-benzyl-2-deoxy-2-(trichloroacetamido)-β-D-glucopyranoside (13):

According to General procedure III, from **12** (150 mg, 59.2 µmol, 1.0 equiv.) and **6** (120 mg, 75.1 µmol, 1.27 equiv.), octasaccharide **13** was obtained in a mixture of α/β anomers (3/1, 82 mg, 33%) along with inseparable regioisomeric byproducts and unreacted **12** (43 mg). The mixture of a/b products was purified by PTLC (silica gel, toluene/EtOAc) to afforded pure **13** for NMR characterization.

13: ^{1}H NMR (600 MHz, $CDCl_3$) δ = 7.94 (d, *J* = 7.8 Hz, 2H), 7.53 (t, *J* = 7.5 Hz, 1H), 7.39 (t, *J* = 7.8 Hz, 2H), 7.35 – 7.04 (m, 95H), 6.87 (d, *J* = 8.0 Hz, 1H), 6.48 (br d, *J* = 6.1 Hz, 1H), 5.60 (dd, *J* = 10.1, 7.9 Hz, 1H), 5.29 (d, *J* = 2.3 Hz, 1H), 5.04 (br d, *J* = 8.2 Hz, 1H), 4.97 (d, *J* = 11.8 Hz, 1H), 4.93 – 4.16 (m, 49H), 4.09 (d, *J* = 2.9 Hz, 1H), 4.05 – 4.00 (m, 2H), 3.95 (d, *J* = 12.0 Hz, 1H), 3.89 – 3.16 (m, 41H), 3.15 – 3.09 (m, 2H), 1.60 – 1.53 (m, 4H), 1.46 – 1.34 (m, 2H) ppm.

Scheme S7. Synthesis of 14

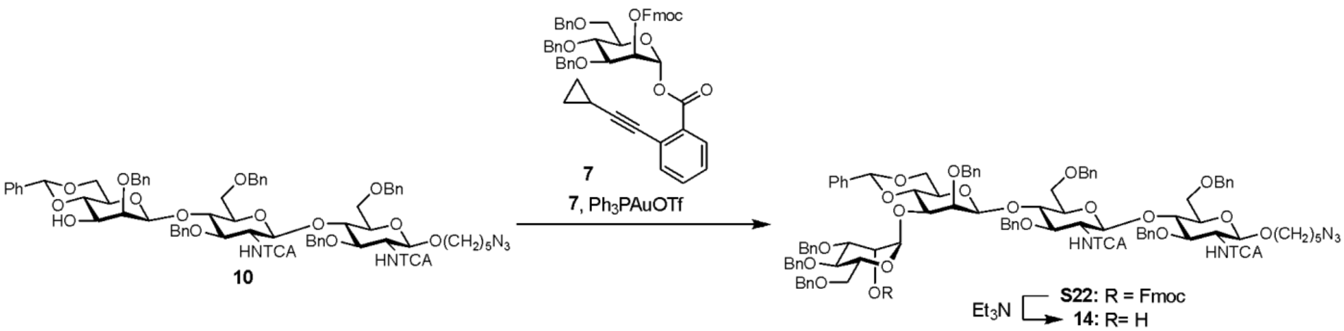

5-Azidopentyl 2-*O*-fluorenylmethoxycarbonyl-3,4,6-tri-*O*-benzyl-α-D-mannopyranosyl-(1 -> 4)-2-*O*-benzyl-4,6-benzylidene-β-D-mannopyranosyl-(1 -> 4)-3,6-di-*O*-benzyl-2-deoxy-2-(trichloroacetamido)-β-D-glucopyranosyl-(1 -> 4)-3,6-di-*O*-benzyl-2-deoxy-2-(trichloroacetamido)-β-D-glucopyranoside (S22):

o-Alkynylbenzoate **S22** was prepared *according to General procedure IV* from **10** (255 mg, 177 µmol, 1.0 equiv.) and **7**[3] (193 mg, 230 µmol, 1.3 equiv.) as a colorless glassy film (320 mg, 153 µmol, 86%).

5-Azidopentyl 3,4,6-tri-*O*-benzyl-α-D-mannopyranosyl-(1 -> 4)-2-*O*-benzyl-4,6-benzylidene-β-D-mannopyranosyl-(1 -> 4)-3,6-di-*O*-benzyl-2-deoxy-2-(trichloroacetamido)-β-D-glucopyranosyl-(1 -> 4)-3,6-di-*O*-benzyl-2-deoxy-2-(trichloroacetamido)-β-D-glucopyranoside (14):

Tetrasaccharide **14** was prepared *according to General procedure VI* from **S27** (220 mg, 0.10 mmol) as a colorless glassy film (170 mg, 0.91 mmol, 92%).

14: ^{1}H NMR (600 MHz, $CDCl_3$) δ = 7.45 – 7.14 (m, 45H), 6.92 (d, *J* = 7.9 Hz, 1H), 6.61 (d, *J* = 8.0 Hz, 1H), 5.45 (s, 1H), 5.27 (d, *J* = 1.8 Hz, 1H), 5.05 (d, *J* = 10.6 Hz, 1H), 4.93 (d, *J* = 11.4 Hz, 1H), 4.84 (d, *J* = 11.2 Hz, 2H), 4.80 – 4.76 (m, 3H), 4.71 (d, *J* = 12.0 Hz, 1H), 4.63 – 4.58 (m, 3H), 4.56 (d, *J* = 11.4 Hz, 1H), 4.53 (d, *J* = 12.1 Hz, 1H), 4.51 – 4.47 (m, 3H), 4.46 – 4.39 (m, 2H), 4.23 (d, *J* = 12.0 Hz, 1H), 4.16 – 4.10 (m, 2H), 4.03 (dd, *J* = 10.4, 4.8 Hz, 1H), 4.00 – 3.93 (m, 3H), 3.89 – 3.77 (m, 5H), 3.76 – 3.65 (m, 6H), 3.62 (q, *J* = 8.1 Hz, 1H), 3.58 – 3.52 (m, 2H), 3.50 (dt, *J* = 7.5, 3.5 Hz, 1H), 3.48 – 3.41 (m, 2H), 3.38 (dd, *J* = 11.1, 3.5 Hz, 1H), 3.23 (t, *J* = 6.9 Hz, 2H), 3.18 (dt, *J* = 9.3, 3.0 Hz, 1H), 2.97 (td, *J* = 9.7, 4.9 Hz, 1H), 2.39 (d, *J* = 2.4 Hz, 1H), 1.62 – 1.54 (m, 4H), 1.48 – 1.34 (m, 2H) ppm.

^{13}C NMR (151 MHz, $CDCl_3$) δ = 161.9, 161.8, 138.51, 138.48, 138.4, 138.25, 138.16, 137.9, 137.7, 137.4, 129.1, 128.8, 128.7, 128.6, 128.47, 128.46, 128.44, 128.39, 128.2, 128.11, 128.07, 128.06, 127.99, 127.97, 127.9, 127.85, 127.83, 127.80, 127.79, 127.7, 126.1, 101.6, 101.3, 100.4, 99.7, 98.6, 92.6, 80.0, 78.8, 78.7,

78.05, 77.99, 77.6, 75.8, 75.7, 75.4, 75.2, 75.1, 74.9, 74.8, 74.4, 74.3, 73.6, 73.5, 73.4, 72.1, 72.0, 69.6, 69.3, 68.5, 68.4, 68.1, 67.1, 58.4, 57.4, 51.4, 29.2, 28.7, 23.4 ppm.

Synthesis of S23 and S24

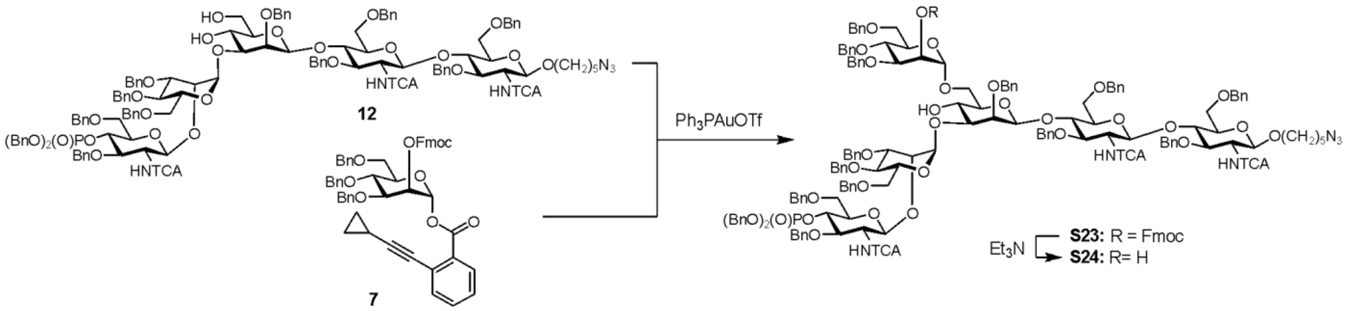

5-Azidopentyl 4-*O*-dibenzylphosphoryl-3,6-di-*O*-benzyl-2-deoxy-2-(trichloroacetamido)-β-D-gluco-pyranosyl-(1 -> 2)-3,4,6-tri-*O*-benzyl-α-D-mannopyranosyl-(1 -> 4)-[2-*O*-fluorenylmethoxycarbonyl-3,4,6-tri-*O*-benzyl-α-D-mannopyranosyl-(1 -> 6)]-2-*O*-benzyl-β-D-mannopyranosyl-(1 -> 4)-3,6-di-*O*-benzyl-2-deoxy-2-(trichloroacetamido)-β-D-glucopyranosyl-(1 -> 4)-3,6-di-*O*-benzyl-2-deoxy-2-(trichloroacetamido)-β-D-glucopyranosid (S23)

S23 was prepared *according to General procedure IV* in 60% yield.

S23: ^{1}H NMR (600 MHz, $CDCl_3$) δ = 7.75 (d, *J* = 7.6 Hz, 2H), 7.61 – 7.53 (m, 2H), 7.40 – 7.02 (m, 79H), 6.85 (d, *J* = 7.9 Hz, 1H), 6.56 (d, *J* = 7.8 Hz, 1H), 5.31 (d, *J* = 2.3 Hz, 1H), 5.19 (dd, *J* = 3.1, 1.8 Hz, 1H), 5.03 (d, *J* = 11.2 Hz, 1H), 4.96 – 4.89 (m, 2H), 4.89 – 4.67 (m, 12H), 4.64 (d, *J* = 12.0 Hz, 1H), 4.61 – 4.55 (m, 2H), 4.55 – 4.32 (m, 14H), 4.33 – 4.27 (m, 2H), 4.23 (t, *J* = 2.7 Hz, 1H), 4.20 – 4.07 (m, 5H), 4.04 (t, *J* = 8.0 Hz, 1H), 3.91 (t, *J* = 9.3 Hz, 2H), 3.88 – 3.74 (m, 9H), 3.73 – 3.33 (m, 21H), 3.22 (t, *J* = 6.9 Hz, 2H), 3.19 – 3.12 (m, 2H), 1.60 – 1.52 (m, 4H), 1.46 – 1.33 (m, 2H) ppm.

^{13}C NMR (151 MHz, $CDCl_3$) δ = 162.1, 161.7, 154.8, 143.6, 143.5, 141.33, 141.31, 139.0, 138.7, 138.54, 138.48, 138.42, 138.38, 138.2, 138.1, 138.02, 137.99, 137.95, 137.9, 137.7, 135.73, 135.71, 135.68, 135.66, 128.72, 128.70, 128.63, 128.60, 128.55, 128.53, 128.51, 128.49, 128.46, 128.40, 128.37, 128.24, 128.22, 128.20, 128.13, 128.09, 128.02, 128.00, 127.97, 127.93, 127.91, 127.85, 127.79, 127.77, 127.74, 127.72, 127.68, 127.66, 127.6, 127.5, 127.31, 127.29, 127.1, 125.6, 125.4, 120.09, 120.05, 101.2, 99.6, 99.4, 98.7, 97.4, 97.2, 92.62, 92.61, 92.5, 80.7, 78.8, 78.6, 78.3, 78.2, 77.93, 77.87, 76.0 (d, *J* = 6.5 Hz), 75.5, 75.4, 75.3, 75.1, 74.9, 74.8, 74.44, 74.40, 74.3, 74.2 (d, *J* = 4.2 Hz), 73.8, 73.5, 73.42, 73.40, 73.3, 72.8, 72.7, 72.3, 71.62, 71.59, 70.3, 69.8, 69.7 (d, *J* = 5.3 Hz), 69.63 (d, *J* = 5.3 Hz), 69.56, 69.0, 68.8, 68.7, 68.4, 67.29, 67.25, 58.4, 57.5, 57.3, 51.4, 46.7, 29.2, 28.7, 23.4 ppm.

5-Azidopentyl 4-*O*-dibenzylphosphoryl-3,6-di-*O*-benzyl-2-deoxy-2-(trichloroacetamido)-β-D-gluco-pyranosyl-(1 -> 2)-3,4,6-tri-*O*-benzyl-α-D-mannopyranosyl-(1 -> 4)-[3,4,6-tri-*O*-benzyl-α-D-manno-pyranosyl-(1 -> 6)]-2-*O*-benzyl-β-D-mannopyranosyl-(1 -> 4)-3,6-di-*O*-benzyl-2-deoxy-2-(trichloro-acetamido)-β-D-glucopyranosyl-(1 -> 4)-3,6-di-*O*-benzyl-2-deoxy-2-(trichloroacetamido)-β-D-gluco-pyranoside (S24)

Hexasaccharide **S24** was prepared *according to General procedure VI* from **S23** in 89% yield.

S24: ^{1}H NMR (500 MHz, $CDCl_3$) δ = 7.40 – 7.07 (m, 75H), 6.91 (d, *J* = 7.9 Hz, 1H), 6.49 (br d, *J* = 7.9 Hz, 1H), 5.31 (d, *J* = 2.5 Hz, 1H), 5.02 (d, *J* = 11.4 Hz, 1H), 4.97 (br d, *J* = 8.4 Hz, 1H), 4.92 (d, *J* = 12.1 Hz, 1H), 4.90 – 4.82 (m, 5H), 4.82 – 4.79 (m, 1H), 4.79 – 4.65 (m, 8H), 4.59 (d, *J* = 10.7 Hz, 1H), 4.56 – 4.31 (m, 16H), 4.29 (d, *J* = 11.3 Hz, 1H), 4.26 (t, *J* = 2.8 Hz, 1H), 4.17 (t, *J* = 9.3 Hz, 1H), 4.08 (t, *J* = 7.8 Hz, 1H), 3.97 (t, *J* = 8.8 Hz, 1H), 3.92 – 3.34 (m, 32H), 3.22 (t, *J* = 6.9 Hz, 2H), 3.19 (dt, *J* = 6.4, 3.1 Hz, 1H), 3.11 (ddd, *J* = 9.0, 5.2, 3.3 Hz, 1H), 2.40 (br s, 1H), 1.63 – 1.53 (m, 4H), 1.46 – 1.35 (m, 2H) ppm.

^{13}C NMR (151 MHz, $CDCl_3$) δ = 162.1, 161.78, 161.77, 139.0, 138.7, 138.5, 138.4, 138.3, 138.12, 138.08, 138.04, 138.03, 137.9, 137.7, 135.73, 135.70, 135.68, 135.65, 128.71, 128.69, 128.62, 128.59, 128.54, 128.50, 128.48, 128.46, 128.44, 128.42, 128.40, 128.37, 128.34, 128.27, 128.25, 128.07, 128.05, 128.00, 127.98, 127.96, 127.9, 127.81, 127.77, 127.73, 127.69, 127.64, 127.62, 127.59, 127.0, 100.9, 99.6, 99.4, 98.9, 97.2, 92.60, 92.56, 80.8, 80.3, 78.9, 78.2, 78.1, 78.0, 77.6, 76.1 (d, *J* = 6.8 Hz), 75.5, 75.4, 75.2, 75.1, 75.0, 74.9, 74.54, 74.52, 74.32, 74.2 (d, *J* = 4.4 Hz), 73.8, 73.5, 73.42, 73.39, 73.37, 72.9, 72.8, 72.3, 71.7, 71.3, 69.8, 69.7 (d, *J* = 5.3 Hz), 69.62 (d, *J* = 5.3 Hz), 69.58, 69.0, 68.9, 68.6, 68.5, 68.2, 67.6, 67.5, 58.2, 57.6, 57.3, 51.4, 29.1, 28.7, 23.3 ppm.

Scheme S8. Synthesis of 9a

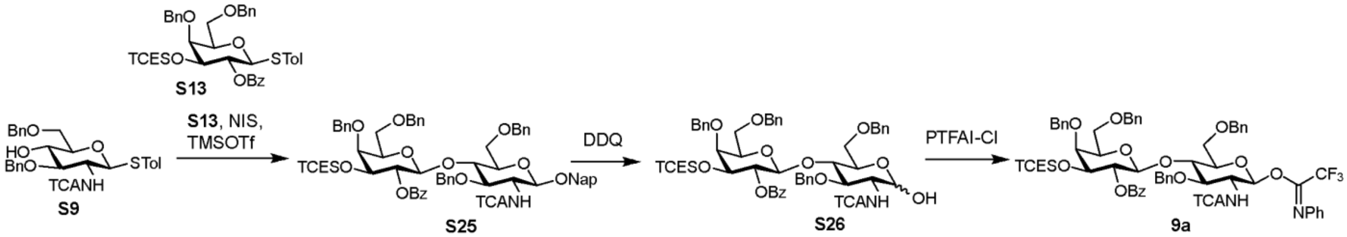

Synthesis of S25

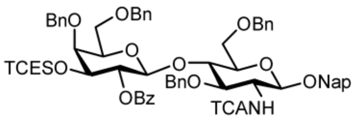

S25

2-Naphthyl 2-*O*-benzoyl-4,6-di-*O*-benzyl-3-*O*-(2,2,2-trichloroethoxysulfonyl)-β-D-galactopyranosyl-(1 -> 4)-3,6-di-*O*-benzyl-2-deoxy-2-(trichloroacetamido)-β-D-glucopyranoside (S25):

A mixture of **S9** (61 mg, 0.1 mmol, 1.0 equiv.), 2-naphthalenemethanol (16 mg, 0.1 mmol, 1.0 equiv.), flame-dried 4Å molecular sieves (100 mg/mL) in CH_2Cl_2 (2 mL) at −45 °C was stirred for 5 min before NIS (25 mg, 0.11 mmol, 1.1 equiv.) and TMSOTf (4 µL, 0.022 mmol, 0.22 equiv.). were added sequentially. The reaction mixture was slowly warmed to 0 °C over the course of 30 min and then again lowered to −45 °C. The reaction mixture was stirred at this temperature for 5 min before **S13** (78 mg, 0.1 mmol, 1.0 equiv.) and NIS (25 mg, 0.11 mmol, 1.1 equiv.) were added. The reaction mixture was slowly warmed to 0 °C over the course of 30 min and then quenched with Et_3N/$NaHCO_3$ (sat.)/$Na_2S_2O_3$ (s) at −60 °C. The mixture was extracted with CH_2Cl_2 and the combined organic layers were dried over anhydrous Na_2SO_4, filtered, and concentrated under vacuum to give a crude mixture, which was purified by flash chromatography (silica gel, hexane/EtOAc) to give disaccharide **S25** as a colorless glassy film (99 mg, 0.076 mmol, 76% over 2 steps).

S25: ^{1}H NMR (500 MHz, $CDCl_3$) δ = 7.99 (d, *J* = 8.2 Hz, 2H), 7.84 – 7.73 (m, 3H), 7.70 (s, 1H), 7.65 – 7.57 (m, 1H), 7.53 – 7.23 (m, 22H), 7.23 – 7.17 (m, 1H), 7.16 – 7.09 (m, 2H), 6.90 (d, *J* = 8.0 Hz, 1H), 5.64 (dd, *J* = 10.3, 7.7 Hz, 1H), 4.97 – 4.92 (m, 2H), 4.92 – 4.88 (m, 1H), 4.84 (d, *J* = 7.8 Hz, 1H), 4.79 – 4.72 (m, 2H), 4.67 – 4.54 (m, 4H), 4.49 (d, *J* = 10.9 Hz, 1H), 4.42 – 4.33 (m, 2H), 4.33 – 4.26 (m, 3H),

4.08 (t, *J* = 8.3 Hz, 1H), 3.93 (t, *J* = 8.6 Hz, 1H), 3.74 – 3.65 (m, 2H), 3.57 (dd, *J* = 10.8, 2.7 Hz, 1H), 3.53 – 3.46 (m, 2H), 3.44 – 3.37 (m, 1H), 3.31 (dt, *J* = 8.6, 2.9 Hz, 1H) ppm.

^{13}C NMR (126 MHz, $CDCl_3$) δ = 164.9, 161.8, 138.2, 138.0, 137.8, 137.7, 134.5, 133.9, 133.3, 133.2, 130.0, 129.0, 128.9, 128.8, 128.6, 128.54, 128.52, 128.44, 128.35, 128.30, 128.26, 128.1, 128.04, 128.01, 127.99, 127.8, 127.6, 126.9, 126.3, 126.1, 125.9, 100.0, 98.6, 92.6, 92.4, 84.4, 79.7, 77.8, 76.2, 75.7, 74.8, 74.5, 73.8, 73.6, 72.8, 71.1, 70.2, 67.8, 67.1, 57.6 ppm.

Synthesis of S26

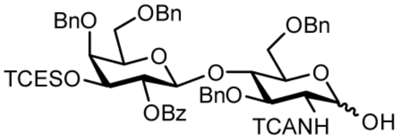

S26

2-*O*-benzoyl-4,6-di-*O*-benzyl-3-*O*-(2,2,2-trichloroethoxysulfonyl)-β-D-galactopyranosyl-(1 -> 4)-3,6-di-*O*-benzyl-2-deoxy-2-(trichloroacetamido)-β-D-glucopyranose (S26):

S26 was prepared from **S25** as a colorless oil, following the procedure for deprotecting **S21**.

S26: ^{1}H NMR (500 MHz, $CDCl_3$) δ = 7.99 – 7.95 (m, 2H), 7.63 – 7.56 (m, 1H), 7.48 – 7.44 (m, 2H), 7.44 – 7.40 (m, 2H), 7.37 – 7.26 (m, 11H), 7.25 – 7.21 (m, 4H), 7.20 – 7.16 (m, 1H), 7.16 – 7.10 (m, 2H), 6.79 (d, *J* = 8.7 Hz, 1H), 5.63 (dd, *J* = 10.3, 7.8 Hz, 1H), 5.30 (dd, *J* = 4.2, 3.3 Hz, 1H), 4.98 (d, *J* = 10.9 Hz, 1H), 4.86 (d, *J* = 11.4 Hz, 1H), 4.71 (d, *J* = 12.2 Hz, 1H), 4.68 (dd, *J* = 10.2, 3.2 Hz, 1H), 4.60 – 4.55 (m, 2H), 4.48 (d, *J* = 7.8 Hz, 1H), 4.45 (d, *J* = 10.9 Hz, 1H), 4.31 (d, *J* = 11.8 Hz, 1H), 4.29 – 4.25 (m, 2H), 4.24 – 4.20 (m, 2H), 4.11 – 4.06 (m, 1H), 4.00 (dd, *J* = 10.0, 8.8 Hz, 1H), 3.75 – 3.70 (m, 2H), 3.62 (dd, *J* = 10.8, 3.2 Hz, 1H), 3.45 (dd, *J* = 8.0, 5.7 Hz, 1H), 3.38 – 3.28 (m, 3H), 3.16 – 3.11 (m, 1H) ppm.

^{13}C NMR (126 MHz, $CDCl_3$) δ 165.0, 161.9, 138.5, 137.9, 137.83, 137.77, 133.9, 130.0, 128.89, 128.86, 128.7, 128.61, 128.60, 128.56, 128.5, 128.2, 128.01, 128.00, 127.96, 127.9, 127.4, 100.1, 91.1, 84.5, 79.7, 77.2, 76.6, 75.7, 75.1, 74.5, 73.8, 73.6, 72.8, 70.4, 70.3, 67.5, 67.1, 54.8 ppm.

Synthesis of 9a

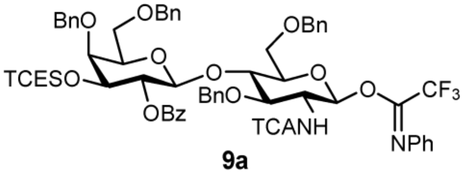

9a

2-*O*-benzoyl-4,6-di-*O*-benzyl-3-*O*-(2,2,2-trichloroethoxysulfonyl)-β-D-galactopyranosyl-(1 -> 4)-3,6-di-*O*-benzyl-2-deoxy-2-(trichloroacetamido)-β-D-glucopyranosyl 1-(*N*-phenyltrifluoroacetimidate) (9a):

A mixture of **S26** (83 mg, 0.071 mmol, 1.0 equiv.), 2,2,2-trifluoro-*N*-phenylacetimidoyl chloride (18 µL, 0.086 mmol, 1.2 equiv), and K_2CO_3 (20 mg, 0.076 mmol, 2.0 equiv.) in acetone (1 mL) was stirred at 22 °C for 6 hrs before it was filtered and concentrated under vacuum to give a crude mixture. The mixture was purified by flash chromatography (silica gel pre-washed with 5% Et_3N in hexane, hexane/EtOAc/Et_3N) to give trifluoroacetimidate **9a** as a colorless oil (78 mg, 0.058 mmol, 82%).

9a: ^{1}H NMR (500 MHz, $CDCl_3$) δ = 8.01 – 7.97 (m, 2H), 7.64 (td, *J* = 7.4, 1.3 Hz, 1H), 7.49 (t, *J* = 7.7 Hz, 2H), 7.47 – 7.15 (m, 22H), 7.07 – 6.99 (m, 1H), 6.70 (d, *J* = 7.8 Hz, 2H), 6.55 (d, *J* = 7.7 Hz, 1H), 6.40 (br s, 1H), 5.66 (dd, *J* = 10.2, 7.8 Hz, 1H), 5.02 (d, *J* = 11.1 Hz, 1H), 4.89 (d, *J* = 11.4 Hz, 1H), 4.76 (d, *J* = 12.1 Hz, 1H), 4.70 (dd, *J* = 10.2, 3.1 Hz, 1H), 4.64 (d, *J* = 11.1 Hz, 1H), 4.59 (d, *J* = 11.4 Hz, 1H), 4.56 (d, *J* = 7.9 Hz, 1H), 4.47 (d, *J* = 11.0 Hz, 1H), 4.38 – 4.25 (m, 5H), 4.24 – 4.11 (m, 2H), 3.74 (br t, *J* = 9.1 Hz, 1H), 3.64 (br d, *J* = 10.9 Hz, 1H), 3.58 – 3.46 (m, 2H), 3.45 – 3.34 (m, 3H) ppm.

Synthesis of 13

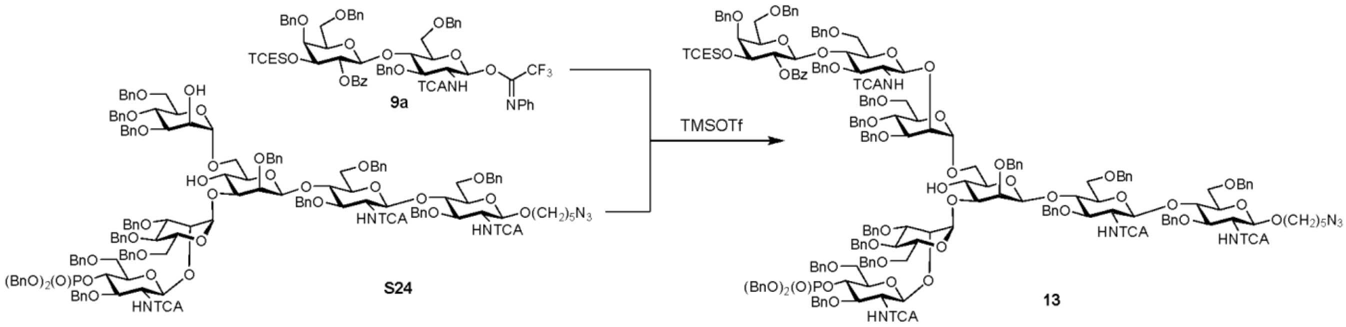

5-Azidopentyl 2-*O*-benzoyl-4,6-di-*O*-benzyl-3-*O*-(2,2,2-trichloroethoxysulfonyl)-β-D-galactopyranosyl-(1 -> 4)-3,6-di-*O*-benzyl-2-deoxy-2-(trichloroacetamido)-β-D-glucopyranosyl-(1 -> 2)-3,4,6-tri-*O*-benzyl-α-D-mannopyranosyl-(1 -> 6)-[4-*O*-dibenzylphosphoryl-3,6-di-*O*-benzyl-2-deoxy-2-(trichloroacet-amido)-β-D-glucopyranosyl-(1 -> 2)-3,4,6-tri-*O*-benzyl-α-D-mannopyranosyl-(1 -> 4)]-2-*O*-benzyl-β-D-mannopyranosyl-(1 -> 4)-3,6-di-*O*-benzyl-2-deoxy-2-(trichloroacetamido)-β-D-glucopyranosyl-(1 -> 4)-3,6-di-*O*-benzyl-2-deoxy-2-(trichloroacetamido)-β-D-glucopyranoside (13):

A mixture of **9a** (, 24 mg, 18 µmol, 1.8 equiv), **S24** (10 µmol, 1.0 equiv.), and flame-dried 4Å molecular sieves (100 mg/mL) in CH_2Cl_2 were stirred at −30 °C for 5 min before TMSOTf (1 uM, 20 µL, 0.2 equiv.) were added. The reaction mixture was kept at −30 °C – 0 °C for 40 min. The reaction was quenched by sequential addition of Et_3N, $NaHCO_3$ (sat. aq.) and $Na_2S_2O_3$ (s). The resulting mixture was stirred until its yellow color faded to white and then filtered to remove 4Å molecular sieves. The filtration was diluted with CH_2Cl_2 and extracted with CH_2Cl_2. The organic phases were combined, dried over Na_2SO_4, filtered, and concentrated under vacuum. The resulting residue was purified by flash chromatography (silica gel) to afford **13** in 69% as β anomer.

Synthesis of 19

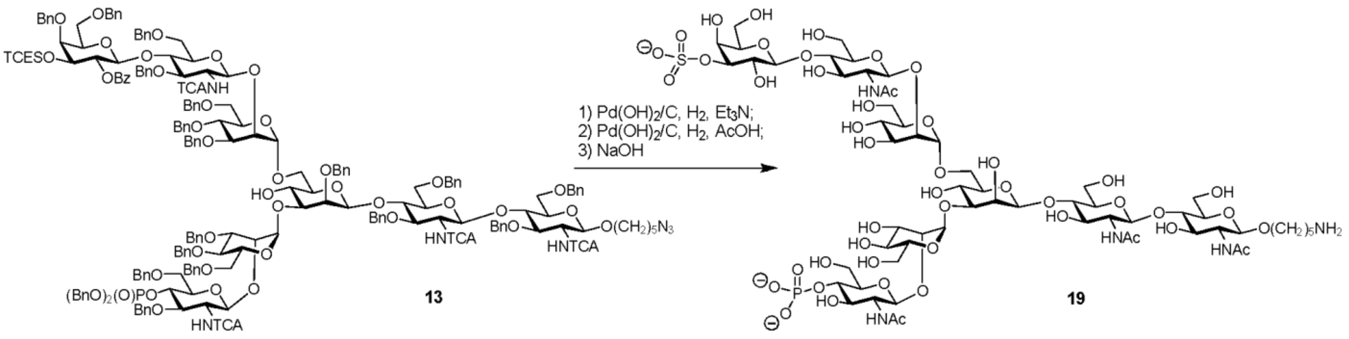

5-Aminopentyl 3-*O*-sulfo-β-D-galactopyranosyl-(1 -> 4)-2-acetamido-2-deoxy-β-D-glucopyranosyl-(1 -> 2)-α-D-mannopyranosyl-(1 -> 6)-[2-acetamido-2-deoxy-4-*O*-phosphoryl-β-D-glucopyranosyl-(1 -> 2)-α-D-mannopyranosyl-(1 -> 4)]-β-D-mannopyranosyl-(1 -> 4)-2-acetamido-2-deoxy-β-D-glucopyranosyl-(1 -> 4)-2-acetamido-2-deoxy-β-D-glucopyranoside (19)

Octasaccharide **19** was prepared from **11** (13 mg, 3.16 µmol) according to *General procedure VII* as a fluffy white solid (3.7 mg, 2.0 µmol, 63% over 3 steps).

19: ^{1}H NMR (600 MHz, D_2O) δ = 5.12 (s, 1H), 4.93 (d, *J* = 1.6 Hz, 1H), 4.77 (s, 1H), 4.64 – 4.54 (m, 4H), 4.50 (d, *J* = 7.7 Hz, 1H), 4.35 (dd, *J* = 9.9, 3.2 Hz, 1H), 4.30 (d, *J* = 3.3 Hz, 1H), 4.26 (d, *J* = 2.8 Hz, 1H), 4.19 (dd, *J* = 3.4, 1.6 Hz, 1H), 4.12 (dd, *J* = 3.7, 1.7 Hz, 1H), 4.03 – 3.55 (m, 41H), 3.54 – 3.45 (m, 4H), 2.99 (t, *J* = 7.6 Hz, 2H), 2.09 (s, 3H), 2.07 – 2.04 (m, 6H), 2.03 (s, 3H), 1.67 (quint, *J* = 7.7 Hz, 2H), 1.60 (quint, *J* = 6.6 Hz, 2H), 1.44 – 1.36 (m, 2H) ppm.

^{13}C NMR (151 MHz, D_2O) δ 174.7, 174.6 (2C), 174.4, 102.5, 101.4, 101.0, 100.3, 99.62, 99.58, 99.5, 96.9, 80.5, 80.0, 79.4, 79.3, 78.3, 76.5, 76.3, 75.40, 75.36 (d, *J* = 3.7 Hz), 74.9, 74.6, 74.5, 74.4, 74.2, 73.5, 73.4, 72.8, 72.42 (d, *J* = 4.1 Hz), 72.37, 72.0, 71.9, 70.15, 70.06, 69.4 (d, *J* = 4.5 Hz), 69.1, 67.33, 67.29, 66.8, 65.7, 65.6, 61.7, 61.6, 60.9, 60.5, 60.1, 59.9, 55.03, 54.95 (2C), 54.8, 39.3, 28.0, 26.3, 22.3 (2C), 22.2, 22.12, 22.06 ppm; ^{31}P NMR (162 MHz, D_2O) δ = 4.2 ppm.
HRMS (ESI-ToF) *m/z* calculated for $C_{61}H_{104}N_5O_{47}PS^{2-}$ $[M-2H]^{2-}$: 860.7686, found: 860.7574.

Scheme S9. Synthesis of 16 from 14 via sequential glycosylation reactions with donors 9b, 7, and 8b

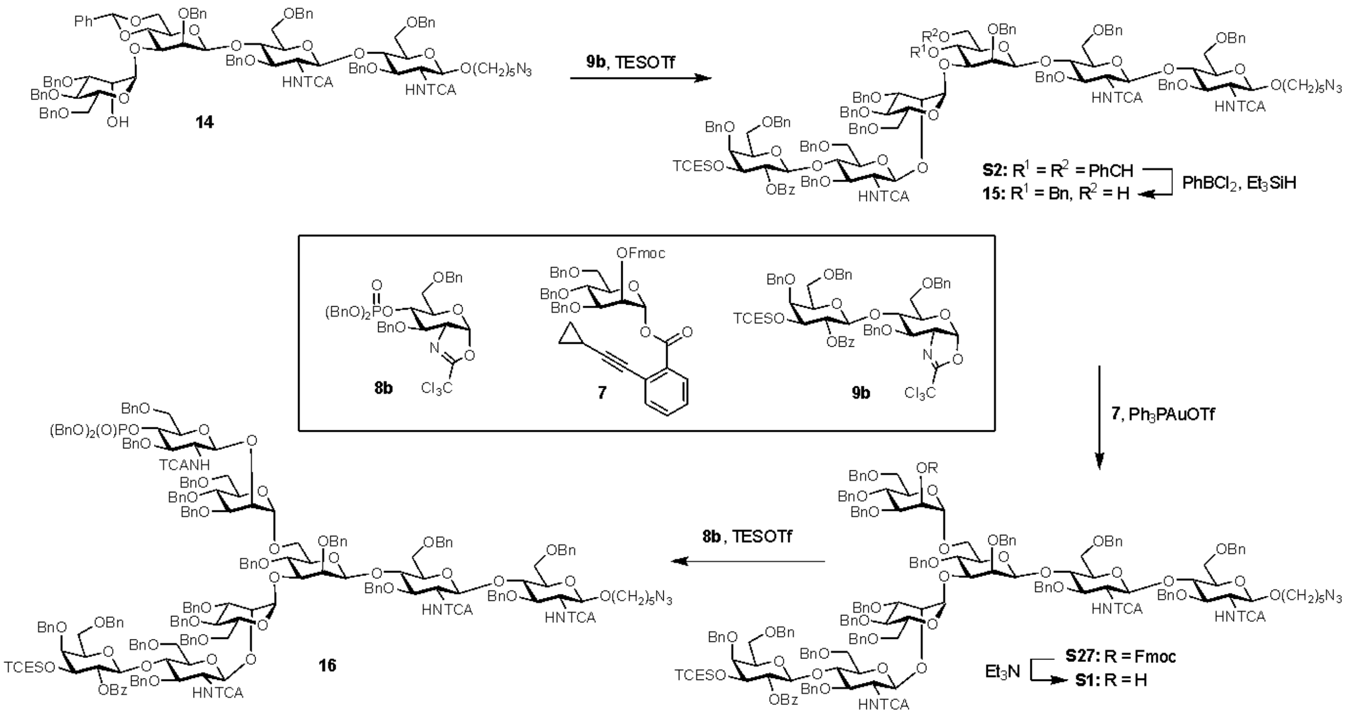

Synthesis of 9b

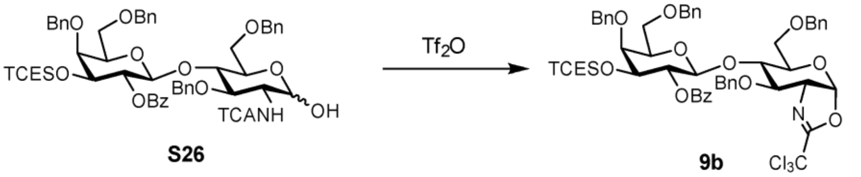

2-*O*-benzoyl-4,6-di-*O*-benzyl-3-*O*-(2,2,2-trichloroethoxysulfonyl)-β-D-galactopyranosyl-(1 -> 4)-3,6-di-*O*-benzyl-2-deoxy-2-(trichloroacetamido)-D-glucopyranosyl 1,2-oxazoline (9b):

To a mixture of **S26** (206 mg, 0.18 mmol, 1.0 equiv.) and Tf_2O (62 mg, 0.35 mmol, 2.0 equiv.) in MeCN (2 mL) was stirred at 0 °C for 30 min before Et_3N (100 μL, 0.72 mmol, 4.0 equiv.) was added. The reaction mixture was stirred at this temperature for 1 hr. The reaction was quenched with saturated $NaHCO_3$ (aq.) and partitioned. The organic phase was dried over Na_2SO_4, filtered, and concentrated under vacuum to give a crude mixture, which was purified by flash chromatography (silica gel saturated with 0.5% Et_3N in hexane, hexane/EtOAc) to give **9b** as a colorless glassy film (106 mg, 0.093 mmol, 52% yield, 72% *brsm.*) with 56 mg **S26** recovered.

9b: ^{1}H NMR (500 MHz, $CDCl_3$) δ = 7.96 (d, *J* = 7.7 Hz, 2H), 7.55 (t, *J* = 7.5 Hz, 1H), 7.42 – 7.20 (m, 22H), 6.23 (dd, *J* = 7.3, 1.6 Hz, 1H), 5.62 (dd, *J* = 10.1, 8.0 Hz, 1H), 4.88 (dd, *J* = 11.4, 1.6 Hz, 1H), 4.73

(dt, J = 10.3, 2.3 Hz, 1H), 4.66 (d, J = 12.0 Hz, 1H), 4.63 – 4.55 (m, 2H), 4.51 (d, J = 12.1 Hz, 1H), 4.49 – 4.42 (m, 2H), 4.40 – 4.33 (m, 3H), 4.33 – 4.22 (m, 4H), 4.04 (d, J = 8.9 Hz, 1H), 3.59 – 3.41 (m, 5H), 3.38 – 3.32 (m, 1H) ppm.

^{13}C NMR (126 MHz, $CDCl_3$) δ 164.5, 162.8, 138.0, 137.6, 137.5, 133.8, 130.0, 129.1, 128.8, 128.7, 128.60, 128.58, 128.4, 128.24, 128.18, 128.1, 128.0, 127.9, 127.7, 126.6, 104.1, 102.0, 84.4, 79.8, 77.3, 75.7, 75.5, 74.4, 73.7, 73.4, 73.1, 71.9, 70.5, 69.6, 68.5, 67.8, 66.2 ppm.

Synthesis of S2

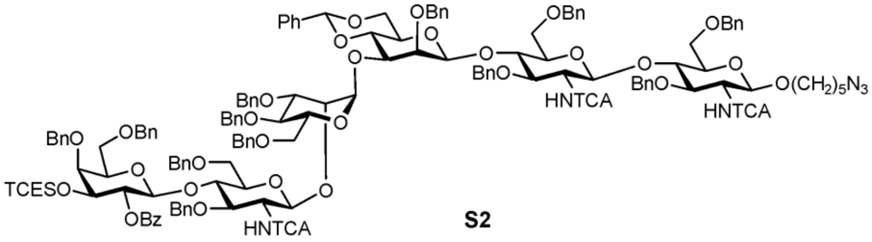

S2

5-Azidopentyl 4-*O*-(2,2,2-trichloroethoxysulfonyl)-β-D-galactopyranosyl-(1 -> 4)-3,6-di-*O*-benzyl-2-deoxy-2-(trichloroacetamido)-β-D-glucopyranosyl-(1 -> 2)-3,4,6-tri-*O*-benzyl-α-D-mannopyranosyl-(1 -> 4)-2-*O*-benzyl-β-D-mannopyranosyl-(1 -> 4)-3,6-di-*O*-benzyl-2-deoxy-2-(trichloroacetamido)-β-D-glucopyranosyl-(1 -> 4)-3,6-di-*O*-benzyl-2-deoxy-2-(trichloroacetamido)-β-D-glucopyranoside (S2)

S2 was prepared *according to General procedure V* from **14** (102 mg, 0.054 mmol, 1.0 equiv.) and **9b** (106 mg, 0.093 mmol, 1.7 equiv.) with TESOTf (3.7 µL, 0.016 mmol, 0.3 equiv.) as a colorless glassy film (121 mg, 0.047 mmol, 86%).

S2: ^{1}H NMR (500 MHz, $CDCl_3$) δ = 8.08 – 8.03 (m, 2H), 7.68 – 7.62 (m, 1H), 7.50 (t, *J* = 7.7 Hz, 2H), 7.42 – 7.08 (m, 65H), 6.93 (d, *J* = 7.9 Hz, 1H), 6.60 (d, *J* = 8.0 Hz, 1H), 6.35 (d, *J* = 8.2 Hz, 1H), 5.71 (dd, *J* = 10.2, 7.8 Hz, 1H), 5.19 (s, 1H), 5.06 – 5.00 (m, 3H), 4.96 – 4.88 (m, 3H), 4.82 – 4.66 (m, 8H), 4.63 – 4.57 (m, 3H), 4.52 (d, *J* = 12.1 Hz, 1H), 4.50 – 4.46 (m, 3H), 4.44 – 4.29 (m, 9H), 4.27 – 4.21 (m, 2H), 4.16 – 4.09 (m, 3H), 4.09 – 4.03 (m, 2H), 4.00 – 3.91 (m, 3H), 3.90 – 3.81 (m, 3H), 3.80 – 3.41 (m, 19H), 3.38 – 3.30 (m, 3H), 3.28 – 3.20 (m, 3H), 3.16 (br d, *J* = 9.3 Hz, 1H), 2.82 – 2.75 (m, 1H), 2.44 (d, *J* = 9.8 Hz, 1H), 1.64 – 1.52 (m, 4H), 1.47 – 1.35 (m, 2H) ppm.

Synthesis of 15

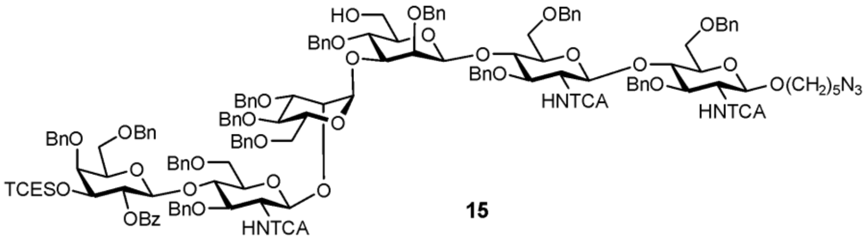

15

5-Azidopentyl 4-*O*-(2,2,2-trichloroethoxysulfonyl)-β-D-galactopyranosyl-(1 -> 4)-3,6-di-*O*-benzyl-2-deoxy-2-(trichloroacetamido)-β-D-glucopyranosyl-(1 -> 2)-3,4,6-tri-*O*-benzyl-α-D-mannopyranosyl-(1 -> 4)-2,4-di-*O*-benzyl-β-D-mannopyranosyl-(1 -> 4)-3,6-di-*O*-benzyl-2-deoxy-2-(trichloroacetamido)-β-D-glucopyranosyl-(1 -> 4)-3,6-di-*O*-benzyl-2-deoxy-2-(trichloroacetamido)-β-D-glucopyranoside (15):

To a mixture of **S5** (121 mg, 0.047 mmol, 1.0 equiv.), Et_3SiH (15 µL, 0.094 mmol, 2.0 equiv.) in CH_2Cl_2 (1 mL) at −78 °C was added $PhBCl_2$ (14 µL, 0.11 mmol, 2.4 equiv.). The reaction mixture was stirred at this temperature for 30 min. The reaction was quenched with Et_3N/MeOH 1/1 and saturated $NaHCO_3$ (aq.) at this temperature and then stirred at 22 °C for 10 min before the reaction mixture was partitioned. The organic phase was dried over Na_2SO_4, filtered, and concentrated under vacuum to give a crude mixture, which was purified by flash chromatography (silica gel, toluene/EtOAc) to give primary alcohol **15** as a colorless glassy film (96 mg, 0.037 mmol, 80%).

15: ^{1}H NMR (500 MHz, $CDCl_3$) δ = 8.06 (d, *J* = 7.6 Hz, 2H), 7.67 (t, *J* = 7.5 Hz, 1H), 7.53 (t, *J* = 7.7 Hz, 2H), 7.41 – 7.03 (m, 65H), 6.93 (d, *J* = 7.9 Hz, 1H), 6.59 – 6.50 (m, 2H), 5.70 (dd, *J* = 10.3, 7.9 Hz, 1H), 5.08 (d, *J* = 11.0 Hz, 1H), 5.03 (d, *J* = 1.8 Hz, 1H), 5.01 – 4.87 (m, 5H), 4.84 (d, *J* = 11.1 Hz, 1H), 4.82 – 4.69 (m, 5H), 4.68 – 4.25 (m, 21H), 4.22 (d, *J* = 12.0 Hz, 1H), 4.17 (d, *J* = 7.6 Hz, 1H), 4.13 (t, *J* = 8.0 Hz, 1H), 4.08 (d, *J* = 12.0 Hz, 1H), 4.01 – 3.90 (m, 4H), 3.90 – 3.80 (m, 4H), 3.78 – 3.71 (m, 3H), 3.70 – 3.33 (m, 18H), 3.29 – 3.19 (m, 4H), 3.17 (dt, *J* = 9.7, 2.7 Hz, 1H), 2.78 (dt, *J* = 9.4, 3.2 Hz, 1H), 2.56 (dt, *J* = 9.7, 2.6 Hz, 1H), 1.59 (quint, *J* = 7.0 Hz, 4H), 1.51 – 1.39 (m, 2H) ppm.

Synthesis of S27

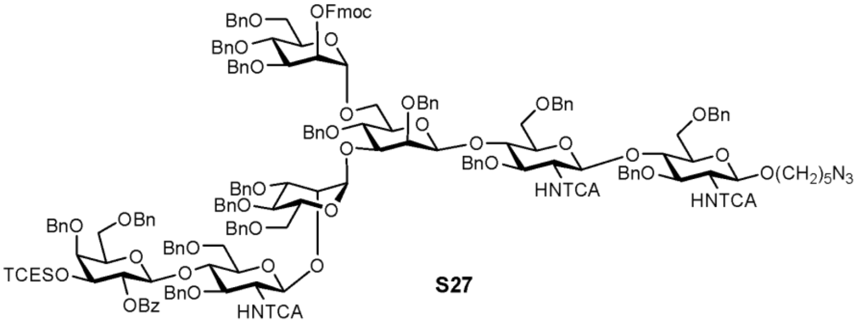

S27

5-Azidopentyl 4-*O*-(2,2,2-trichloroethoxysulfonyl)-β-D-galactopyranosyl-(1 -> 4)-3,6-di-*O*-benzyl-2-deoxy-2-(trichloroacetamido)-β-D-glucopyranosyl-(1 -> 2)-3,4,6-tri-*O*-benzyl-α-D-mannopyranosyl-(1 -> 4)-[2-*O*-fluorenylmethoxycarbonyl-3,4,6-tri-*O*-benzyl-α-D-mannopyranosyl-(1 -> 6)-]-2,4-di-*O*-benzyl-β-D-mannopyranosyl-(1 -> 4)-3,6-di-*O*-benzyl-2-deoxy-2-(trichloroacetamido)-β-D-glucopyranosyl-(1 -> 4)-3,6-di-*O*-benzyl-2-deoxy-2-(trichloroacetamido)-β-D-glucopyranoside (S27)

Heptasaccharide **S27** was prepared *according to General procedure IV* from **7** (60 mg, 0.072 mmol, 2.0 equiv.), **15** (93 mg, 0.036 mmol, 1.0 equiv.), $Ph_3PAuOTf$ prepared *in situ* (360 µL, 0.018 mmol, 0.5 equiv., 0.05 M), and CH_2Cl_2 (1 mL) at 0 °C as colorless glassy film (57 mg, 0.25 mmol, 50%). Flash chromatography column solvent: toluene/EtOAc 10/1 □8/1.

S27: ^{1}H NMR (700 MHz, $CDCl_3$) δ = 8.02 (d, *J* = 7.8 Hz, 2H), 7.76 (d, *J* = 7.6 Hz, 2H), 7.60 – 7.54 (m, 3H), 7.45 (t, *J* = 7.7 Hz, 2H), 7.41 – 7.06 (m, 81H), 7.00 (t, *J* = 7.4 Hz, 2H), 6.99 – 6.95 (m, 1H), 6.88 (d, *J* = 8.0 Hz, 1H), 6.53 (d, *J* = 7.7 Hz, 1H), 6.36 (d, *J* = 7.9 Hz, 1H), 5.68 (dd, *J* = 10.2, 7.9 Hz, 1H), 5.17 – 5.12 (m, 1H), 5.06 (d, *J* = 11.6 Hz, 1H), 4.99 – 4.78 (m, 8H), 4.75 (d, *J* = 11.9 Hz, 1H), 4.73 – 4.69 (m, 2H), 4.66 (d, *J* = 12.1 Hz, 1H), 4.62 – 4.49 (m, 11H), 4.49 – 4.34 (m, 10H), 4.33 – 4.23 (m, 7H), 4.19 (d, *J* = 8.2 Hz, 1H), 4.18 – 4.13 (m, 2H), 4.09 (dd, *J* = 10.2, 8.1 Hz, 1H), 4.06 – 4.02 (m, 2H), 3.97 – 3.90 (m, 3H), 3.90 – 3.79 (m, 7H), 3.74 (t, *J* = 9.6 Hz, 1H), 3.70 – 3.31 (m, 24H), 3.22 (t, *J* = 7.0 Hz, 2H), 3.19 (d, *J* = 10.7 Hz, 1H), 3.10 – 3.05 (m, 1H), 3.04 – 2.98 (m, 1H), 2.55 (d, *J* = 9.8 Hz, 1H), 1.66 – 1.52 (m, 4H), 1.47 – 1.32 (m, 2H) ppm.

^{13}C NMR (176 MHz, $CDCl_3$) δ 164.8, 161.8, 161.7, 161.6, 154.7, 143.7, 143.5, 141.4, 141.3, 138.9, 138.8, 138.72, 138.68, 138.52, 138.47, 138.42, 138.41, 138.10, 138.09, 138.0, 137.91, 137.86, 137.7, 134.0, 130.1, 129.1, 129.0, 128.8, 128.73, 128.68, 128.64, 128.60, 128.56, 128.5, 128.42, 128.39, 128.36, 128.32, 128.29, 128.25, 128.21, 128.19, 128.11, 128.07, 128.05, 128.02, 128.00, 127.99, 128.0, 127.93, 127.90,

127.86, 127.84, 127.79, 127.71, 127.69, 127.67, 127.61, 127.55, 127.53, 127.52, 127.49, 127.42, 127.3, 125.8, 125.7, 125.4, 120.10, 120.06, 101.4, 99.8, 99.74, 99.67, 99.1, 98.3, 97.6, 92.8, 92.63, 92.55, 92.4, 84.6, 81.0, 79.8, 78.43, 78.35, 78.20, 78.16, 77.9, 77.3, 77.2, 77.0, 76.1, 75.7, 75.5, 75.4, 75.1, 75.0, 74.9, 74.64, 74.62, 74.4, 74.3, 74.1, 73.7, 73.5, 73.4, 73.2, 72.9, 72.8, 72.2, 71.8, 70.3, 70.2, 68.9, 68.5, 67.7, 67.2, 66.9, 58.1, 57.6, 57.1, 46.7 29.2, 28.8, 23.4 ppm.

Synthesis of S1

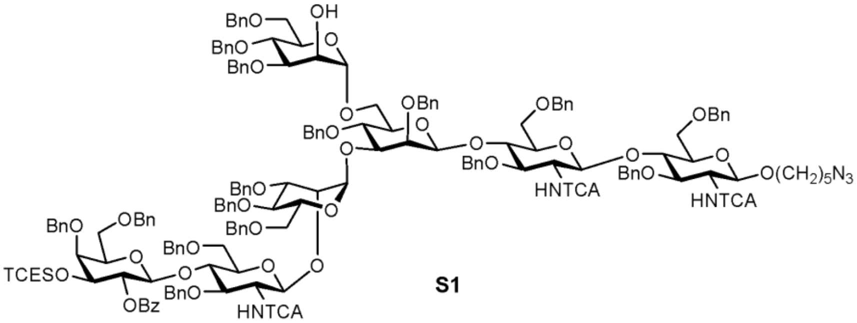

S1

5-Azidopentyl 4-*O*-(2,2,2-trichloroethoxysulfonyl)-β-D-galactopyranosyl-(1 -> 4)-3,6-di-*O*-benzyl-2-deoxy-2-(trichloroacetamido)-β-D-glucopyranosyl-(1 -> 2)-3,4,6-tri-*O*-benzyl-α-D-mannopyranosyl-(1 -> 4)-[3,4,6-tri-*O*-benzyl-α-D-mannopyranosyl-(1 -> 6)-]-2,4-di-*O*-benzyl-β-D-mannopyranosyl-(1 -> 4)-3,6-di-*O*-benzyl-2-deoxy-2-(trichloroacetamido)-β-D-glucopyranosyl-(1 -> 4)-3,6-di-*O*-benzyl-2-deoxy-2-(trichloroacetamido)-β-D-glucopyranoside (S1)

S1 was prepared *according to General procedure VII* from **S27**.

S1: ^{1}H NMR (500 MHz, $CDCl_3$) δ = 8.02 (d, *J* = 7.7 Hz, 2H), 7.60 (t, *J* = 7.4 Hz, 1H), 7.46 (t, *J* = 7.6 Hz, 2H), 7.40 – 7.04 (m, 77H), 7.03 – 6.94 (m, 3H), 6.92 (d, *J* = 8.0 Hz, 1H), 6.59 (d, *J* = 7.6 Hz, 1H), 5.67 (dd, *J* = 10.2, 7.8 Hz, 1H), 5.06 (d, *J* = 11.7 Hz, 1H), 5.00 – 4.17 (m, 42H), 4.10 – 4.02 (m, 2H), 3.99 – 3.41 (m, 32H), 3.39 – 3.32 (m, 3H), 3.22 (t, *J* = 7.0 Hz, 2H), 3.18 (br d, *J* = 11.0 Hz, 1H), 3.11 (br d, *J* = 9.3 Hz, 1H), 3.01 – 2.88 (m, 1H), 2.53 (br d, *J* = 9.7 Hz, 1H), 2.28 (br s, 1H), 1.61 – 1.53 (m, 4H), 1.45 – 1.35 (m, 2H) ppm.

Synthesis of 8b

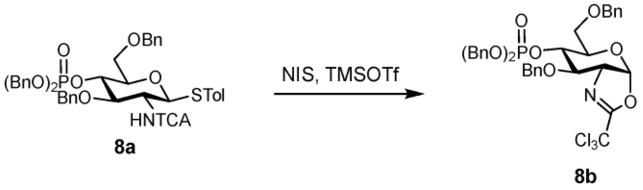

4-*O*-Dibenzylphosphoryl-3,6-di-*O*-benzyl-2-deoxy-2-(trichloroacetamido)-D-glucopyranosyl 1,2-oxazoline (8b):

A mixture of **8a** (112 mg, 0.13 mmol, 1.0 equiv.) and flame-dried 4Å molecular sieves (100 mg/mL) in CH_2Cl_2 (2 mL) were stirred at −50 °C for 5 min before NIS (35 mg, 0.15 mmol, 1.2 equiv.) and TMSOTf (5 µL, 0.026 mmol, 0.2 equiv.) were added. The reaction mixture was stirred at proper temperature for 30 min before it was lowered to below −70 °C and quenched sequentially with Et_3N, $NaHCO_3$ (sat. aq.) and $Na_2S_2O_3$ (s). The cooling bath was removed, and the resulting mixture was stirred until its yellow color faded to white. The resulting mixture was filtered to remove 4Å molecular sieves, diluted with CH_2Cl_2 and H_2O and extracted with CH_2Cl_2 (3 x 1 mL). The organic phases were combined, dried over Na_2SO_4, filtered, and concentrated under vacuum. The resulting residue was purified by flash chromatography (silica gel, hex/EtOAc 5/1 to 3/1) to afford **8b** as colorless film (98 mg, 0.10 mmol, 80%).

Synthesis of 16

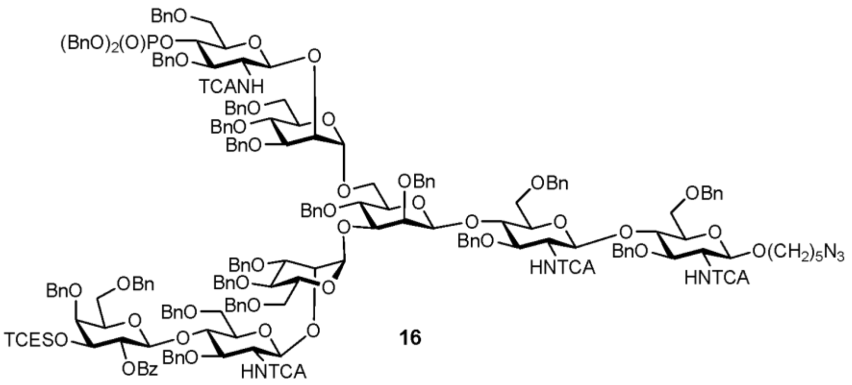

5-Azidopentyl 4-*O*-(2,2,2-trichloroethoxysulfonyl)-β-D-galactopyranosyl-(1 -> 4)-3,6-di-*O*-benzyl-2-deoxy-2-(trichloroacetamido)-β-D-glucopyranosyl-(1 -> 2)-3,4,6-tri-*O*-benzyl-α-D-mannopyranosyl-(1 -> 4)-[4-*O*-dibenzylphosphoryl-3,6-*O*-dibenzyl-2-deoxy-2-(trichloroacetamido)-β-D-glucopyranosyl-(1 -> 2)-3,4,6-tri-*O*-benzyl-α-D-mannopyranosyl-(1 -> 6)]-2,4-di-*O*-benzyl-β-D-mannopyranosyl-(1 -> 4)-3,6-di-*O*-benzyl-2-deoxy-2-(trichloroacetamido)-β-D-glucopyranosyl-(1 -> 4)-3,6-di-*O*-benzyl-2-deoxy-2-(trichloroacetamido)-β-D-glucopyranoside (16)

16 was prepared *according to General procedure V* from **S3** (38 mg, 0.051 mmol, 1.6 equiv.), **8b** (96 mg, 0.031 mmol, 1.0 equiv.), and TESOTf (3 μL, 0.01 mmol, 0.3 equiv.) in toluene (1 mL) as a colorless glassy film (82 mg, 0.02 mmol, 62%). Flash column solvent: toluene/EtOAc 10/1 8/1.

16: ^{1}H NMR (600 MHz, $CDCl_3$) δ = 8.04 – 7.96 (m, 2H), 7.59 (t, *J* = 7.5 Hz, 1H), 7.45 (t, *J* = 7.8 Hz, 2H), 7.39 – 7.03 (m, 96H), 7.02 – 6.90 (m, 6H), 6.53 (d, *J* = 7.7 Hz, 1H), 6.47 (d, *J* = 8.1 Hz, 1H), 5.67 (dd, *J* = 10.2, 7.8 Hz, 1H), 4.98 – 4.91 (m, 4H), 4.91 – 4.21 (m, 47H), 4.18 (d, *J* = 12.1 Hz, 1H), 4.14 – 4.08 (m, 2H), 4.02 (dt, *J* = 7.9, 4.8 Hz, 3H), 3.96 – 3.39 (m, 34H), 3.38 – 3.34 (m, 1H), 3.33 – 3.25 (m, 2H), 3.21 (t, *J* = 6.9 Hz, 2H), 3.20 – 3.14 (m, 2H), 3.12 (br d, *J* = 9.3 Hz, 1H), 3.08 – 3.01 (m, 1H), 2.49 (br d, *J* = 9.7 Hz, 1H), 1.59 – 1.52 (m, 4H), 1.46 – 1.32 (m, 2H) ppm.

^{13}C NMR (151 MHz, $CDCl_3$) δ = 164.7, 162.3, 161.8, 161.7, 161.6, 138.9, 138.8, 138.70, 138.67, 138.6, 138.5, 138.43, 138.37, 138.21, 138.16, 138.08, 138.06, 138.0, 137.88, 137.8, 137.7, 137.6, 135.9, 135.8, 135.7, 134.1, 130.0, 129.1, 128.9, 128.85, 128.80, 128.71, 128.68, 128.64, 128.62, 128.60, 128.56, 128.55, 128.51, 128.46, 128.42, 128.40, 128.37, 128.34, 128.30, 128.25, 128.23, 128.21, 128.11, 128.06, 128.01, 127.99, 127.96, 127.91, 127.86, 127.84, 127.79, 127.71, 127.69, 127.67, 127.63, 127.62, 127.59, 127.54, 127.49, 127.4, 125.8, 101.1, 99.8, 99.73, 99.69, 99.1, 99.0, 98.4, 97.0, 92.8, 92.63, 92.55, 92.4, 92.3, 84.6,

80.8, 79.7, 78.6, 78.5, 78.21, 78.17, 77.9, 76.4, 76.3, 76.0, 75.7, 75.5, 75.2, 75.13, 75.08, 75.01, 74.99, 74.85, 74.80, 74.65, 74.58, 74.5, 74.25, 74.19, 74.1, 74.05, 73.95, 73.7, 73.6, 73.5, 73.44, 73.40, 73.31, 73.25, 73.2, 72.9, 72.8, 72.5, 72.1, 72.0, 71.1, 70.22, 70.19, 69.61, 69.57, 69.5, 69.1, 68.7, 68.5, 67.8, 67.5, 67.2, 58.6, 58.1, 57.5, 57.2, 51.4, 29.2, 28.7, 23.4 ppm.

^{31}P NMR (202 MHz, $CDCl_3$) δ = −2.1 ppm.

Synthesis of 1

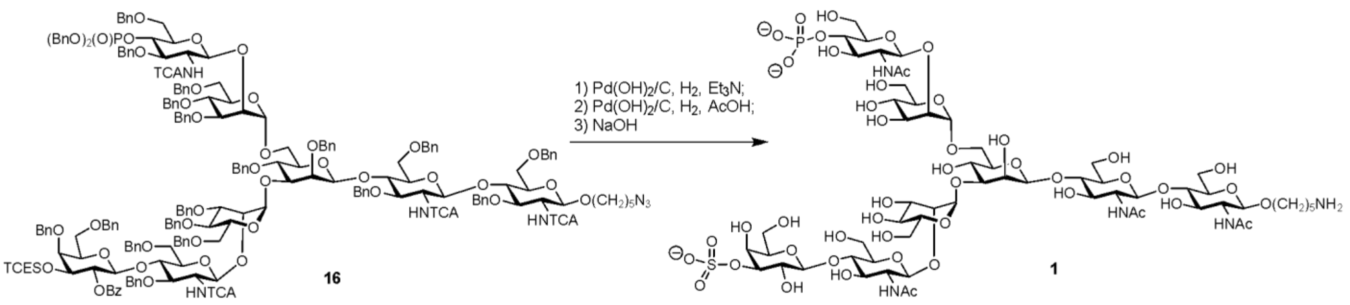

5-Aminopentyl 4-*O*-sulfo-β-D-galactopyranosyl-(1 -> 4)-2-acetamido-2-deoxy-β-D-glucopyranosyl-(1 -> 2)-α-D-mannopyranosyl-(1 -> 4)-[2-acetamido-2-deoxy-4-*O*-phosphono-β-D-glucopyranosyl-(1 -> 2)-α-D-mannopyranosyl-(1 -> 6)]-β-D-mannopyranosyl-(1 -> 4)-2-acetamido-2-deoxy-β-D-gluco-pyranosyl-(1 -> 4)-2-acetamido-2-deoxy-β-D-glucopyranoside (1)

1 was prepared from **16** (92 mg, 224 μmol) according to *General procedure VII* as fluffy white powders (23.2 mg, 123 μmol, 55% over three steps).

1: ^{1}H NMR (600 MHz, D_2O) δ = 5.09 (s, 1H), 4.91 (s, 1H), 4.74 (s, 1H), 4.63 – 4.50 (m, 4H), 4.46 (d, *J* = 7.4 Hz, 1H), 4.31 (dd, *J* = 10.0, 3.2 Hz, 1H), 4.27 (d, *J* = 3.3 Hz, 1H), 4.22 (d, *J* = 3.0 Hz, 1H), 4.17 (d, *J* = 2.6 Hz, 1H), 4.09 (d, *J* = 2.9 Hz, 1H), 3.99 – 3.54 (m, 41H), 3.52 – 3.41 (m, 4H), 2.96 (t, *J* = 7.7 Hz, 2H), 2.06 (s, 3H), 2.03 (s, 3H), 2.01 (s, 3H), 2.00 (s, 3H), 1.64 (quint, *J* = 7.8 Hz, 2H), 1.57 (quint, *J* = 6.9 Hz, 2H), 1.42 – 1.32 (m, 2H) ppm.

^{13}C NMR (151 MHz, D_2O) δ = 174.7, 174.6 (2C), 174.4, 102.4, 101.3, 101.0, 100.3, 99.5, 97.0, 80.4, 79.9, 79.3, 79.2, 78.2, 76.4, 76.3, 75.17, 75.14 (d, *J* = 3.7 Hz), 74.9, 74.6, 74.5, 74.3, 74.3, 73.5, 73.3, 72.92, 72.85 (d, *J* = 4.1 Hz), 72.8, 72.3, 71.9, 70.1, 70.0, 69.4, 69.3, 69.0, 67.3, 67.2, 66.7, 65.6, 65.5, 61.7, 61.6, 60.9, 60.4, 60.0, 59.8, 54.9 (3C), 54.8, 39.3, 28.0, 26.3, 22.3 (2C), 22.2, 22.1, 22.0 ppm; ^{31}P NMR (202 MHz, D_2O) δ = 2.9 ppm.

HRMS (ESI-ToF) *m/z* calculated for $C_{61}H_{104}N_5O_{47}PS^{2-}$ $[M-2H]^{2-}$: 860.7686, found: 860.7572.

2. Enzymatic Synthesis

Preparation of Enzymes

All the enzymes used in this study were expressed and purified as reported in the previous literature (Table S1).

Sulfatases from *Bacteroides thetaiotaomicron* (BT1636, 3057, 1628, and 1918) were prepared as active proteins with serine to cysteine mutants according to the literature.[6] BT1613 is stored at 5 °C and remains active for half a year.

Vs16 was prepared with mutants A235D and A145D.

Table S1. Enzymes used in this work.

Enzyme	Description	Source	Referenc e
BT1636	Sulfatase	*Bacteroides thetaiotaomicron*	(6)
BT1918			
BT3057			
BT3796			
β4GalT1	β1,4-galactosyltransferase	*Bos taurus*	(7)
Vs16	α2,3-sialyltransferase	*Vibrio sp.* (JT-FAJ-16)	(8)

Commercial Sources of Sugar Donors and Enzymes

UDP-GlcNAc, UDP-galactose, GDP-fucose, and CMP-sialic acid were purchased from BioChemSyn (Shanghai, China). Alkaline phosphatase from calf intestinal (CIAP) was purchased from Promega (WI, USA) with catalog No. M2825. Sulfatases from abalone entrails, *Helix pomatia*, *Aerobacter aerogenes*, *Patella vulgate* were purchased from Millipore Sigma (WI, USA).

Experimental Procedures

General procedure VIII: enzymatic hydrolysis of phosphate

The mixture of the substrate (final concentration 2-10 mM) and CIAP (1 u/µL, 0.2%wt of the substrate) in Tris-HCl buffer (pH 7.5, 50 mM) was incubated at 37 °C overnight. Upon the completion of the reaction, confirmed by TLC and/or LC-MS, it was quenched with an equal volume of cold MeOH. The resulting mixture was loaded onto a Sephadex G25 (fine) column, eluted with dH_2O. Fractions containing the product were lyophilized to give the product as a white, fluffy solid.

General procedure IX: enzymatic hydrolysis of sulfate

The mixture of the substrate (final concentration 2-10 mM), $MgCl_2$ (final concentration 10 mM), $CaCl_2$ (final concentration 10 mM), NaCl (final concentration 100 mM), and sulfatases (0.2~1%wt of the substrate) in Tris-HCl buffer (pH 7.5, 50 mM) was incubated at 37 °C overnight. Upon the completion of the reaction, confirmed by LC-MS, it was quenched with an equal volume of cold MeOH. The resulting mixture was loaded onto a P2 Bio-Gel® (fine) column, eluted with dH_2O. Fractions containing the product were lyophilized to give the product as a white, fluffy solid.

General procedure X: enzymatic β–1,4-N-acetylglucosaminyltransferase

The mixture of the substrate (final concentration 2 mM), UDP-GlcNAc (1.2 equiv., final concentration 2.4 mM), $MnCl_2$ (final concentration of 10 mM), GnT-IV or GnT-V (0.2%wt of the substrate) in Tris-HCl buffer (pH 7.5, 50 mM) was incubated at 37 °C overnight. Upon the completion of the reaction, confirmed by LC-MS, it was quenched with an equal volume of cold MeOH. The resulting mixture was concentrated under vacuum, loaded onto Sephadex G25 (fine) column, and eluted with dH_2O. Fractions containing the product were lyophilized to give the product as a white, fluffy solid.

General procedure XI: enzymatic β–1,4-galactosylation

The mixture of the substrate (final concentration 2 mM), UDP-Gal (1.2 equiv. per glycosylation), $MnCl_2$ (final concentration of 10 mM), bovine ®1,4-GalT (0.2%wt of the substrate) in Tris-HCl buffer (pH 7.5, 50 mM) was incubated at 37 °C overnight. Upon the completion of the reaction, confirmed by LC-MS, it was quenched with an equal volume of cold MeOH. The resulting mixture was concentrated under vacuum, loaded onto Sephadex G25 (fine) column, and eluted with dH_2O. Fractions containing the product were lyophilized to give the product as a white, fluffy solid.

General procedure XII: enzymatic a2,3- and α2,6-sialylation

The mixture of the substrate (final concentration 2 mM), CMP-Sia (1.2 equiv. per glycosylation), $MgCl_2$ (final concentration of 10 mM), sialyltransferase (0.2%wt of the substrate) in Tris-HCl buffer (pH 7.5, 50 mM) was incubated at 37 °C overnight. Upon the completion of the reaction, confirmed by LC-MS, it was quenched with an equal volume of cold MeOH. The resulting mixture was concentrated under vacuum, loaded onto Sephadex G25 (fine) column, and eluted with dH_2O. Fractions containing the product were lyophilized to give the product as a white, fluffy solid.

Enzymatic synthesis of 20

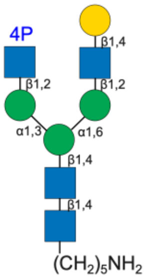

20

5-Aminopentyl β-D-galactopyranosyl-(1 -> 4)-2-acetamido-2-deoxy-β-D-glucopyranosyl-(1 -> 2)-α-D-mannopyranosyl-(1 -> 6)-[2-acetamido-2-deoxy-4-*O*-phosphono-β-D-glucopyranosyl-(1 -> 2)-α-D-mannopyranosyl-(1 -> 3)]-β-D-mannopyranosyl-(1 -> 4)-2-acetamido-2-deoxy-β-D-glucopyranosyl-(1 -> 4)-2-acetamido-2-deoxy-β-D-glucopyranoside (20) was prepared from **19** (1.3 mg, 2.0 umol) based on General procedure IX as fluffy powders. HRMS (ESI) *m/z* calculated for $C_{61}H_{104}N_5O_{44}P^{2-}$ $[M-2H]^{2-}$: 820.7901, found: 820.7853.

Enzymatic synthesis of 21

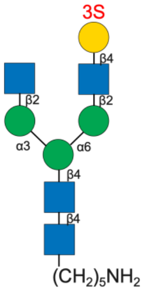

21

5-Aminopentyl 3-*O*-sulfo-β-D-galactopyranosyl-(1 -> 4)-2-acetamido-2-deoxy-β-D-glucopyranosyl-(1 -> 2)-α-D-mannopyranosyl-(1 -> 6)-[2-acetamido-2-deoxy-β-D-glucopyranosyl-(1 -> 2)-α-D-mannopyranosyl-(1 -> 3)]-β-D-mannopyranosyl-(1 -> 4)-2-acetamido-2-deoxy-β-D-glucopyranosyl-(1 -> 4)-2-acetamido-2-deoxy-β-D-glucopyranoside (21) was prepared from **19** (1.3 mg, 2.0 umol) based on General procedure VIII as fluffy powders.

21: ^{1}H NMR (600 MHz, D_2O) δ = 5.12 (s, 1H), 4.93 (s, 1H), 4.76 (s, 1H), 4.64 – 4.57 (m, 3H), 4.56 (d, *J* = 8.4 Hz, 1H), 4.50 (d, *J* = 7.5 Hz, 1H), 4.35 (dd, *J* = 10.0, 3.3 Hz, 1H), 4.30 (d, *J* = 3.3 Hz, 1H), 4.25 (d, *J* = 2.9 Hz, 1H), 4.21 – 4.18 (m, 1H), 4.14 – 4.10 (m, 1H), 4.03 – 3.83 (m, 11H), 3.83 – 3.53 (m, 29H), 3.53 – 3.41 (m, 5H), 2.99 (t, *J* = 7.3 Hz, 2H), 2.09 (s, 3H), 2.06 (s, 3H), 2.05 (s, 3H), 2.03 (s, 3H), 1.67

(quint, J = 7.7 Hz, 2H), 1.60 (quint, J = 6.7 Hz, 2H), 1.44 – 1.35 (m, 2H) ppm. HRMS (ESI) *m/z* calculated for $C_{61}H_{103}N_5O_{44}S^{2-}$ [M-2H]$^{2-}$: 820.7854, found: 820.7838.

Enzymatic synthesis of 22

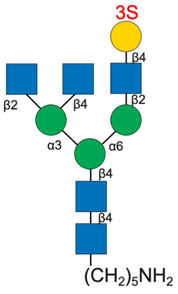

22

5-Aminopentyl 3-*O*-sulfo-β-D-galactopyranosyl-(1 -> 4)-2-acetamido-2-deoxy-β-D-glucopyranosyl-(1 -> 2)-α-D-mannopyranosyl-(1 -> 6)-[2-acetamido-2-deoxy-β-D-glucopyranosyl-(1 -> 2)-[2-acetamido-2-deoxy-β-D-glucopyranosyl-(1 -> 4)]-α-D-mannopyranosyl-(1 -> 3)]-β-D-mannopyranosyl-(1 -> 4)-2-acetamido-2-deoxy-β-D-glucopyranosyl-(1 -> 4)-2-acetamido-2-deoxy-β-D-glucopyranoside (22) was prepared from **21** based on General procedure X as fluffy powders.

22: ^{1}H NMR (700 MHz, D_2O) δ = 5.13 (s, 1H), 4.94 (s, 1H), 4.77 (s, 1H), 4.63 – 4.57 (m, 3H), 4.56 – 4.52 (m, 2H), 4.51 (d, J = 7.5 Hz, 1H), 4.38 – 4.34 (m, 1H), 4.31 (d, J = 3.3 Hz, 1H), 4.24 – 4.20 (m, 2H), 4.13 (s, 1H), 4.06 (d, J = 9.3 Hz, 1H), 4.02 – 3.41 (m, 50H), 2.91 (t, J = 7.6 Hz, 2H), 2.10 (s, 3H), 2.09 (s, 3H), 2.06 (s, 3H), 2.06 (s, 3H), 2.04 (s, 3H), 1.67 – 1.56 (m, 4H), 1.42 – 1.35 (m, 2H) ppm.

Enzymatic synthesis of 23

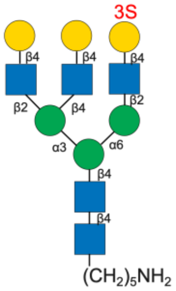

23

5-Aminopentyl 3-*O*-sulfo-β-D-galactopyranosyl-(1 -> 4)-2-acetamido-2-deoxy-β-D-glucopyranosyl-(1 -> 2)-α-D-mannopyranosyl-(1 -> 6)-[β-D-galactopyranosyl-(1 -> 4)-2-acetamido-2-deoxy-β-D-glucopyranosyl-(1 -> 2)-[β-D-galactopyranosyl-(1 -> 4)-2-acetamido-2-deoxy-β-D-glucopyranosyl-(1 -> 4)]-α-D-mannopyranosyl-(1 -> 3)]-β-D-mannopyranosyl-(1 -> 4)-2-acetamido-2-deoxy-β-D-glucopyranosyl-(1 -> 4)-2-acetamido-2-deoxy-β-D-glucopyranoside (23) was prepared from **22** based on General procedure XI as fluffy powders.

Enzymatic synthesis of 24

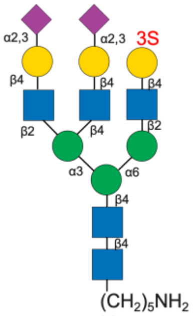

24

5-Aminopentyl α-*N*-acetylneuraminyl-(2 -> 3)-β-D-galactopyranosyl-(1 -> 4)-2-acetamido-2-deoxy-β-D-glucopyranosyl-(1 -> 2)-[α-*N*-acetylneuraminyl-(2 -> 3)-β-D-galactopyranosyl-(1 -> 4)-2-acetamido-2-deoxy-β-D-glucopyranosyl-(1 -> 4)]-α-D-mannopyranosyl-(1 -> 3)-[3-O-sulfo-β-D-galactopyranosyl-(1 -> 4)-2-acetamido-2-deoxy-β-D-glucopyranosyl-(1 -> 2)-α-D-mannopyranosyl-(1 -> 6)-α-D-mannopyranosyl-(1 -> 3)]-β-D-mannopyranosyl-(1 -> 4)-2-acetamido-2-deoxy-β-D-glucopyranosyl-(1 -> 4)-2-acetamido-2-deoxy-β-D-glucopyranoside (24) was prepared from **23** based on General procedure XII as fluffy powders. HRMS (ESI-ToF) *m/z* calculated for $C_{103}H_{169}N_8O_{75}S^{3-}$ [M-3H]$^{3-}$: 916.6464, found: 916.9709.

Enzymatic synthesis of 25

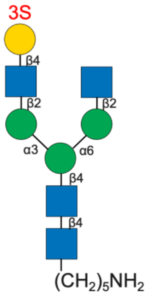

25

5-Aminopentyl 3-*O*-sulfo-β-D-galactopyranosyl-(1 -> 4)-2-acetamido-2-deoxy-β-D-glucopyranosyl-(1 -> 2)-α-D-mannopyranosyl-(1 -> 3)-[2-acetamido-2-deoxy-β-D-glucopyranosyl-(1 -> 2)-α-D-mannopyranosyl-(1 -> 6)]-β-D-mannopyranosyl-(1 -> 4)-2-acetamido-2-deoxy-β-D-glucopyranosyl-(1 -> 4)-2-acetamido-2-deoxy-β-D-glucopyranoside (25) was prepared from **1** based on General procedure VIII as fluffy powders.

25: ^{1}H NMR (600 MHz, D_2O) δ = 5.00 (s, 1H), 4.80 (s, 1H), 4.66 (s, 1H), 4.50 – 4.45 (m, 3H), 4.44 (d, *J* = 8.4 Hz, 1H), 4.37 (d, *J* = 7.7 Hz, 1H), 4.22 (dd, *J* = 9.9, 3.3 Hz, 1H), 4.18 (d, *J* = 3.3 Hz, 1H), 4.16 – 4.12 (m, 1H), 4.08 (d, *J* = 3.2 Hz, 1H), 4.01 – 3.97 (m, 1H), 3.91 – 3.28 (m, 45H), 2.87 (t, *J* = 7.7 Hz, 2H),

1.97 (s, 3H), 1.94 (s, 6H), 1.91 (s, 3H), 1.55 (quint, J = 7.7 Hz, 2H), 1.47 (quint, J = 6.6 Hz, 2H), 1.32 – 1.23 (m, 2H) ppm; ^{13}C NMR (151 MHz, D_2O) δ = 174.7, 174.64, 174.61, 174.4, 102.4, 101.3, 101.0, 100.4, 99.50, 99.49, 99.4, 97.0, 80.4, 79.9, 79.5, 79.2, 78.2, 76.3, 76.2, 75.7, 74.9, 74.7, 74.5, 74.29, 74.27, 73.5, 73.3, 72.8, 72.3, 71.91, 71.87, 70.1, 70.0, 69.8, 69.4, 69.3, 69.1, 67.3, 67.2, 66.7, 65.9, 65.7, 61.7, 61.6, 60.9, 60.5, 60.0, 59.9, 59.8, 55.3, 54.9, 54.8, 39.2, 28.0, 26.3, 22.3, 22.2, 22.14, 22.06, 22.0 ppm.

Enzymatic synthesis of 26

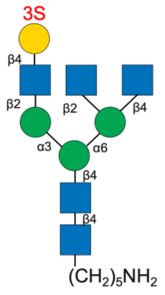

26

5-Aminopentyl 3-*O*-sulfo-β-D-galactopyranosyl-(1 -> 4)-2-acetamido-2-deoxy-β-D-glucopyranosyl-(1 -> 2)-α-D-mannopyranosyl-(1 -> 3)-[2-acetamido-2-deoxy-β-D-glucopyranosyl-(1 -> 2)]-[2-acetamido-2-deoxy-β-D-glucopyranosyl-(1 -> 4)]-α-D-mannopyranosyl-(1 -> 6)]-β-D-mannopyranosyl-(1 -> 4)-2-acetamido-2-deoxy-β-D-glucopyranosyl-(1 -> 4)-2-acetamido-2-deoxy-β-D-glucopyranoside (26) was prepared from **25** based on General procedure XI as fluffy powders.

26: ^{1}H NMR (700 MHz, D_2O) δ = 5.15 (s, 1H), 4.89 (s, 1H), 4.79 (s, 1H), 4.64 – 4.59 (m, 3H), 4.58 (d, J = 8.4 Hz, 1H), 4.56 (d, J = 8.4 Hz, 1H), 4.51 (d, J = 7.5 Hz, 1H), 4.36 (dd, J = 9.9, 3.2 Hz, 1H), 4.32 (d, J = 3.3 Hz, 1H), 4.29 – 4.26 (m, 1H), 4.24 – 4.20 (m, 2H), 4.13 – 4.09 (m, 1H), 4.01 (br d, J = 12.0 Hz, 1H), 3.98 – 3.40 (m, 49H), 3.00 (t, J = 7.7 Hz, 2H), 2.10 (s, 3H), 2.08 (s, 3H), 2.07 (s, 3H), 2.06 (s, 3H), 2.05 (s, 3H), 1.69 (quint, J = 7.8 Hz, 2H), 1.62 (quint, J = 7.0 Hz, 2H), 1.47 – 1.36 (m, 2H) ppm.
HRMS (ESI-ToF) *m/z* calculated for $C_{69}H_{116}N_6O_{49}S^{2-}$ $[M-2H]^{2-}$: 922.3251, found: 922.8133.

Enzymatic synthesis of 27

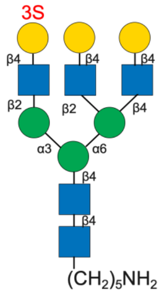

27

5-Aminopentyl 3-*O*-sulfo-β-D-galactopyranosyl-(1 -> 4)-2-acetamido-2-deoxy-β-D-glucopyranosyl-(1 -> 2)-α-D-mannopyranosyl-(1 -> 3)-[β-D-galactopyranosyl-(1 -> 4)-2-acetamido-2-deoxy-β-D-glucopyranosyl-(1 -> 2)]-[β-D-galactopyranosyl-(1 -> 4)-2-acetamido-2-deoxy-β-D-glucopyranosyl-(1 -> 4)]-α-D-mannopyranosyl-(1 -> 6)]-β-D-mannopyranosyl-(1 -> 4)-2-acetamido-2-deoxy-β-D-glucopyranosyl-(1 -> 4)-2-acetamido-2-deoxy-β-D-glucopyranoside (27) was prepared from **26** based on General procedure XI as fluffy powders.

27: ^{1}H NMR (700 MHz, D_2O) δ = 5.14 (s, 1H), 4.88 (s, 1H), 4.78 (s, 1H), 4.63 – 4.58 (m, 4H), 4.56 (d, *J* = 8.2 Hz, 1H), 4.51 – 4.48 (m, 3H), 4.35 (dd, *J* = 9.9, 3.3 Hz, 1H), 4.31 (d, *J* = 3.3 Hz, 1H), 4.29 – 4.18 (m, 5H), 4.13 – 4.09 (m, 1H), 4.06 – 3.46 (m, 59H), 3.42 (t, *J* = 9.8 Hz, 1H), 2.99 (t, *J* = 7.6 Hz, 2H), 2.10 (s, 3H), 2.07 (s, 3H), 2.06 (s, 3H), 2.05 (s, 3H), 2.04 (s, 3H), 1.68 (quint, *J* = 7.8 Hz, 2H), 1.60 (quint, *J* = 6.9 Hz, 2H), 1.45 – 1.37 (m, 2H) ppm. HRMS (ESI-ToF) *m/z* calculated for $C_{81}H_{136}N_6O_{59}S^{2-}$[M-2H]$^{2-}$: 1084.3779, found: 1084.3636.

Enzymatic synthesis of 28

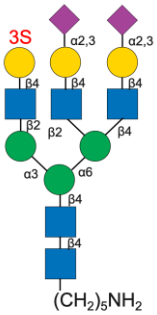

28

5-Aminopentyl α-*N*-acetylneuraminyl-(2 -> 3)-β-D-galactopyranosyl-(1 -> 4)-2-acetamido-2-deoxy-β-D-glucopyranosyl-(1 -> 2)-[α-*N*-acetylneuraminyl-(2 -> 3)-β-D-galactopyranosyl-(1 -> 4)-2-acetamido-2-deoxy-β-D-glucopyranosyl-(1 -> 4)]-α-D-mannopyranosyl-(1 -> 6)-[3-*O*-sulfo-β-D-galactopyranosyl-(1 -> 4)-2-acetamido-2-deoxy-β-D-glucopyranosyl-(1 -> 2)-α-D-mannopyranosyl-(1 -> 3)-α-D-mannopyranosyl-(1 -> 3)]-β-D-mannopyranosyl-(1 -> 4)-2-acetamido-2-deoxy-β-D-glucopyranosyl-(1 -> 4)-2-acetamido-2-deoxy-β-D-glucopyranoside (28) was prepared from **27** based on General procedure XII as fluffy powders.

28: ^{1}H NMR (700 MHz, D_2O) δ = 5.14 (s, 1H), 4.89 (s, 1H), 4.78 (s, 1H), 4.62 – 4.54 (m, 7H), 4.50 (d, *J* = 7.5 Hz, 1H), 4.35 (dd, *J* = 9.9, 3.2 Hz, 1H), 4.31 (d, *J* = 3.3 Hz, 1H), 4.28 – 4.25 (m, 1H), 4.22 (d, *J* = 13.5 Hz, 2H), 4.17 – 4.08 (m, 3H), 4.06 – 3.46 (m, 73H), 3.41 (td, *J* = 9.8, 5.0 Hz, 1H), 3.00 (t, *J* = 7.6

Hz, 2H), 2.77 (dd, J = 12.6, 4.7 Hz, 2H), 2.10 (s, 3H), 2.07 (s, 3H), 2.06 – 2.02 (m, 15H), 1.82 (t, J = 12.3 Hz, 2H), 1.68 (quint, J = 7.7 Hz, 2H), 1.60 (quint, J = 7.0 Hz, 2H), 1.44 – 1.37 (m, 2H) ppm.

Enzymatic synthesis of 29

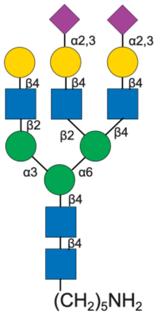

29

5-Aminopentyl α-*N*-acetylneuraminyl-(2 -> 3)-β-D-galactopyranosyl-(1 -> 4)-2-acetamido-2-deoxy-β-D-glucopyranosyl-(1 -> 2)-[α-*N*-acetylneuraminyl-(2 -> 3)-β-D-galactopyranosyl-(1 -> 4)-2-acetamido-2-deoxy-β-D-glucopyranosyl-(1 -> 4)]-α-D-mannopyranosyl-(1 -> 6)-[β-D-galactopyranosyl-(1 -> 4)-2-acetamido-2-deoxy-β-D-glucopyranosyl-(1 -> 2)-α-D-mannopyranosyl-(1 -> 3)-α-D-mannopyranosyl-(1 -> 3)]-β-D-mannopyranosyl-(1 -> 4)-2-acetamido-2-deoxy-β-D-glucopyranosyl-(1 -> 4)-2-acetamido-2-deoxy-β-D-glucopyranoside (29) was prepared from **28** based on General procedure IX as fluffy powders.

27: ^{1}H NMR (600 MHz, D_2O) δ = 5.10 (s, 1H), 4.84 (s, 1H), 4.78 (s, 1H), 4.60 – 4.48 (m, 6H), 4.45 (d, J = 7.8 Hz, 1H), 4.43 (d, J = 7.8 Hz, 1H), 4.23 – 4.20 (m, 1H), 4.20 – 4.15 (m, 2H), 4.12 – 4.04 (m, 4H), 4.01 – 3.39 (m, 74H), 3.39 – 3.33 (m, 1H), 2.95 (t, J = 7.7 Hz, 2H), 2.72 (dd, J = 12.5, 4.6 Hz, 2H), 2.05 (s, 3H), 2.02 (s, 3H), 2.01 – 1.97 (m, 15H), 1.77 (t, J = 12.0 Hz, 2H), 1.63 (quint, J = 7.8 Hz, 2H), 1.56 (quint, J = 6.6 Hz, 2H), 1.39 – 1.32 (m, 2H) ppm.

Enzymatic synthesis of 34

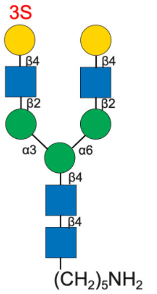

34

5-Aminopentyl 3-*O*-sulfo-β-D-galactopyranosyl-(1 -> 4)-2-acetamido-2-deoxy-β-D-glucopyranosyl-(1 -> 2)-α-D-mannopyranosyl-(1 -> 3)-[β-D-galactopyranosyl-(1 -> 4)-2-acetamido-2-deoxy-β-D-glucopyranosyl-(1 -> 2)-α-D-mannopyranosyl-(1 -> 6)]-β-D-mannopyranosyl-(1 -> 4)-2-acetamido-2-deoxy-β-D-glucopyranosyl-(1 -> 4)-2-acetamido-2-deoxy-β-D-glucopyranoside (34) was prepared from **25** based on General procedure XI as fluffy powders.

34: ^{1}H NMR (600 MHz, D_2O) δ = 5.09 (s, 1H), 4.90 (s, 1H), 4.78 (s, 1H), 4.59 – 4.53 (m, 4H), 4.50 – 4.42 (m, 2H), 4.31 (dd, *J* = 9.9, 3.3 Hz, 1H), 4.27 (d, *J* = 3.3 Hz, 1H), 4.24 – 4.22 (m, 1H), 4.18 – 4.15 (m, 1H), 4.12 – 4.06 (m, 1H), 4.00 – 3.43 (m, 51H), 2.96 (t, *J* = 7.7 Hz, 2H), 2.06 (s, 3H), 2.03 (s, 3H), 2.02 (s, 3H), 2.00 (s, 3H), 1.64 (quint, *J* = 7.7 Hz, 2H), 1.57 (quint, 2H), 1.41 – 1.33 (m, 2H) ppm. HRMS (ESI-ToF) *m/z* calculated for $C_{67}H_{113}N_5O_{49}S^{2-}$ [M-2H]$^{2-}$: 901.8118, found: 901.7985.

Enzymatic synthesis of 35 and 36 from 1 via three-steps synthesis

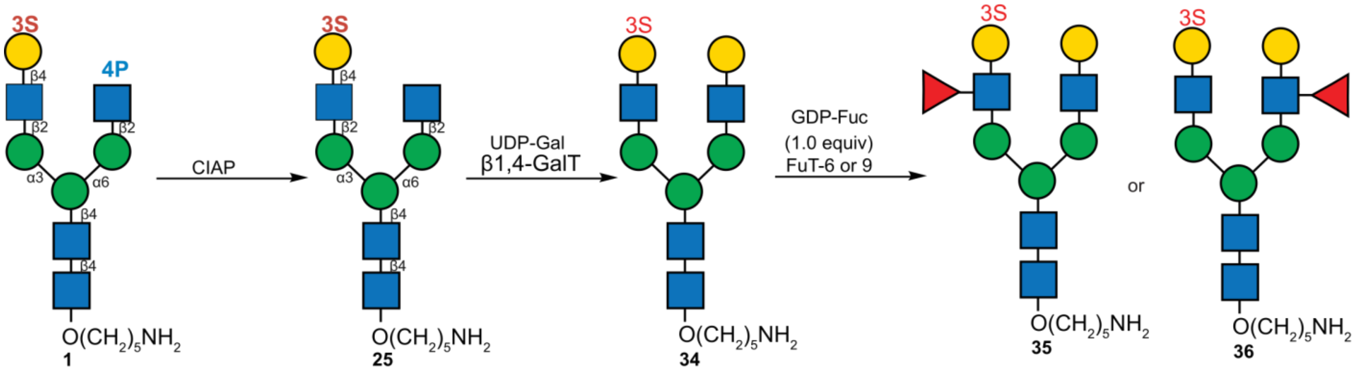

To avoid over-fucosylations of **34** when using **an** excess amount of GDP-Fuc on a small-scale reaction, three-step enzymatic modifications of **1** without isolating the intermediates were adapted to minimize the excess of Fuc-GDP.

Enzymatic reactions on 1 mediated by CIAP and ®1,4-GalT:

1 (6.7 mM, 190 µL 2.2 mg, 1.3 µmol) was mixed with Tris-HCl (pH 7.5, 1M, 21 µL) to make a final concentration of 6 mM in Tris-HCl (pH 7.5, 85.8 mM). The solution was treated with CIAP (1 u/µL, 4 µL).

Upon the completion of dephosphorylation confirmed by LC-MS, UDP-Gal (20 mM in dH_2O, 74 µL, 1.5 µmol, 1.15 equiv. to **1**), Tris-HCl buffer (pH 7.5, 1M, 19 µL), $MnCl_2$ (200 mM, 8 µL), and bovine ®1,4-GalT (1.98 mg/mL, 2 µL, 4.4 µg, 2‰wt of **1**) were added to make a total volume of 318 µL. The final concentration of the glycan is 4 mM, $MnCl_2$ 5 mM. The reaction mixture was incubated at 37 °C overnight. Upon the completion of the reaction, confirmed by LC-MS, the reaction mixture was centrifuged, and the

liquid was transferred to a new 1 mL Eppendorf tube and equally divided into two portions for the pending fucosylation reactions.

For the synthesis of **5-aminopentyl 3-*O*-sulfo-β-D-galactopyranosyl-(1 -> 4)-[α-L-fucopyranosyl-(1 -> 3)]-2-acetamido-2-deoxy-β-D-glucopyranosyl-(1 -> 2)-α-D-mannopyranosyl-(1 -> 3)-[β-D-galactopyranosyl-(1 -> 4)-2-acetamido-2-deoxy-β-D-glucopyranosyl-(1 -> 2)-α-D-mannopyranosyl-(1 -> 6)]-β-D-mannopyranosyl-(1 -> 4)-2-acetamido-2-deoxy-β-D-glucopyranosyl-(1 -> 4)-2-acetamido-2-deoxy-β-D-glucopyranoside (35)***:*

To one of the two portions of the above galactosylation reaction mixture were added Tris-HCl (pH 7.5, 1 M, 2 μL), GDP-Fuc (1.0 equiv.), $MnCl_2$ (100 mM, 4 μL) and FuT-6 (2.8 mg/mL, 1 μL, 2.6‰wt). The mixture was incubated at 37 °C for 18 hrs for completion, which was confirmed by LC-MS. The reaction was quenched with an equal volume of cold MeOH and 5N NaOH (6 μL). The resulting mixture was concentrated under vacuum, loaded onto Sephadex G25 (fine) column, and eluted with dH_2O. Fractions containing the product were lyophilized to give **35** as white fluffy powders.

For the synthesis of **5-aminopentyl β-D-galactopyranosyl-(1 -> 4)-[α-L-fucopyranosyl-(1 -> 3)]-2-acetamido-2-deoxy-β-D-glucopyranosyl-(1 -> 2)-α-D-mannopyranosyl-(1 -> 6)-[3-*O*-sulfo-β-D-galactopyranosyl-(1 -> 4)-2-acetamido-2-deoxy-β-D-glucopyranosyl-(1 -> 2)-α-D-mannopyranosyl-(1 -> 3)]-β-D-mannopyranosyl-(1 -> 4)-2-acetamido-2-deoxy-β-D-glucopyranosyl-(1 -> 4)-2-acetamido-2-deoxy-β-D-glucopyranoside (36)**:

To the other portion of the above galactosylation reaction mixture were added Tris-HCl (pH 7.5, 1 M, 2 μL), GDP-Fuc (1.0 equiv.), and FuT-9 (1.2 ‰wt). The mixture was incubated at 37 °C for 6 hrs for completion, which was confirmed by LC-MS. The reaction was quenched with an equal volume of cold MeOH and 5N NaOH (6 μL). The resulting mixture was concentrated under vacuum, loaded onto Sephadex G25 (fine) column, and eluted with dH_2O. Fractions containing the product were lyophilized to give **36** as white fluffy powders.

35: ^{1}H NMR (600 MHz, D_2O) δ = 5.14 (d, *J* = 4.0 Hz, 1H), 5.12 (s, 1H), 4.94 (s, 1H), 4.85 (q, *J* = 6.7 Hz, 1H), 4.78 (s, 1H), 4.60 (t, *J* = 8.5 Hz, 3H), 4.57 (d, *J* = 7.9 Hz, 1H), 4.50 (d, *J* = 8.8 Hz, 1H), 4.48 (d, *J* = 8.0 Hz, 1H), 4.33 (dd, *J* = 9.9, 3.2 Hz, 1H), 4.28 (d, *J* = 3.3 Hz, 1H), 4.26 (s, 1H), 4.21 – 4.18 (m, 1H), 4.12 (dd, *J* = 3.8, 1.5 Hz, 1H), 4.05 – 3.47 (m, 54H), 2.99 (t, *J* = 7.6 Hz, 2H), 2.09 (s, 3H), 2.06 (s, 3H), 2.05 (s, 3H), 2.04 (s, 3H), 1.68 (quint, *J* = 7.7 Hz, 2H), 1.60 (quint, *J* = 6.5 Hz, 2H), 1.44 – 1.36 (m, 2H),

1.18 (d, J = 6.7 Hz, 3H) ppm. HRMS (ESI-ToF) *m/z* calculated for $C_{73}H_{123}N_5O_{53}S^{2-}$ [M-2H]$^{2-}$: 974.8408, found: 974.8245.

36: ^{1}H NMR (600 MHz, D_2O) δ = 5.14 (d, J = 4.0 Hz, 1H), 5.13 (s, 1H), 4.93 (s, 1H), 4.85 (q, J = 6.7 Hz, 1H), 4.78 (s, 1H), 4.63 – 4.57 (m, 4H), 4.50 (d, J = 8.0 Hz, 1H), 4.46 (d, J = 7.8 Hz, 1H), 4.35 (dd, J = 9.9, 3.2 Hz, 1H), 4.30 (d, J = 3.3 Hz, 1H), 4.27 – 4.25 (m, 1H), 4.20 (dd, J = 3.5, 1.6 Hz, 1H), 4.11 (dd, J = 3.6, 1.6 Hz, 1H), 4.05 – 3.44 (m, 54H), 2.99 (t, J = 7.6 Hz, 2H), 2.09 (s, 3H), 2.06 (s, 3H), 2.05 (s, 3H), 2.04 (s, 3H), 1.68 (quint, J = 7.7 Hz, 2H), 1.60 (quint, J = 6.5 Hz, 2H), 1.45 – 1.35 (m, 2H), 1.19 (d, J = 6.7 Hz, 3H) ppm. HRMS (ESI-ToF) *m/z* calculated for $C_{73}H_{123}N_5O_{53}S^{2-}$ [M-2H]$^{2-}$: 974.8408, found: 974.8291.

3. References

(1) Silva, D. J.; Wang, H.; Allanson, N. M.; Jain, R. K.; Sofia, M. J. Stereospecific Solution- and Solid-Phase Glycosylations. Synthesis of β-Linked Saccharides and Construction of Disaccharide Libraries Using Phenylsulfenyl 2-Deoxy-2-Trifluoroacetamido Glycopyranosides as Glycosyl Donors1. *J. Org. Chem.* **1999**, *64* (16), 5926–5929. https://doi.org/10.1021/jo9903499.

(2) Joseph, A. A.; Verma, V. P.; Liu, X.-Y.; Wu, C.-H.; Dhurandhare, V. M.; Wang, C.-C. TMSOTf-Catalyzed Silylation: Streamlined Regioselective One-Pot Protection and Acetylation of Carbohydrates. *Eur. J. Org. Chem.* **2012**, *2012* (4), 744–753. https://doi.org/10.1002/ejoc.201101267.

(3) Li, R.; Chen, P.; Zeng, Y.-F.; Tseng, T.-H.; Gannedi, V.; Krasnova, L.; Wong, C.-H. Expedient Assembly of Multiantennary *N*-Glycans from Common *N*-Glycan Cores with Orthogonal Protection for the Profiling of Glycan-Binding Proteins. *J. Am. Chem. Soc.* **2025**, *147* (15), 12937–12948. https://doi.org/10.1021/jacs.5c02356.

(4) Ingram, L. J.; Taylor, S. D. Introduction of 2,2,2-Trichloroethyl-Protected Sulfates into Monosaccharides with a Sulfuryl Imidazolium Salt and Application to the Synthesis of Sulfated Carbohydrates. *Angew. Chem. Int. Ed.* **2006**, *45* (21), 3503–3506. https://doi.org/10.1002/anie.200600153.

(5) Ueda, T.; Itoh, R.; Nakamura, T.; Suzuki, K.; Nakane, S.; Yang, Z. Novel Oligosaccharide, Manufacturing Intermediate for Novel Oligosaccharide, and Method for Manufacturing These. EP4424717A1, September 4, 2024. https://patents.google.com/patent/EP4424717A1/en (accessed 2025-12-08).

(6) Luis, A. S.; Jin, C.; Pereira, G. V.; Glowacki, R. W. P.; Gugel, S. R.; Singh, S.; Byrne, D. P.; Pudlo, N. A.; London, J. A.; Baslé, A.; Reihill, M.; Oscarson, S.; Eyers, P. A.; Czjzek, M.; Michel, G.; Barbeyron, T.; Yates, E. A.; Hansson, G. C.; Karlsson, N. G.; Cartmell, A.; Martens, E. C. A Single Sulfatase Is Required to Access Colonic Mucin by a Gut Bacterium. *Nature* **2021**, *598* (7880), 332–337. https://doi.org/10.1038/s41586-021-03967-5.

(7) Boeggeman, E. E.; Balaji, P. V.; Sethi, N.; Masibay, A. S.; Qasba, P. K. Expression of Deletion Constructs of Bovine β-1, 4-Galactosyltransferase in Escherichia Coli: Importance of Cysl34 for Its Activity. *Protein Engineering* **1993**, *6* (7), 779–785. *https://doi.org/10.1093/protein/6.7.779* .

(8) Zhang, J.; Zhu, Y.; Zhang, W.; Mu, W. Efficient Production of a Functional Human Milk Oligosaccharide 3′-Sialyllactose in Genetically Engineered Escherichia Coli. ACS Synthetic Biology 2022. *https://doi.org/10.1021/acssynbio.2c00243*.

4. NMR assignment of key glycans

Assignment of Glycan **25**

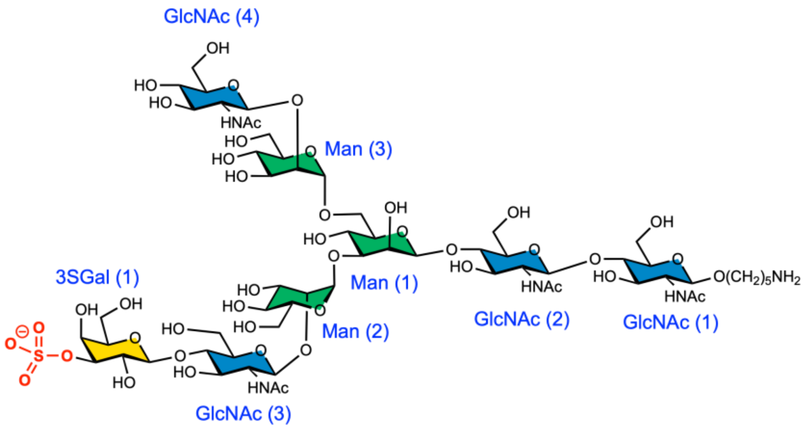

<table>
<tr><th></th><th>H1</th><th>H2</th><th>H3</th><th>H4</th><th>H5</th><th>H6</th><th>NHAc</th></tr>
<tr><td>GlcNAc(1)</td><td>4.46 (d, J=7.4)</td><td>3.67</td><td rowspan="4">3.63-3.75</td><td>3.58</td><td></td><td></td><td rowspan="4">2.00, 2.01, 2.03, 2.06</td></tr>
<tr><td>GlcNAc(2)</td><td>4.57 (d)</td><td>3.74</td><td>3.71</td><td></td><td></td></tr>
<tr><td>GlcNAc(3)</td><td rowspan="2">4.53-4.56</td><td rowspan="2">3.65-3.73</td><td>3.73</td><td></td><td></td></tr>
<tr><td>GlcNAc(4)</td><td></td><td></td><td></td></tr>
<tr><td>Man(1)</td><td>4.73 (s)</td><td>4.22 (d, J=3.0)</td><td>3.76</td><td>3.59</td><td></td><td></td></tr>
<tr><td>Man(2)</td><td>5.08 (s)</td><td>4.17 (d, J=2.7)</td><td>3.88</td><td>3.46-3.47</td><td>3.71</td><td></td></tr>
<tr><td>Man(3)</td><td>4.91 (s)</td><td>4.09 (d, J=3.0)</td><td>3.85</td><td>3.46-3.47</td><td>3.58</td><td></td></tr>
<tr><td>Gal(1)</td><td>4.56 (d)</td><td>3.65</td><td>4.31 (dd, J=9.9, 3.2)</td><td>4.27 (d, J=3.3)</td><td>3.75</td><td></td></tr>
</table>

<table>
<tr><th></th><th>C1(1JCH)</th><th>C2</th><th>C3</th><th>C4</th><th>C5</th><th>C6</th><th>NHAc</th></tr>
<tr><td>GlcNAc(1)</td><td>101.0 (162.4)</td><td>54.8</td><td>72.3</td><td>79.2</td><td>74.3</td><td></td><td rowspan="2">22.1, 22.2, 22.3, 22.3</td></tr>
<tr><td>GlcNAc(2)</td><td>101.3 (163.2)</td><td>54.9</td><td>71.9</td><td>79.3</td><td></td><td></td></tr>
</table>

**GlcNAc(3)**	99.5 (163.2)	54.9	71.9	78.2			
**GlcNAc(4)**	99.5 (163.2)	54.9	71.9				
**Man(1)**	100.3 (155.8)	70.1	80.4	65.5		65.6	
**Man(2)**	99.5 (170.3)	76.4	69.3	67.2	73.5	61.7	
**Man(3)**	97.0 (172.4)	76.3	69.4	67.3	72.8	61.6	
**Gal(1)**	102.4 (164.0)	69.0	79.9	66.7	74.9	60.9	

Assignment of Glycan **36**

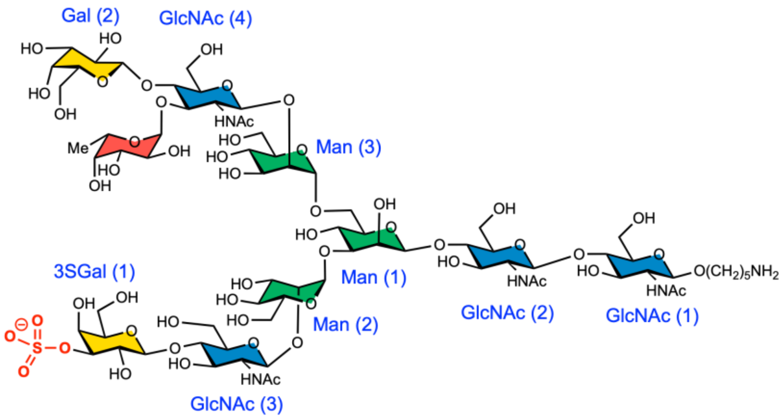

<table>
<tr><th>FUT9P</th><th>H1</th><th>H2</th><th>H3</th><th>H4</th><th>H5</th><th>H6</th><th>NHAc</th></tr>
<tr><td>GlcNAc(1)</td><td>4.46 (d, J=7.7)</td><td>3.68</td><td rowspan="3">3.66-3.75</td><td>3.57</td><td></td><td></td><td rowspan="4">2.00, 2.02, 2.03, 2.06</td></tr>
<tr><td>GlcNAc(2)</td><td>4.57 (d)</td><td>3.77</td><td>3.71</td><td></td><td></td></tr>
<tr><td>GlcNAc(3)</td><td rowspan="2">4.53-4.56</td><td>3.68-3.76</td><td>3.66</td><td></td><td></td></tr>
<tr><td>GlcNAc(4)</td><td>3.90</td><td>3.82</td><td>3.93</td><td></td><td></td></tr>
<tr><td>Man(1)</td><td>4.75 (s)</td><td>4.23 (d, J=3.0)</td><td>3.75</td><td></td><td></td><td>3.78, 3.93</td><td></td></tr>
<tr><td>Man(2)</td><td>5.09 (s)</td><td>4.17 (d, J=3.4)</td><td>3.87</td><td>3.47</td><td>3.72</td><td rowspan="2">3.59, 3.90</td><td></td></tr>
<tr><td>Man(3)</td><td>4.89 (s)</td><td>4.08 (d, J=3.6)</td><td>3.88</td><td>3.45</td><td></td><td></td></tr>
<tr><td>Gal(1)</td><td>4.56 (d)</td><td>3.66</td><td>4.31 (dd, J=9.9, 3.2)</td><td>4.27 (d, J=3.3)</td><td>3.74</td><td></td><td></td></tr>
<tr><td>Gal(2)</td><td>4.42 (d, J=7.8)</td><td>3.47</td><td>3.63</td><td>3.87</td><td></td><td></td><td></td></tr>
<tr><td>Fuc1</td><td>5.11 (d, J=4.0)</td><td>3.67</td><td>3.89</td><td>3.77</td><td>4.82</td><td>1.15 (d, J=6.6)</td><td></td></tr>
</table>

	**C1 (1JCH)**	**C2**	**C3**	**C4**	**C5**	**C6**	**NHAc**
**GlcNAc(1)**	101.0 (161.4)	54.9	72.4	79.2			22.1, 22.2, 22.3, 22.3

**GlcNAc(2)**	101.3 (162.4)	54.8	71.9	79.6			
**GlcNAc(3)**	99.4 (162.4)	54.8	71.9	78.3			
**GlcNAc(4)**	99.1 (162.4)	55.6	74.4	73.2			
**Man(1)**	100.4 (158.4)	70.1	80.3	65.7		65.9	
**Man(2)**	99.5 (170.7)	76.3	69.3	67.2	73.5	61.7	
**Man(3)**	96.8 (170.3)	76.0	69.3	67.3	72.8	61.6	
**Gal(1)**	102.4 (162.4)	69.3	79.8	66.74	74.9	60.9	
**Gal(2)**	101.7 (161.4)	71.0	72.3		73.2	61.4	
**Fuc1**	98.5 (172.5)	67.6			66.66	15.3	

Assignment of Glycan **35**

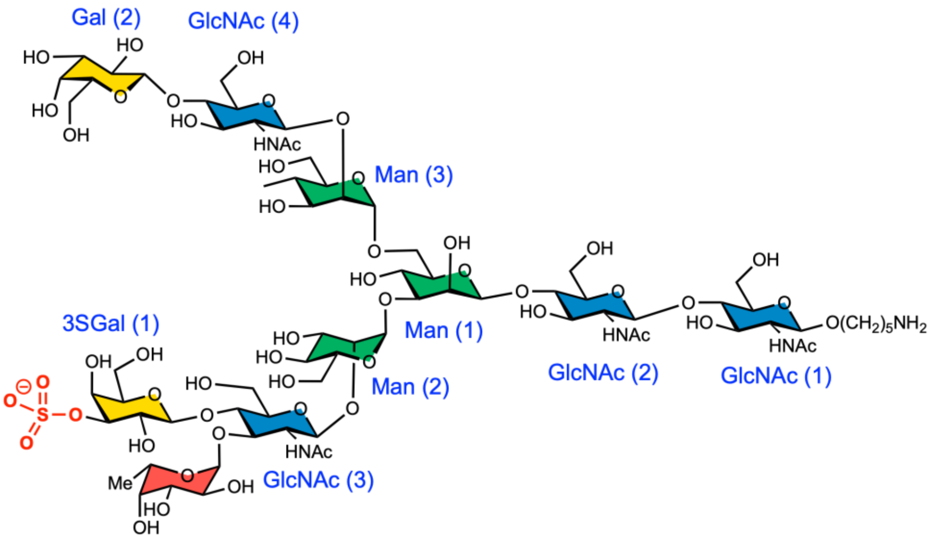

<table>
<tr><th>FUT6P</th><th>H1</th><th>H2</th><th>H3</th><th>H4</th><th>H5</th><th>H6</th><th>NHAc</th></tr>
<tr><td>GlcNAc(1)</td><td>4.50 (d, J=8.6)</td><td>3.71</td><td rowspan="2">3.69-3.78</td><td>3.60</td><td></td><td></td><td rowspan="4">2.04, 2.05, 2.06, 2.09</td></tr>
<tr><td>GlcNAc(2)</td><td>4.61 (d)</td><td>3.81</td><td>3.75</td><td></td><td></td></tr>
<tr><td>GlcNAc(3)</td><td rowspan="2">4.58-4.60</td><td>3.92</td><td>3.84</td><td>3.99</td><td></td><td></td></tr>
<tr><td>GlcNAc(4)</td><td>3.76</td><td>3.69-3.78</td><td>3.75</td><td></td><td></td></tr>
<tr><td>Man(1)</td><td>4.78 (s)</td><td>4.26</td><td>3.78</td><td></td><td></td><td>3.81, 3.95</td><td></td></tr>
<tr><td>Man(2)</td><td>5.12 (s)</td><td>4.20</td><td>3.92</td><td>3.50</td><td>3.75</td><td rowspan="2">3.62, 3.92</td><td></td></tr>
<tr><td>Man(3)</td><td>4.94 (s)</td><td>4.12</td><td>3.90</td><td>3.49</td><td></td><td></td></tr>
<tr><td>Gal(1)</td><td>4.57 (d, J=7.8)</td><td>3.64</td><td>4.33 (dd, J=9.9, 3.2)</td><td>4.28 (d, J=3.3)</td><td></td><td></td><td></td></tr>
<tr><td>Gal(2)</td><td>4.48 (d, J=8.0)</td><td>3.55</td><td></td><td></td><td></td><td></td><td></td></tr>
<tr><td>Fuc1</td><td>5.14 (d, J=4.0)</td><td>3.69</td><td>3.92</td><td>3.80</td><td>4.81</td><td>1.18 (d, J=6.6)</td><td></td></tr>
</table>

<table>
<tr><th></th><th>C1 (1JCH)</th><th>C2</th><th>C3</th><th>C4</th><th>C5</th><th>C6</th><th>NHAc</th></tr>
<tr><td>GlcNAc(1)</td><td>101 (161.5)</td><td>54.8</td><td>72.4</td><td>79.2</td><td></td><td></td><td></td></tr>
</table>

**GlcNAc(2)**	101.3 (162.3)	54.8	71.9	79.4			22.1, 22.2, 22.3, 22.4
**GlcNAc(3)**	99.1 (162.0)	55.6	74.3	73.2			
**GlcNAc(4)**	99.3 (162.0)	54.8	72.0	78.4			
**Man(1)**	100.4 (159.9)	70.1	80.4			65.8	
**Man(2)**	99.5 (171.0)	76.22	69.4	67.2	73.5	61.6, 61.7	
**Man(3)**	97.0 (169.0)	76.15	69.3	67.3			
**Gal(1)**	101.3 (161.3)	69.1	80.1	66.6			
**Gal(2)**	102.9 (161.6)	70.9	72.3				
**Fuc1**	98.5 (172.9)	67.7			66.6	15.2	

5. NMR spectra of Compounds

S13 *^{1}H NMR spectrum*

500 MHz, $CDCl_3$

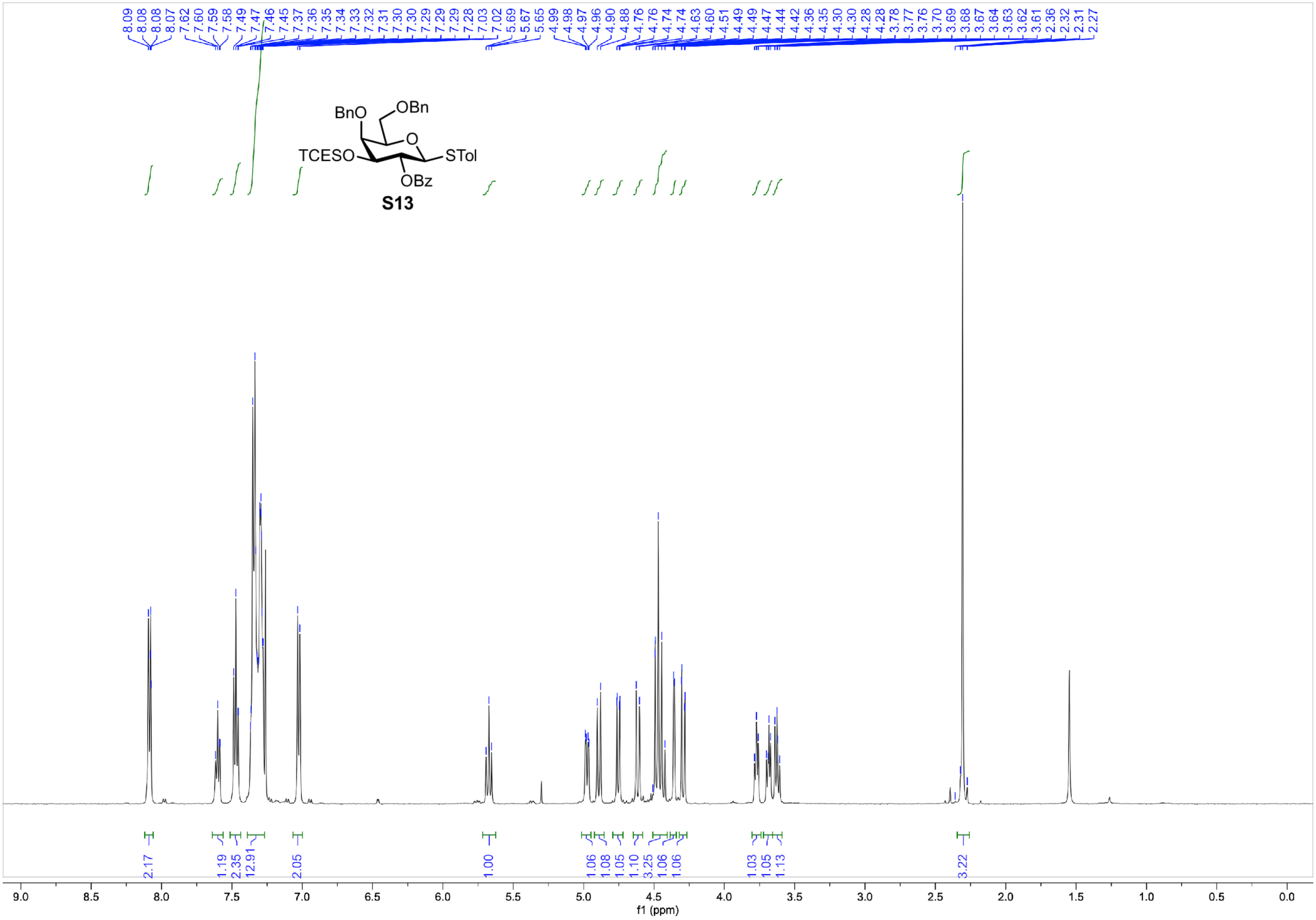

S13 *DEPTQ135* ^{13}C *NMR spectrum*

126 MHz in $CDCl_3$, Pulse Sequence: deptqgpsp, NS 128

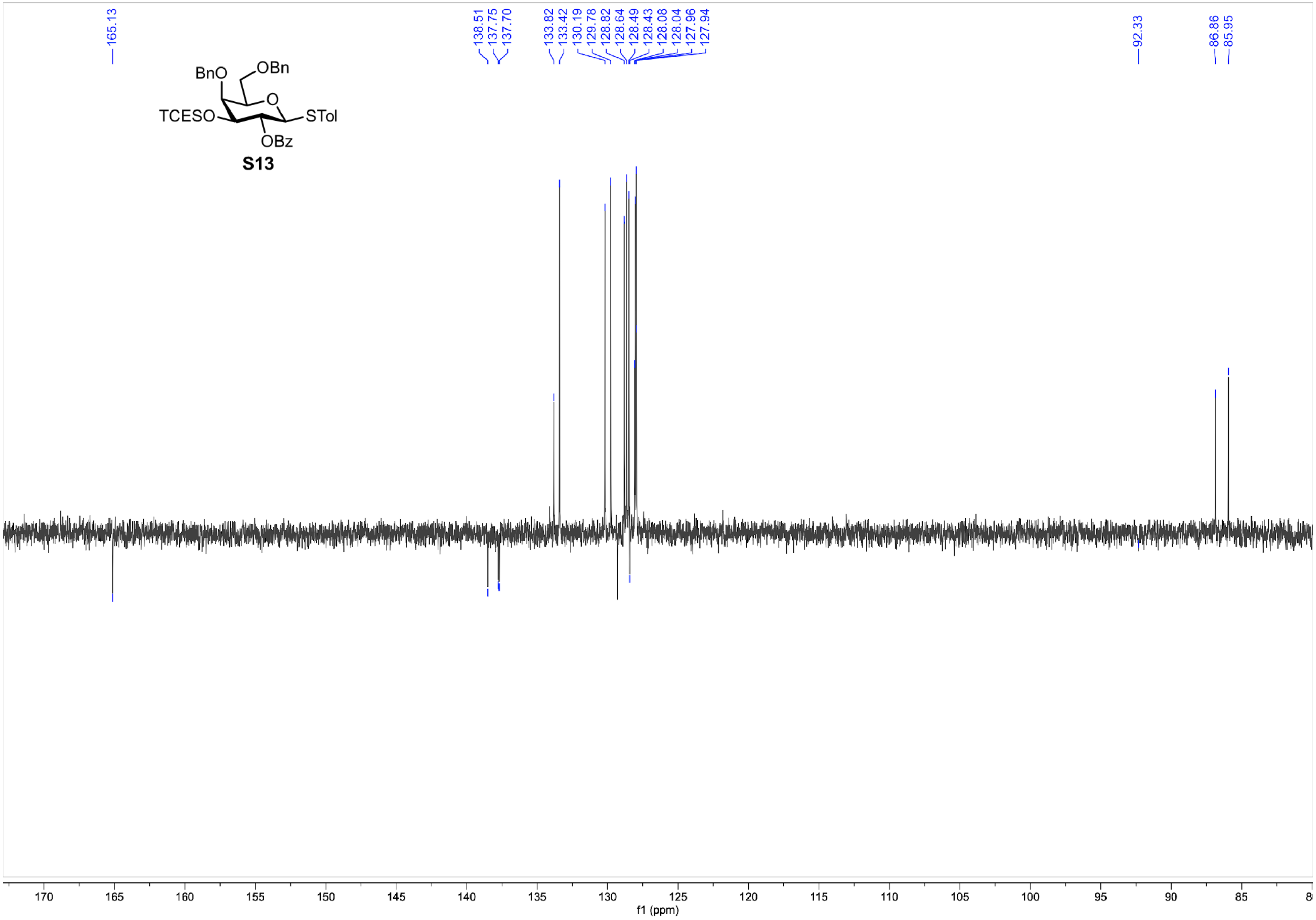

S13 *HSQC spectrum*

500 MHz for ^{1}H in $CDCl_3$, Pulse Sequence: hsqcedetgpsisp2.3, NS 8, NUS 25%

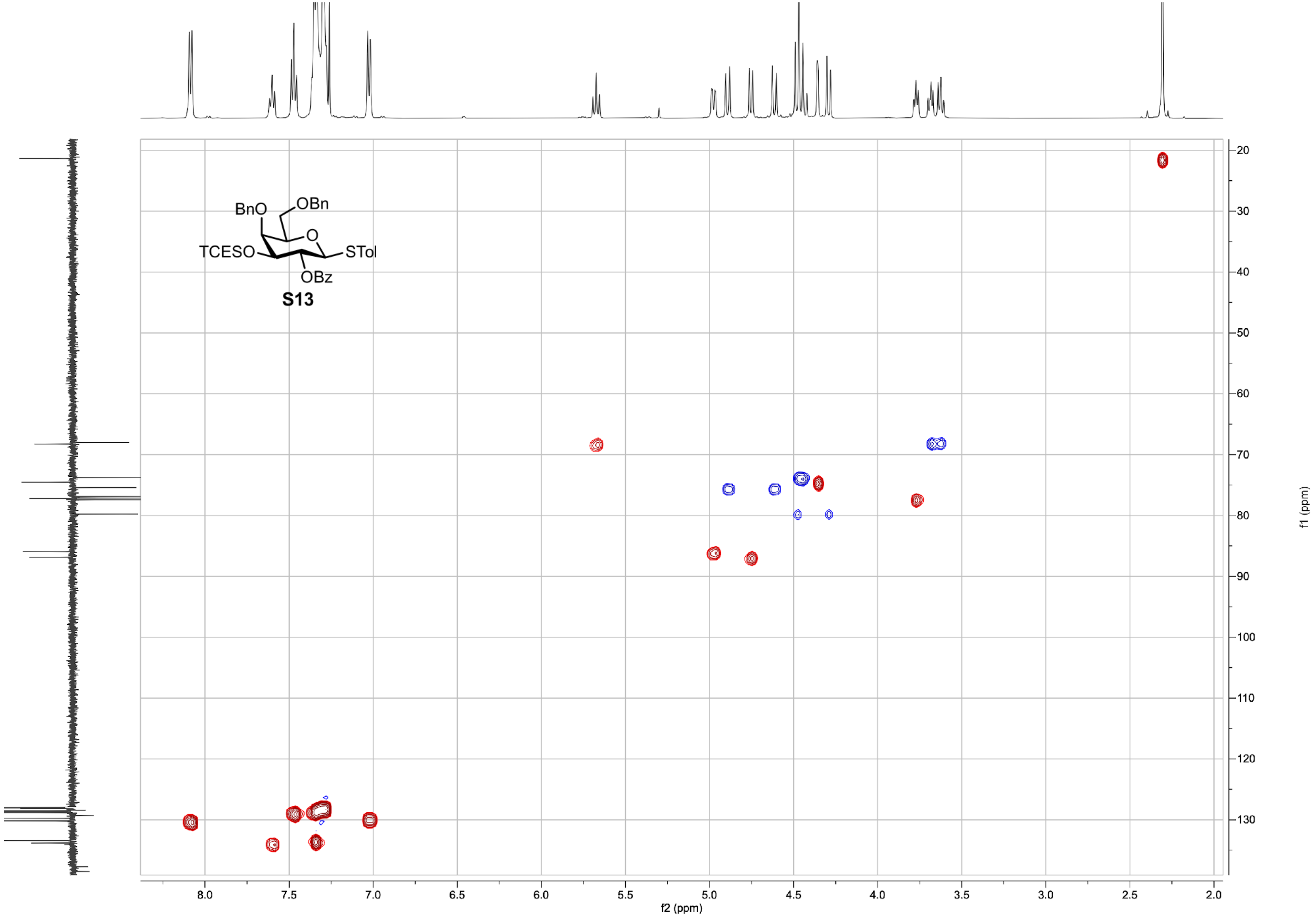

5 ^{1}H NMR spectrum

600 MHz in $CDCl_3$

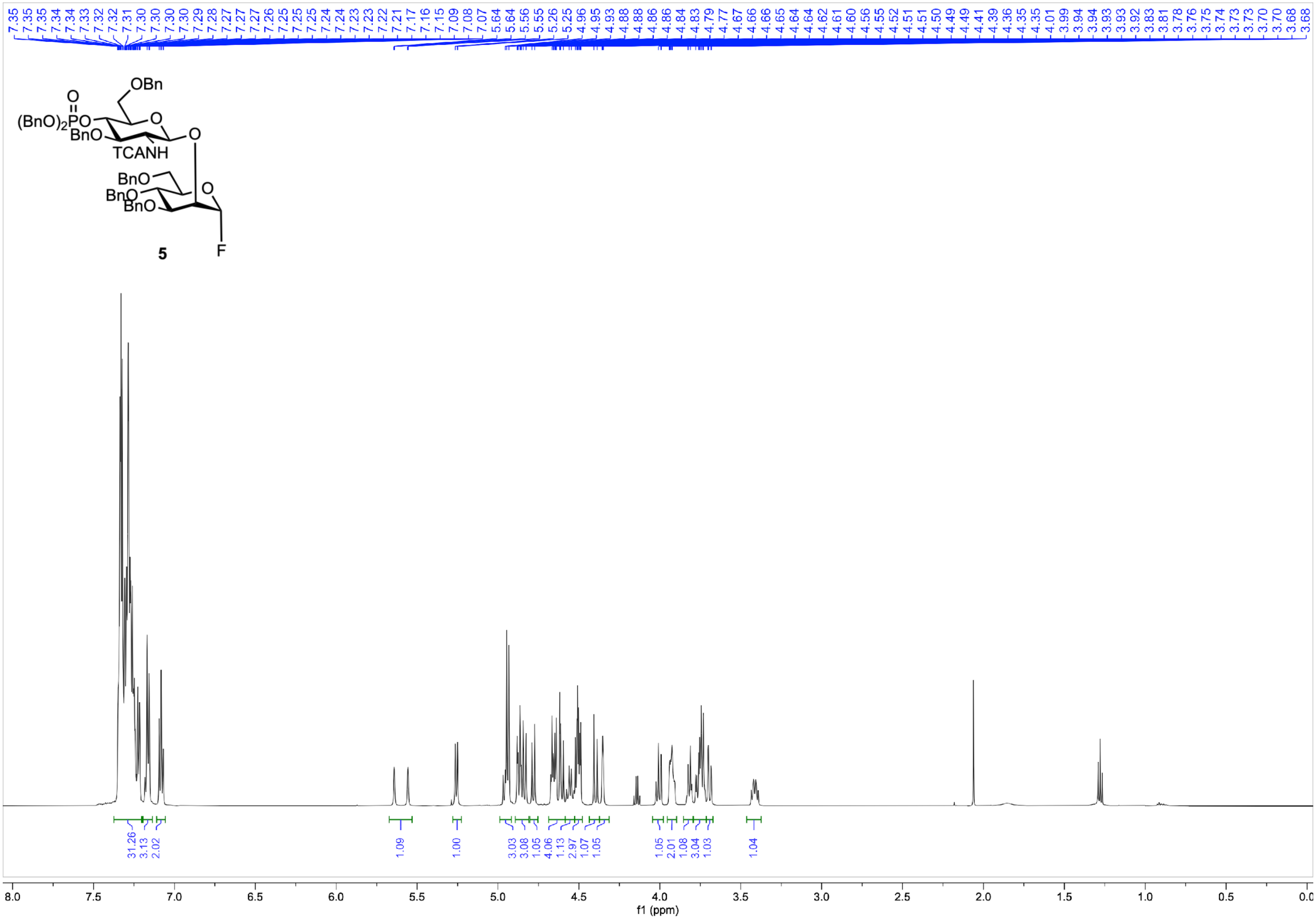

__5__ DEPTQ135 ^{13}C NMR spectrum

151 MHz in $CDCl_3$, Pulse Sequence: deptqgpsp.2, NS 128

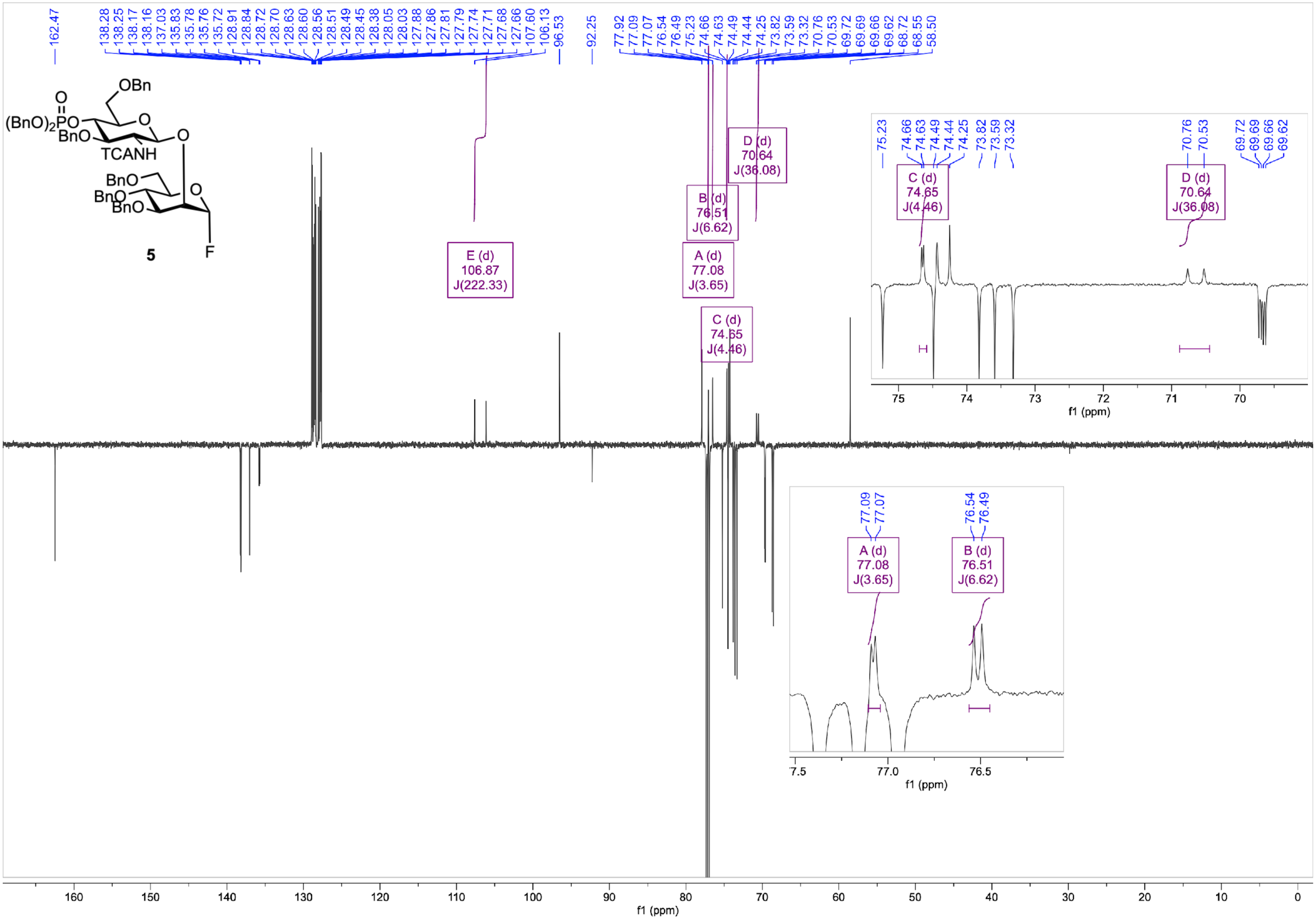

***5** HSQC spectrum*

600 MHz for ^{1}H in $CDCl_3$, Pulse Sequence: hsqcedetgpsisp2.3, NS 2, NUS 25%

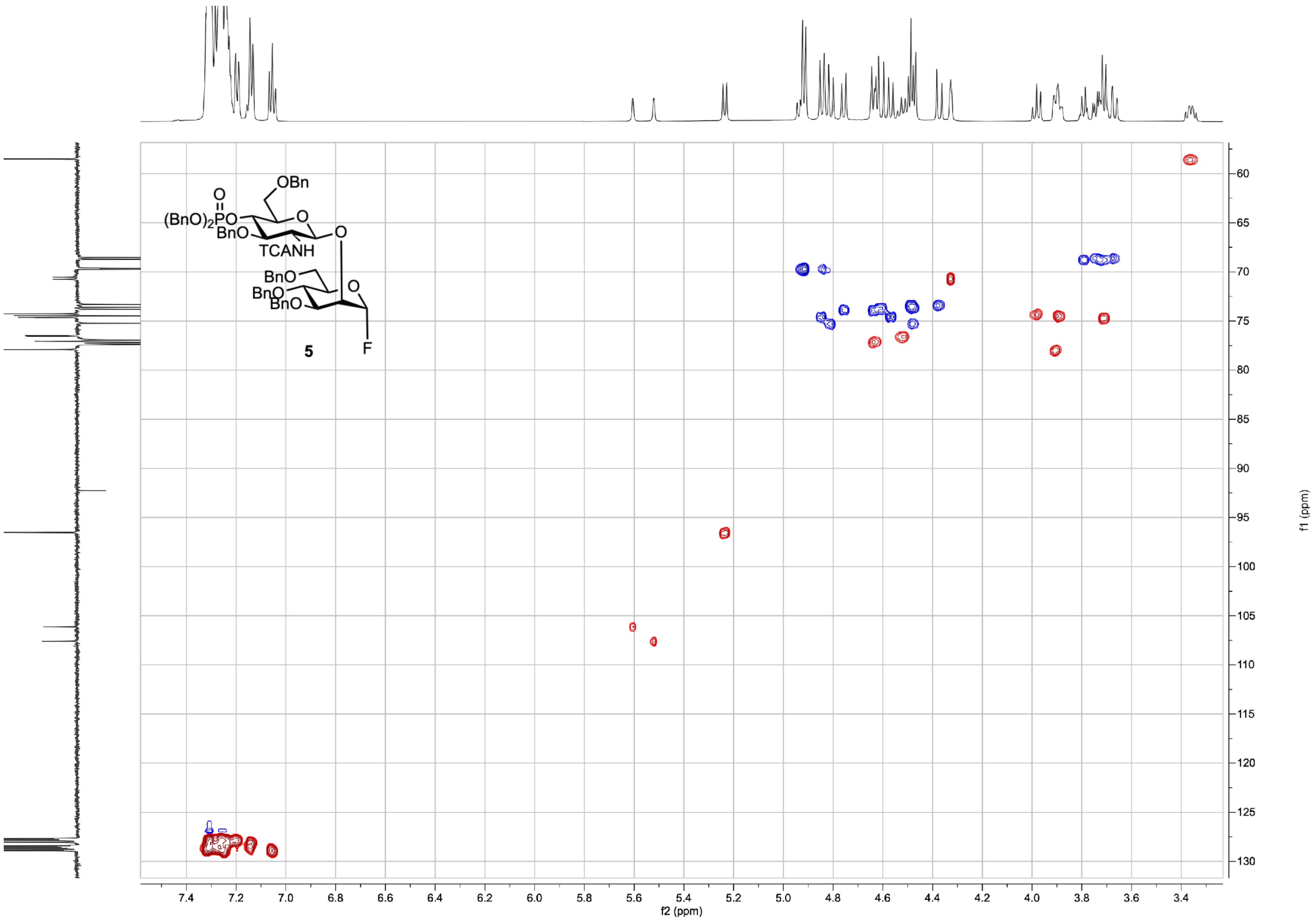

***5** HSQC spectrum with splitting in F2 phase*

600 MHz for ^{1}H in $CDCl_3$, Pulse Sequence: hsqcedetgpsisp2.3, NS 2, NUS 25%

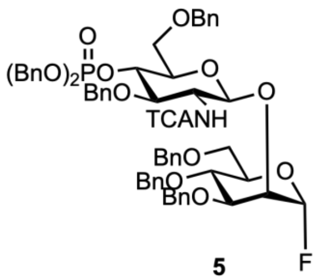

5

5 *^{19}F NMR spectrum*

376 MHz in $CDCl_3$

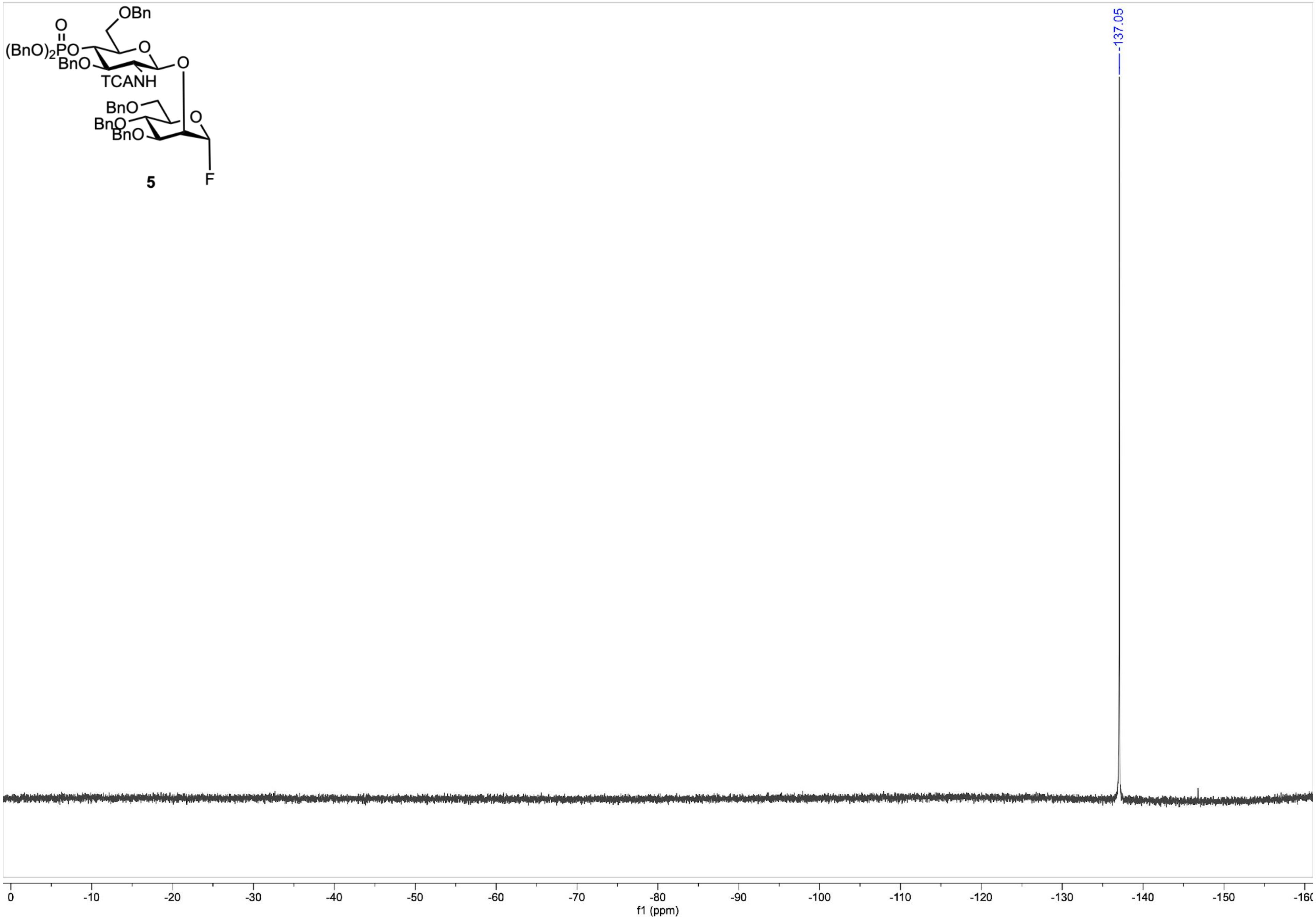

5 *^{31}P NMR spectrum*

162 MHz in $CDCl_3$

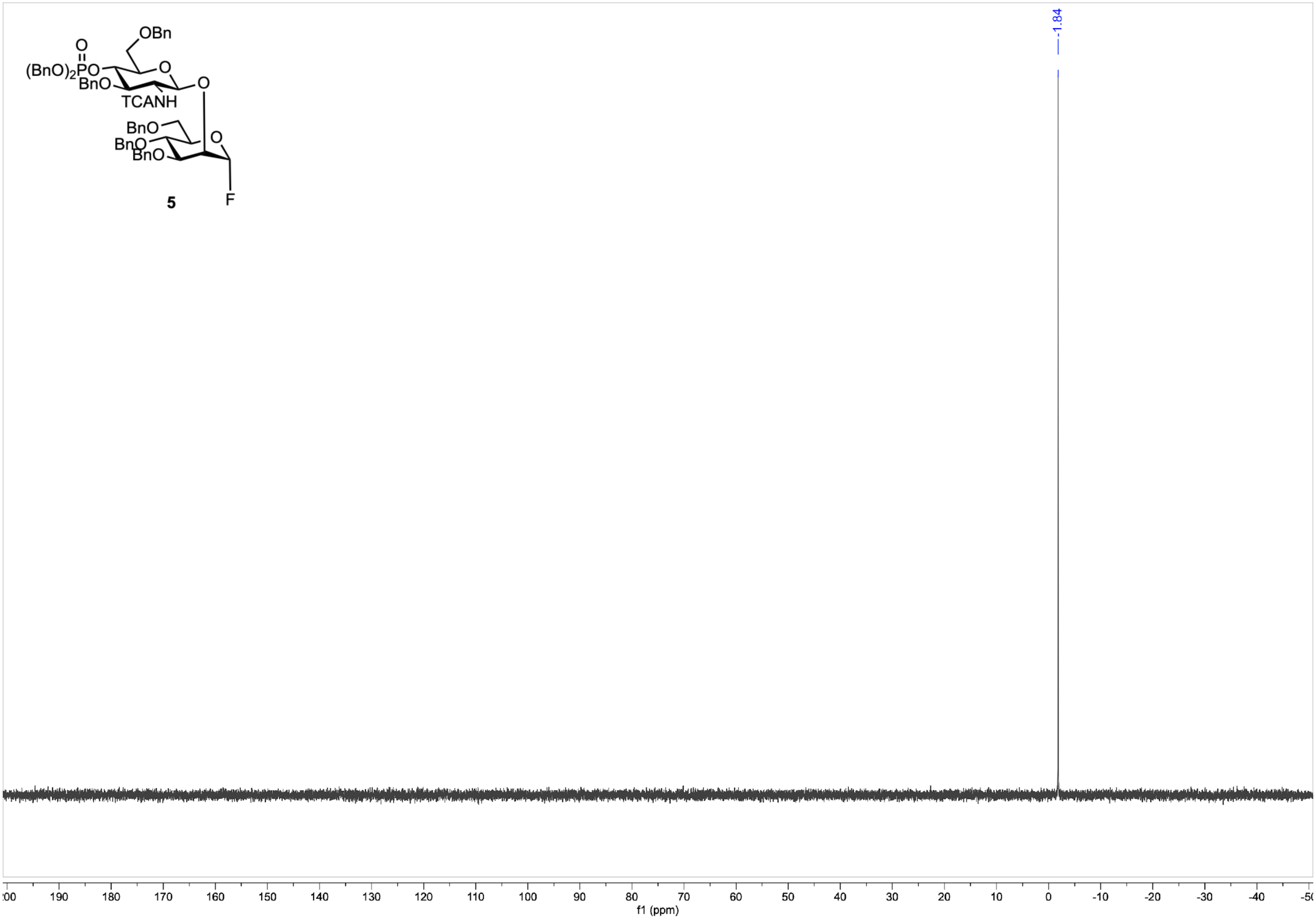

6 ¹H spectrum

600 MHz in $CDCl_3$

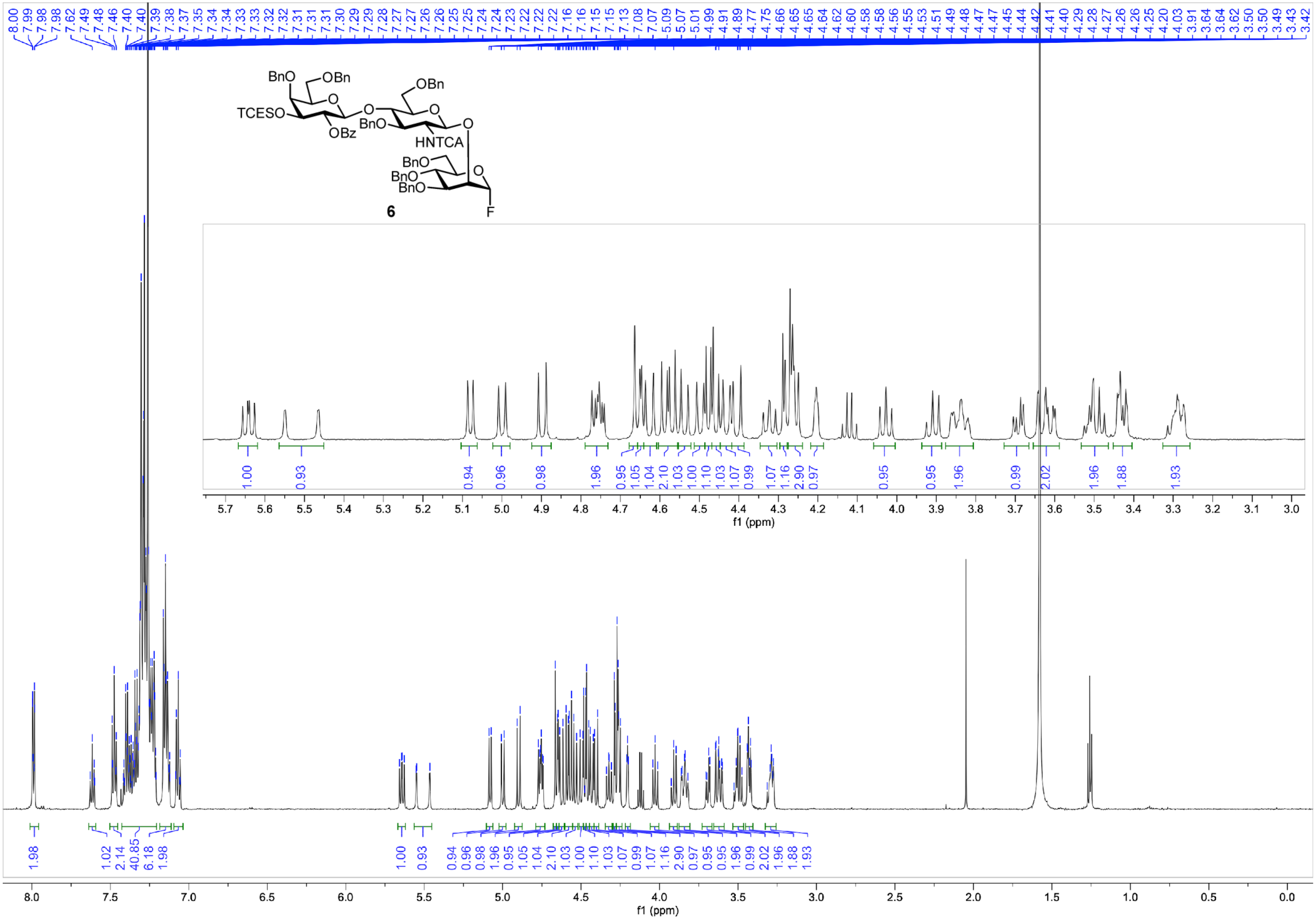

6 DEPTQ135 ^{13}C NMR spectrum

151 MHz in $CDCl_3$, Pulse Sequence: deptqgpsp.2, NS 122

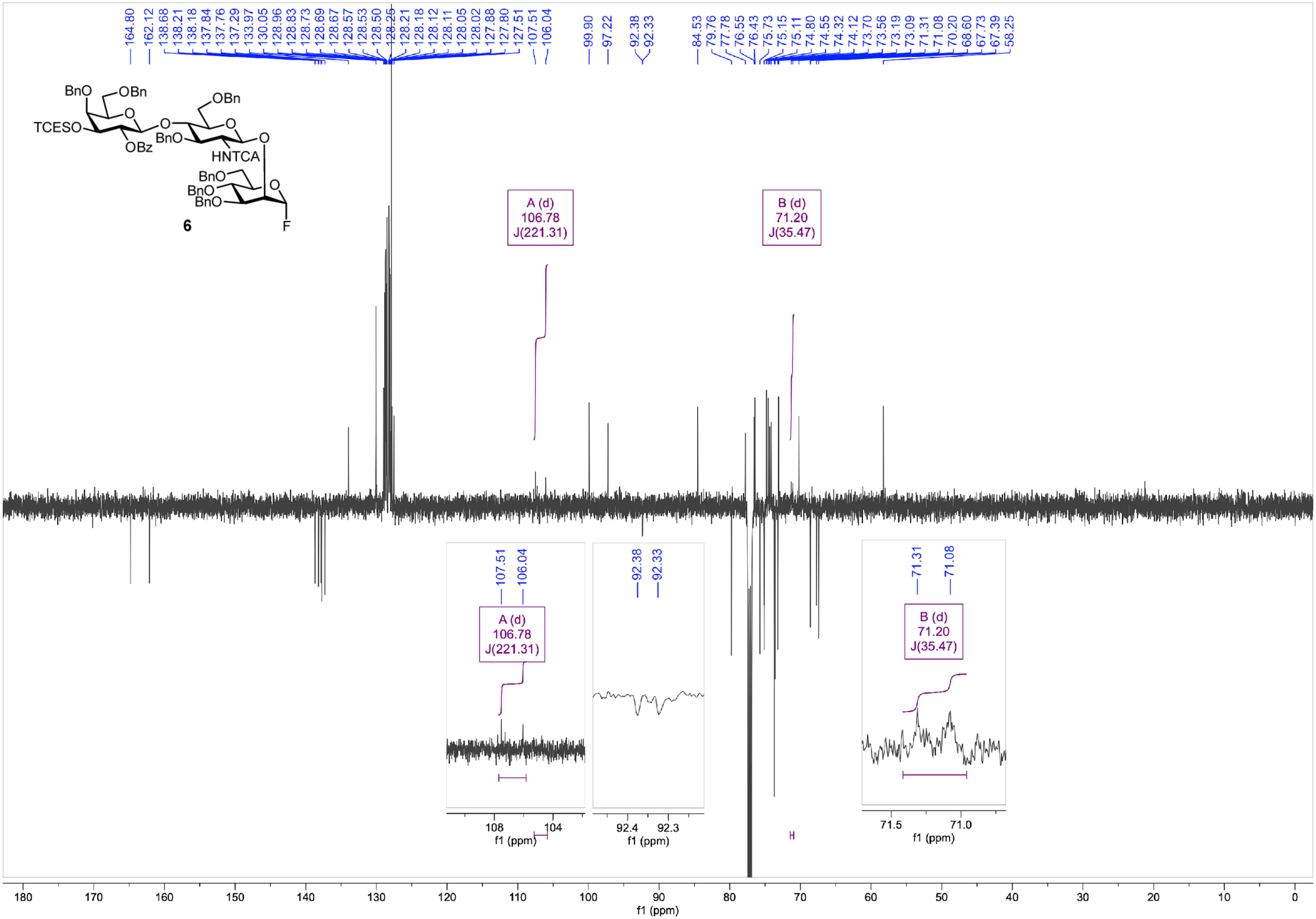

8a *^{1}H NMR spectrum*

600 MHz, $CDCl_3$

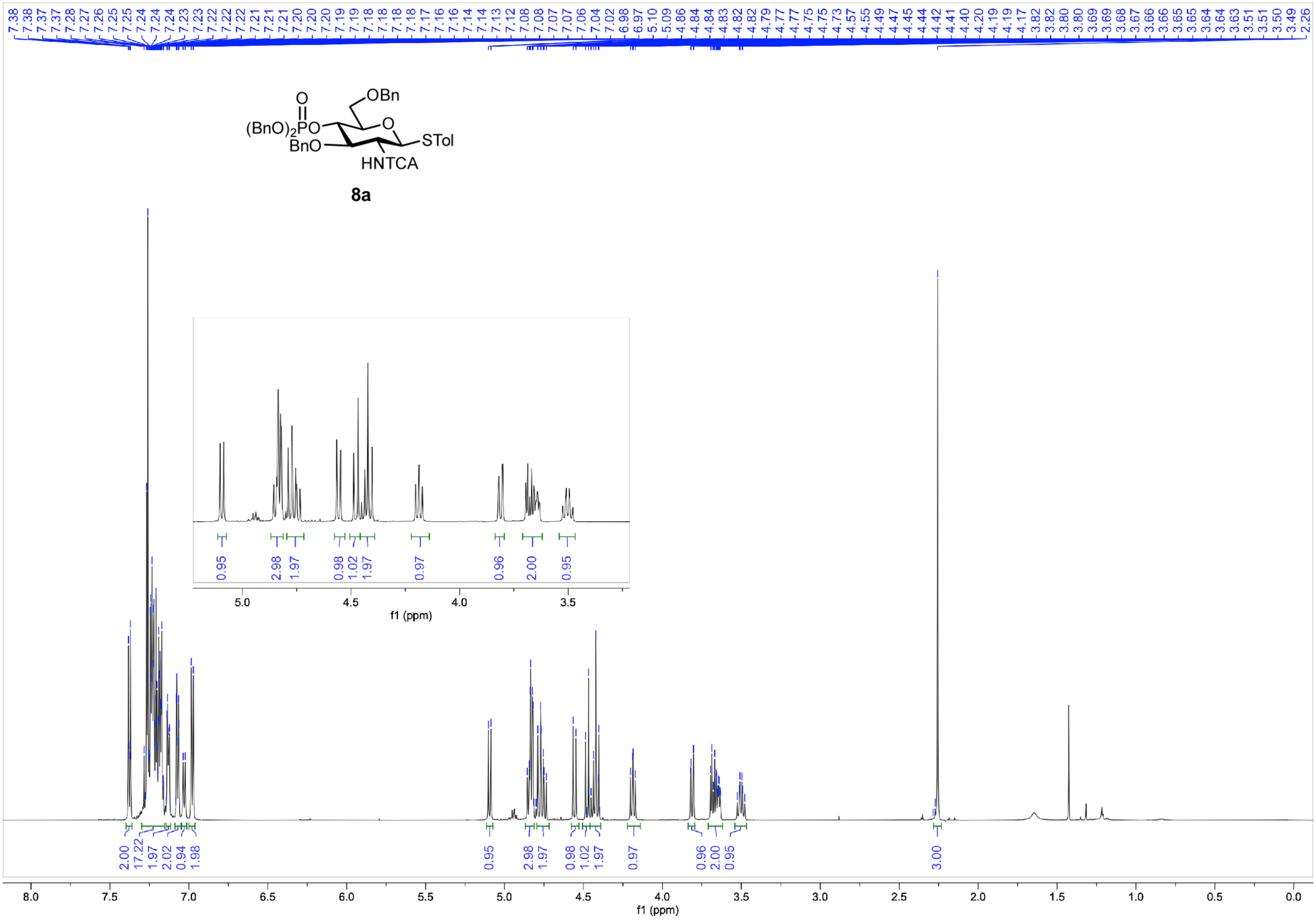

***8a** DEPTQ135 ^{13}C NMR spectrum*

151 MHz in $CDCl_3$, Pulse Sequence: deptqgpsp.2, NS 33

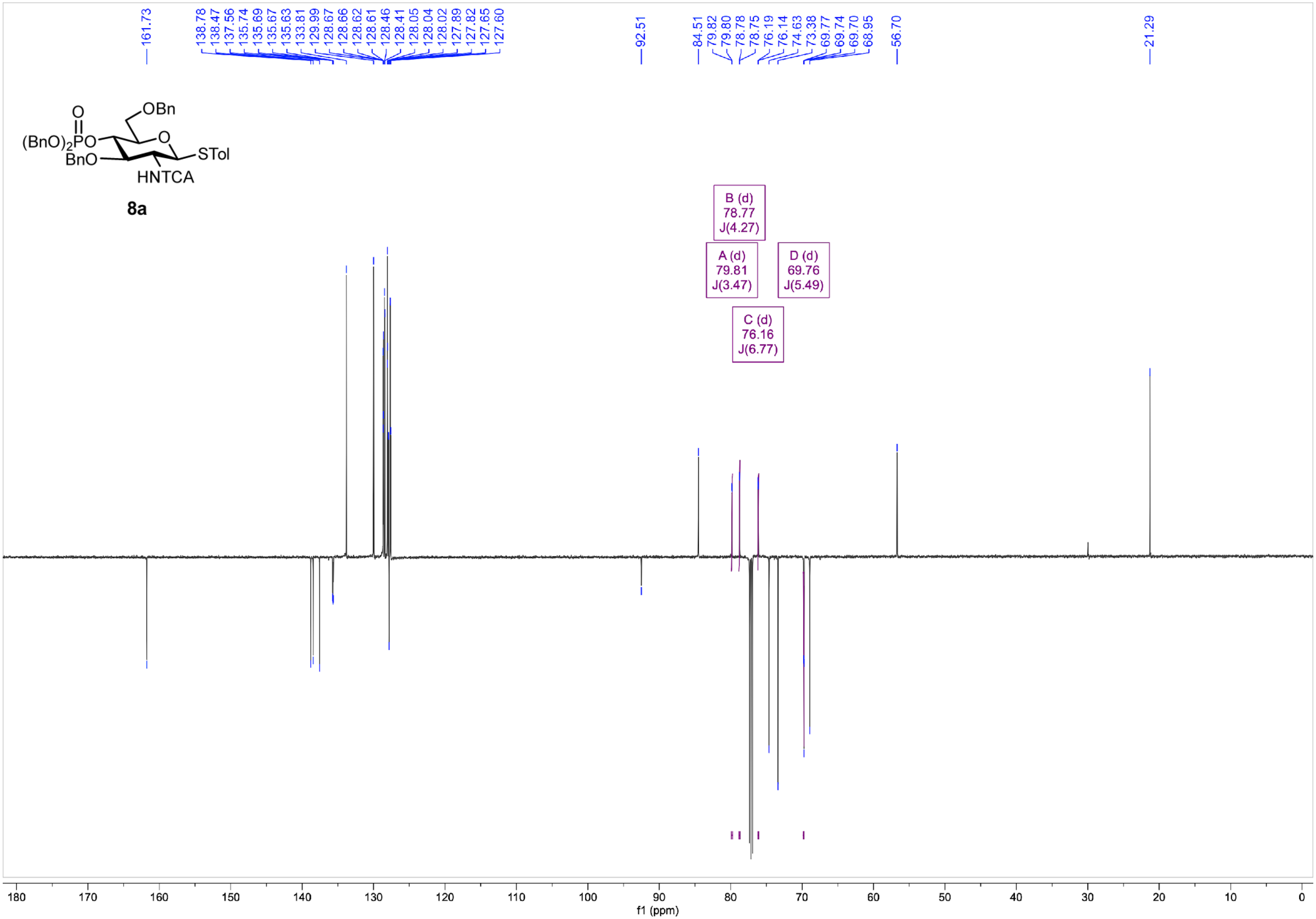

***8a** HSQC spectrum*

600 MHz for ^{1}H in $CDCl_3$, Pulse Sequence: hsqcedetgpsisp2.3, NS 4, NUS 25%

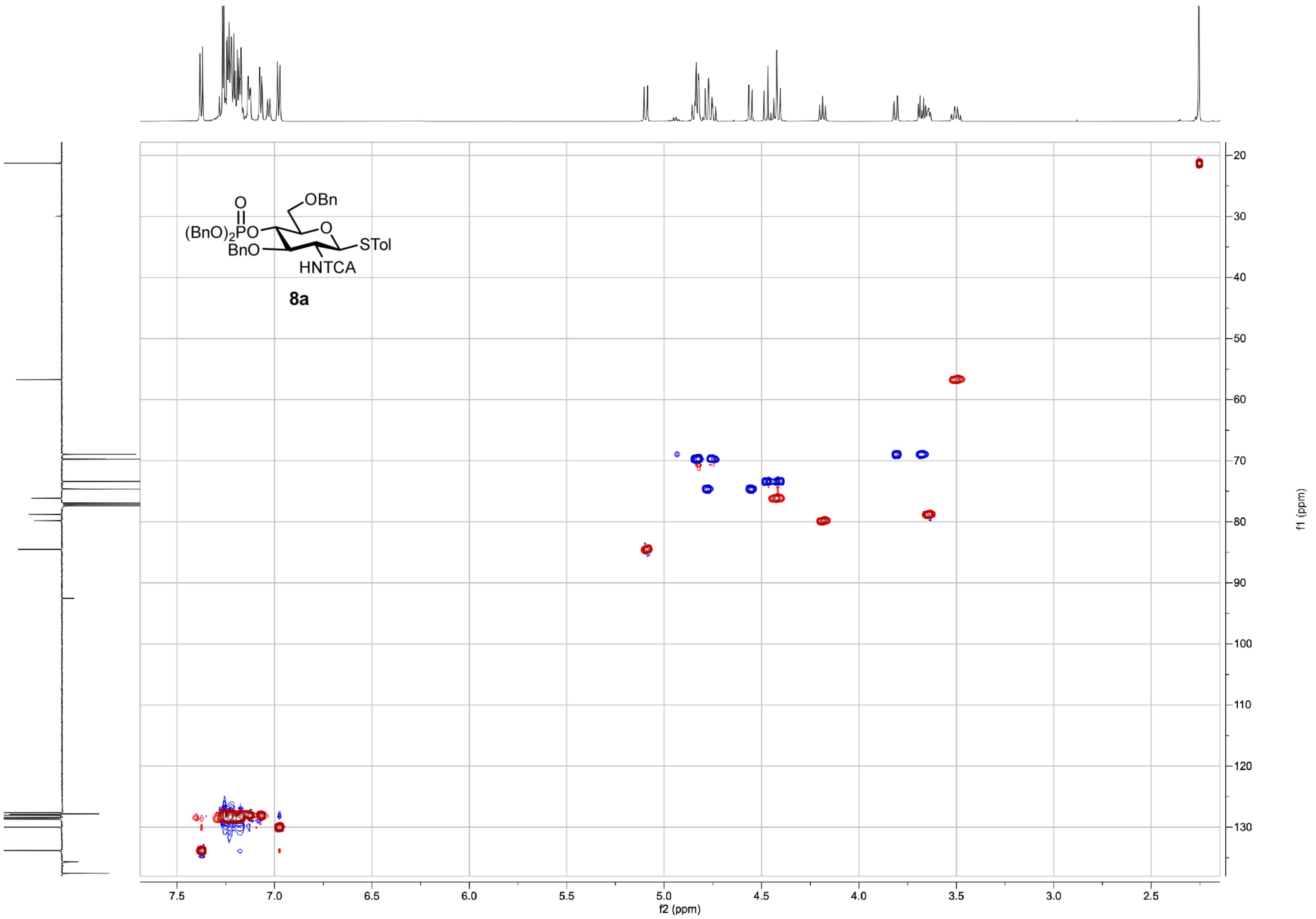

10 *^{1}H NMR spectrum*

600 MHz in $CDCl_3$

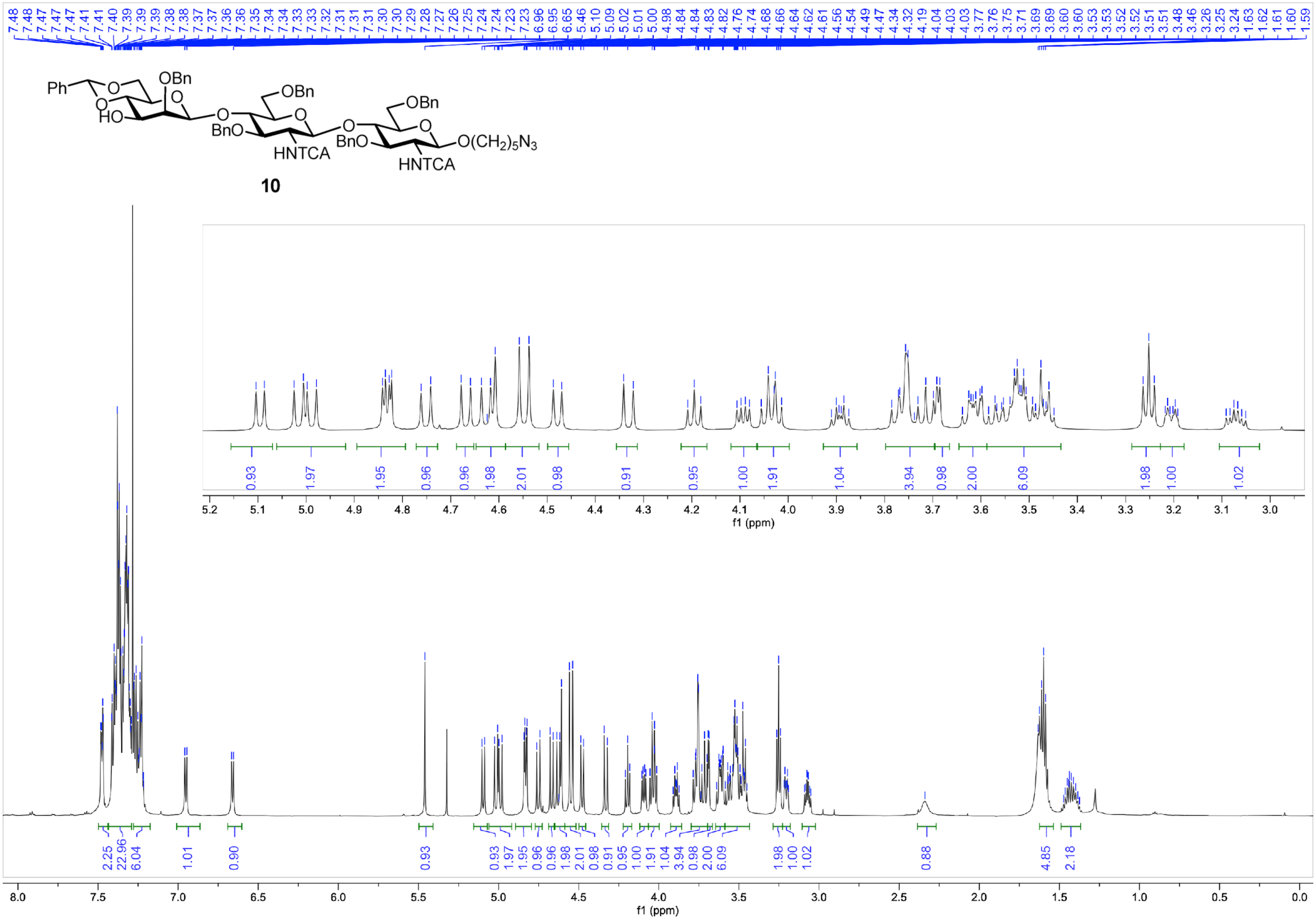

__10__ DEPTQ 135 ^{13}C NMR spectrum

151 MHz in $CDCl_3$, Pulse Sequence: deptqgpsp.2, NS 54

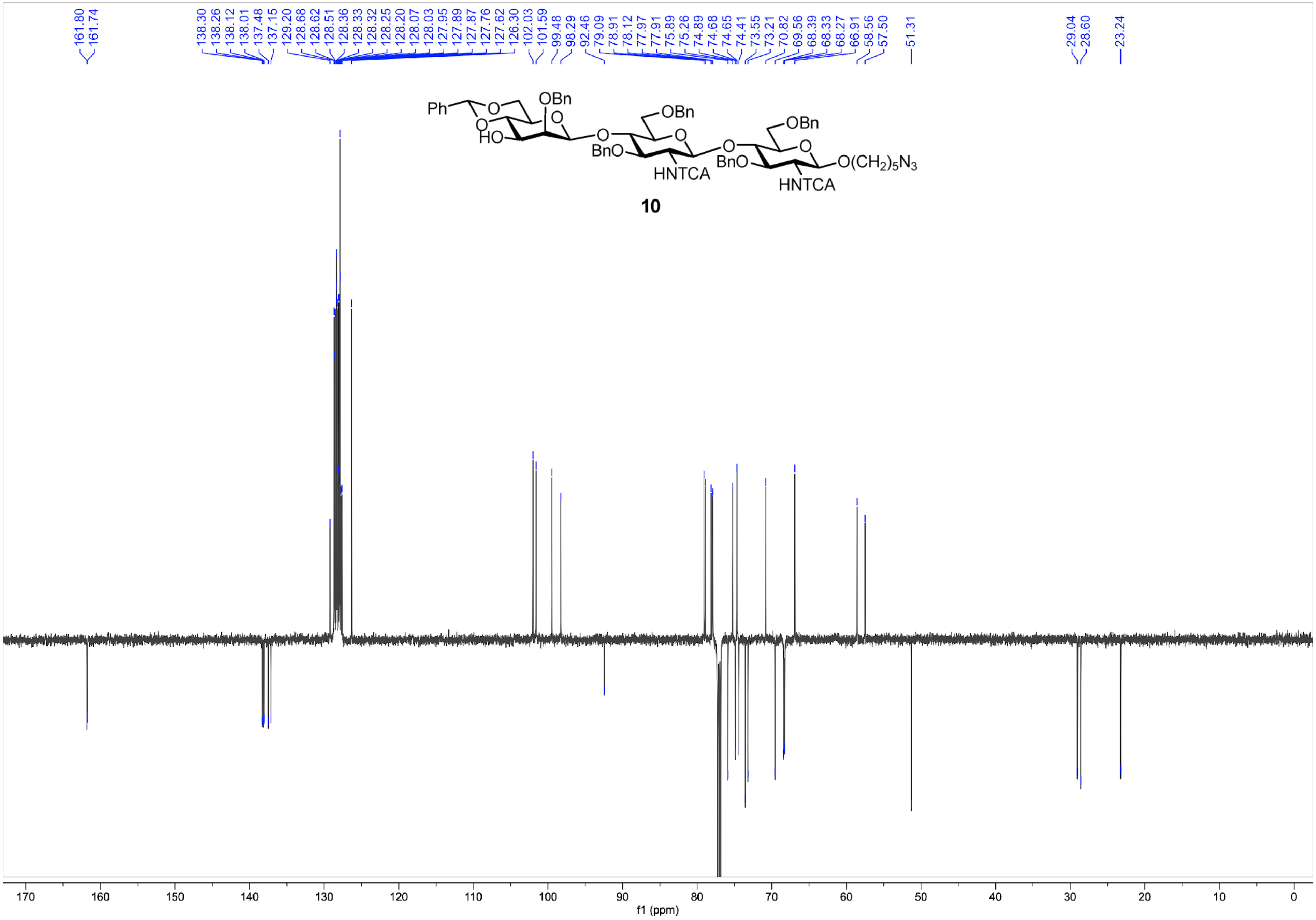

***10** HSQC spectrum*

600 MHz for ^{1}H in CDCl3, Pulse Sequence: hsqcedetgpsisp2.3, NS 2, NUS 25%

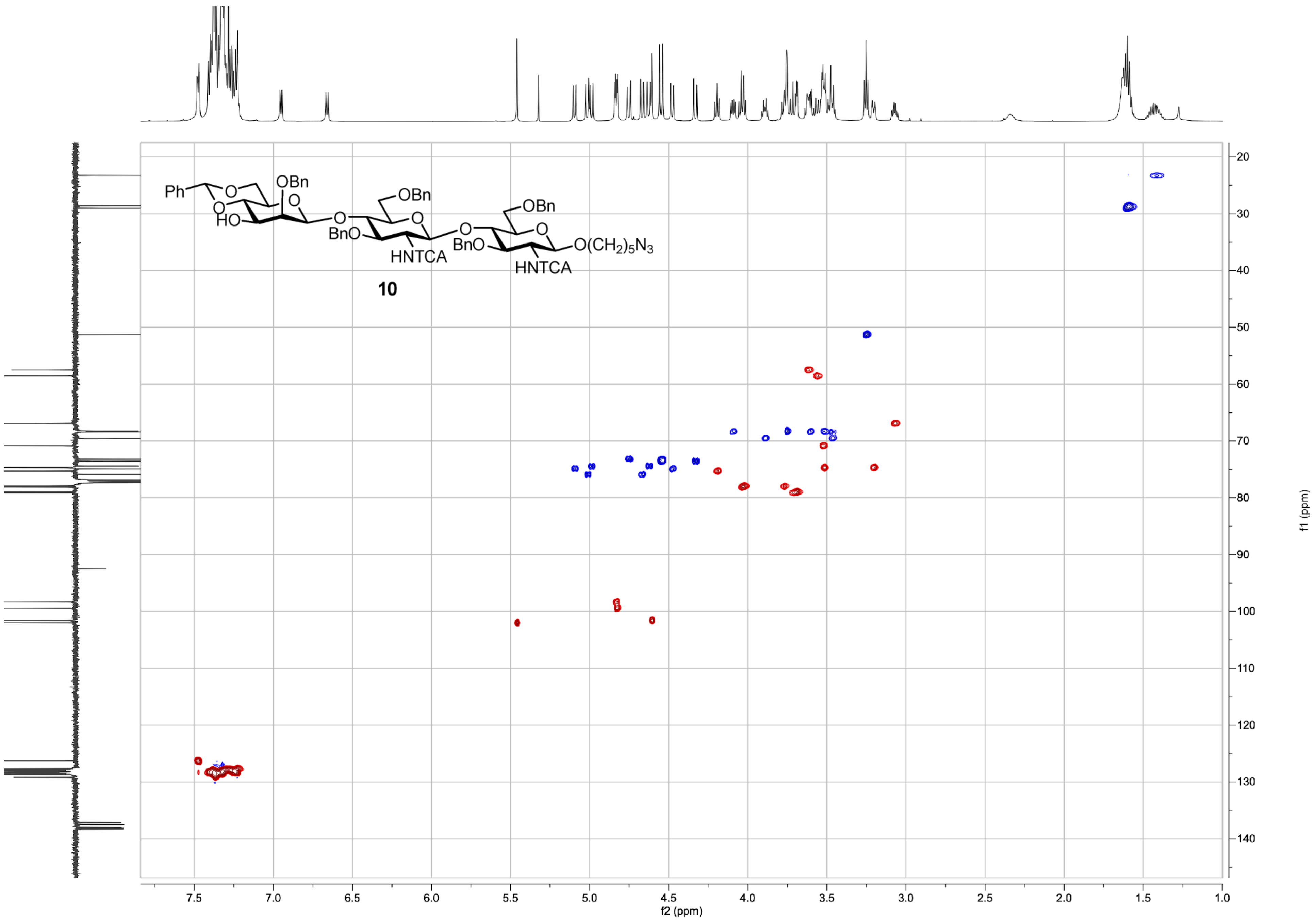

11 *¹H NMR spectrum*

600 MHz in CDCl₃

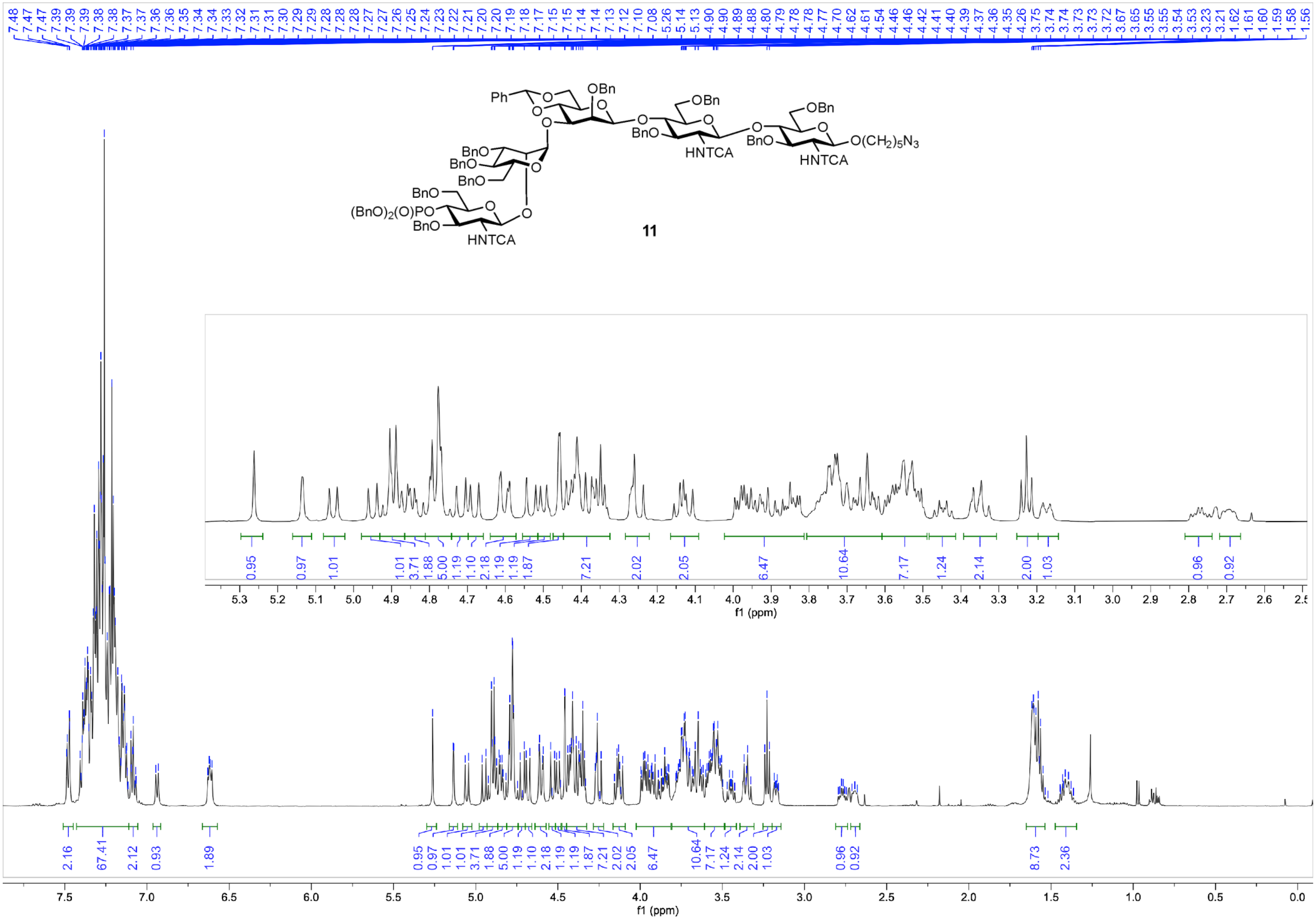

11 *DEPTQ135* ^{13}C *NMR spectrum*

Pulse Sequence: deptqgpsp.2 , NS 128

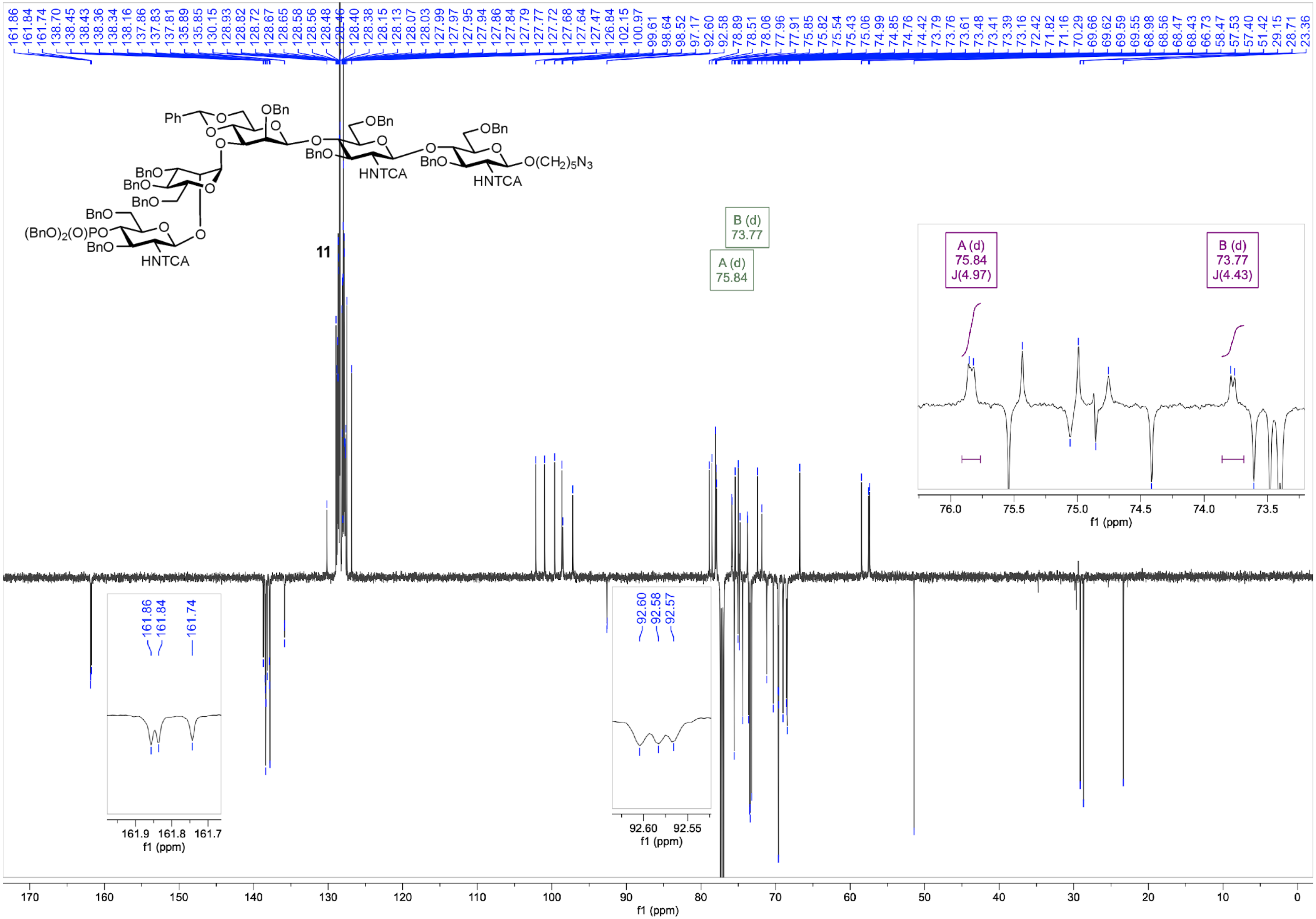

***11** HSQC spectrum*

600 MHz for ^{1}H in $CDCl_3$, Pulse Sequence: hsqcedetgpsisp2.3, NS 8, NUS 25%

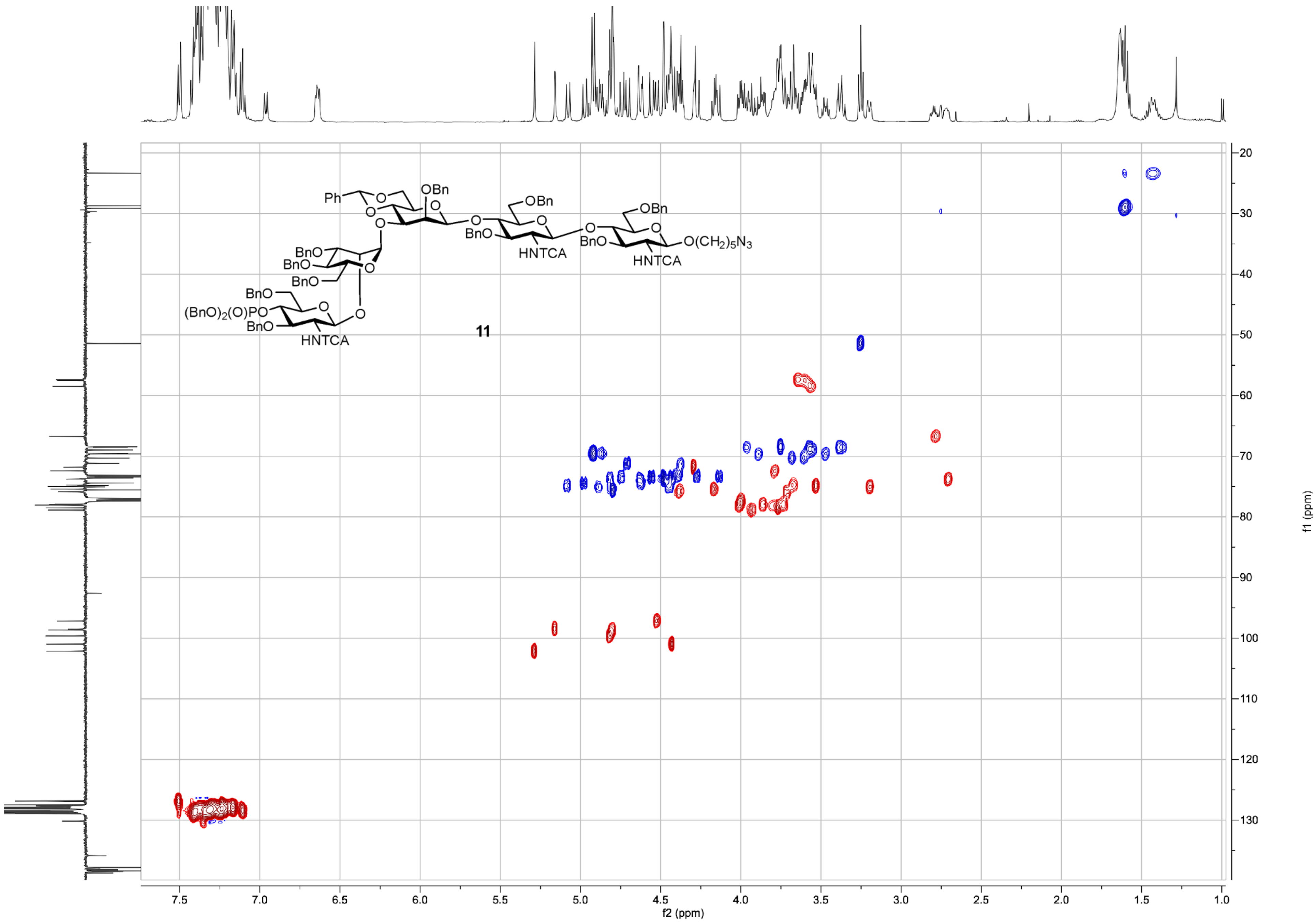

***11** HSQC with splitting in F2 phase*

600 MHz for ^{1}H in $CDCl_3$, Pulse Sequence: hsqcedetgpsisp2.3, NS 2, NUS 25%

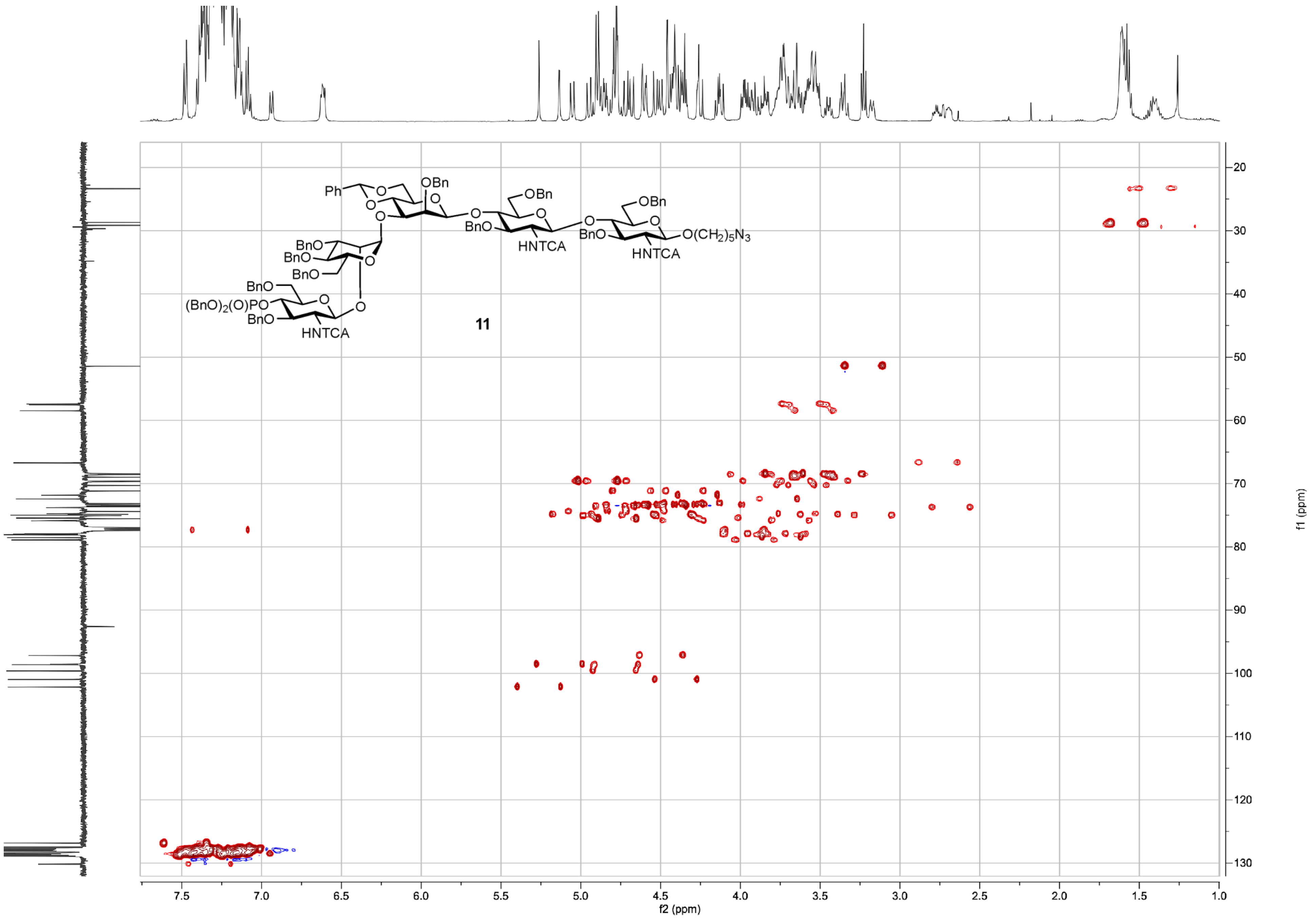

12 *^{1}H NMR spectrum*

600 MHz in $CDCl_3$

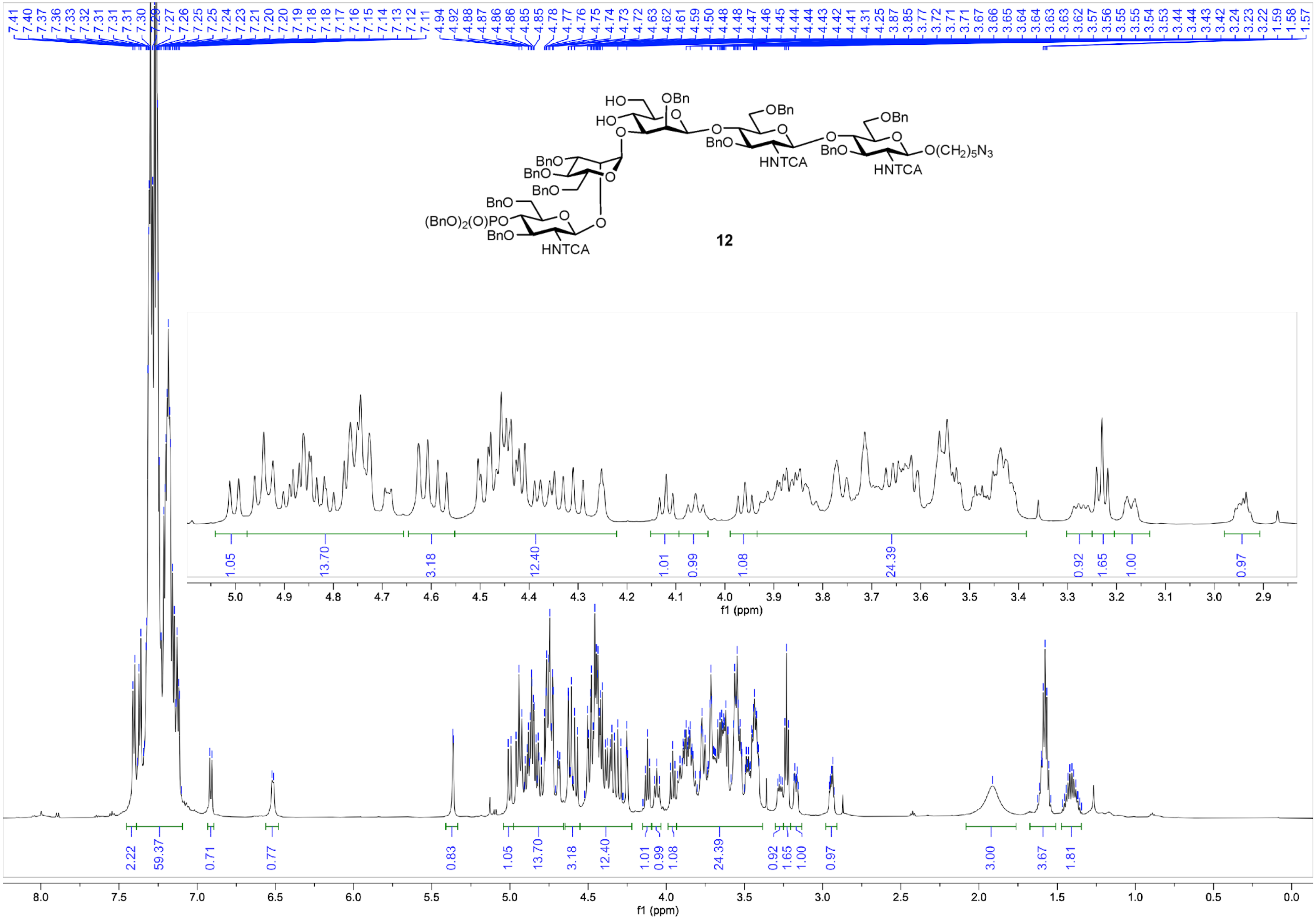

12 DEPTQ135 ^{13}C NMR spectrum

151 MHz in $CDCl_3$, Pulse Sequence: deptqgpsp.2, NS 100

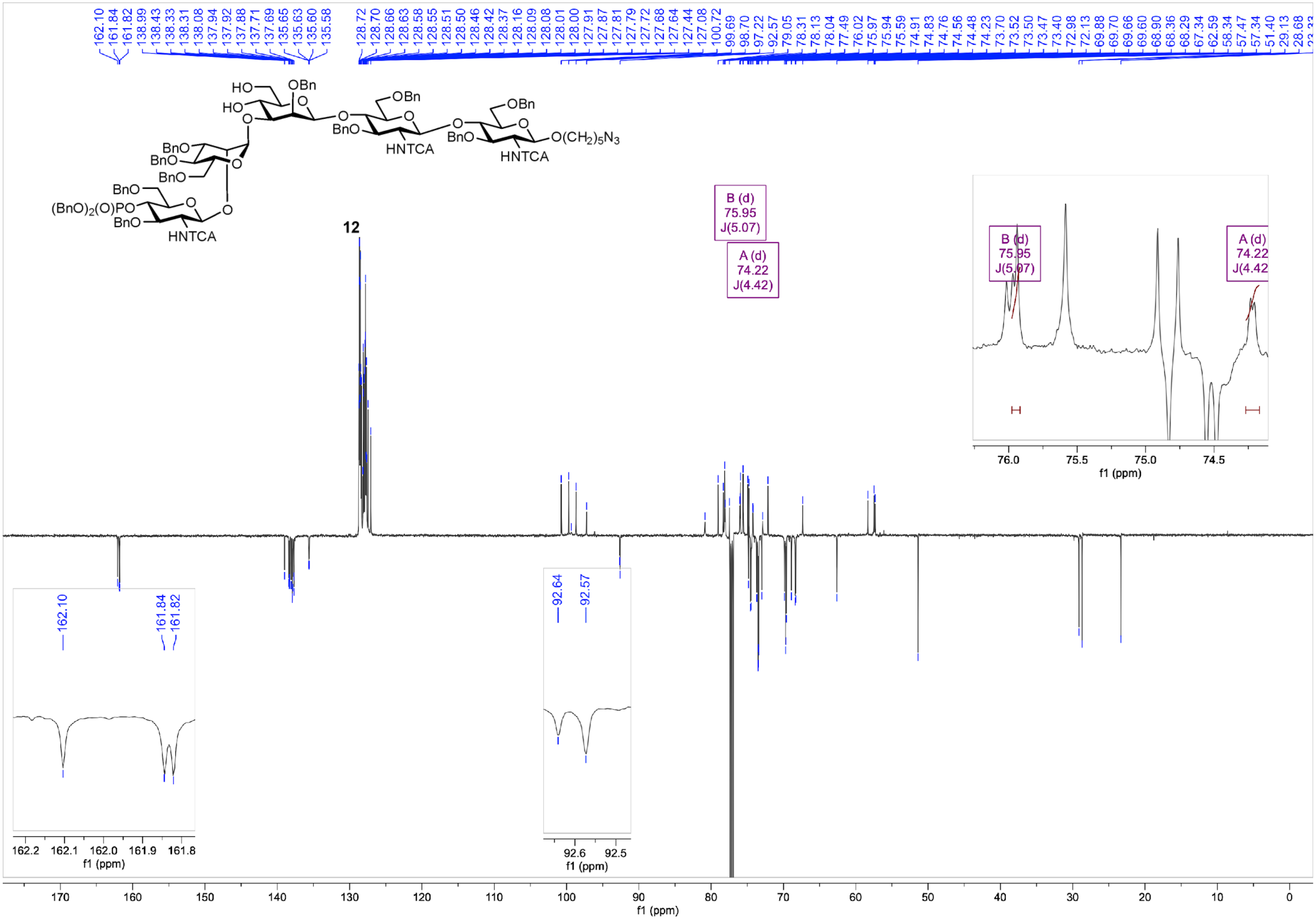

***12** HSQC spectrum*

600 MHz for ^1H in $CDCl_3$, Pulse Sequence: hsqcedetgpsisp2.3, NS 4, NUS 25%

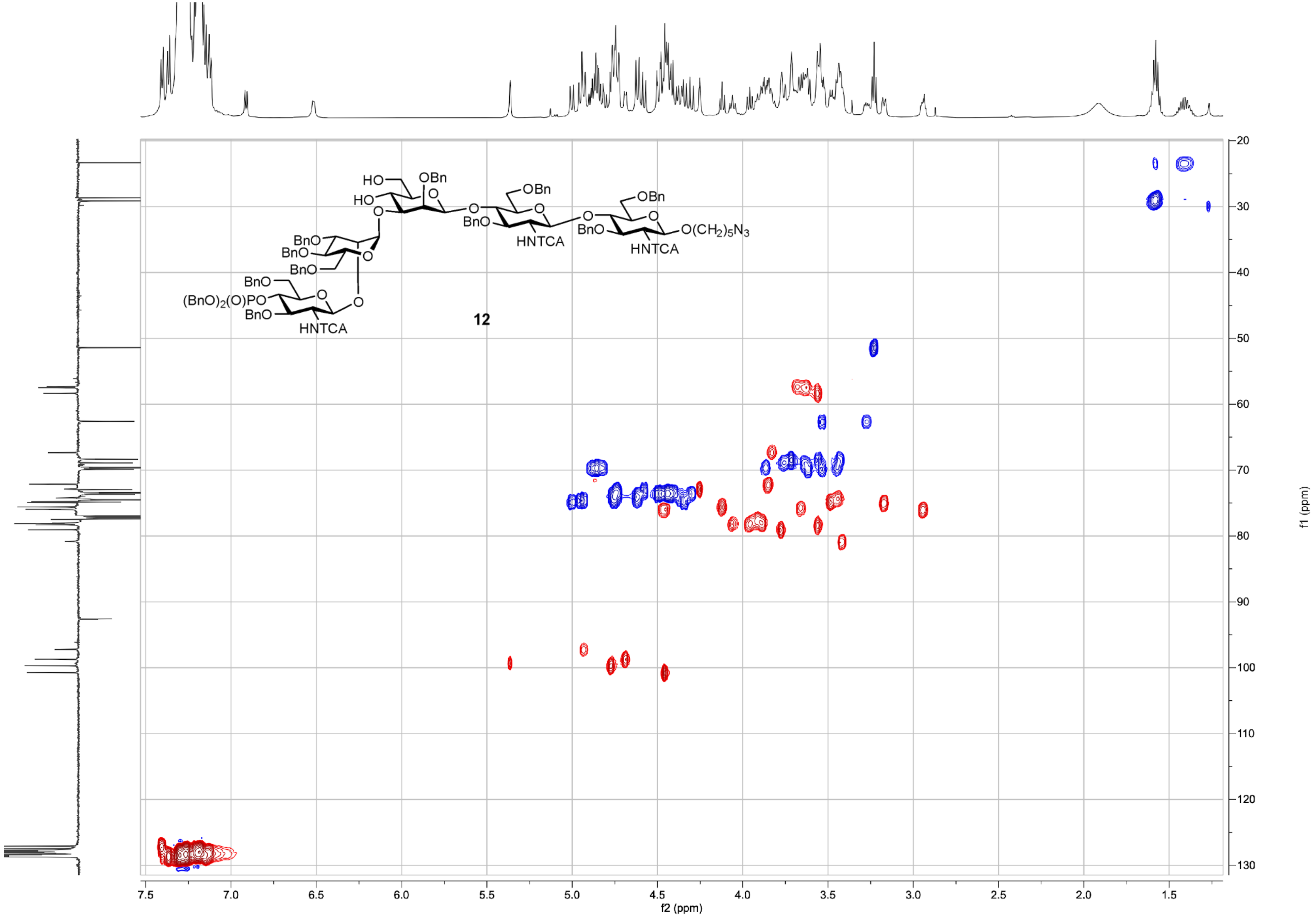

***12** HSQC spectrum with splitting via F2 phase*

Pulse Sequence: hsqcetgpipjcsp, NS 2, NUS 25%

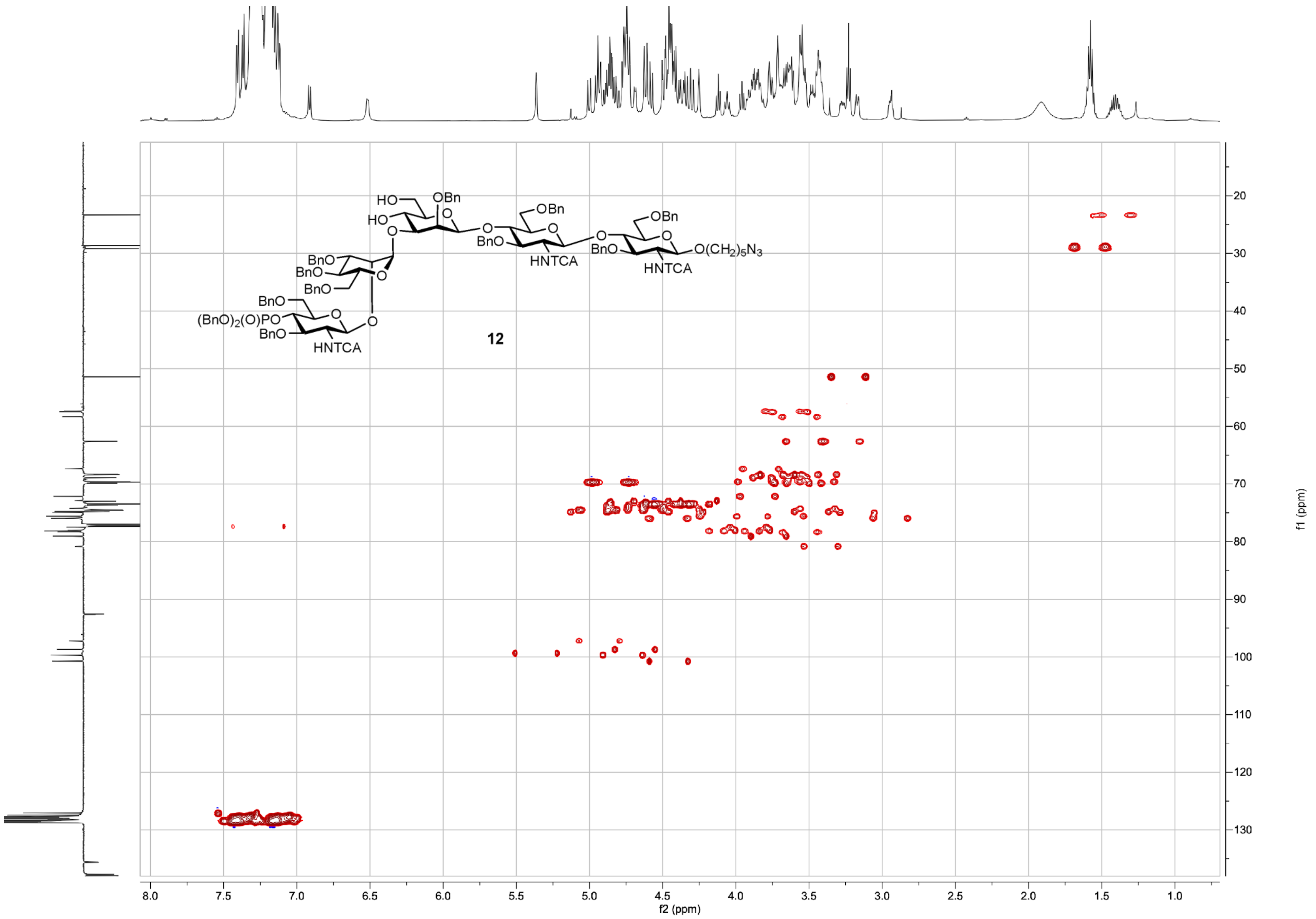

13 *^{1}H spectrum*

600 MHz in $CDCl_3$

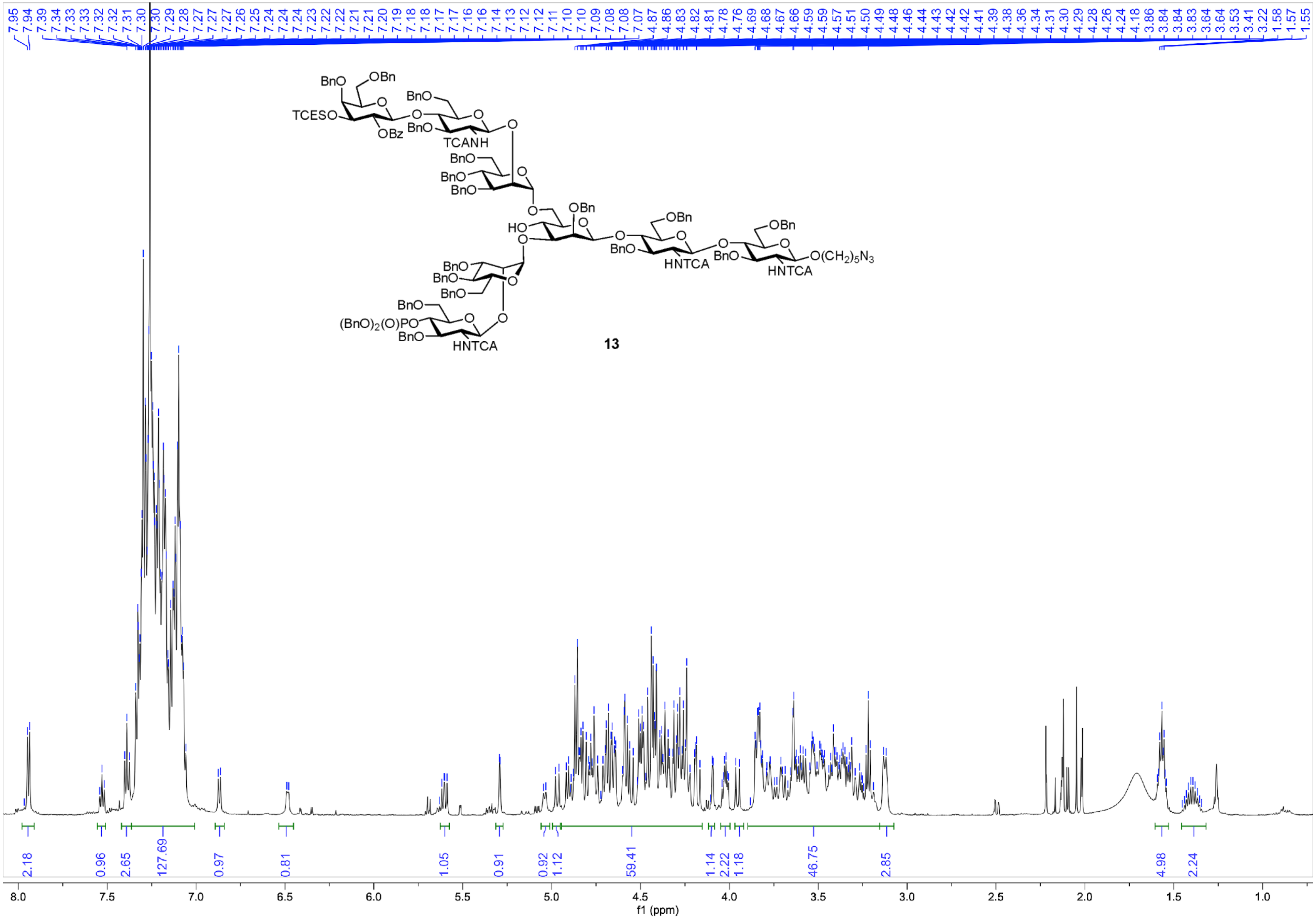

13 HSQC spectrum

600 MHz for ^{1}H in $CDCl_3$, Pulse Sequence: hsqcedetgpsisp2.3, NS 2, NUS 25%

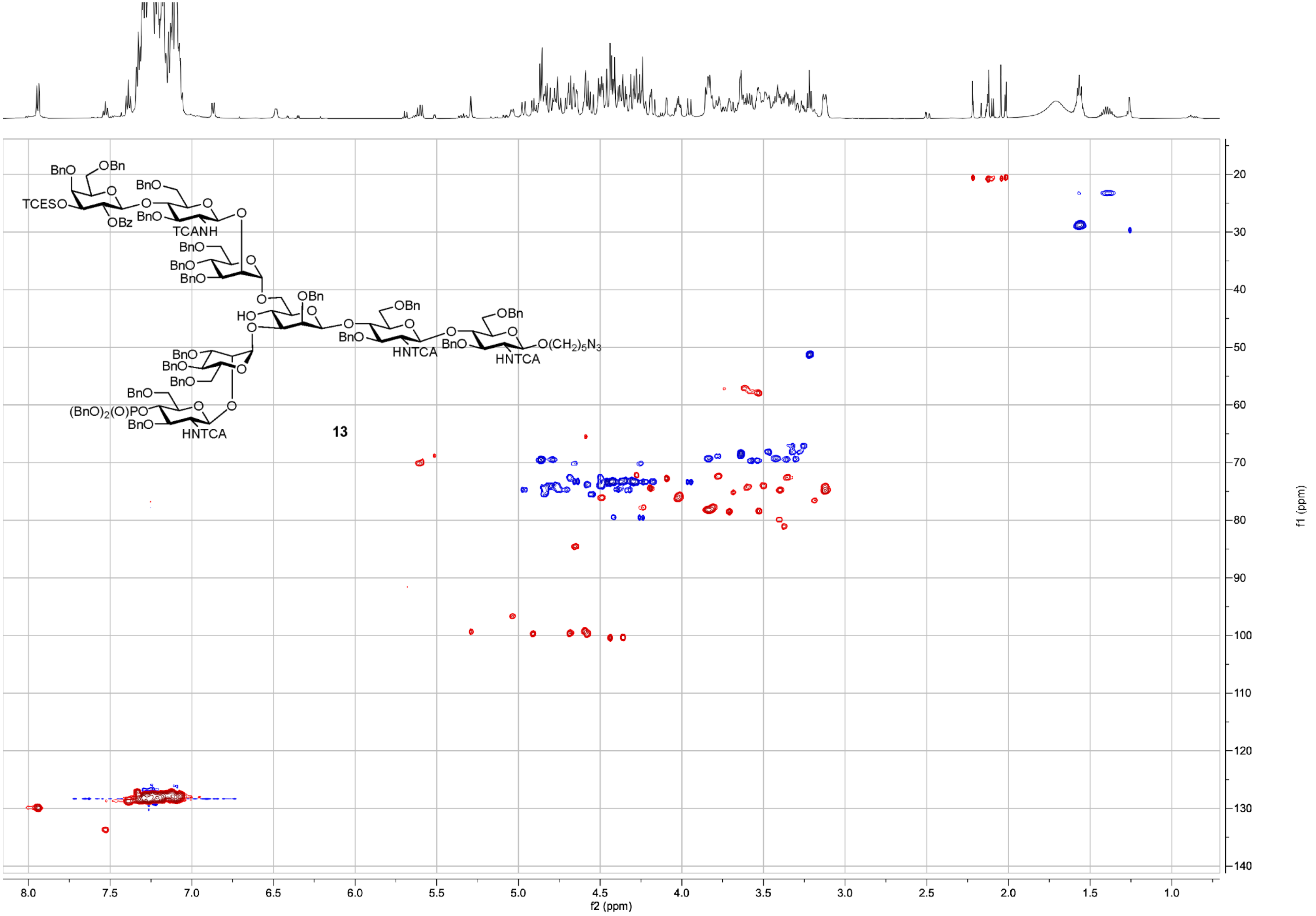

14 *^{1}H NMR spectrum*

600 MHz, $CDCl_3$

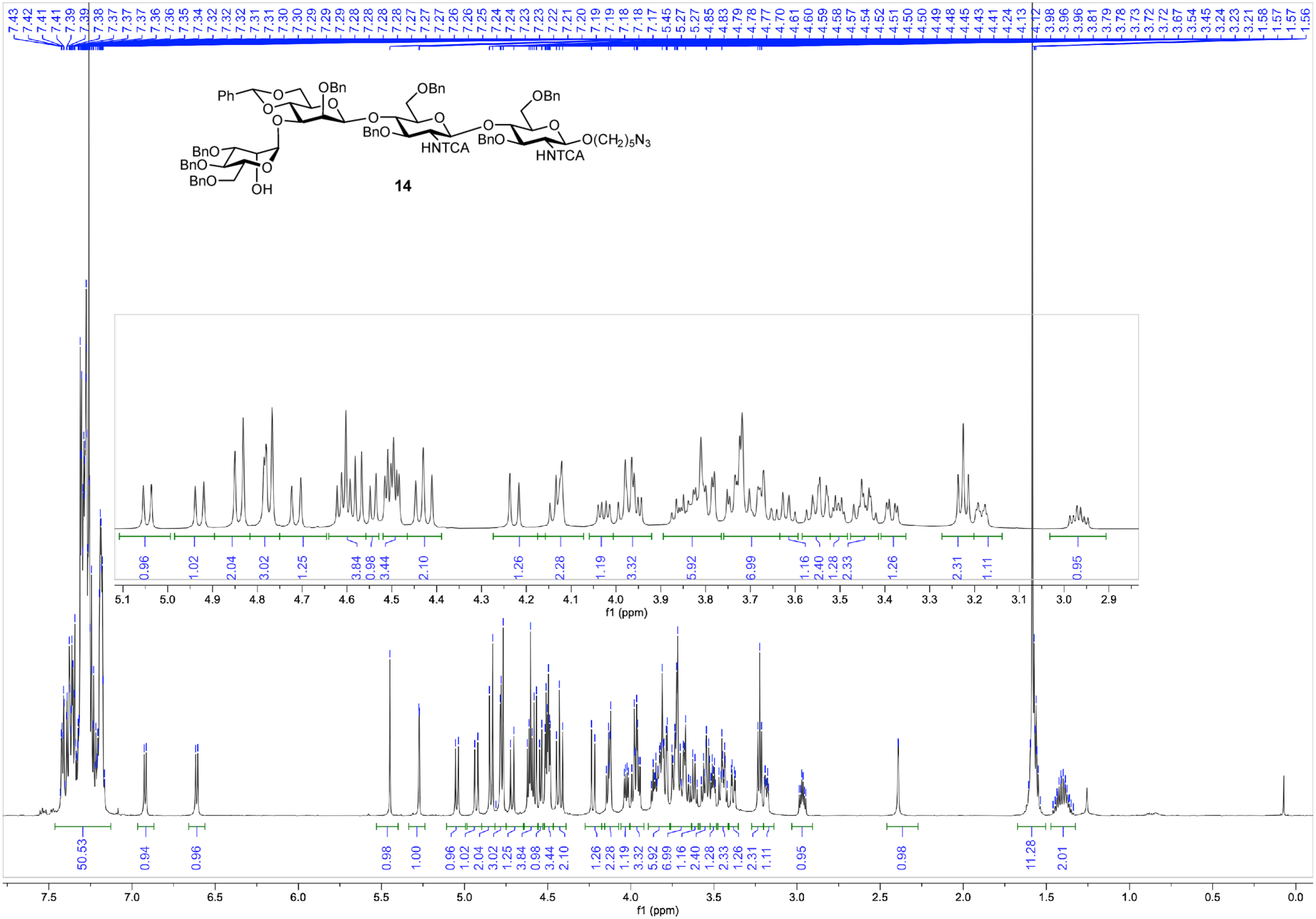

***14** DEPTQ135 ^{13}C NMR spectrum*

151 MHz in $CDCl_3$, Pulse Sequence: deptqgpsp.2, NS 100

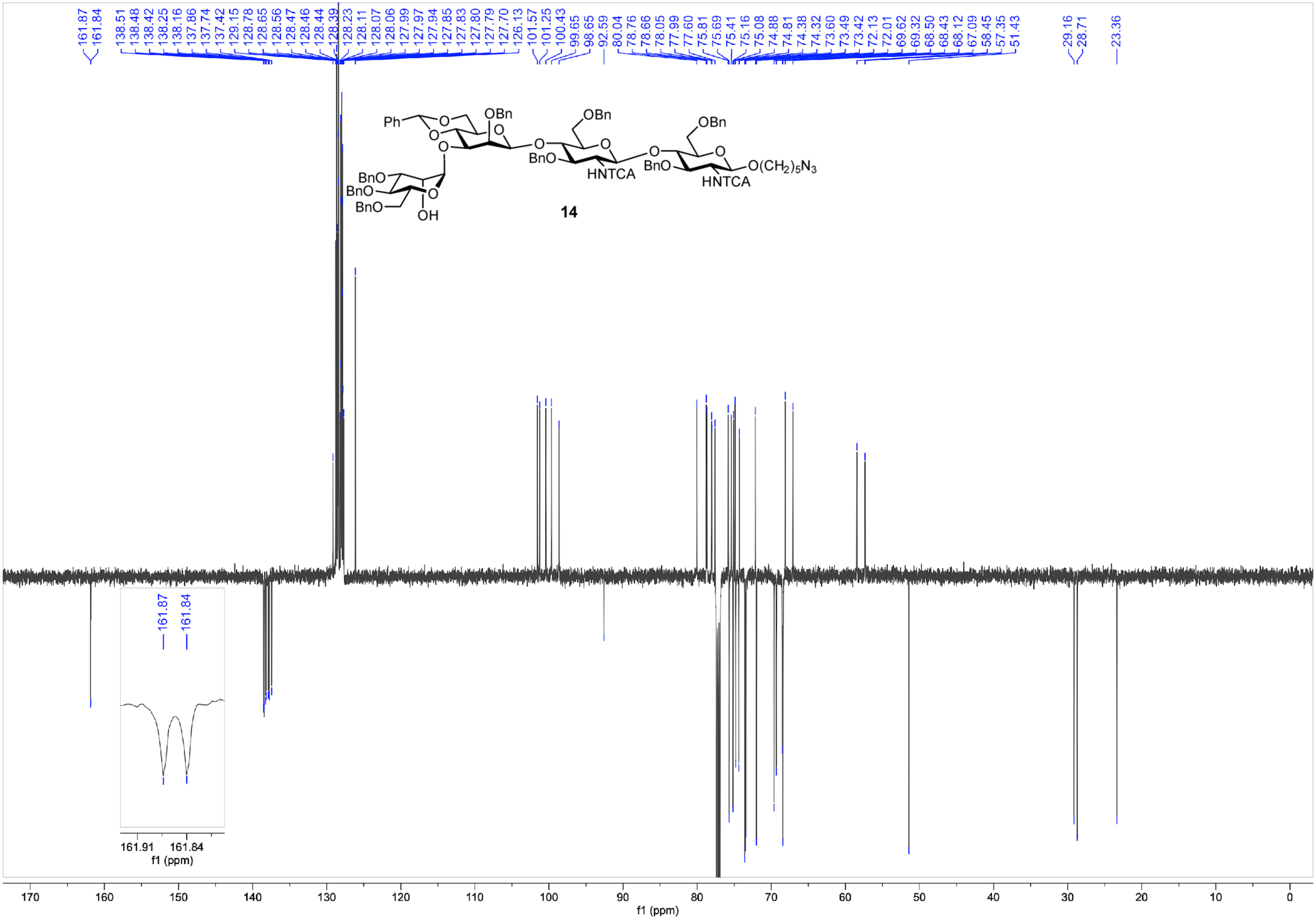

14 *HSQC spectrum*

600 MHz for ^{1}H in $CDCl_3$, Pulse Sequence: hsqcedetgpsisp2.3, NS 4, NUS 25%

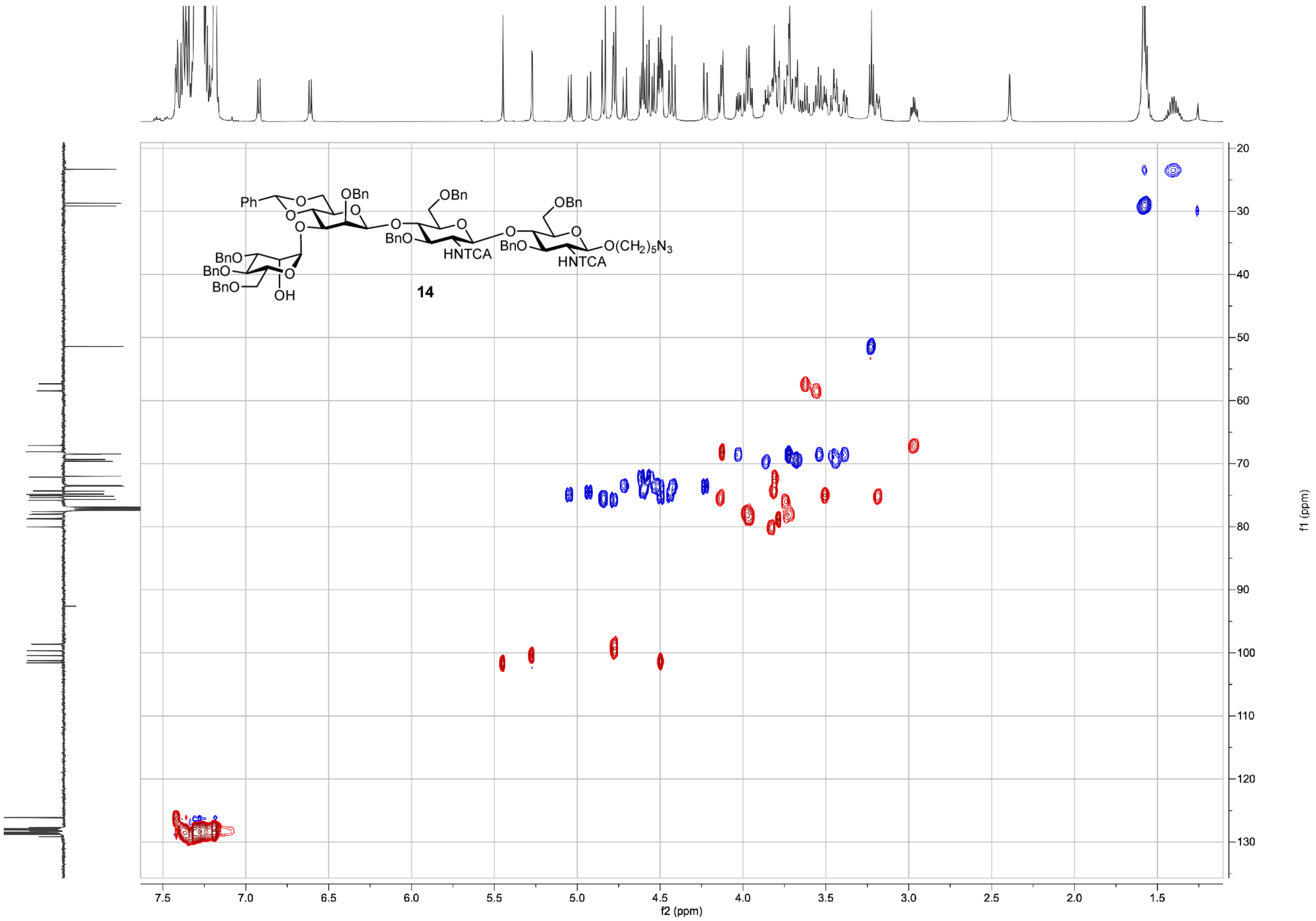

__14__ HSQC spectrum with splitting via F2 phase

600 MHz for ^{1}H in $CDCl_3$, Pulse Sequence: hsqcetgpipjcsp.2, NS 2, NUS 25%

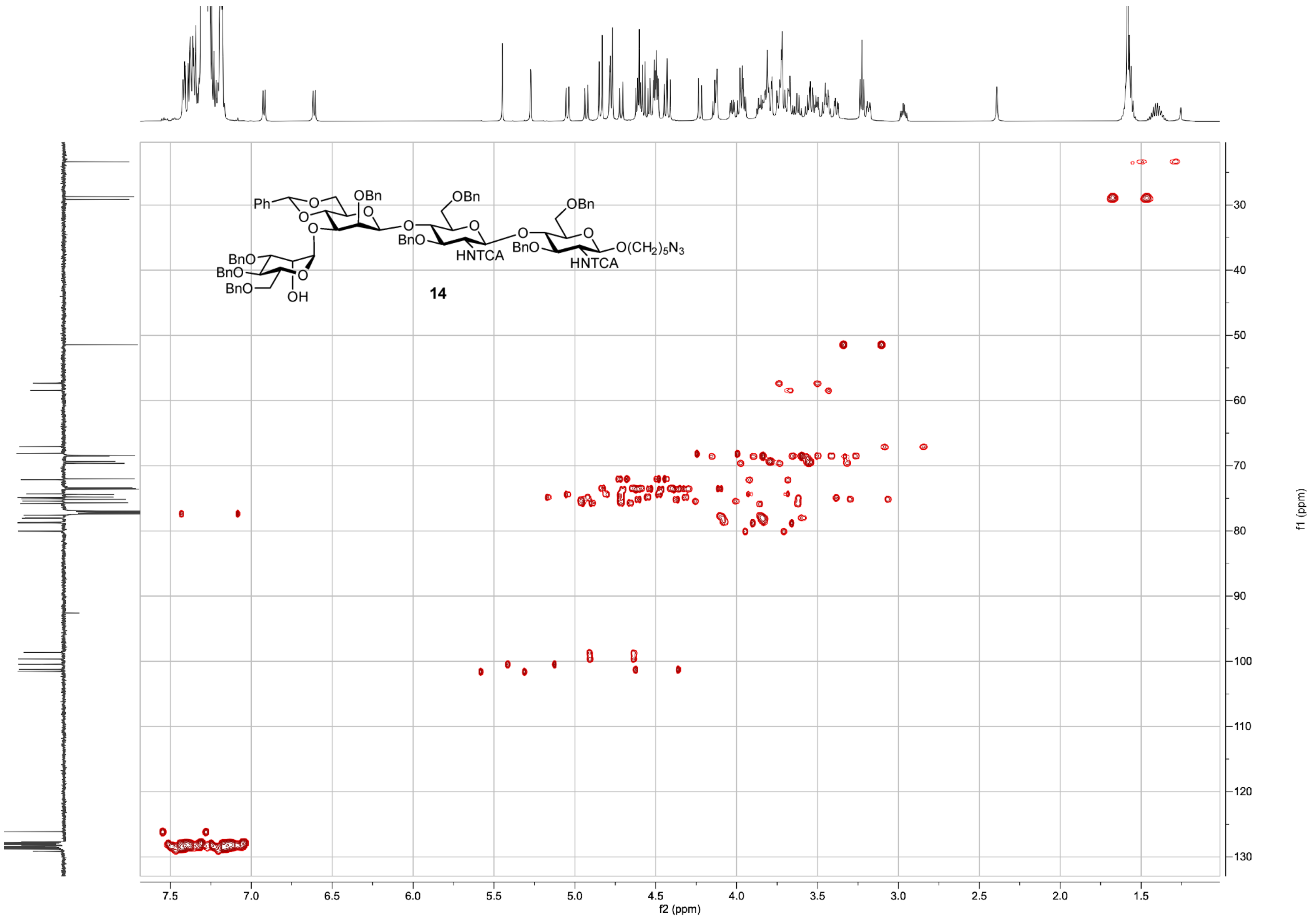

S23 *¹H NMR spectrum*

600 MHz, $CDCl_3$

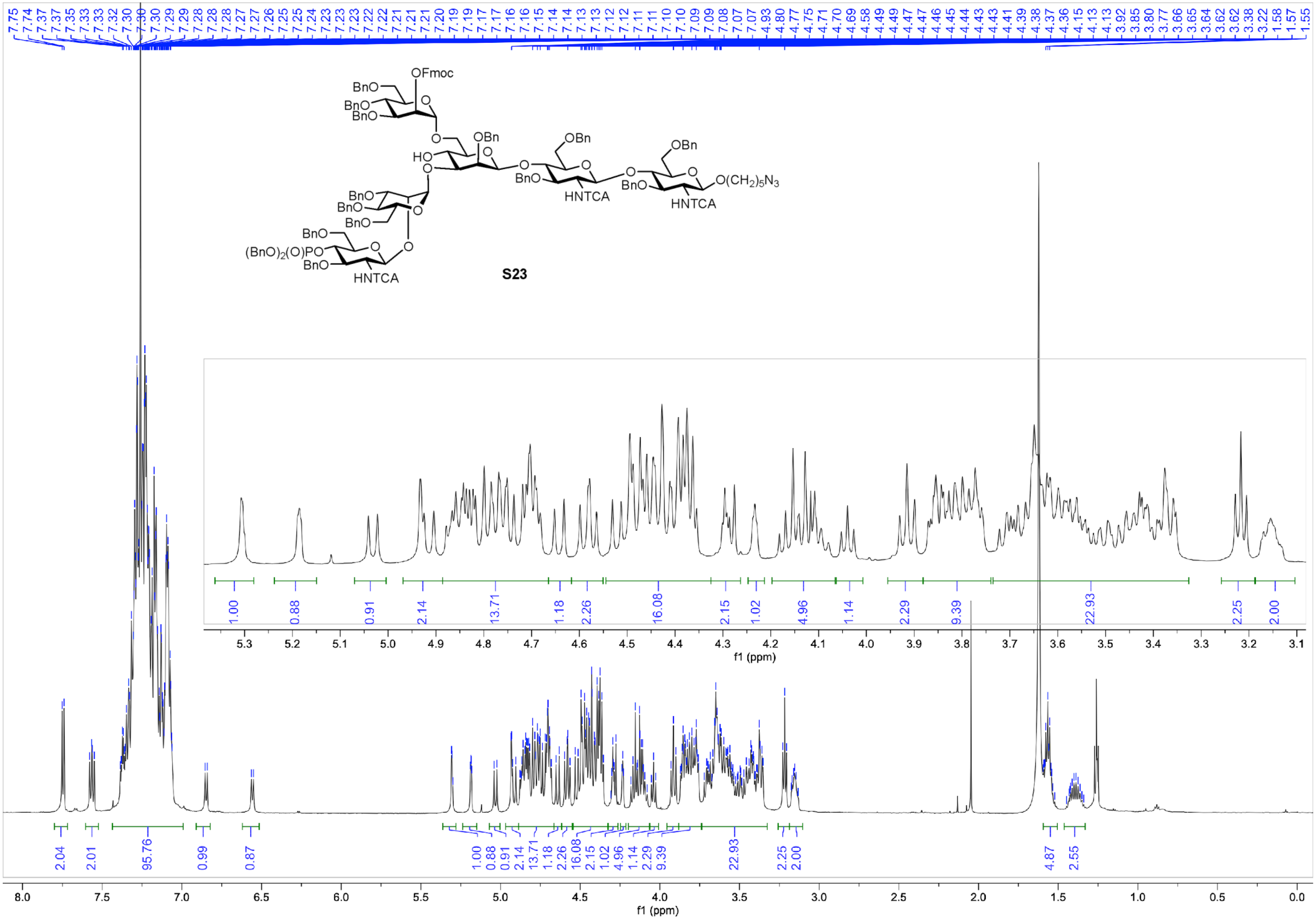

***S23** DEPTQ135 ^{13}C NMR spectrum*

151 MHz in $CDCl_3$, Pulse Sequence: deptqgpsp.2, NS 128

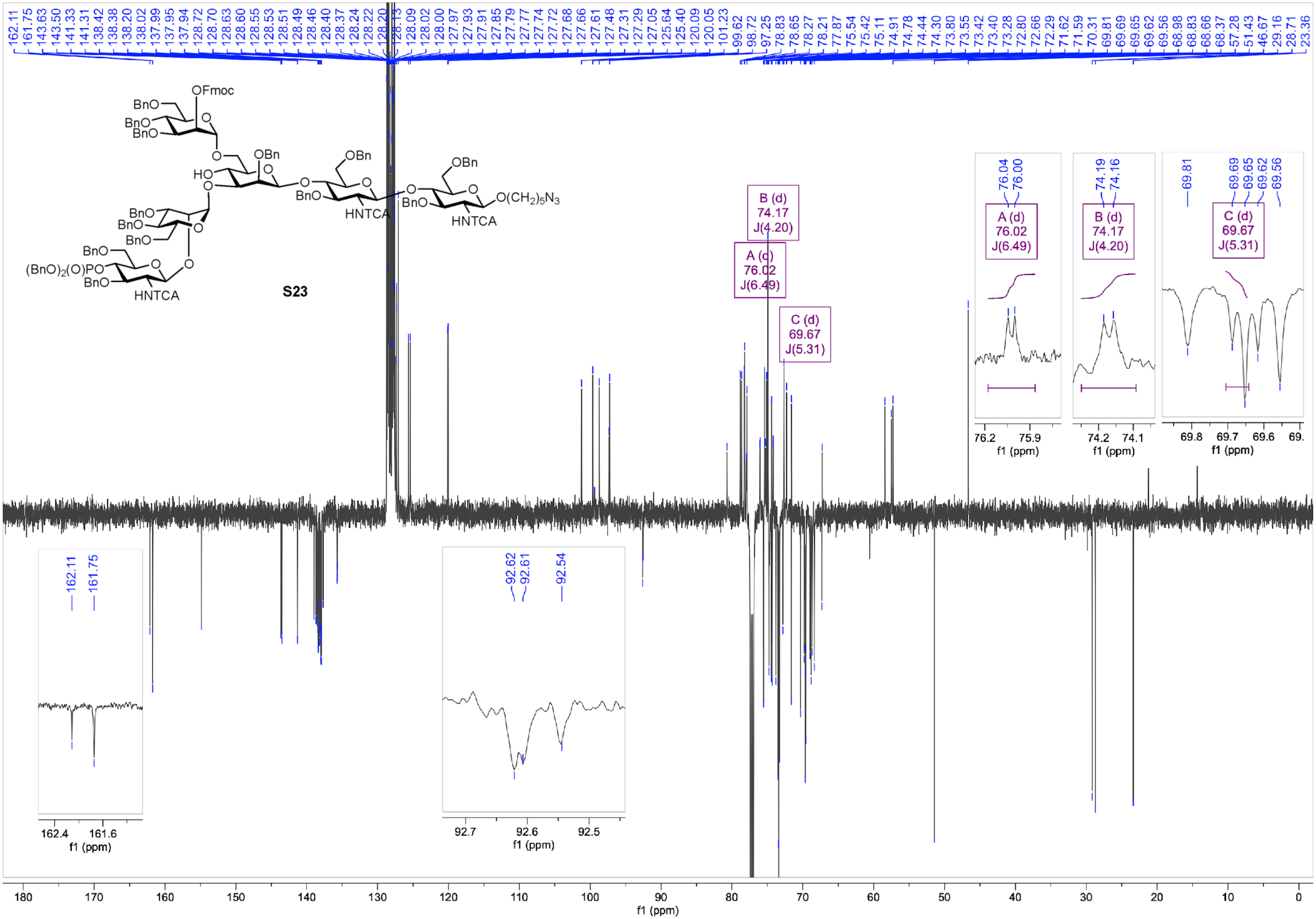

***S23** HSQC spectrum*

600 MHz for ^{1}H in $CDCl_3$, Pulse Sequence: hsqcedetgpsisp2.3, NS 4, NUS 25%

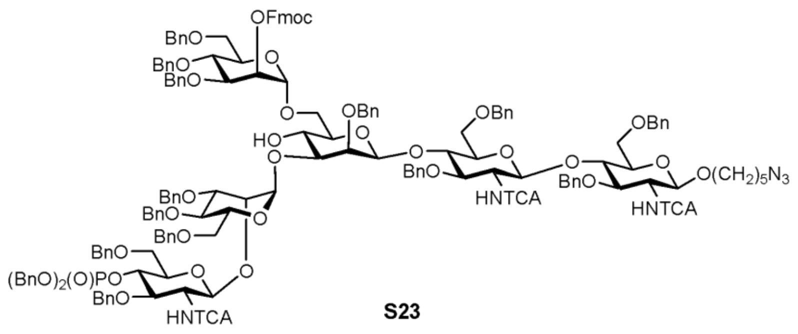

S23

***S23** HSQC spectrum with splitting via F2 phase*

600 MHz for ^{1}H in $CDCl_3$, Pulse Sequence: hsqcetgpipjcsp.2, NS 2, NUS 25%

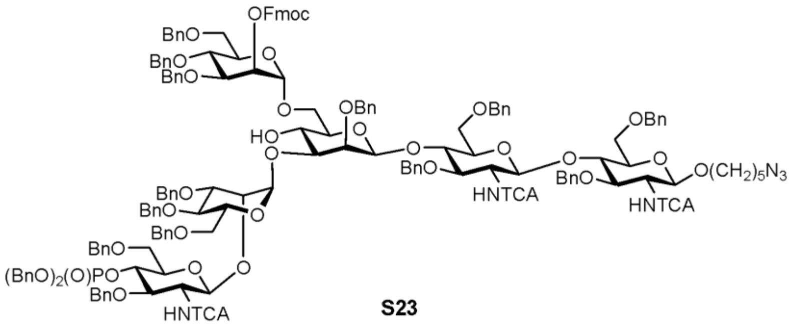

S23

S24 *^{1}H NMR spectrum*

600 MHz, $CDCl_3$

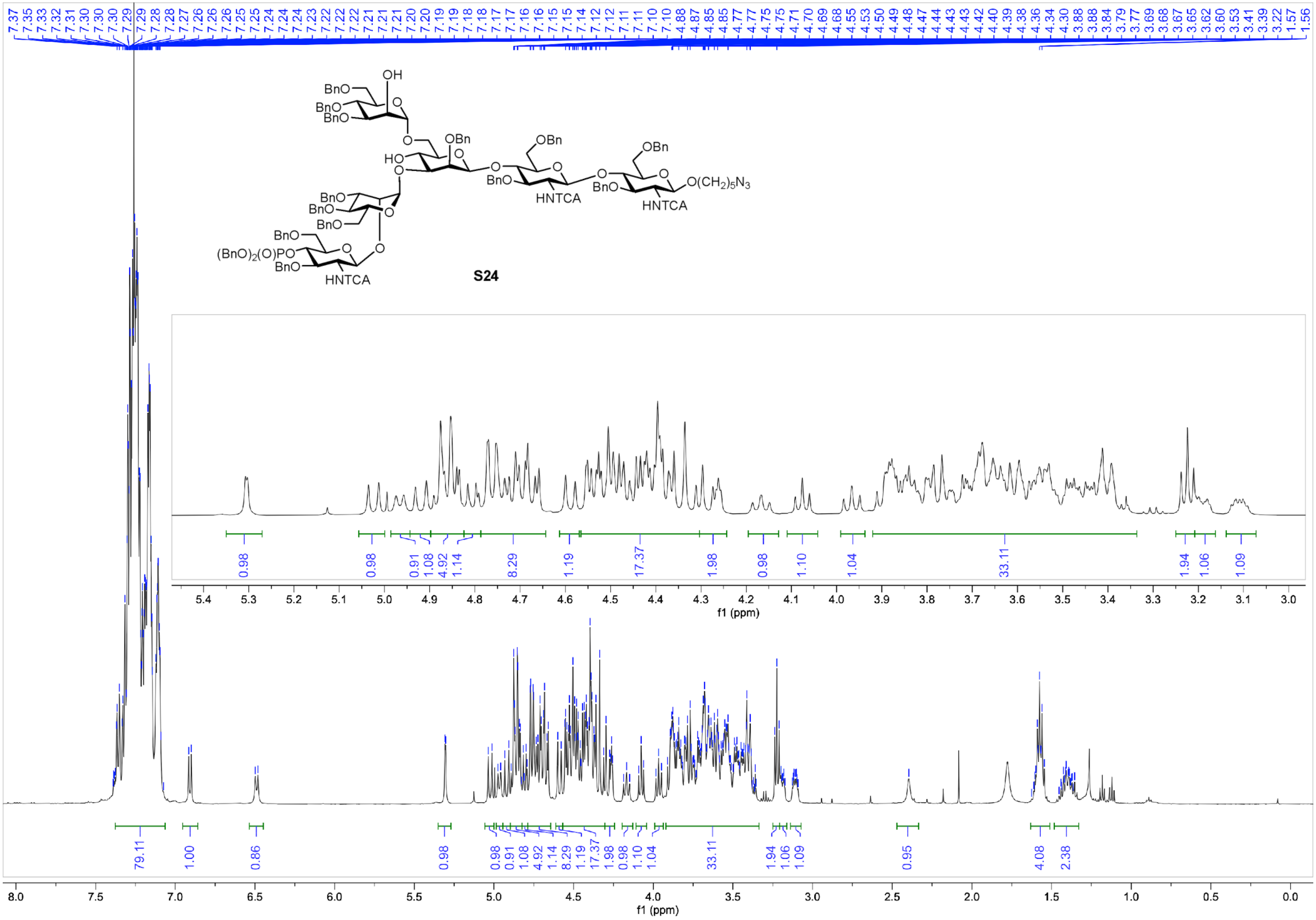

***S24** DEPTQ135 ^{13}C NMR spectrum*

151 MHz in $CDCl_3$, Pulse Sequence: deptqgpsp.2, NS 128

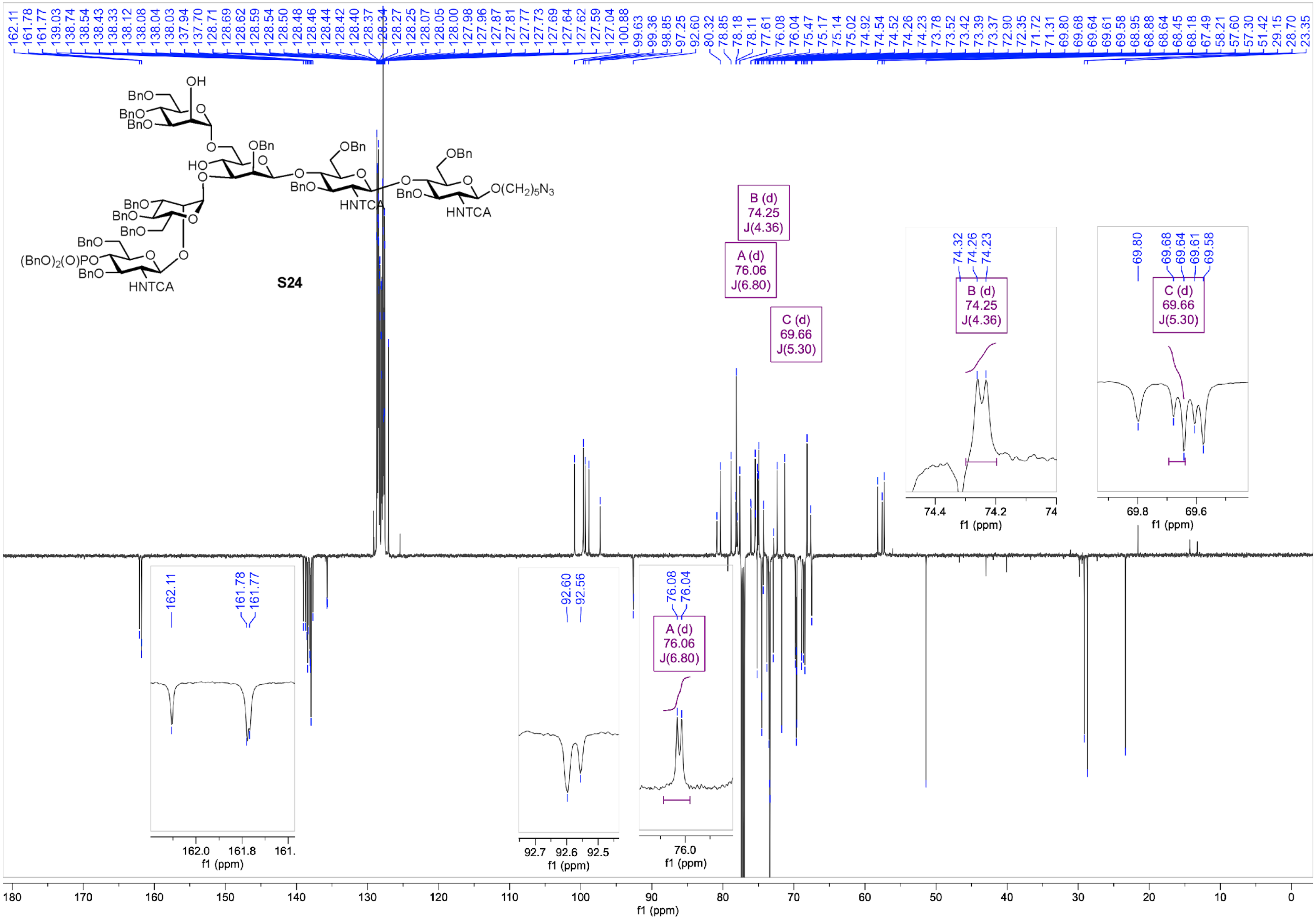

S24 HSQC spectrum

600 MHz for ^{1}H in $CDCl_3$, Pulse Sequence: hsqcedetgpsisp2.3, NS 4, NUS 25%

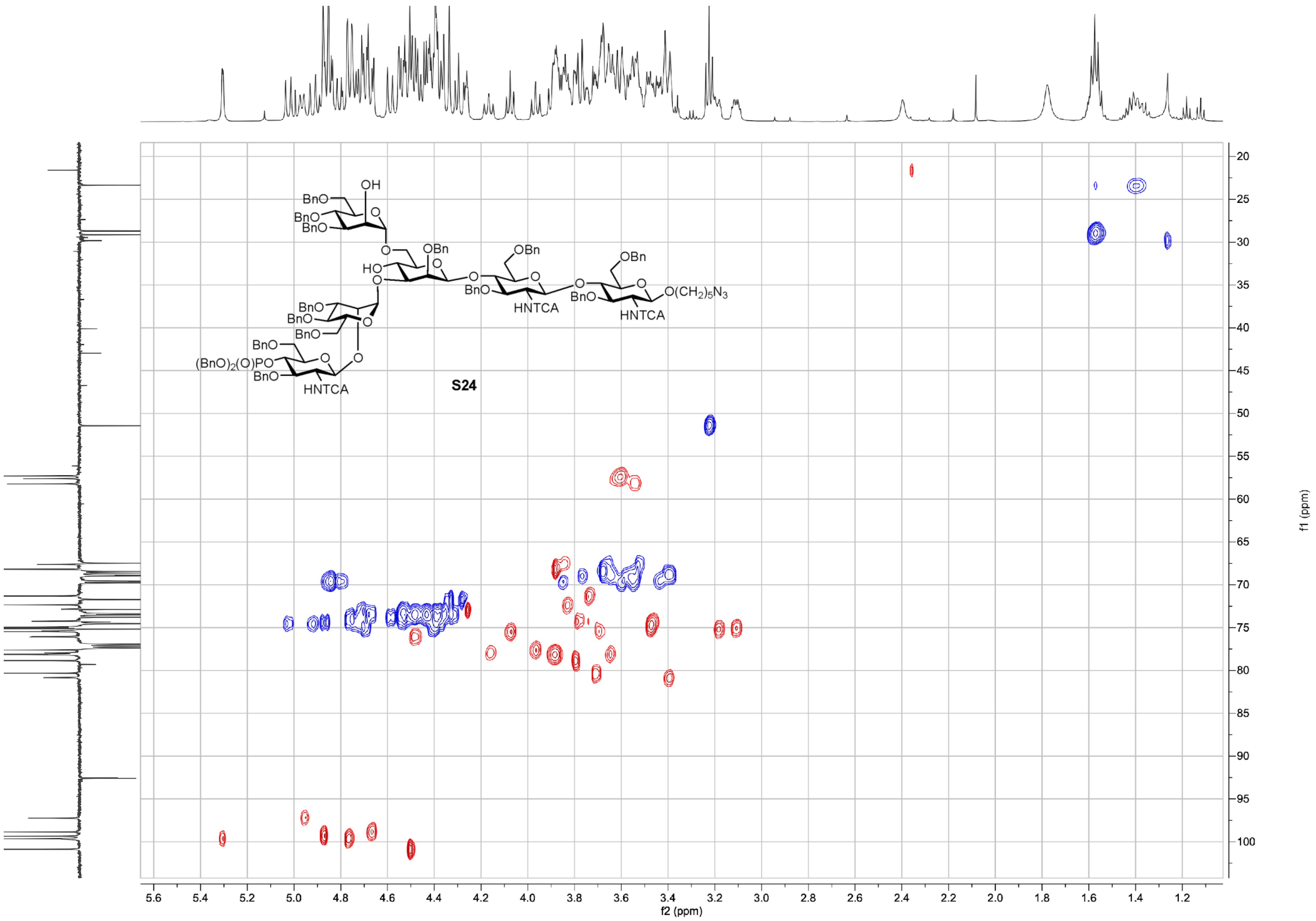

***S24** HSQC spectrum with splitting via F2 phase*

600 MHz for ^{1}H in $CDCl_3$, Pulse Sequence: hsqcetgpipjcsp, NS 2, NUS 25%

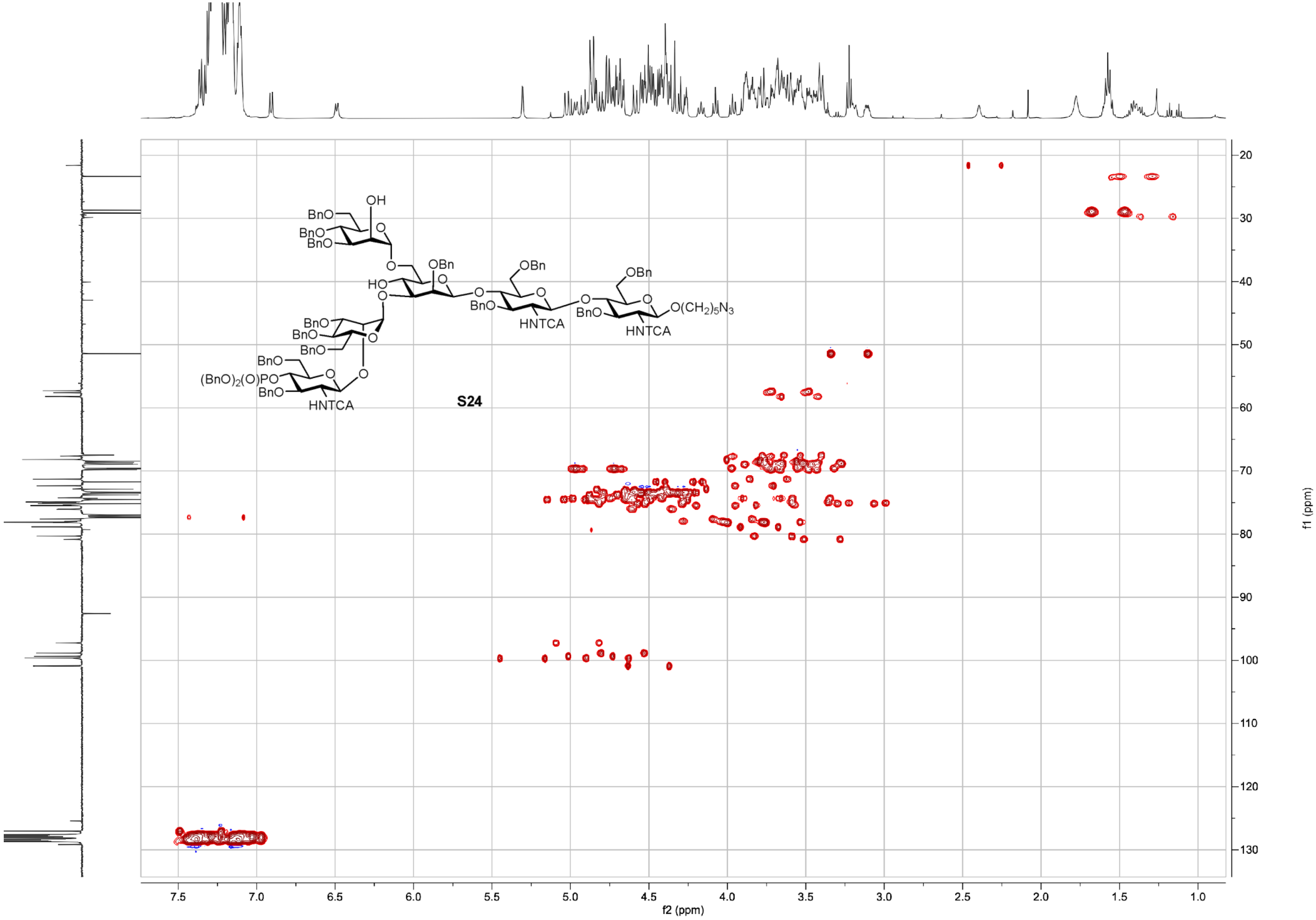

9a *¹H NMR spectrum*

600 MHz, $CDCl_3$

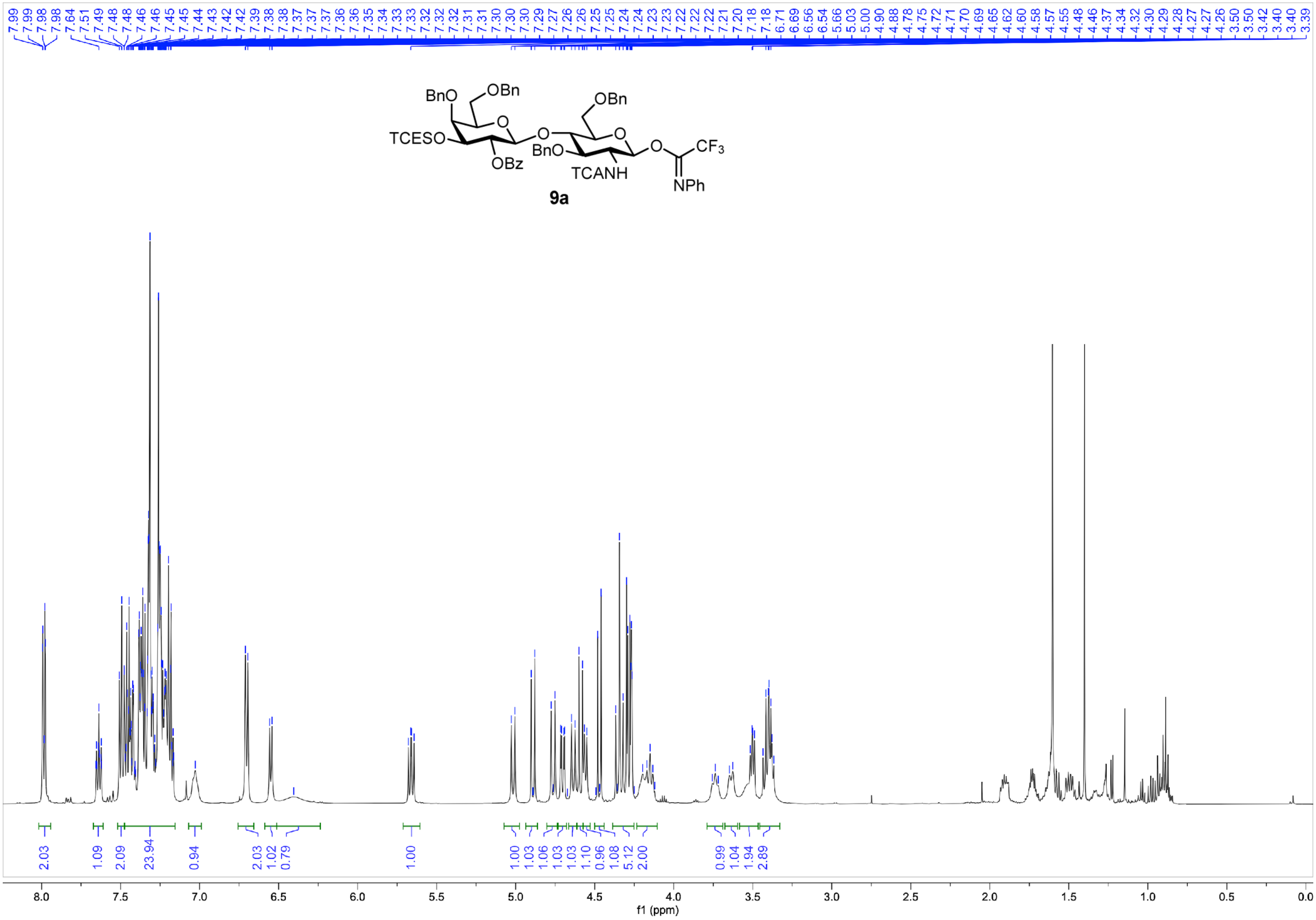

***9a** HSQC spectrum*

600 MHz for ^{1}H in $CDCl_3$, Pulse Sequence: hsqcedetgpsisp2.3, NS 4, NUS 25%

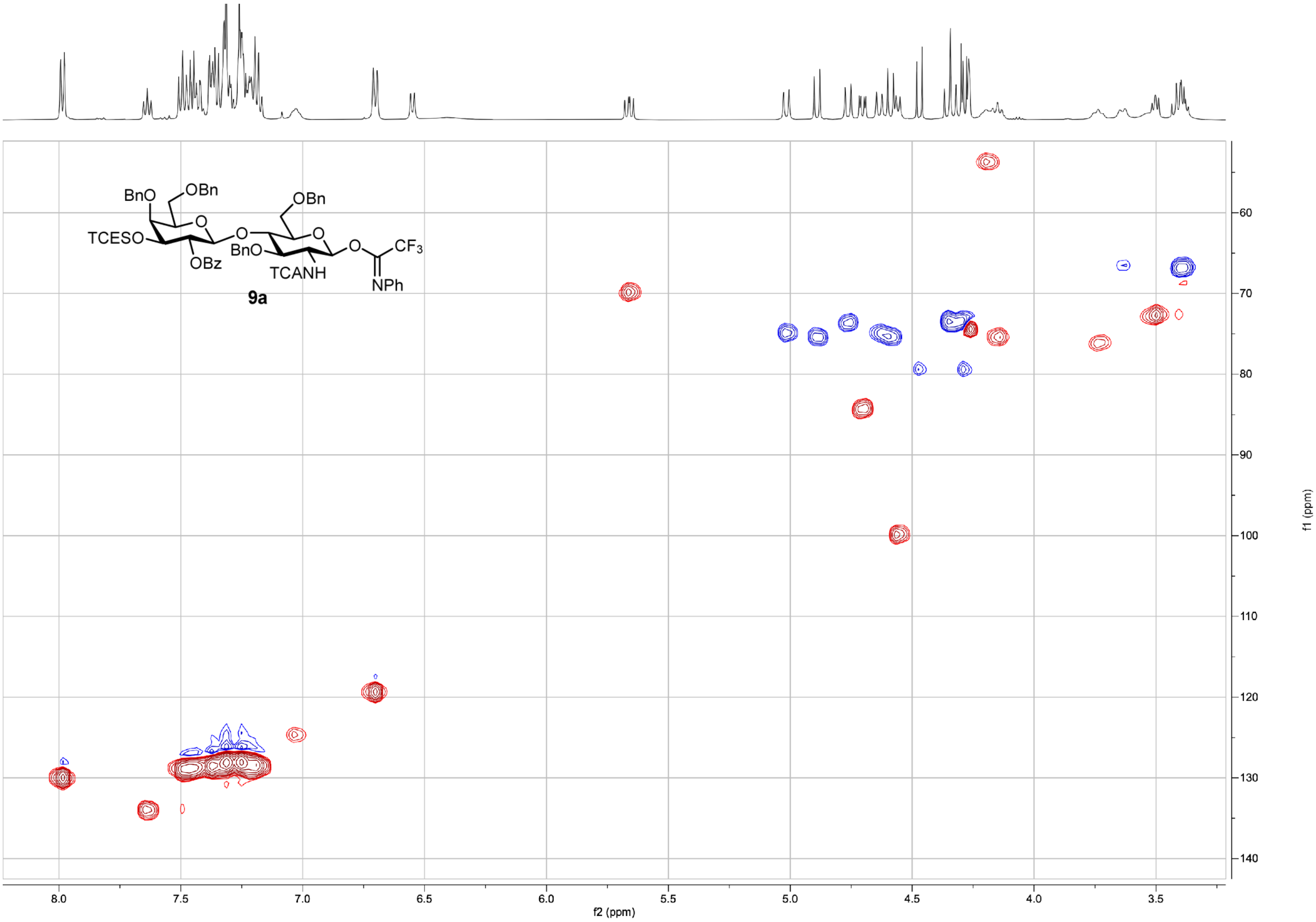

S17 *¹H NMR spectrum*

600 MHz, $CDCl_3$

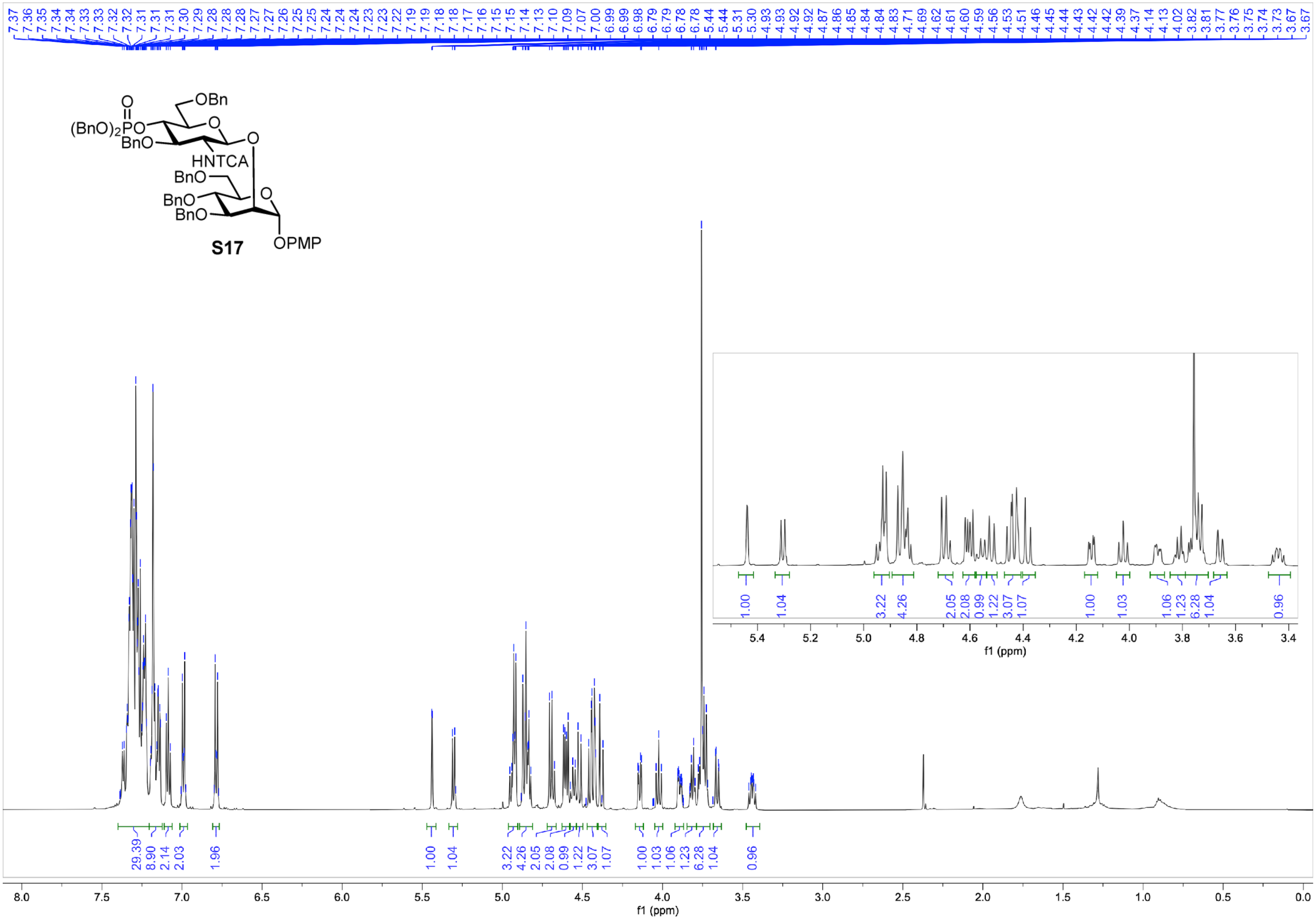

***S17** DEPTQ135 ^{13}C NMR spectrum*

151 MHz in $CDCl_3$, Pulse Sequence: deptqgpsp.2, NS 35

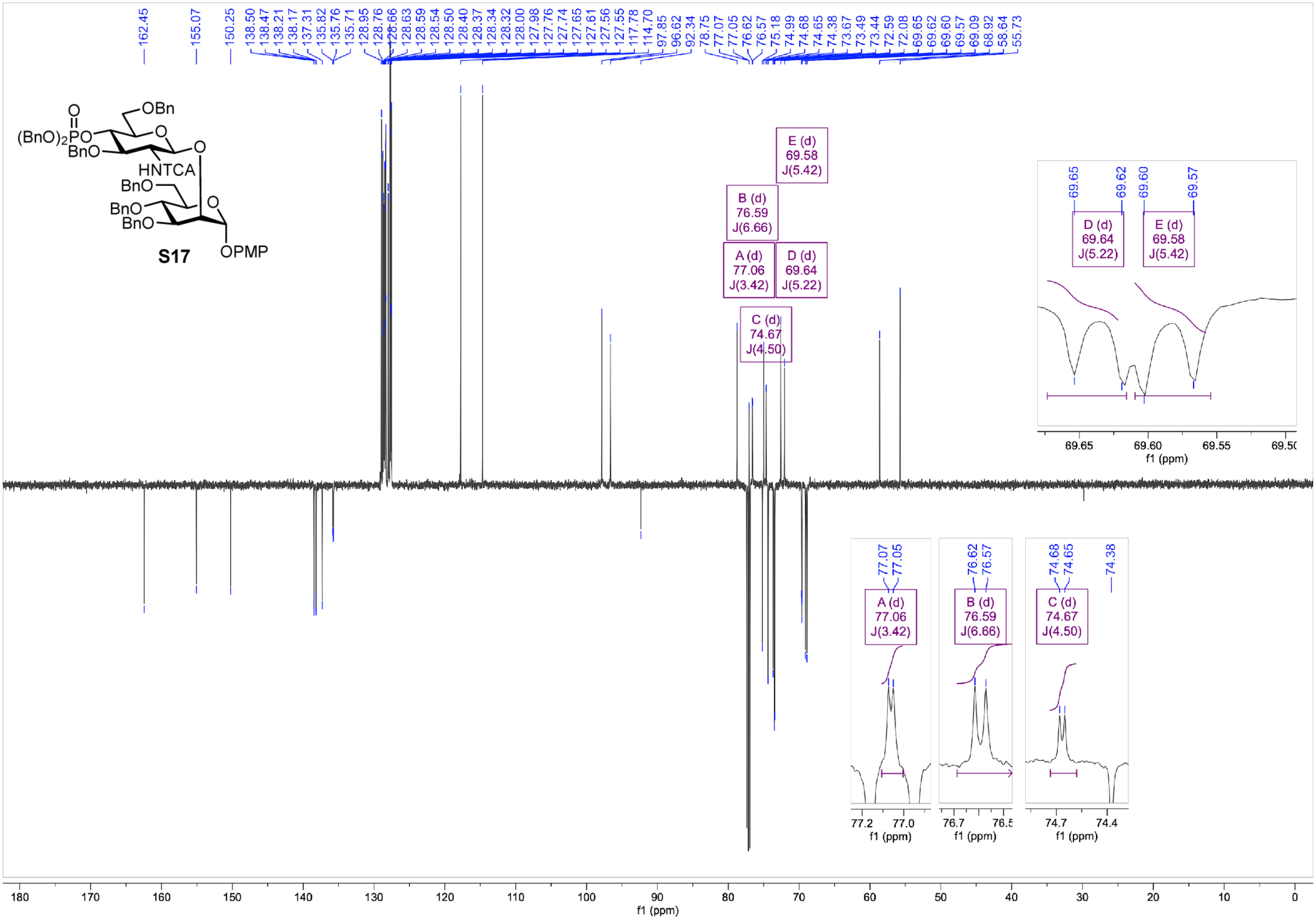

S17 *^{31}P spectrum*

162 MHz in $CDCl_3$

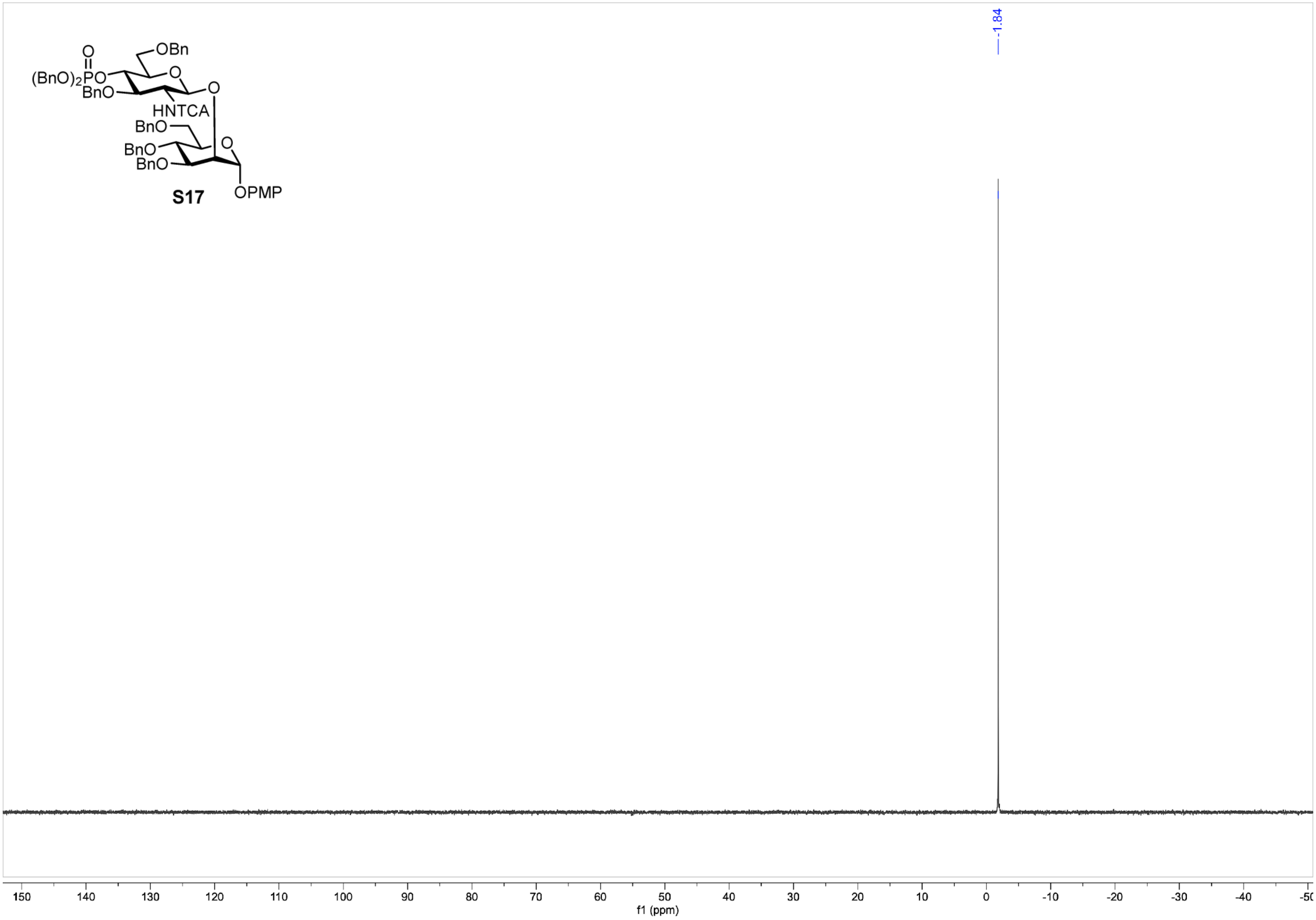

***S17** HSQC spectrum*

600 MHz for ^{1}H in $CDCl_3$, Pulse Sequence: hsqcedetgpsisp2.2, NS 2, NUS 25%

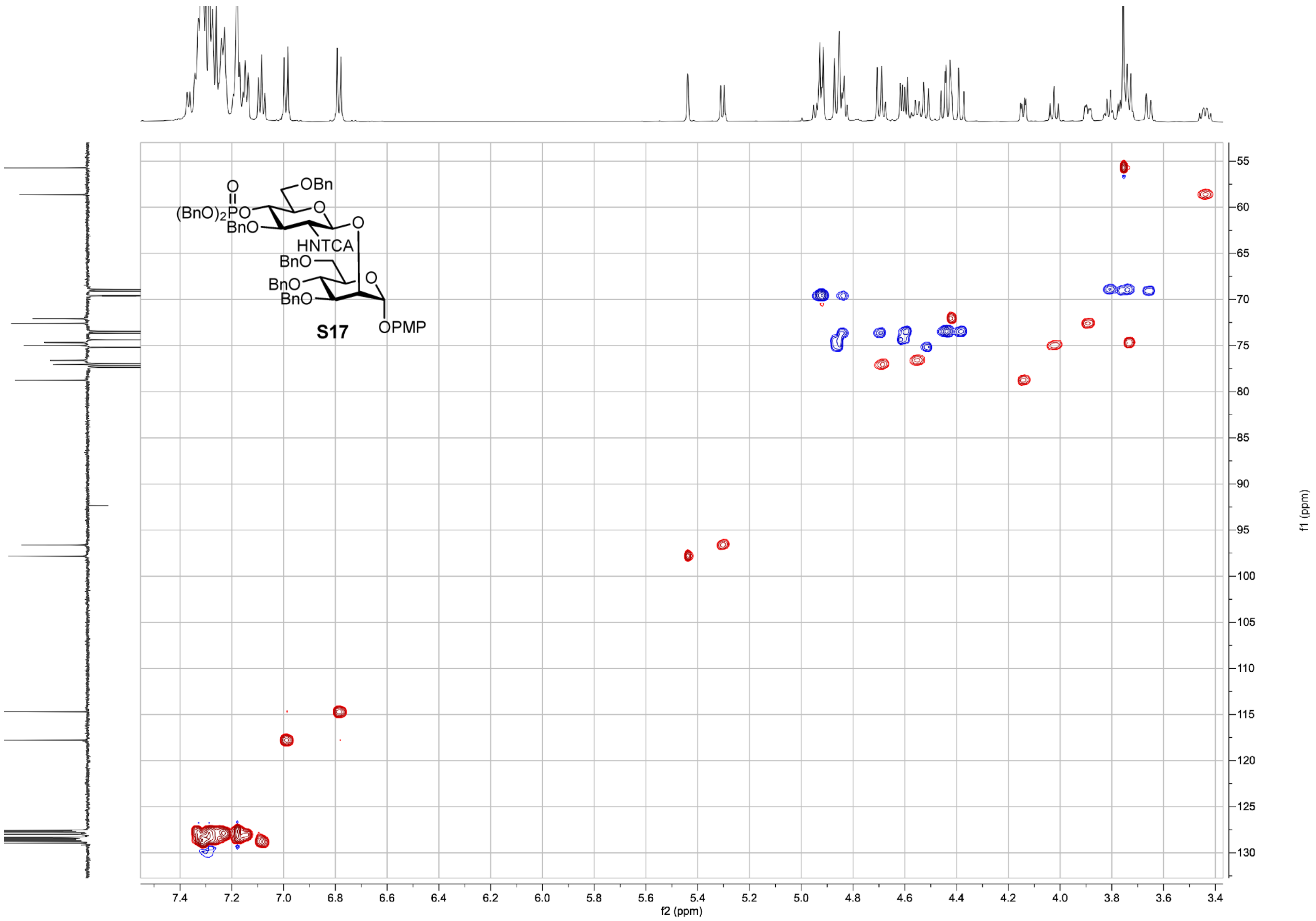

S18 *¹H NMR spectrum*

600 MHz, $CDCl_3$

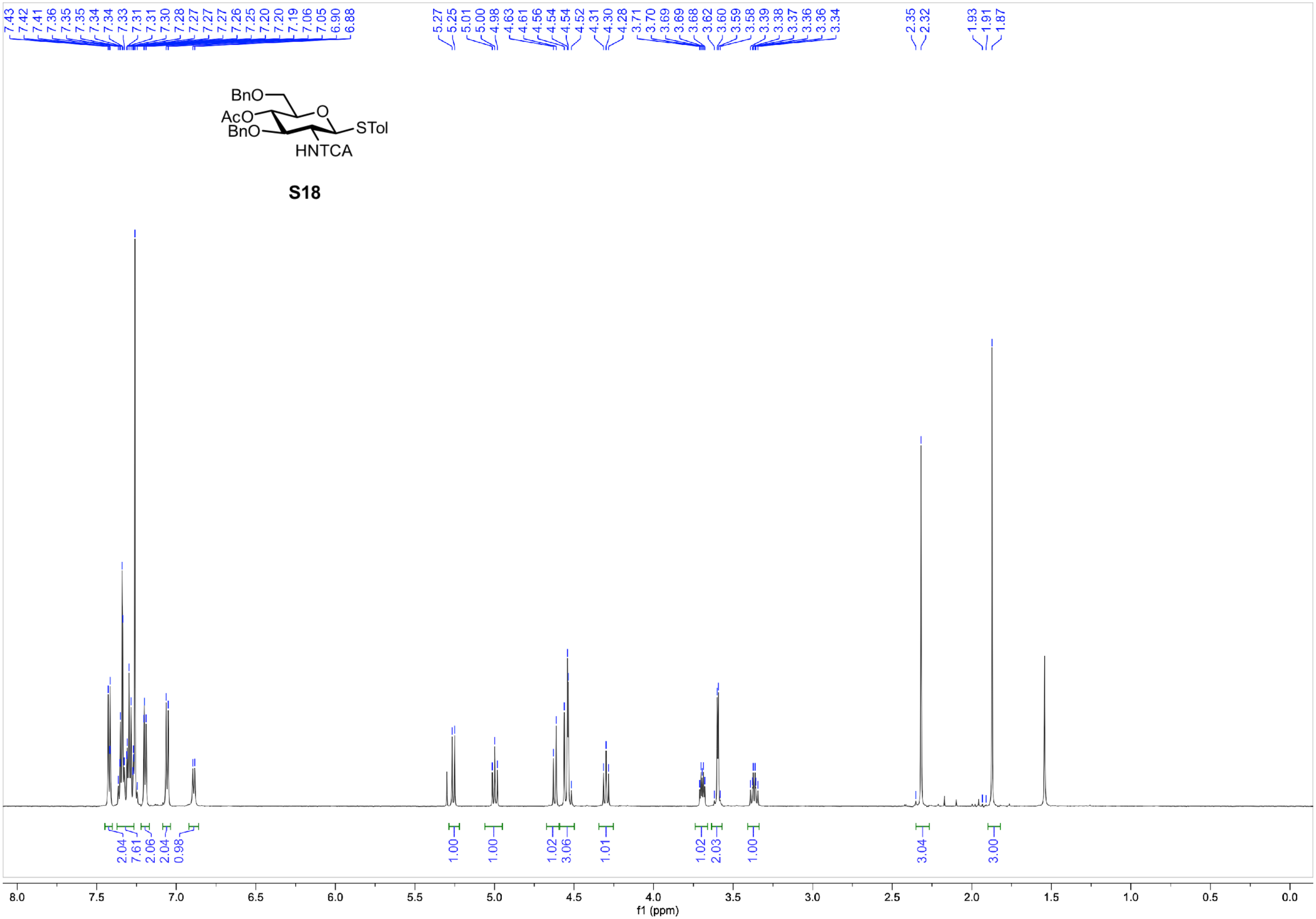

S18 *DEPTQ135* ^{13}C *NMR spectrum*

151 MHz in $CDCl_3$, Pulse Sequence: deptqgpsp.2, NS 21

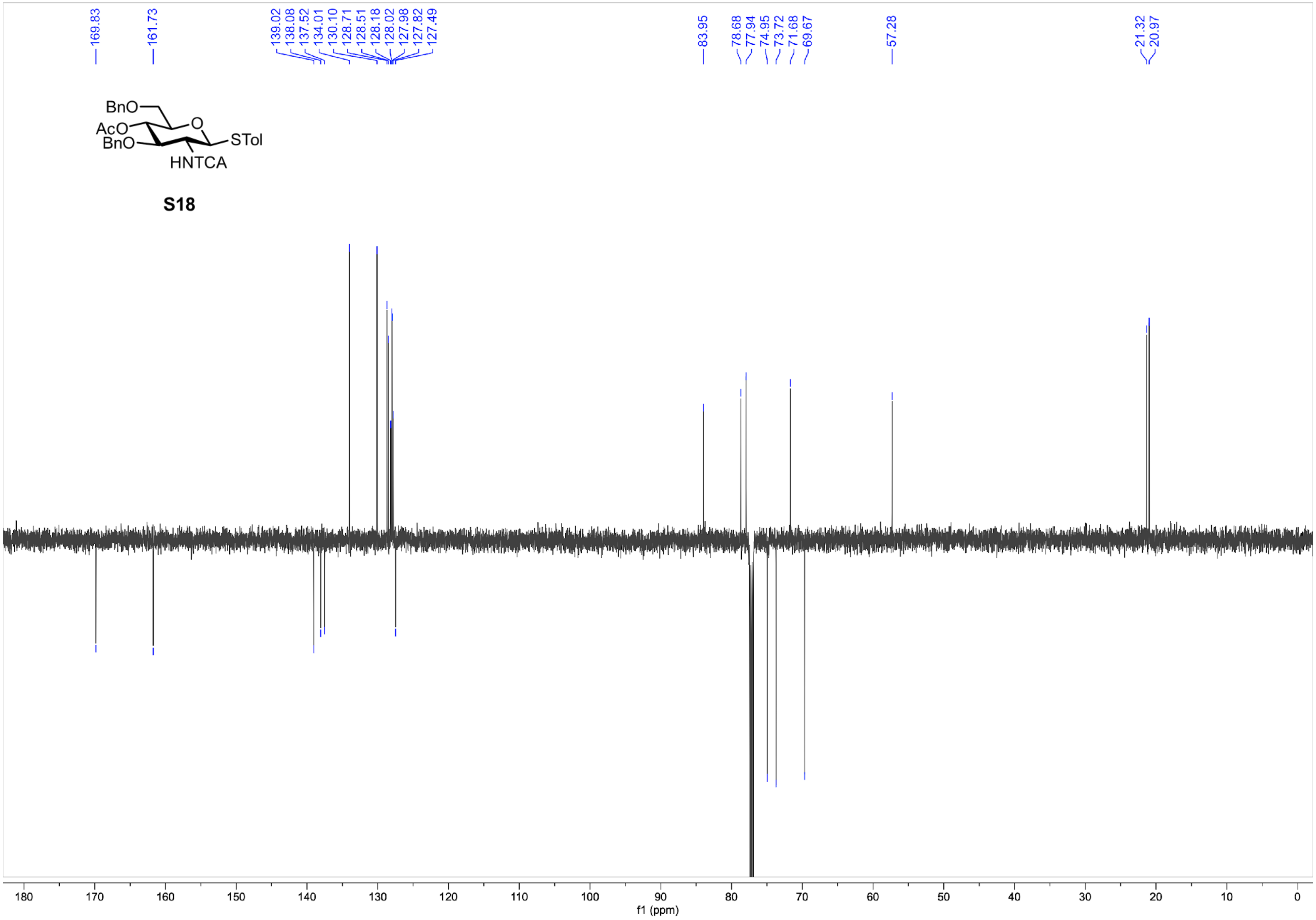

S18 HSQC spectrum

600 MHz for ^{1}H in $CDCl_3$, Pulse Sequence: hsqcedetgpsisp2.3, NS 4, NUS 25%

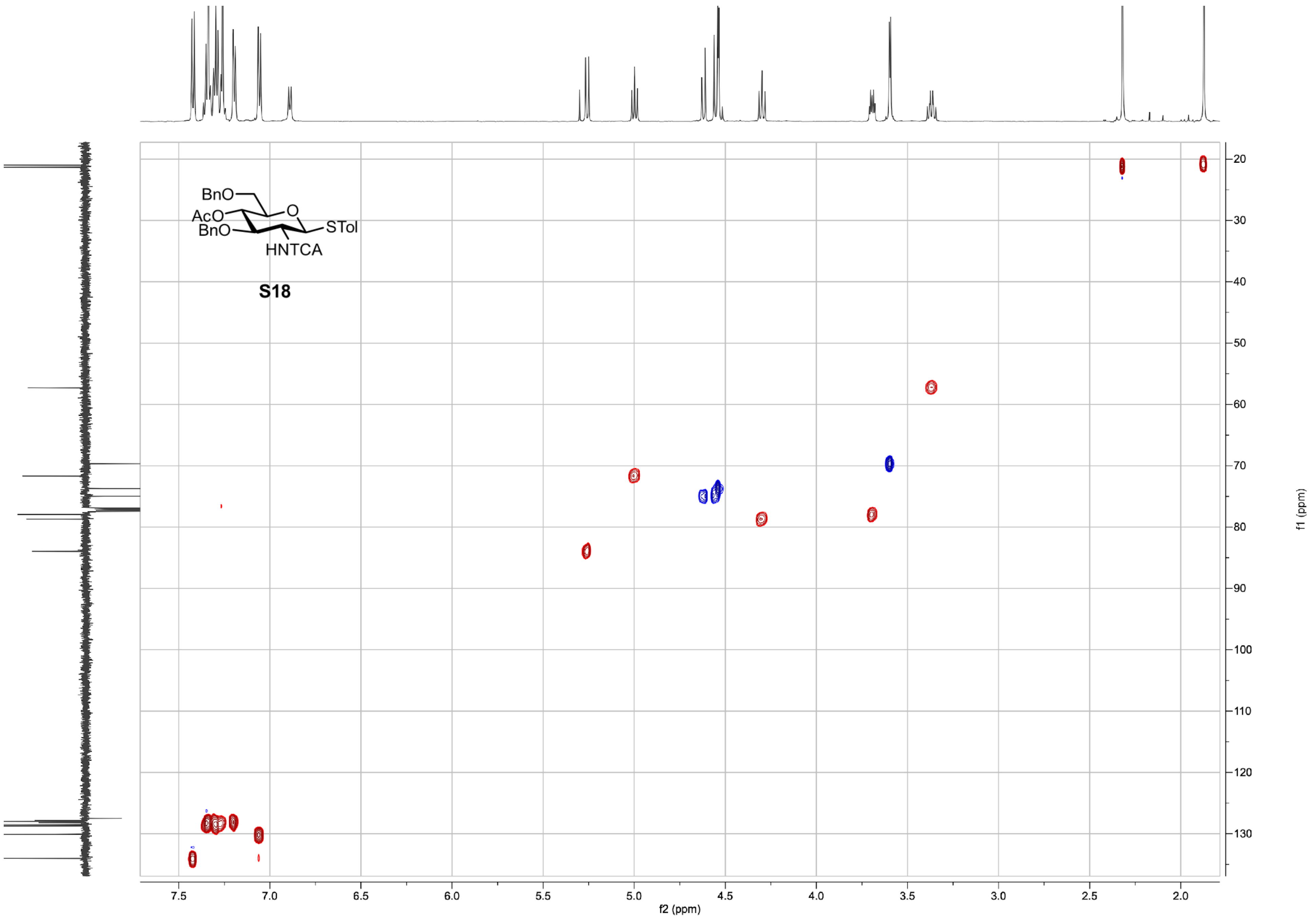

S19 *^{1}H spectrum*

600 MHz, $CDCl_3$

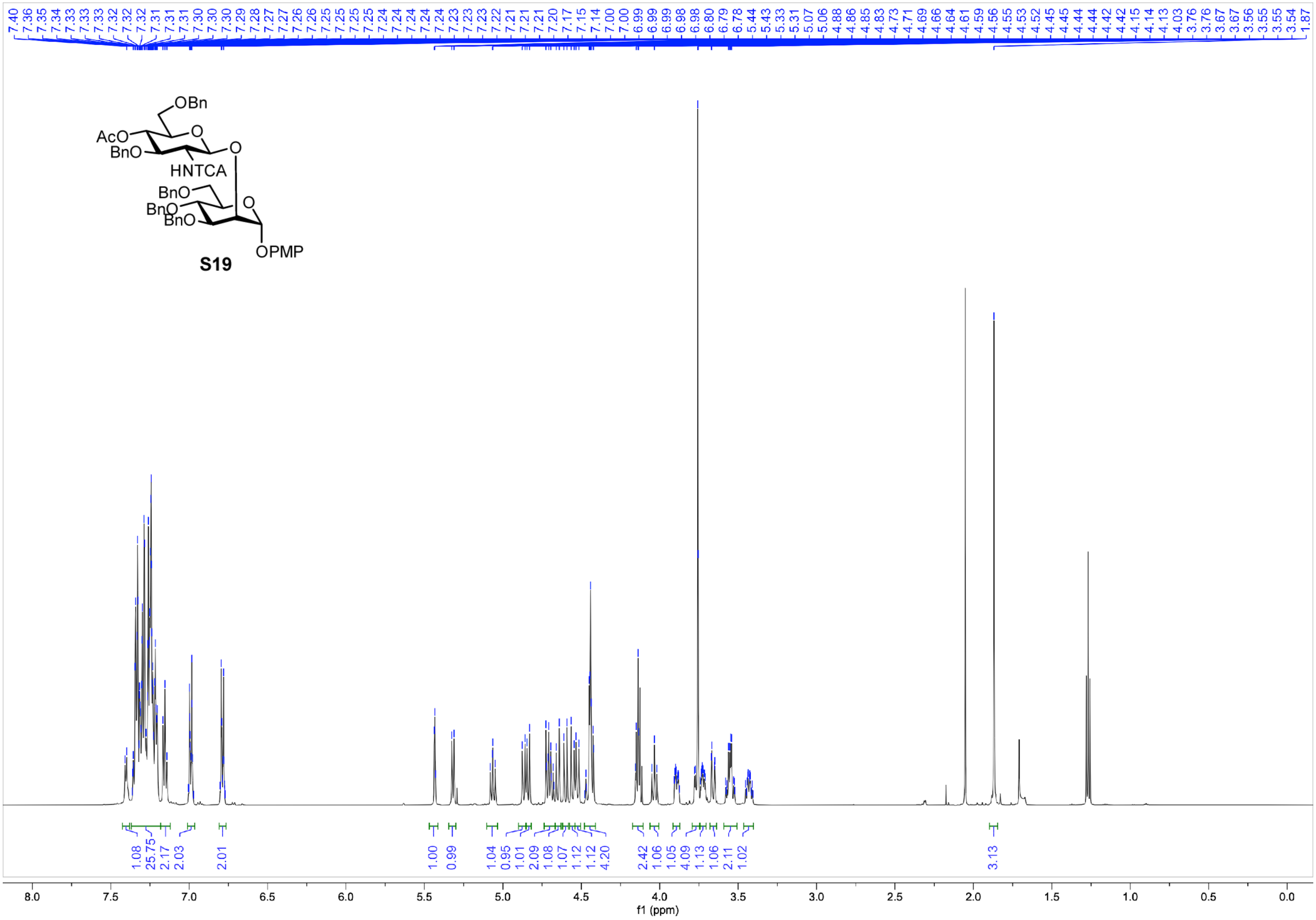

***S19** DEPTQ135 ^{13}C NMR spectrum*

151 MHz in $CDCl_3$, Pulse Sequence: deptqgpsp.2, NS 14

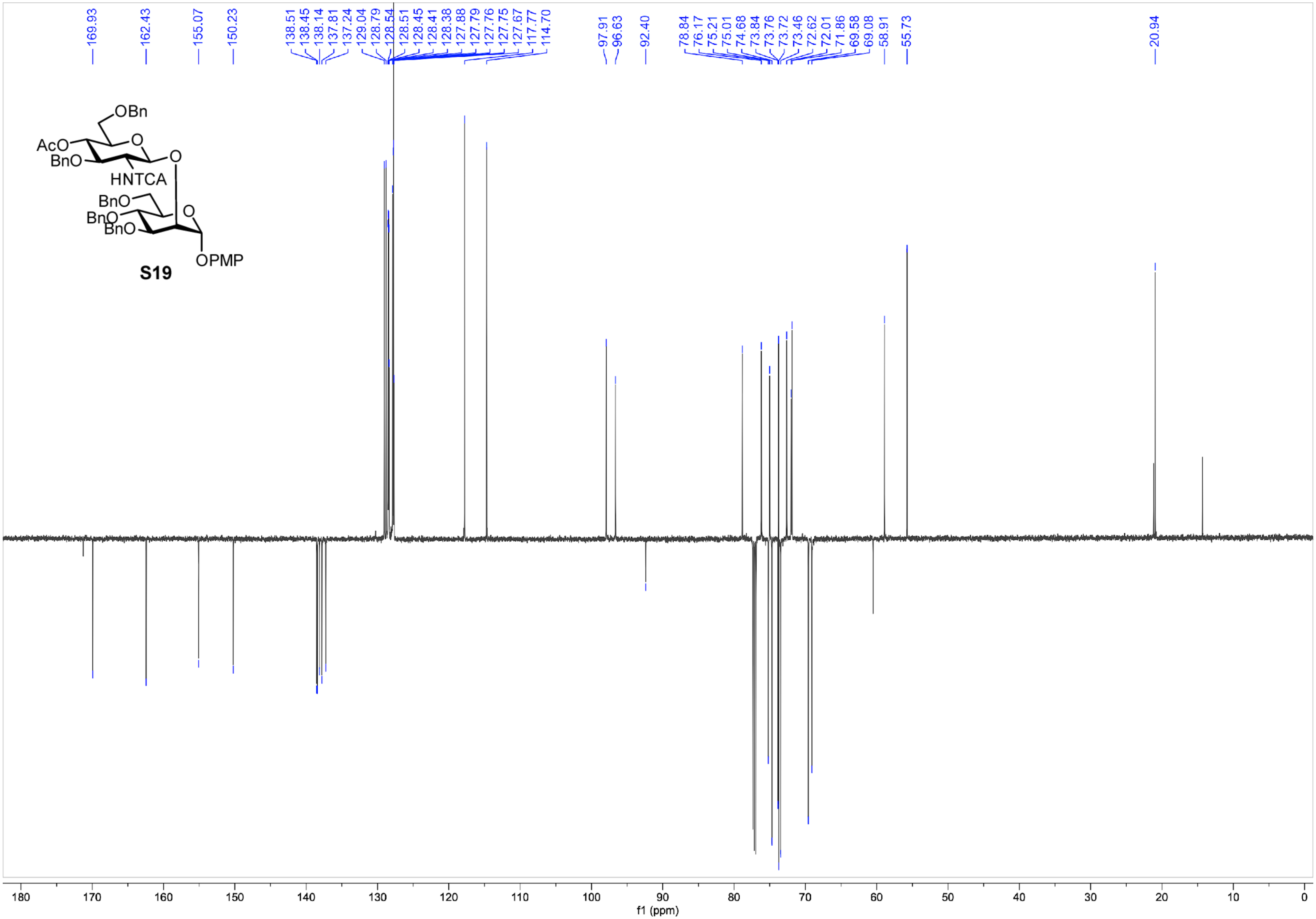

***S19** HSQC spectrum*

600 MHz for ¹H in $CDCl_3$, Pulse Sequence: hsqcedetgpsisp2.3, NS 2, NUS 25%

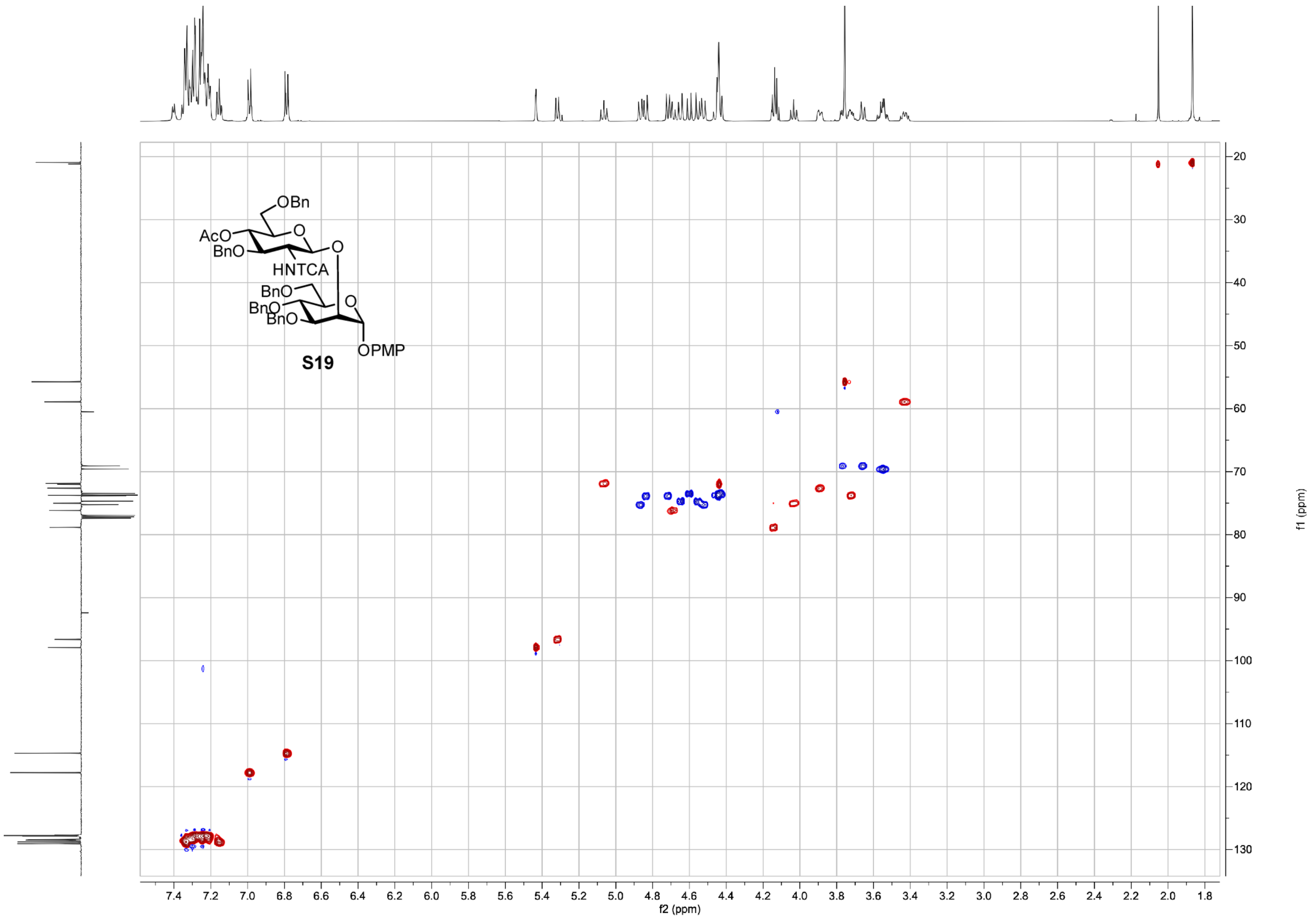

S21 *¹H spectrum*

600 MHz, $CDCl_3$

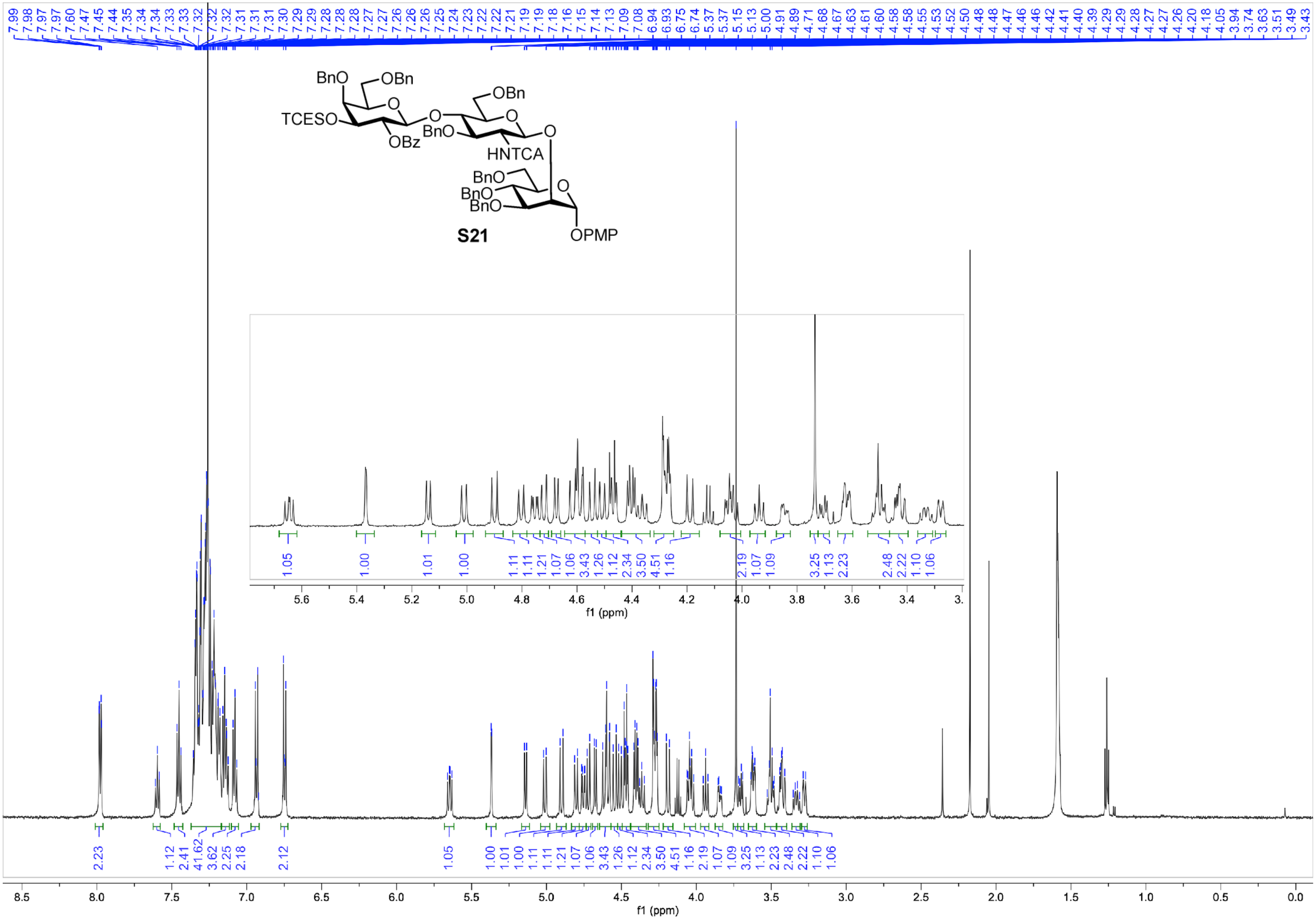

***S21** DEPTQ135 ^{13}C NMR spectrum*

151 MHz in $CDCl_3$, Pulse Sequence: deptqgpsp.2, NS 21, AV600, probe: DCH

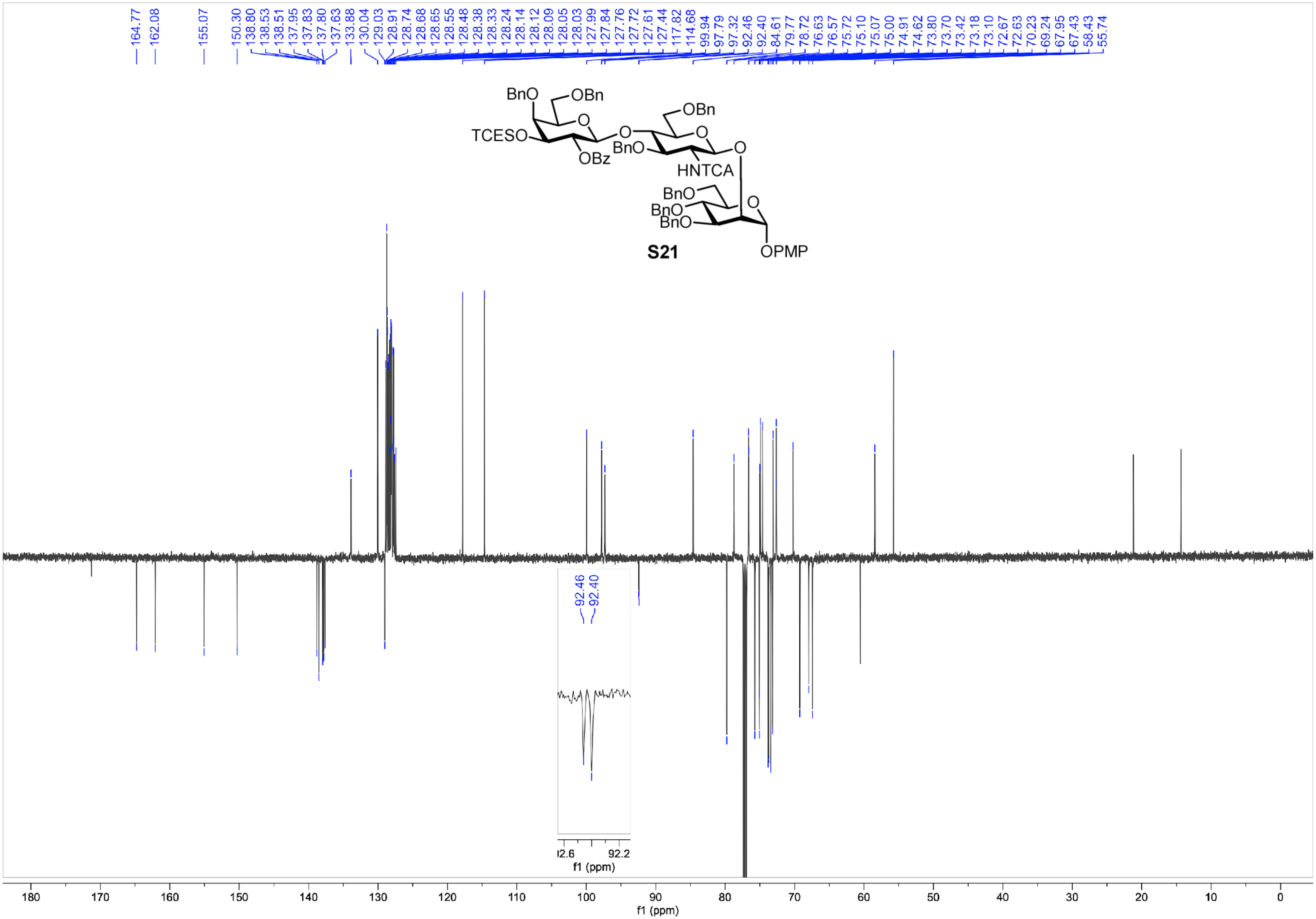

S21 *HSQC spectrum*

600 MHz for ^{1}H in $CDCl_3$, Pulse Sequence: hsqcedetgpsisp2.3, NS 2, NUS 25%, AV 600, probe: DCH

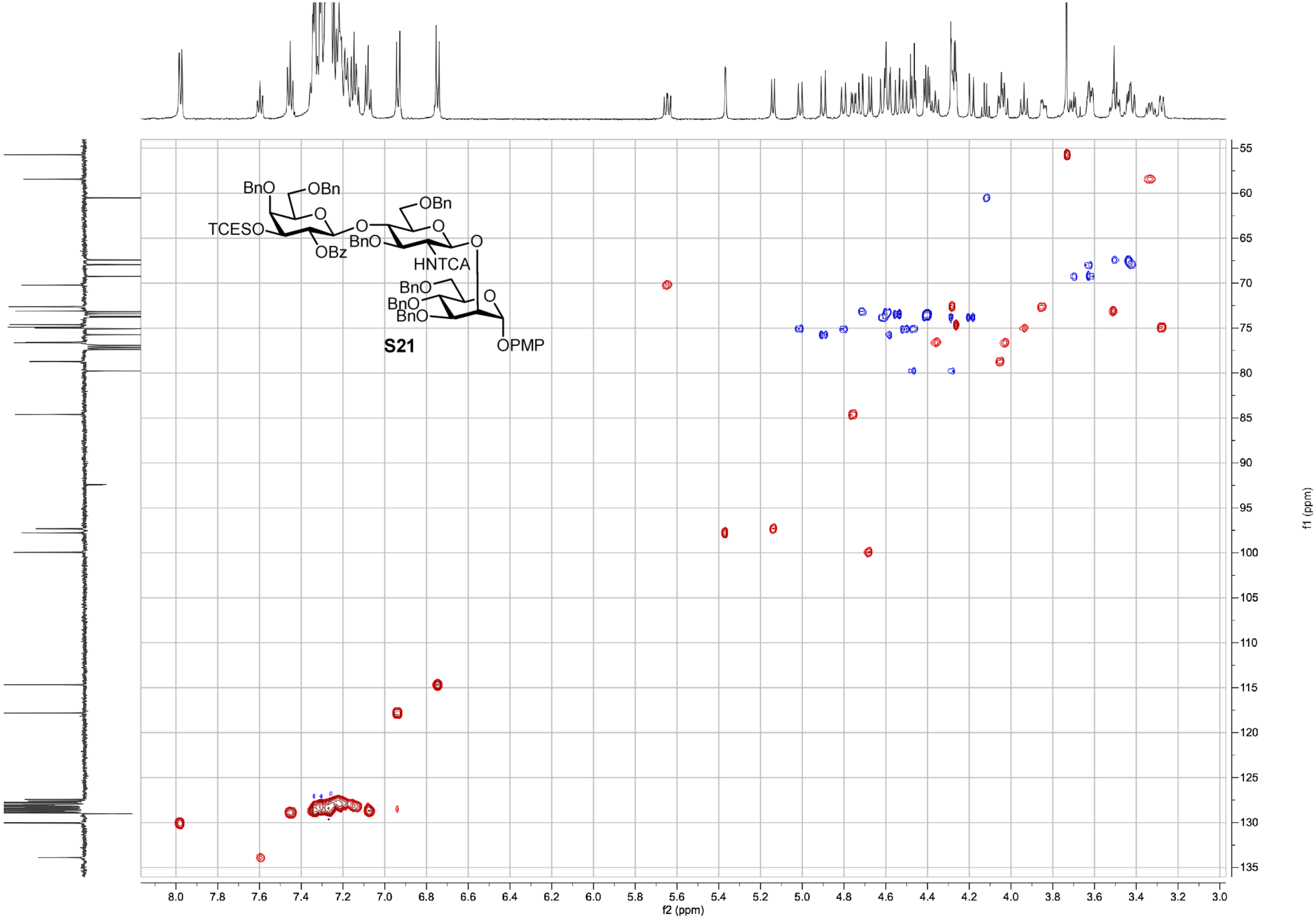

***19** ¹H spectrum*

600 MHz in D_2O

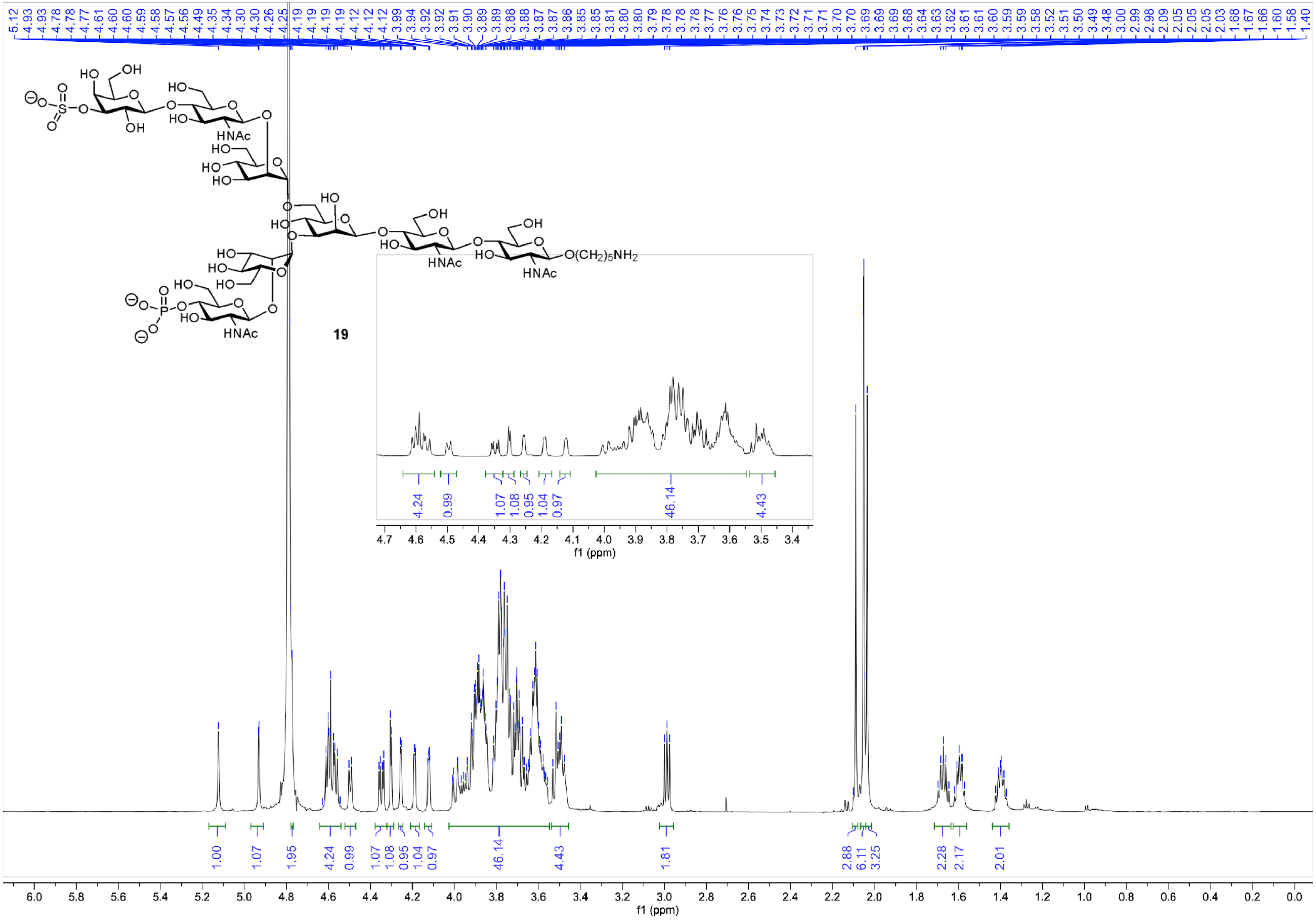

***19** DEPTQ135 ^{13}C NMR spectrum*

151 MHz in D_2O, Pulse Sequence: deptqgpsp.2, NS 432, AV 600, probe: QCI

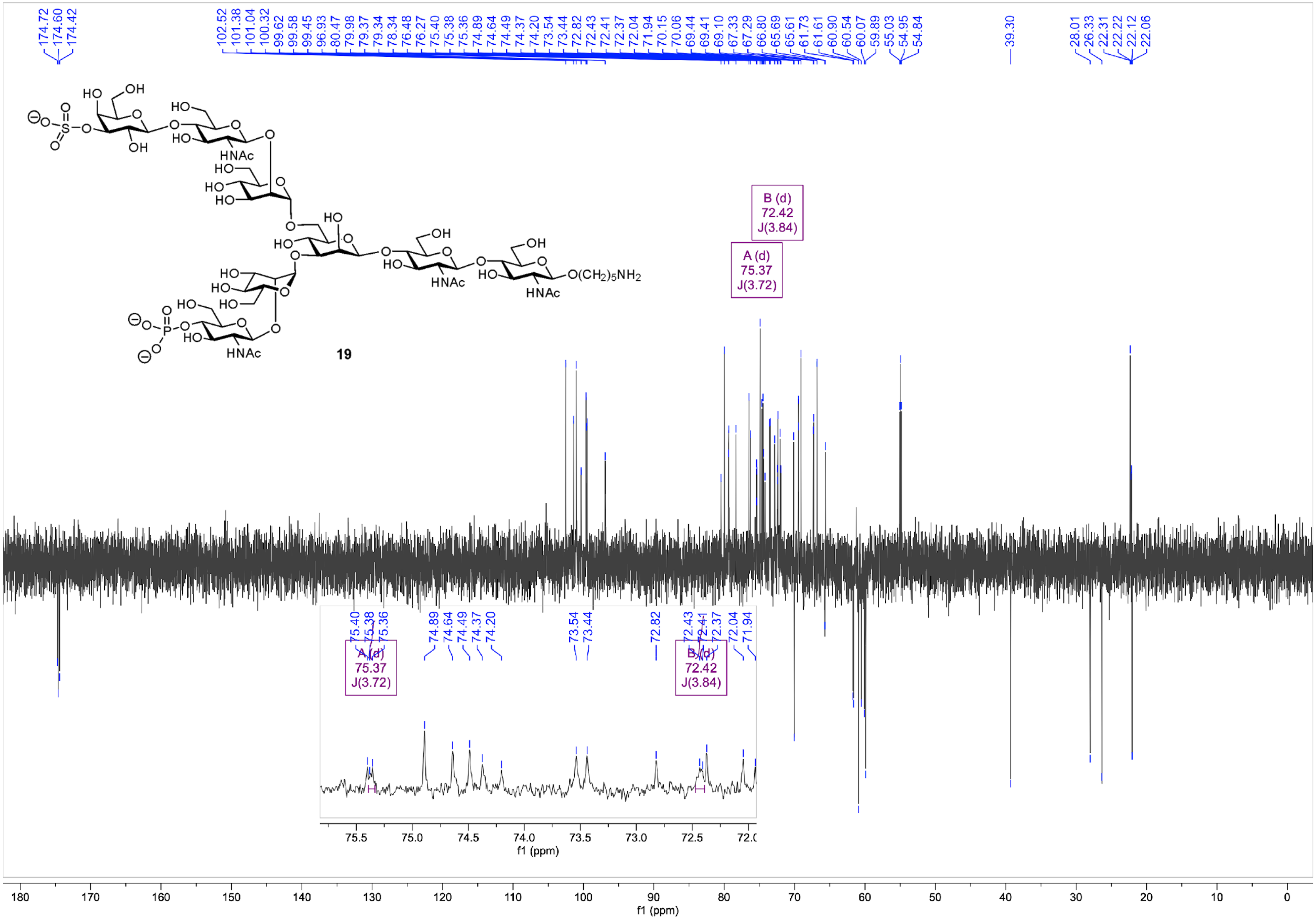

***19** HSQC spectrum*

600 MHz for ^{1}H in D_2O, Pulse Sequence: hsqcedetgpsisp2.2, NS 4, NUS 25%, AV 600, probe: DCH

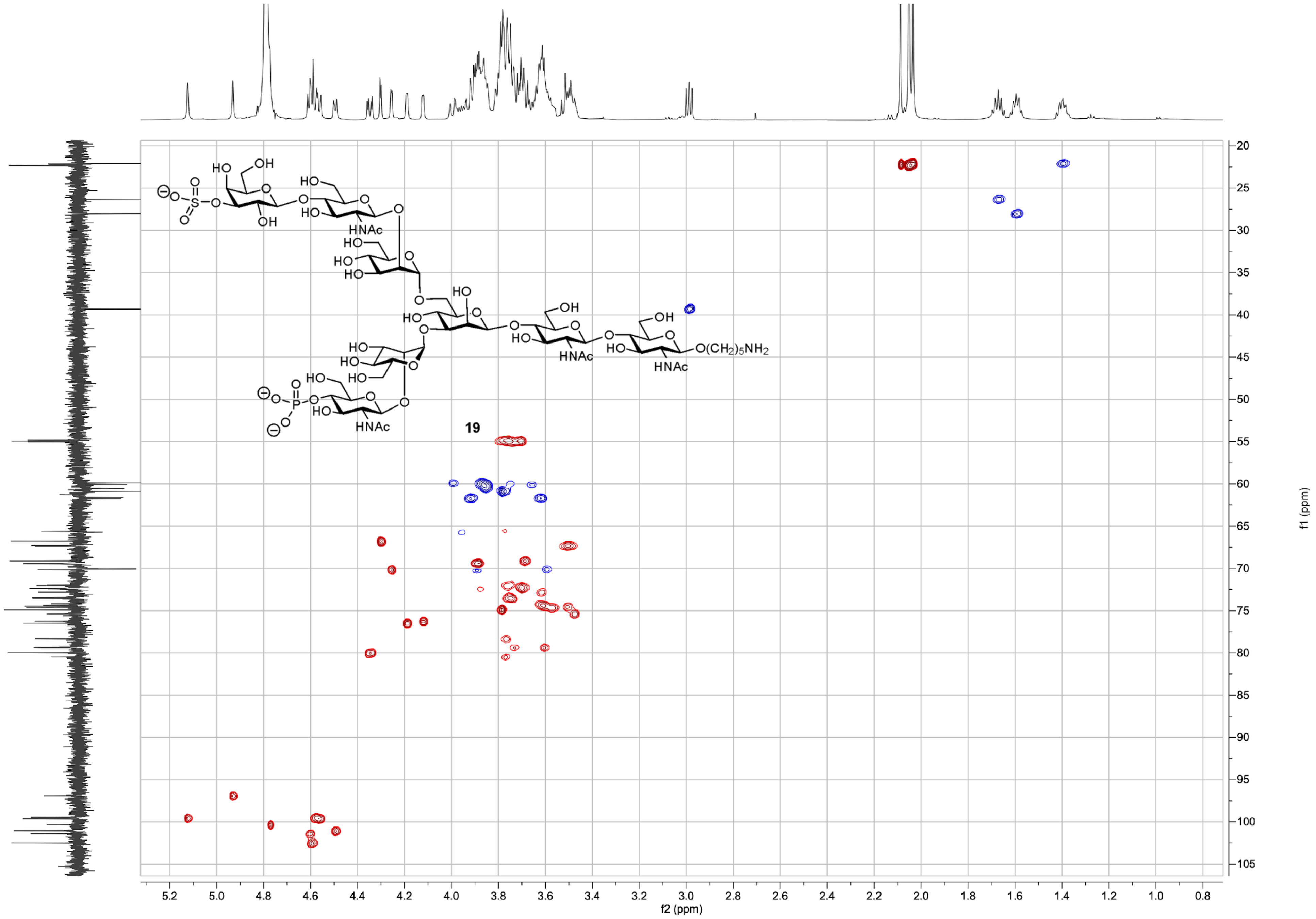

S25 *^{1}H spectrum*

600 MHz, $CDCl_3$

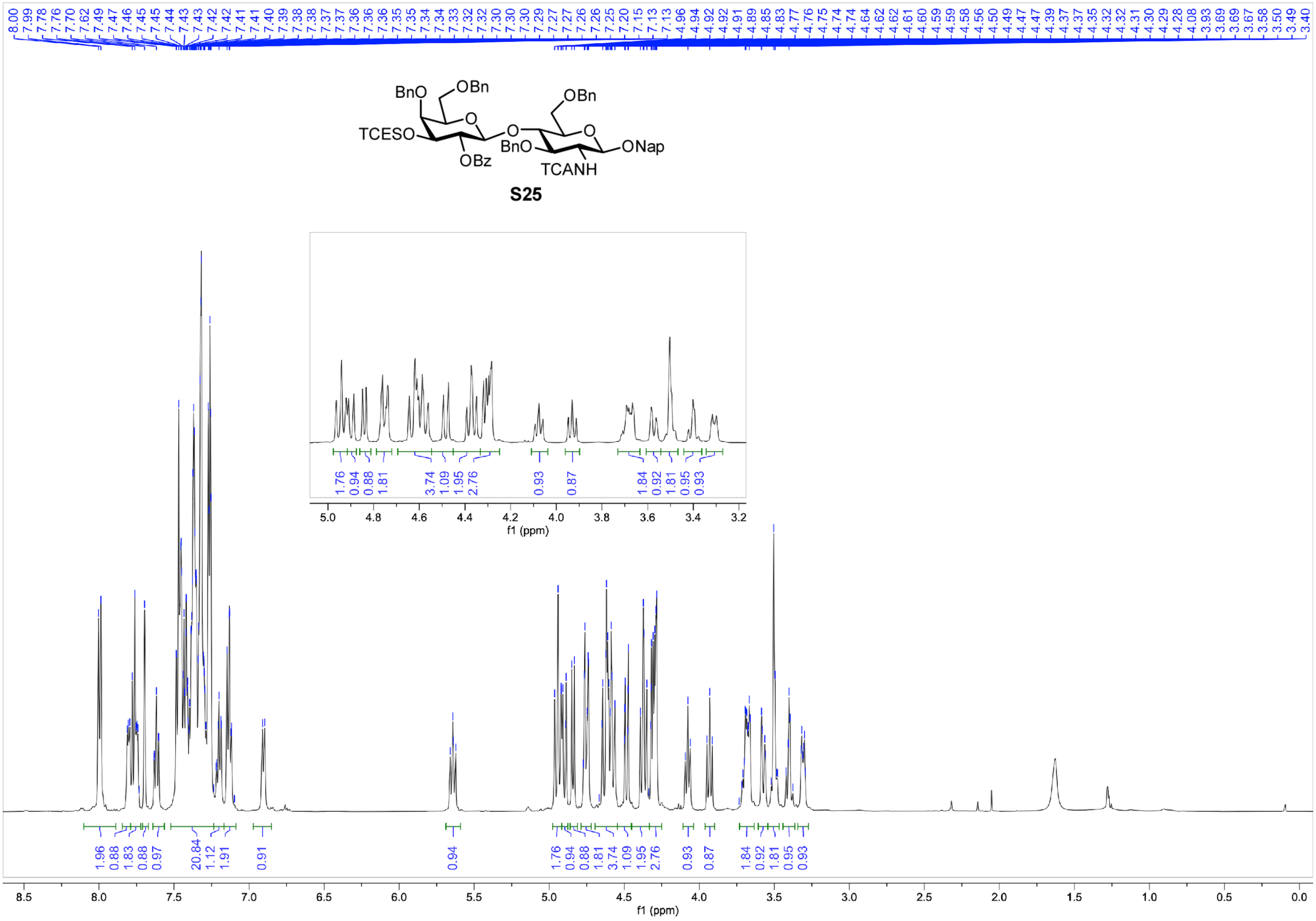

S25 *^{13}C NMR spectrum*

126 MHz in $CDCl_3$, Pulse Sequence: zgpg30 NS 256, AV 500, probe BBO

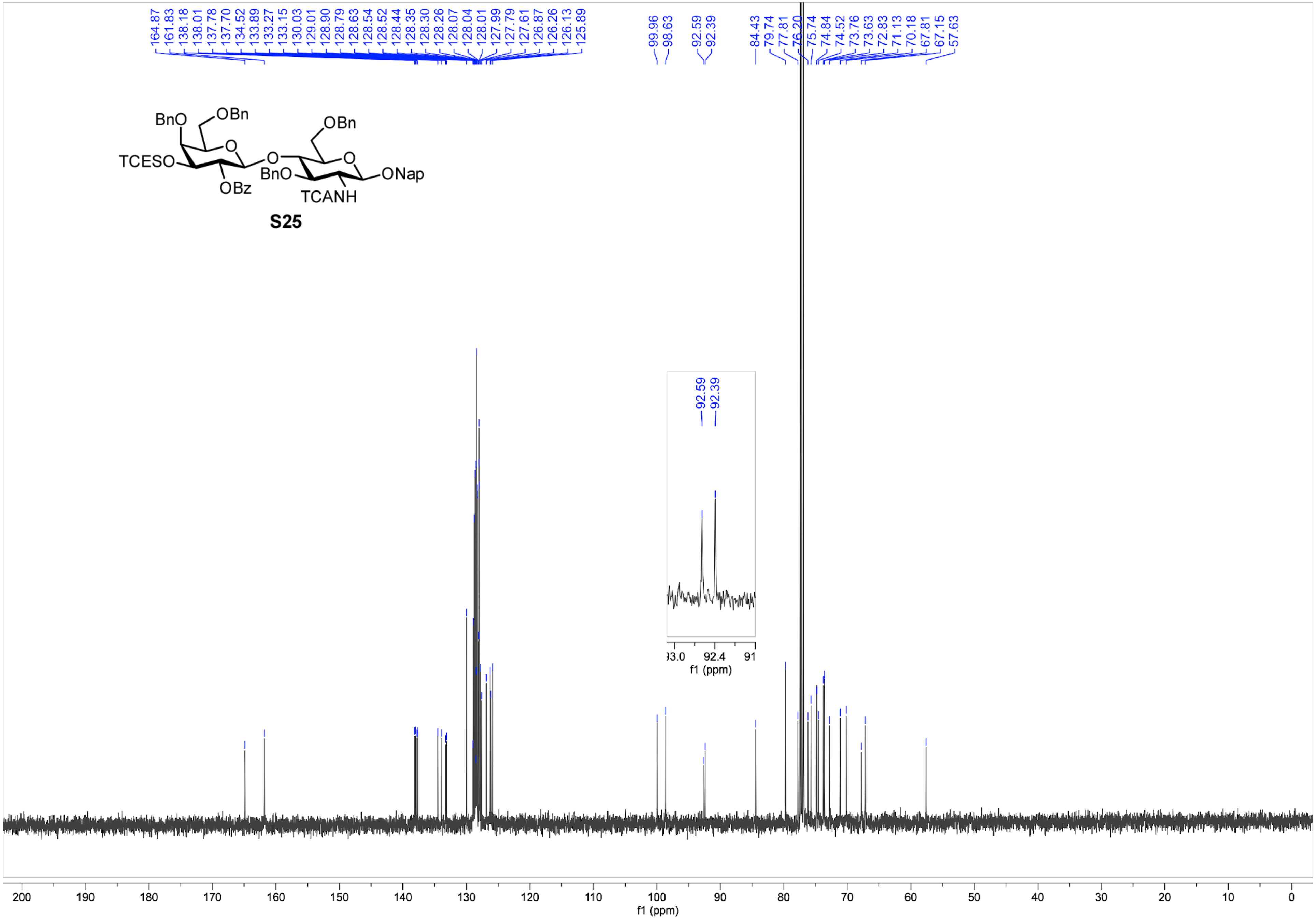

***S25** HSQC spectrum*

500 MHz for ^{1}H in $CDCl_3$, Pulse Sequence: hsqcedetgpsisp2.3, NS 2, NUS 25, AV 500, probe BBO

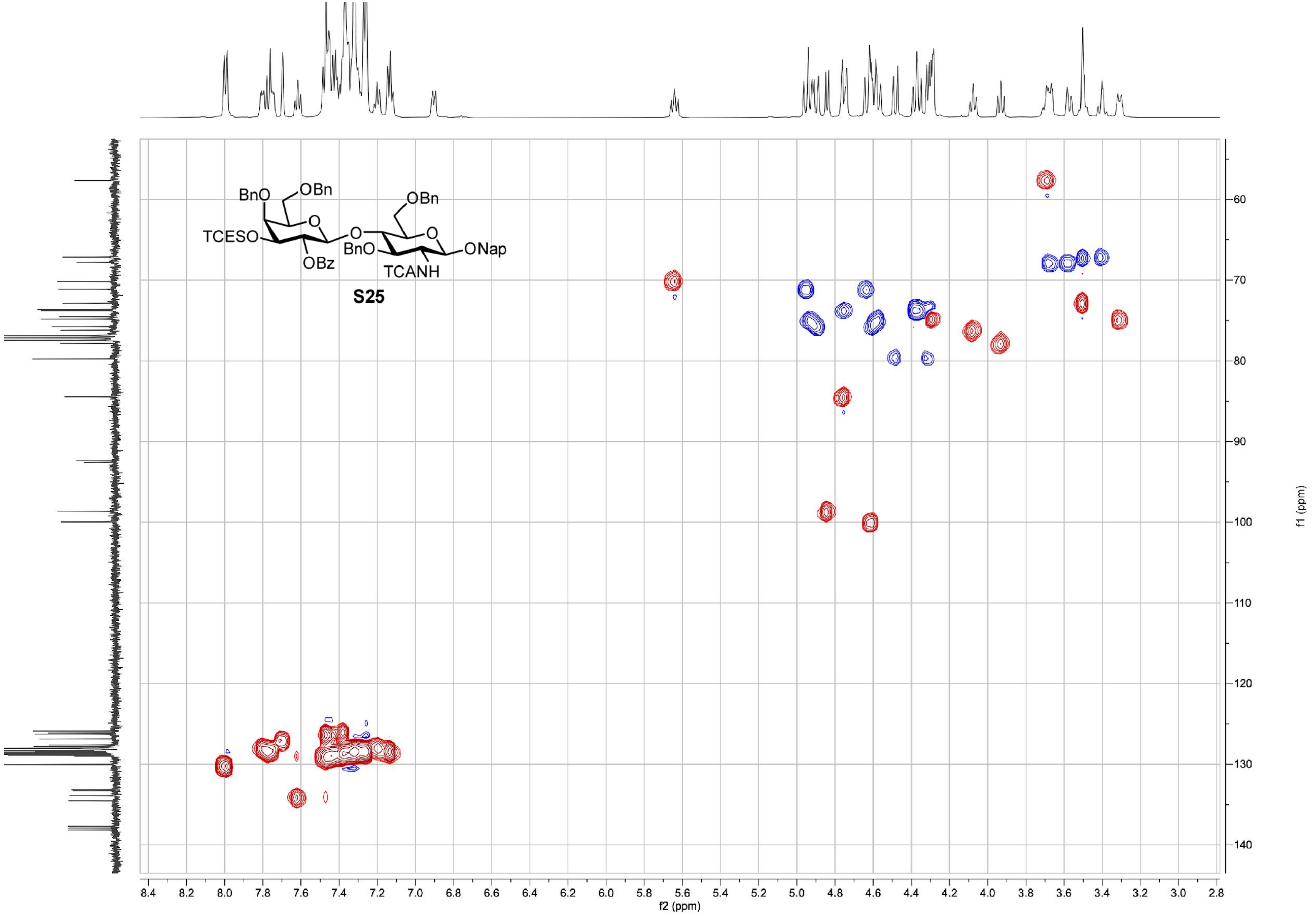

S26 *^{1}H spectrum*

600 MHz, $CDCl_3$

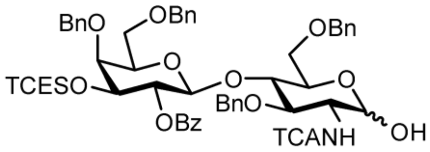

S26

***S26** DEPTQ135 ^13C NMR spectrum*

126 MHz in $CDCl_3$, Pulse Sequence: deptqgpsp, NS 256, AV 500, BBO

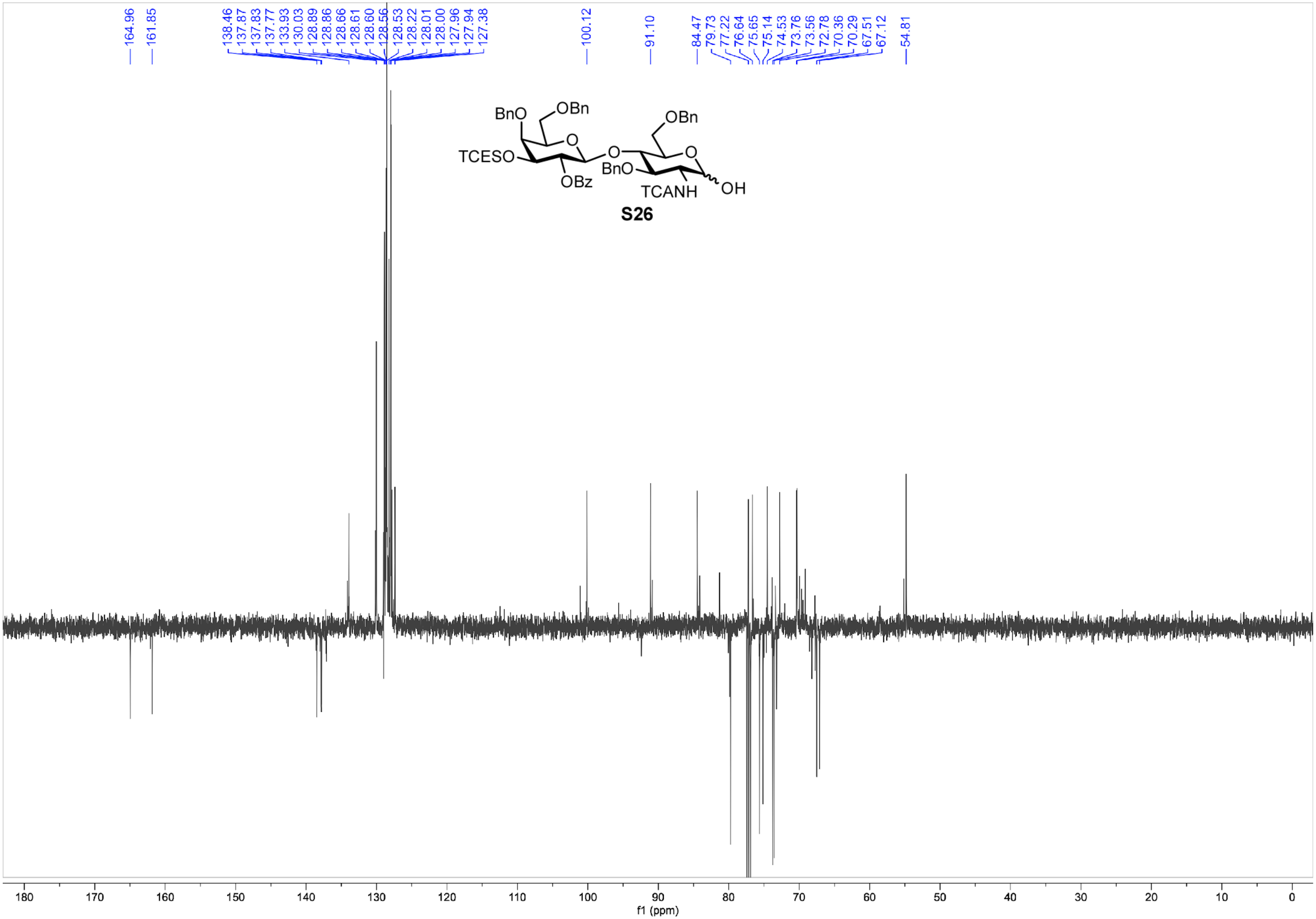

***S26** HSQC spectrum*

500 MHz for ^{1}H in $CDCl_3$, Pulse Sequence: hsqcedetgpsisp2.3, NS 2, NUS 25%, AV 500, BBO probe

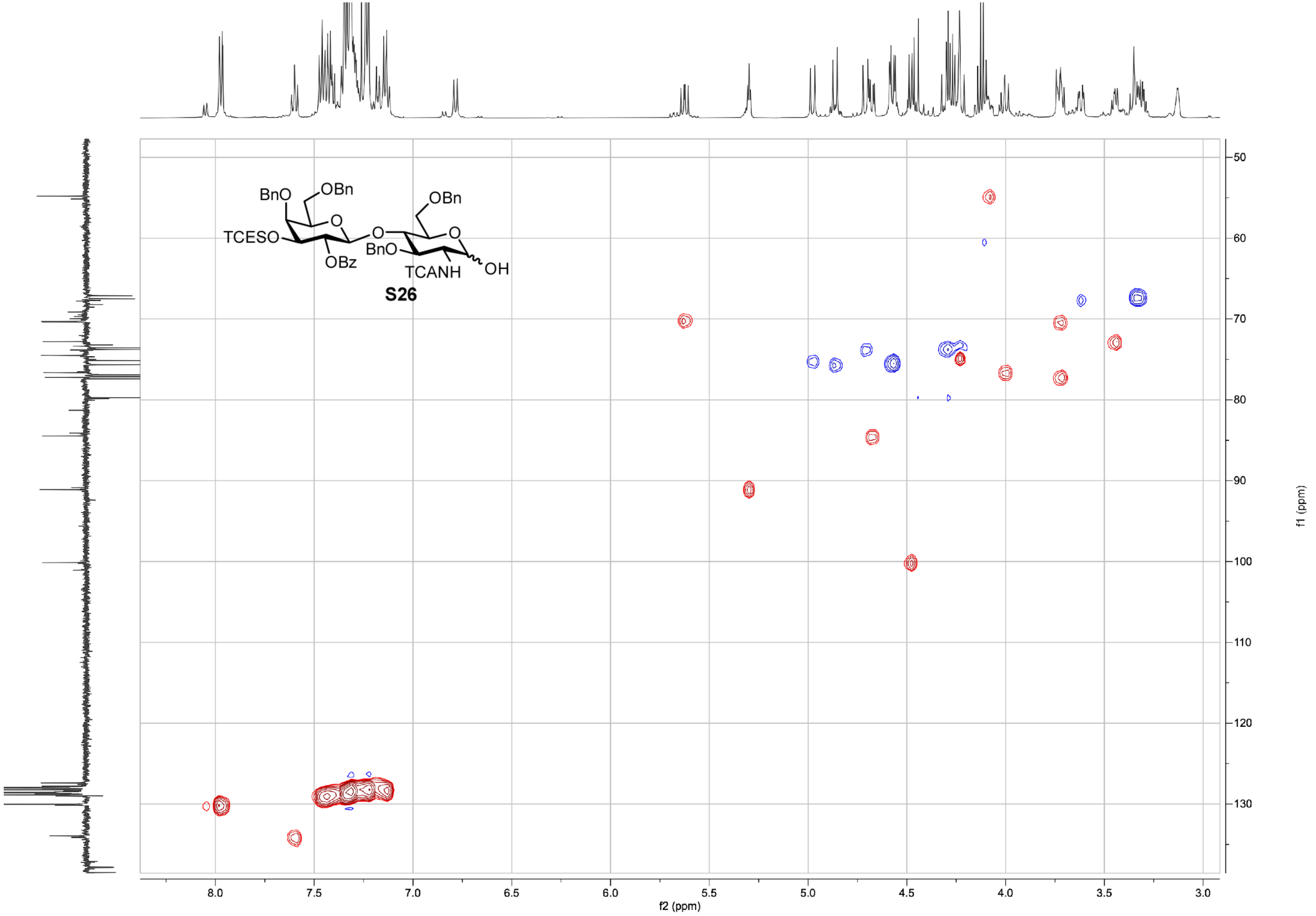

9b *^{1}H spectrum*

600 MHz, $CDCl_3$

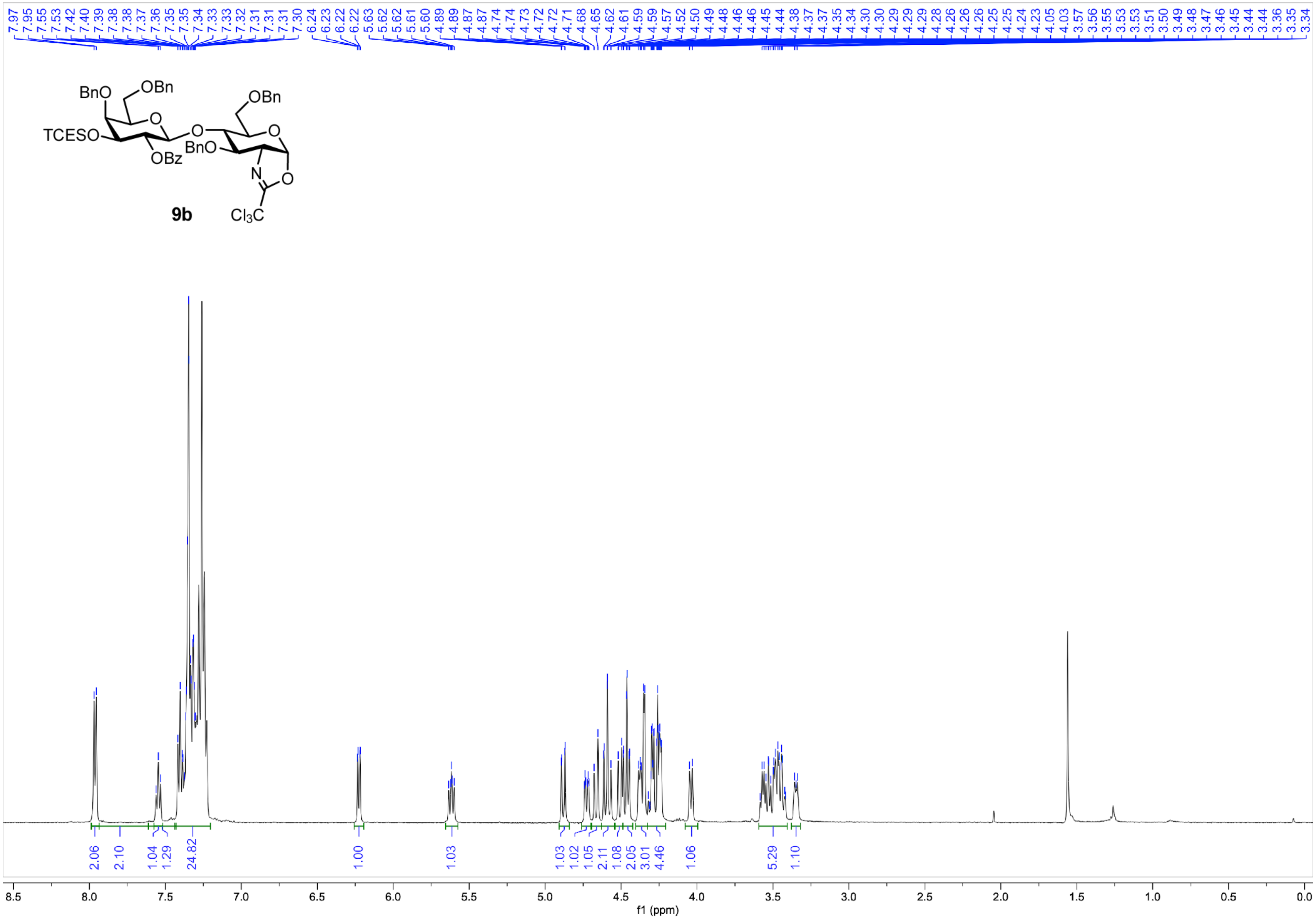

***9b** DEPTQ135 ^{13}C NMR spectrum*

126 MHz in $CDCl_3$, Pulse Sequence: deptqgpsp, NS 128, AV 500 (BBO)

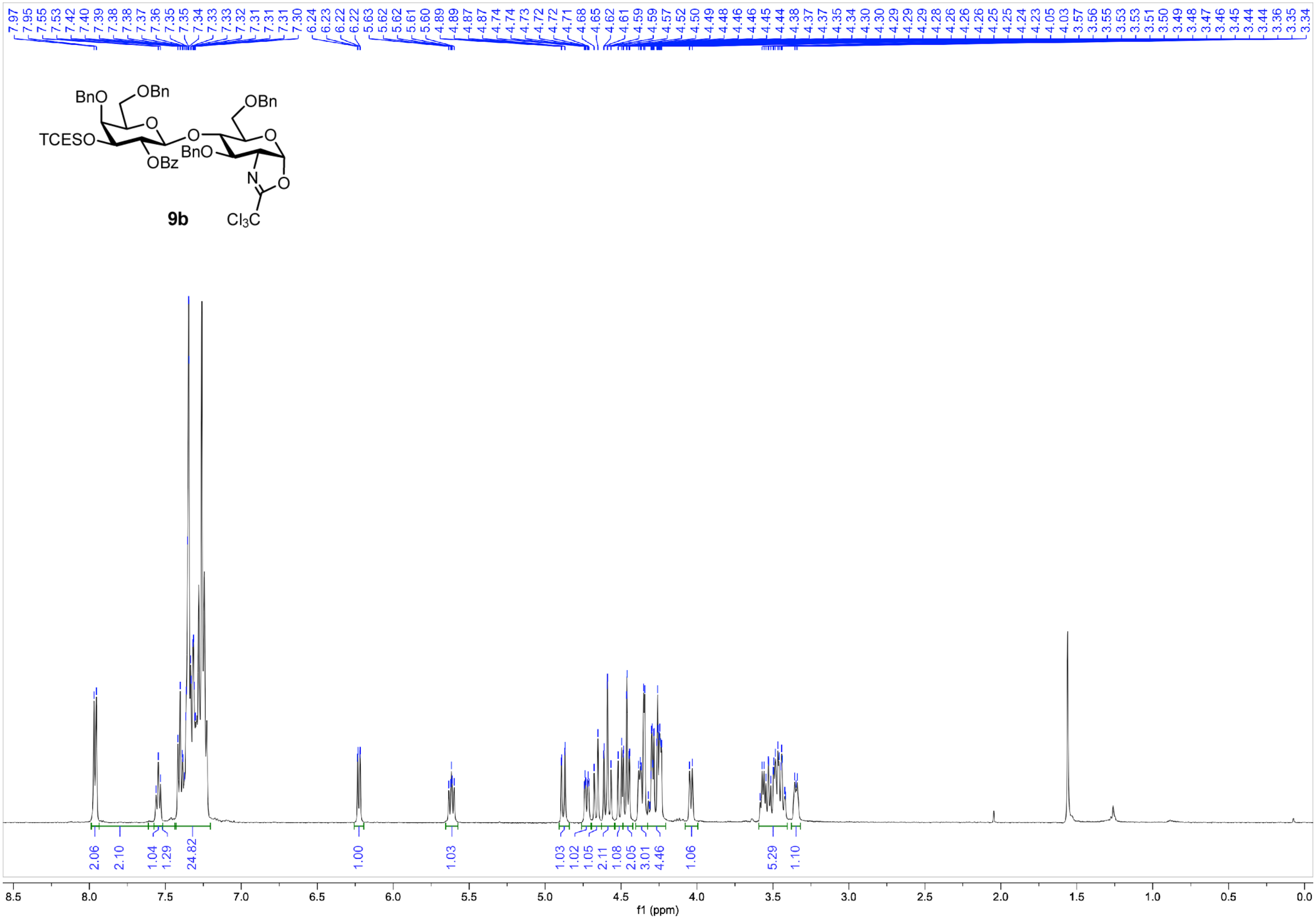

***9b** HSQC spectrum*

500 MHz for ^{1}H in $CDCl_3$, Pulse Sequence: hsqcedetgpsisp2.3, NS 8, NUS 25%, AV 500, BBO probe

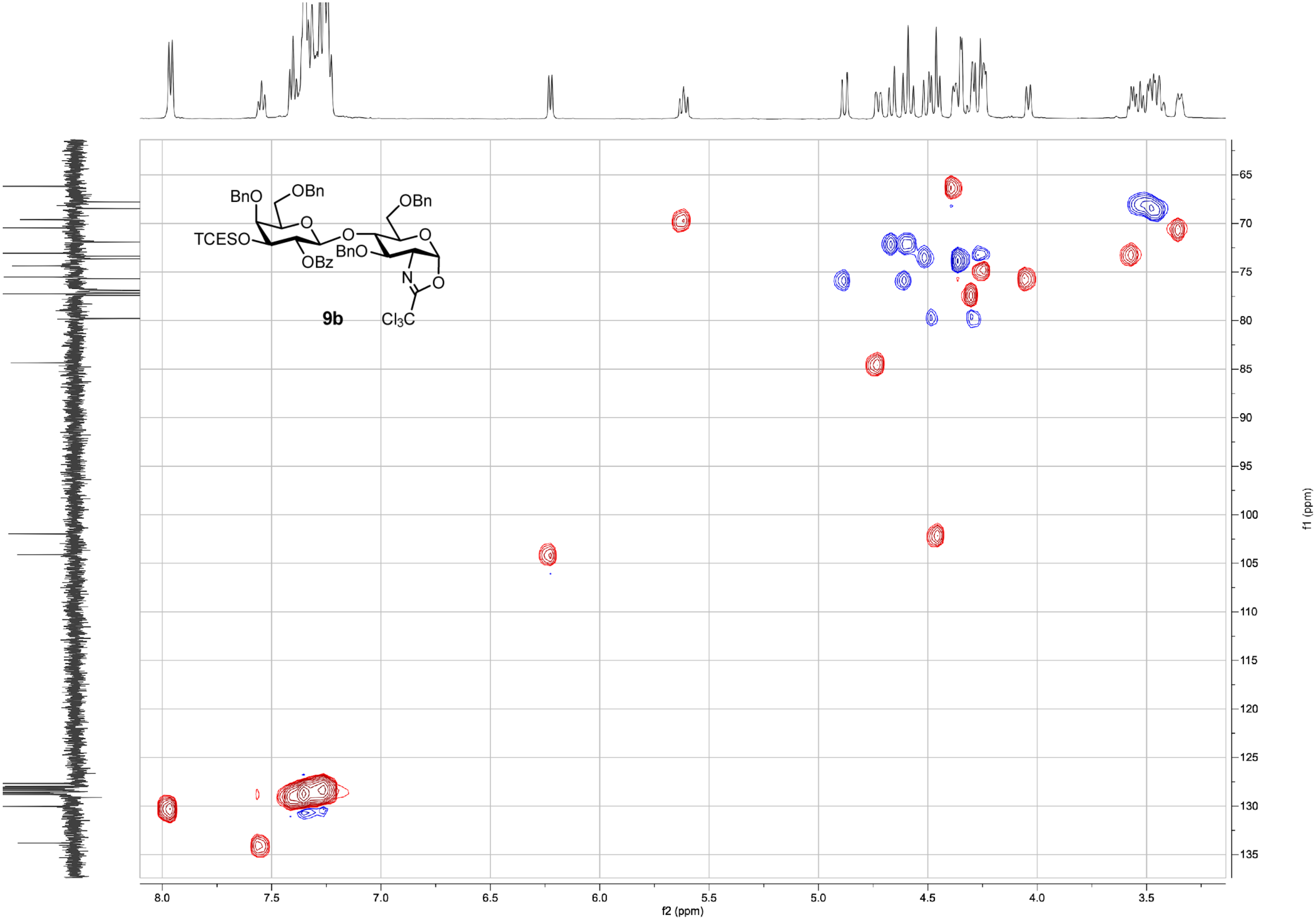

S2 *^{1}H spectrum*

500 MHz, $CDCl_3$

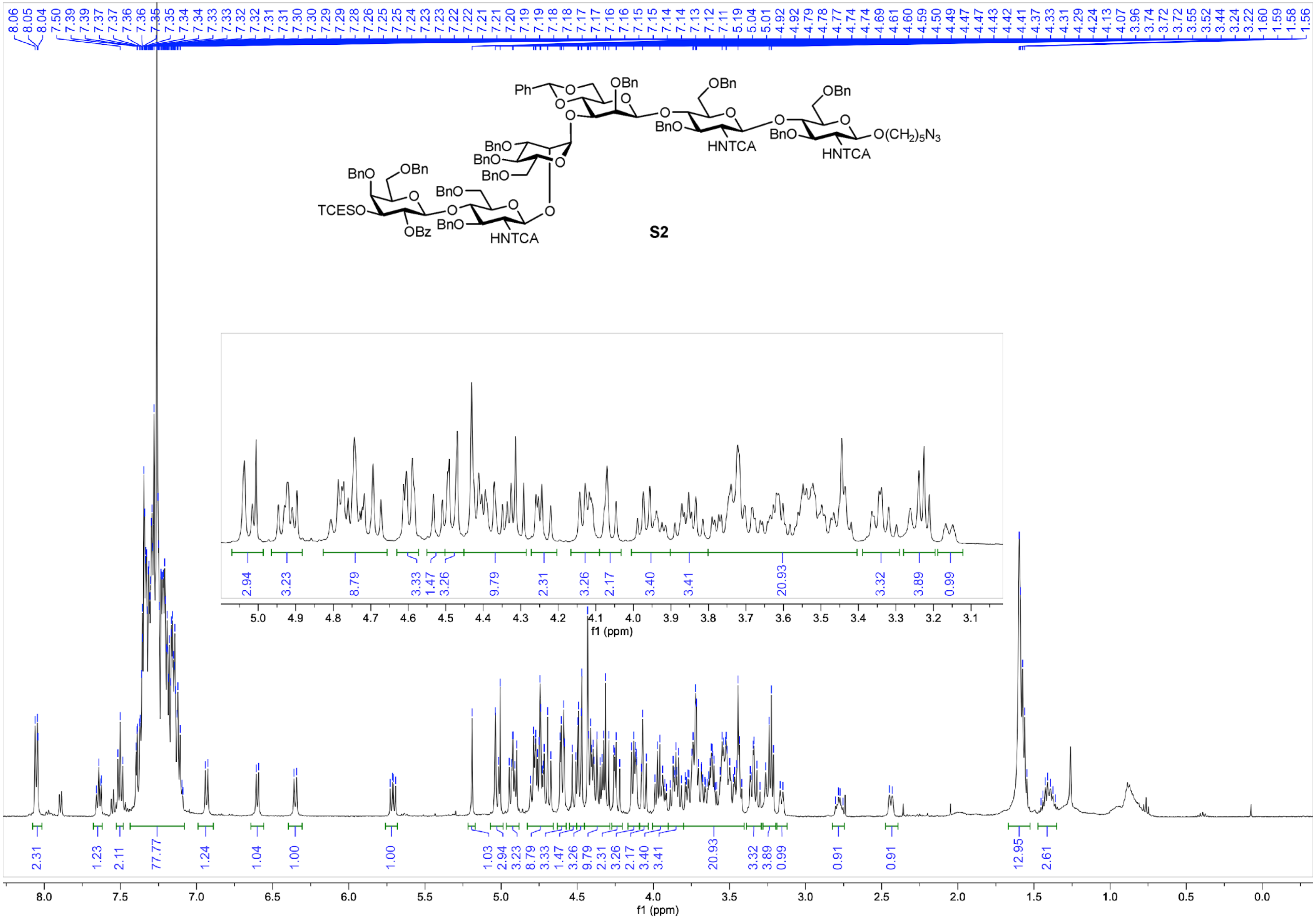

***S2** HSQC spectrum*

500 MHz for ^{1}H in $CDCl_3$, Pulse Sequence: hsqcedetgpsisp2.3, NS 4, NUS 25%, AV 500, BBO probe

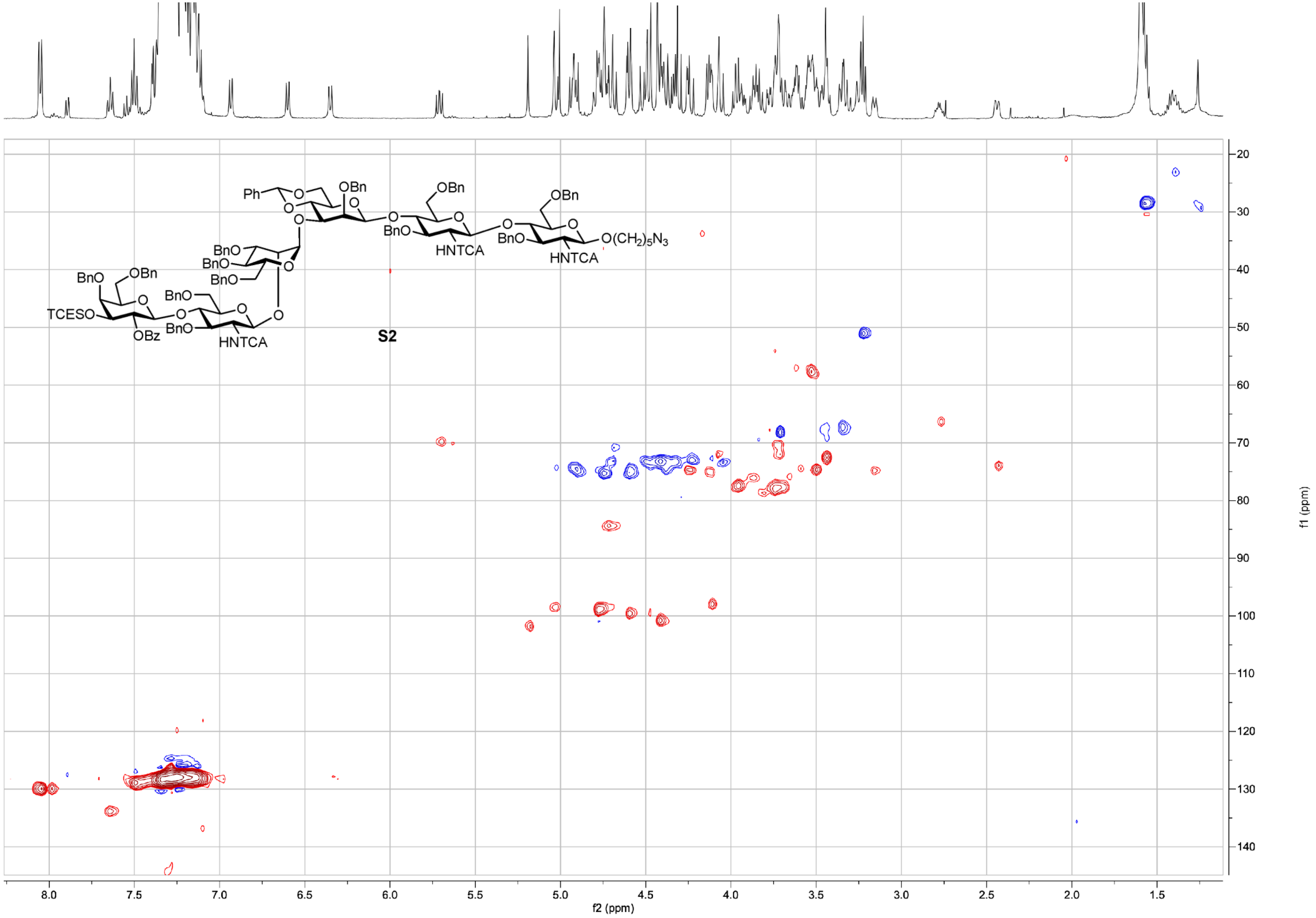

15 *^{1}H spectrum*

500 MHz, $CDCl_3$

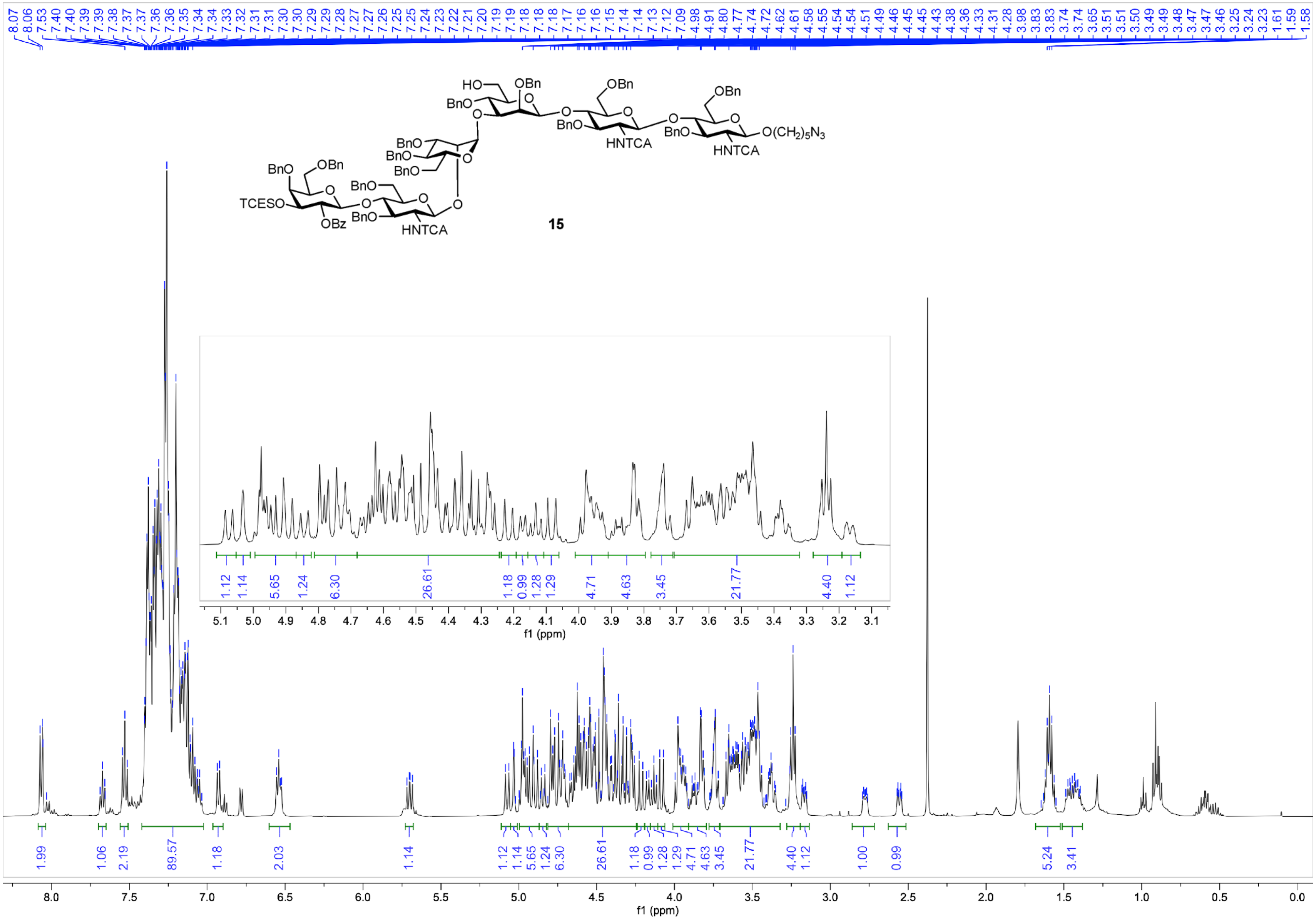

***15** HSQC spectrum*

500 MHz for ^{1}H in $CDCl_3$, Pulse Sequence: hsqcedetgpsisp2.3, NS 4, NUS 25%, AV 500, BBO probe

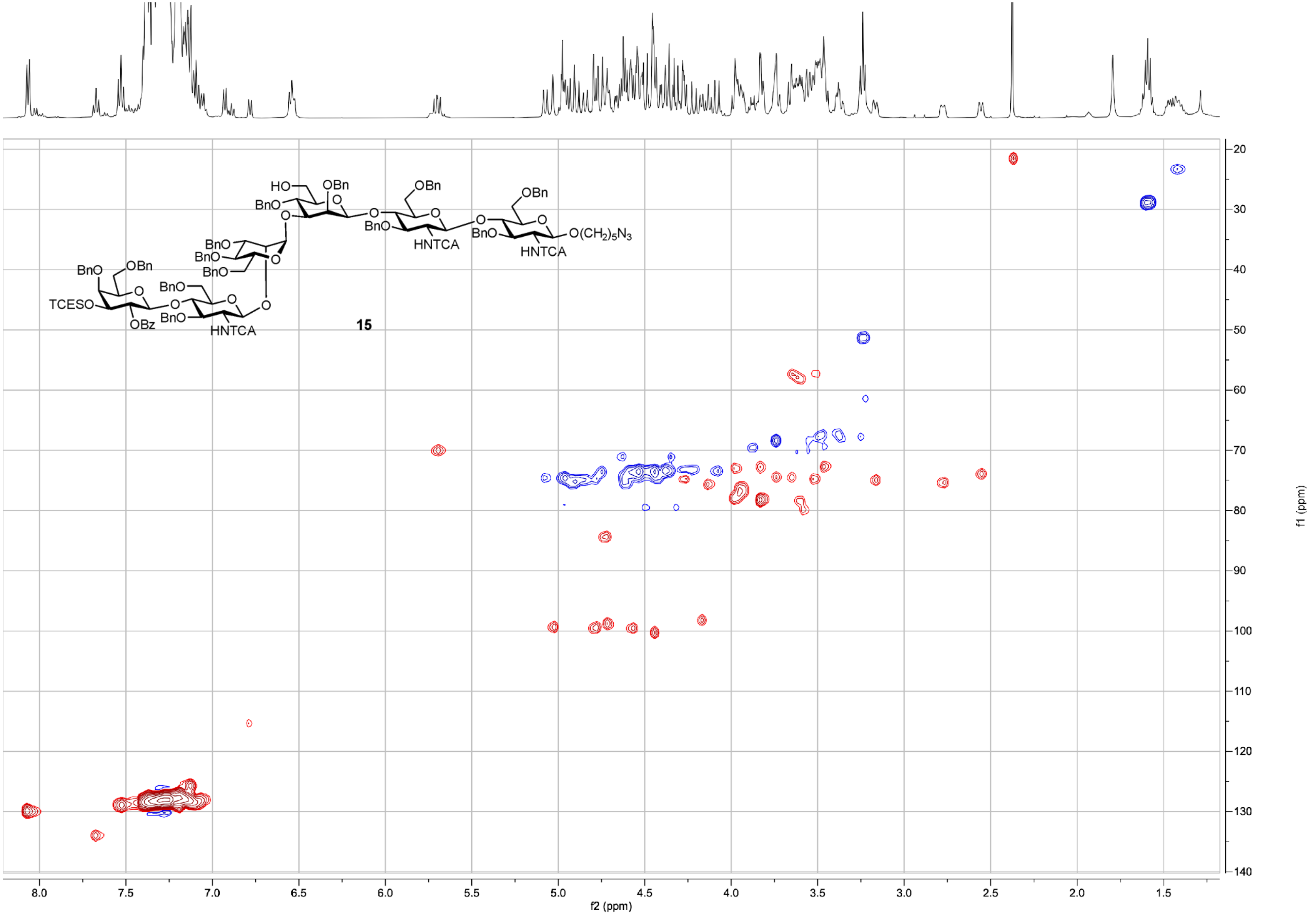

S27 *^{1}H spectrum*

700 MHz, $CDCl_3$

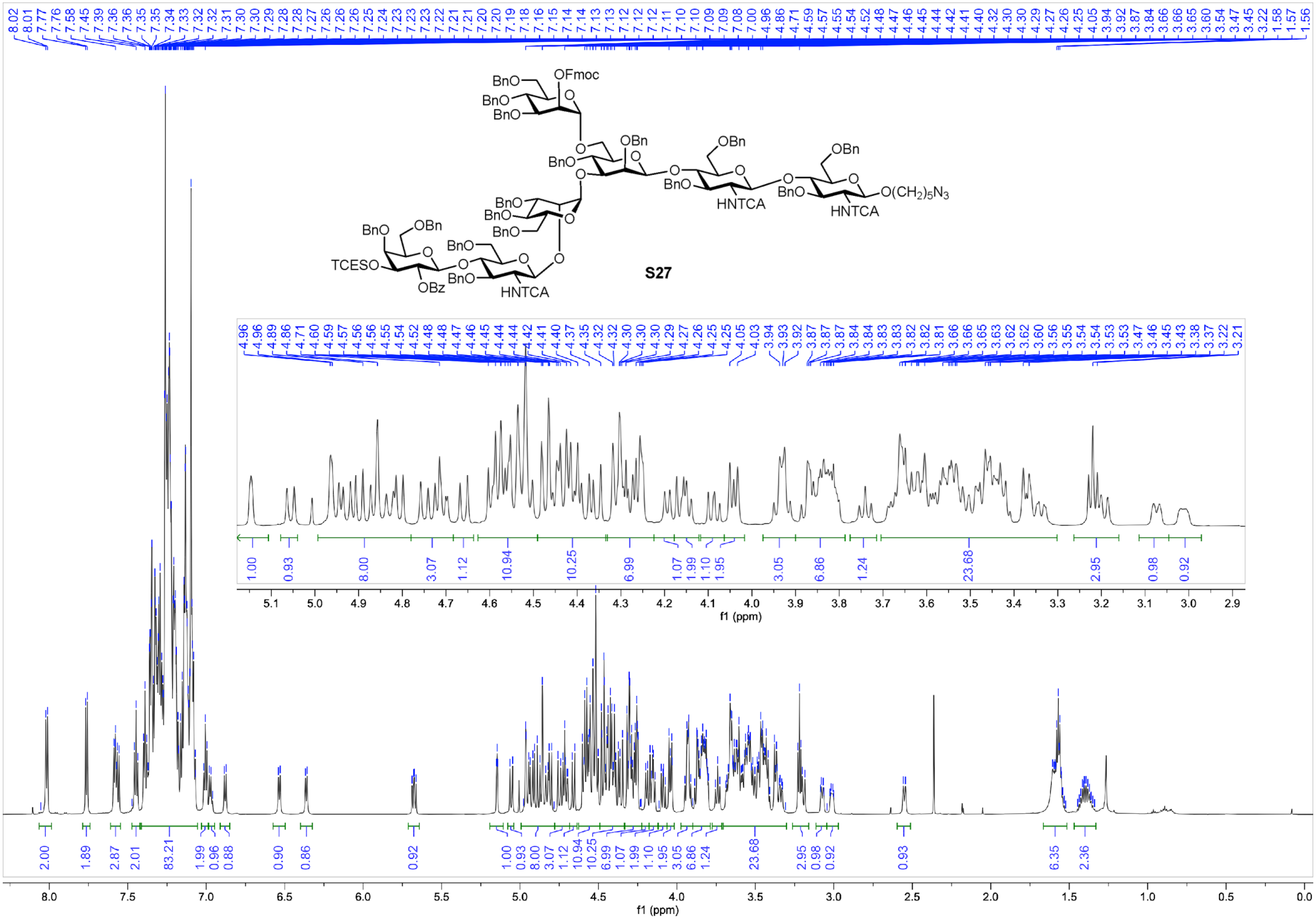

***S27** DEPTQ135 ^{13}C NMR spectrum*

177 MHz in $CDCl_3$, Pulse Sequence: deptqgpsp.2, NS 512, AV 700, probe CPTCI

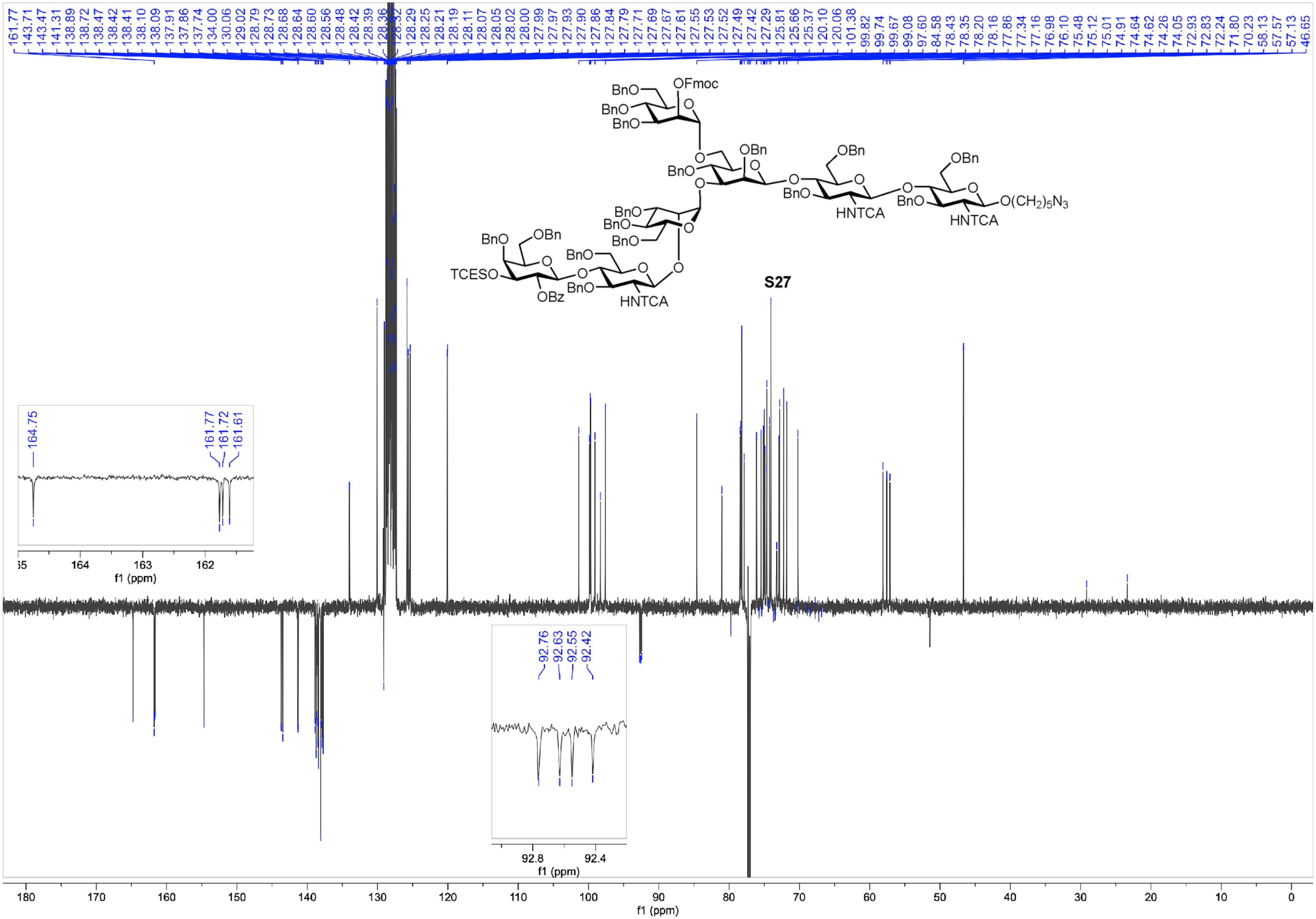

S27 *HSQC spectrum*

700 MHz for ^{1}H in $CDCl_3$, Pulse Sequence: hsqcedetgpsisp2.2, NS 4, NUS 25%, AV 700, probe CPTCI

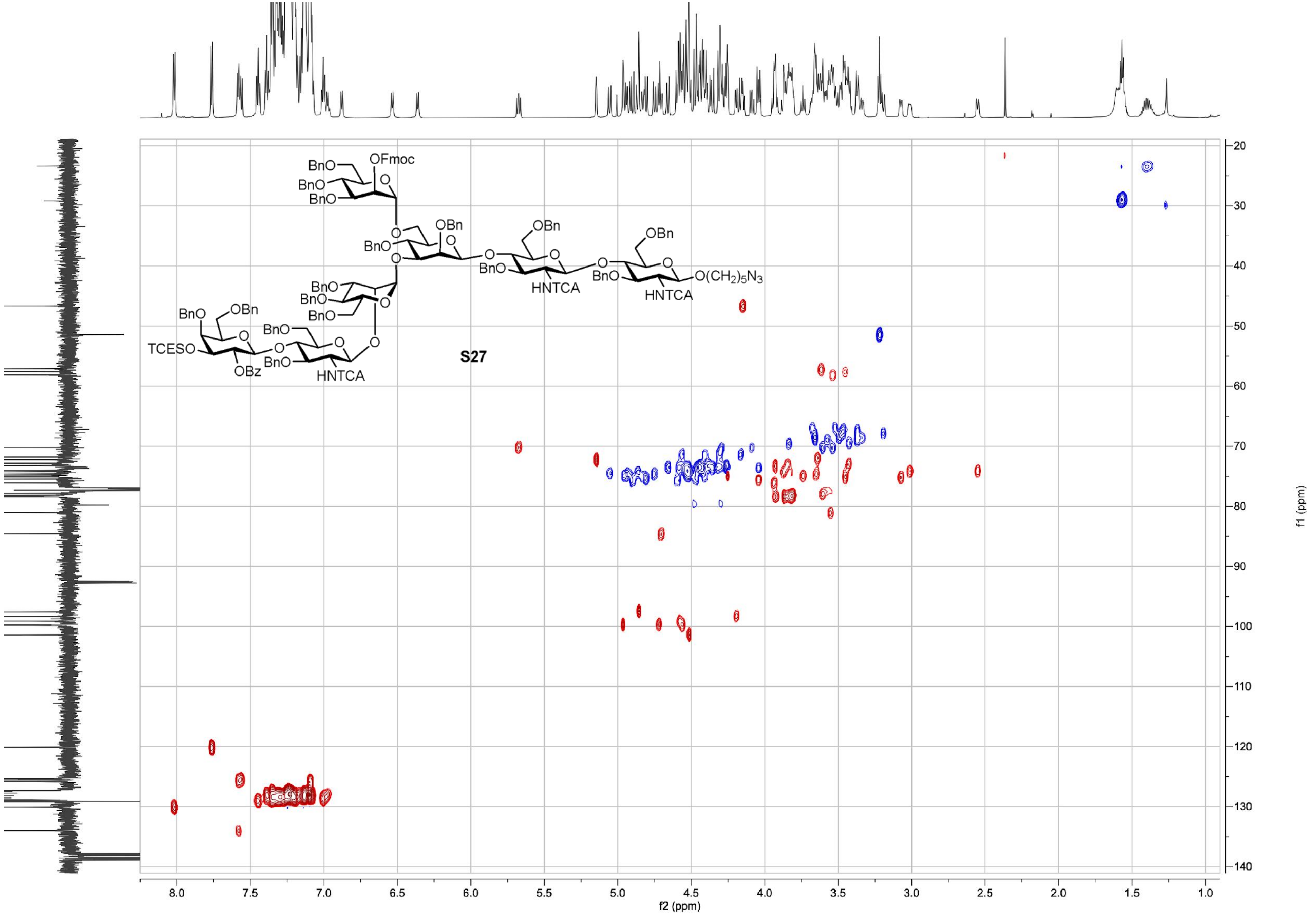

S1 *^{1}H spectrum*

500 MHz, $CDCl_3$

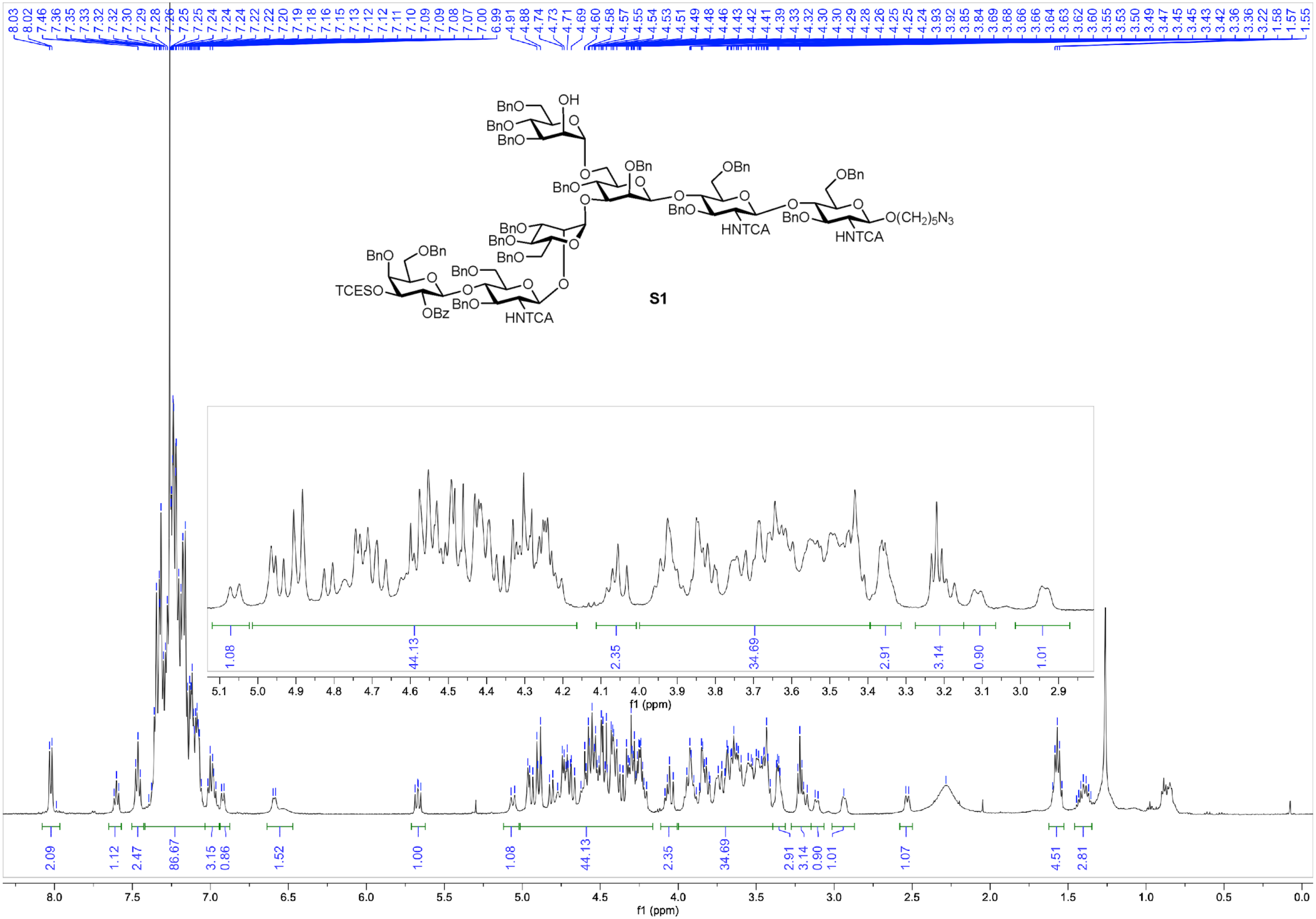

***S1** HSQC spectrum*

500 MHz for ^{1}H in $CDCl_3$, Pulse Sequence: hsqcedetgpsisp2.3, NS 4, NUS 25% (AV 500, BBO probe)

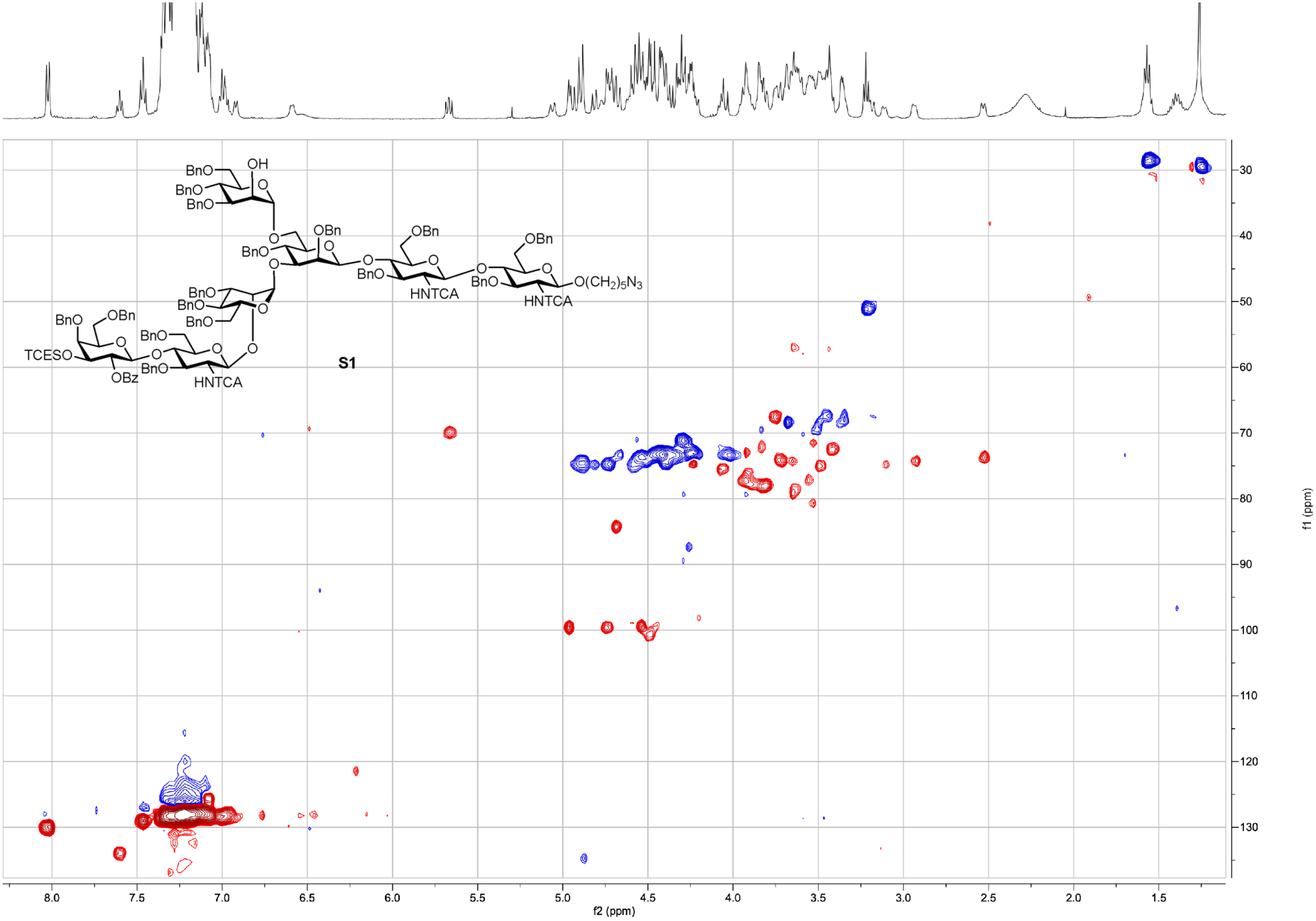

8b *^{1}H spectrum*

500 MHz, $CDCl_3$

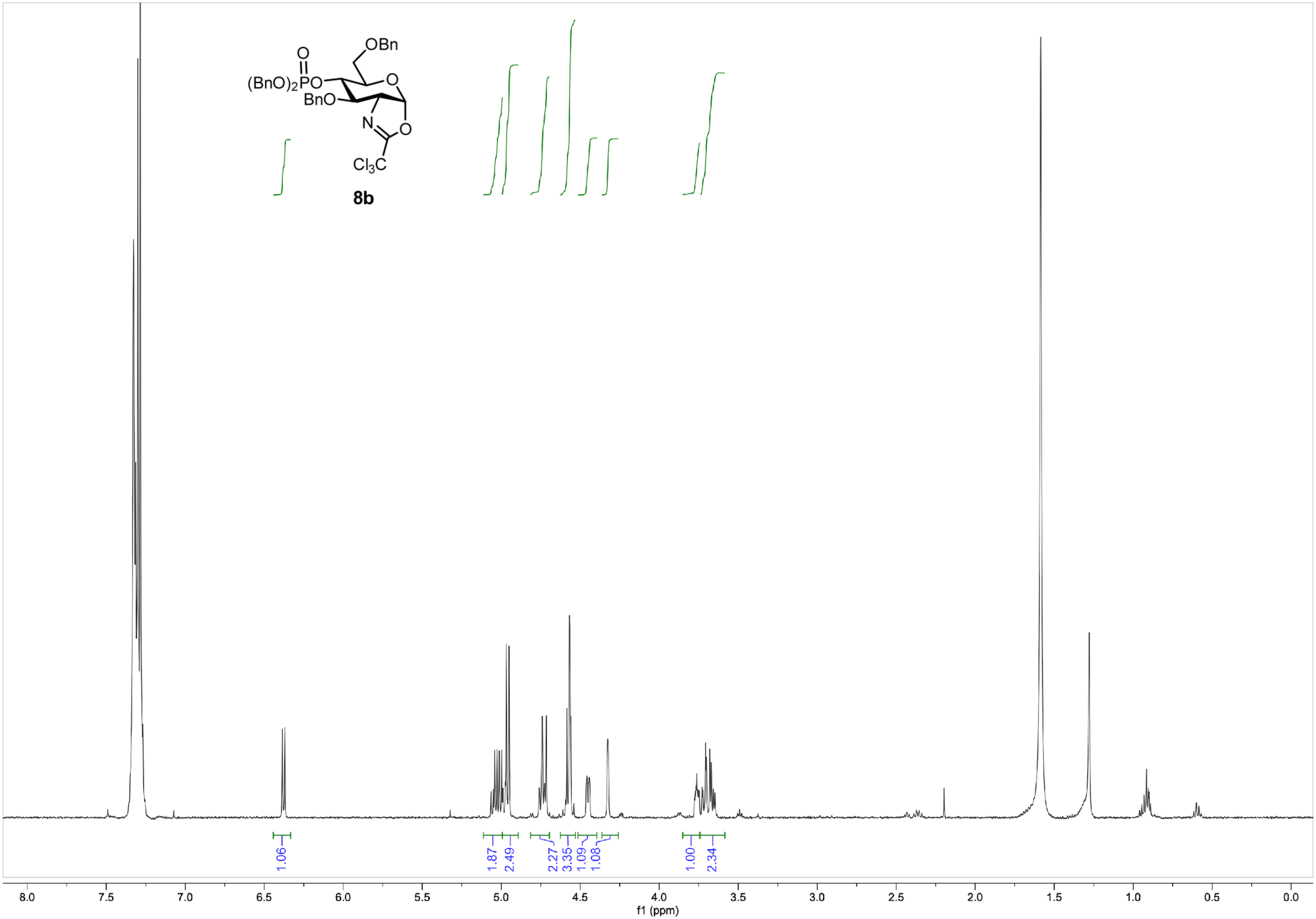

16 *^{1}H spectrum*

600 MHz, $CDCl_3$

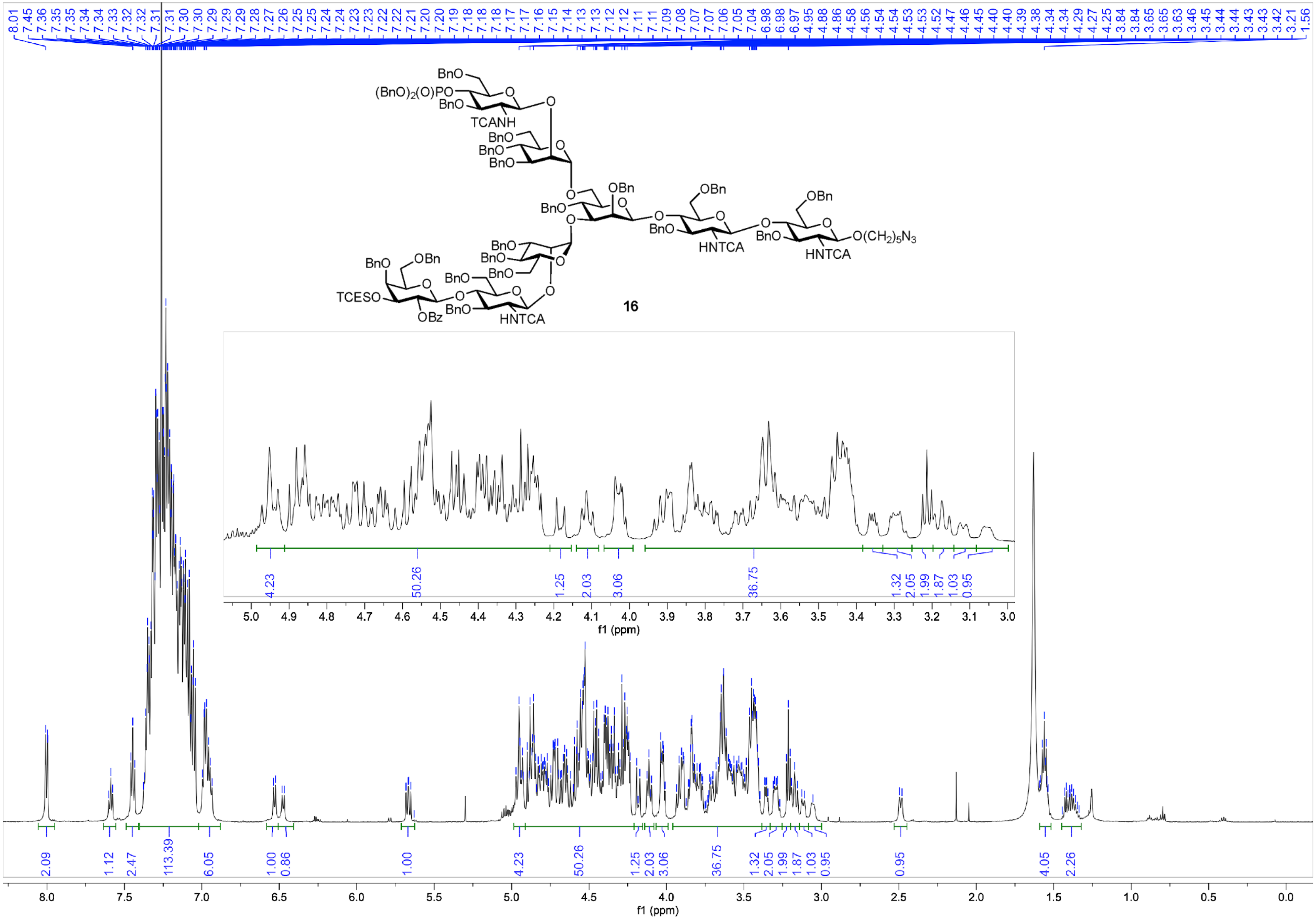

***16** DEPTQ135 ^{13}C NMR spectrum*

151 MHz in $CDCl_3$, Pulse Sequence: deptqgpsp.2, NS 298

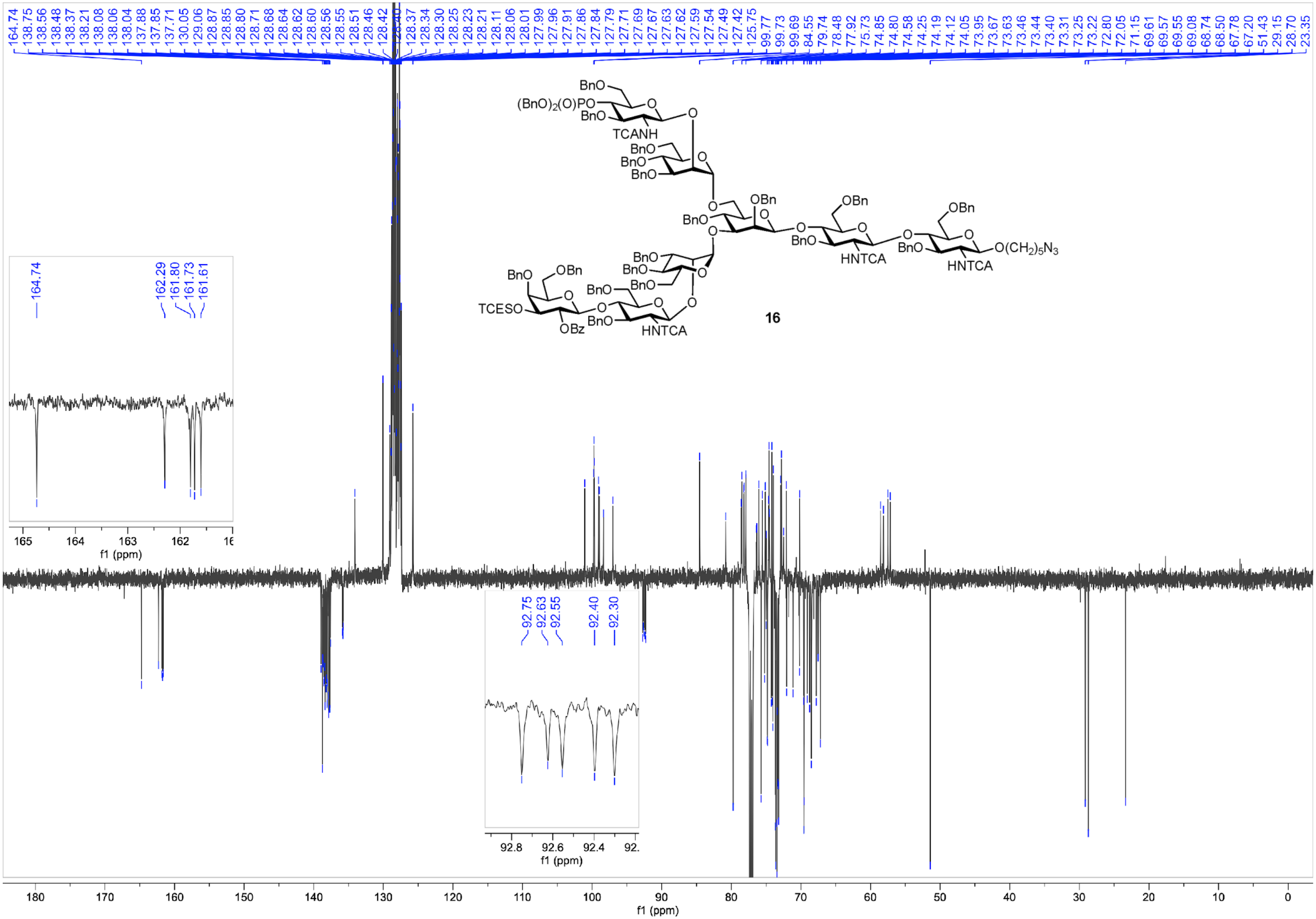

***16** HSQC spectrum*

600 MHz for ¹H in $CDCl_3$, Pulse Sequence: hsqcedetgpsisp2.3, NS 4, NUS 25%

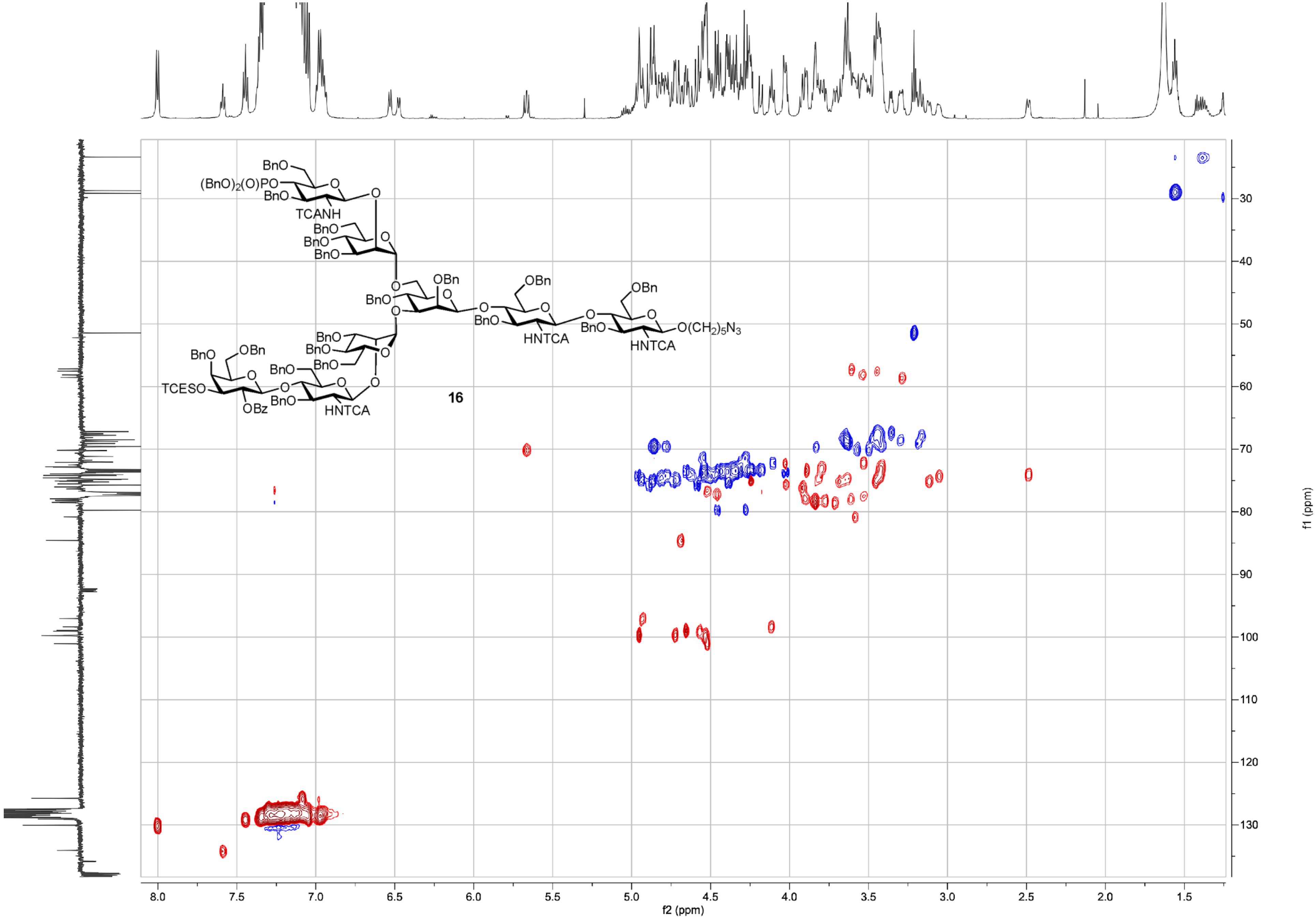

***16** HSQC spectrum with splitting via F2 phase*

600 MHz for ^{1}H in $CDCl_3$, Pulse Sequence: hsqcetgpipjcsp, NS 4, NUS 25%

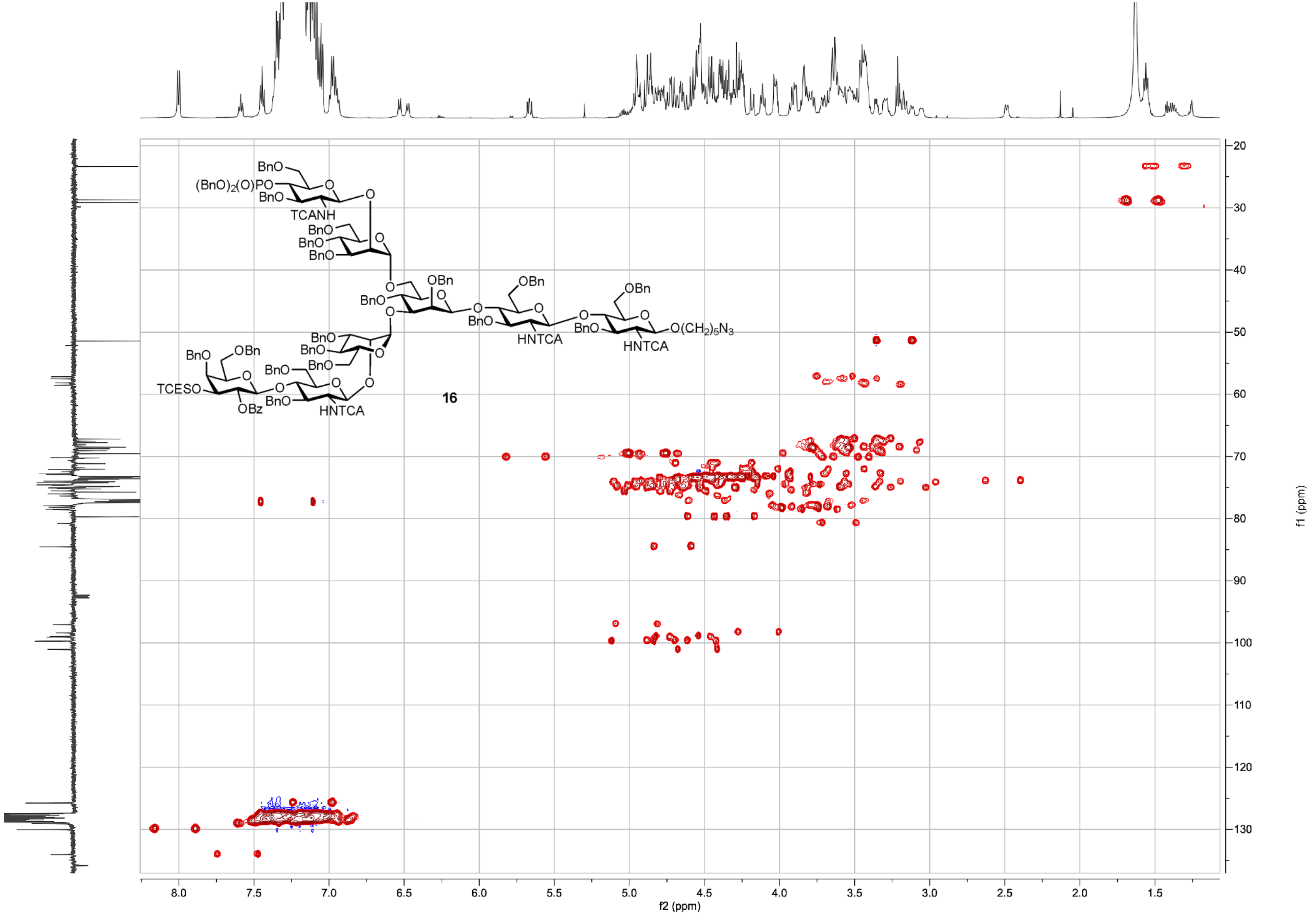

1 *^{1}H spectrum*

600 MHz in D_2O

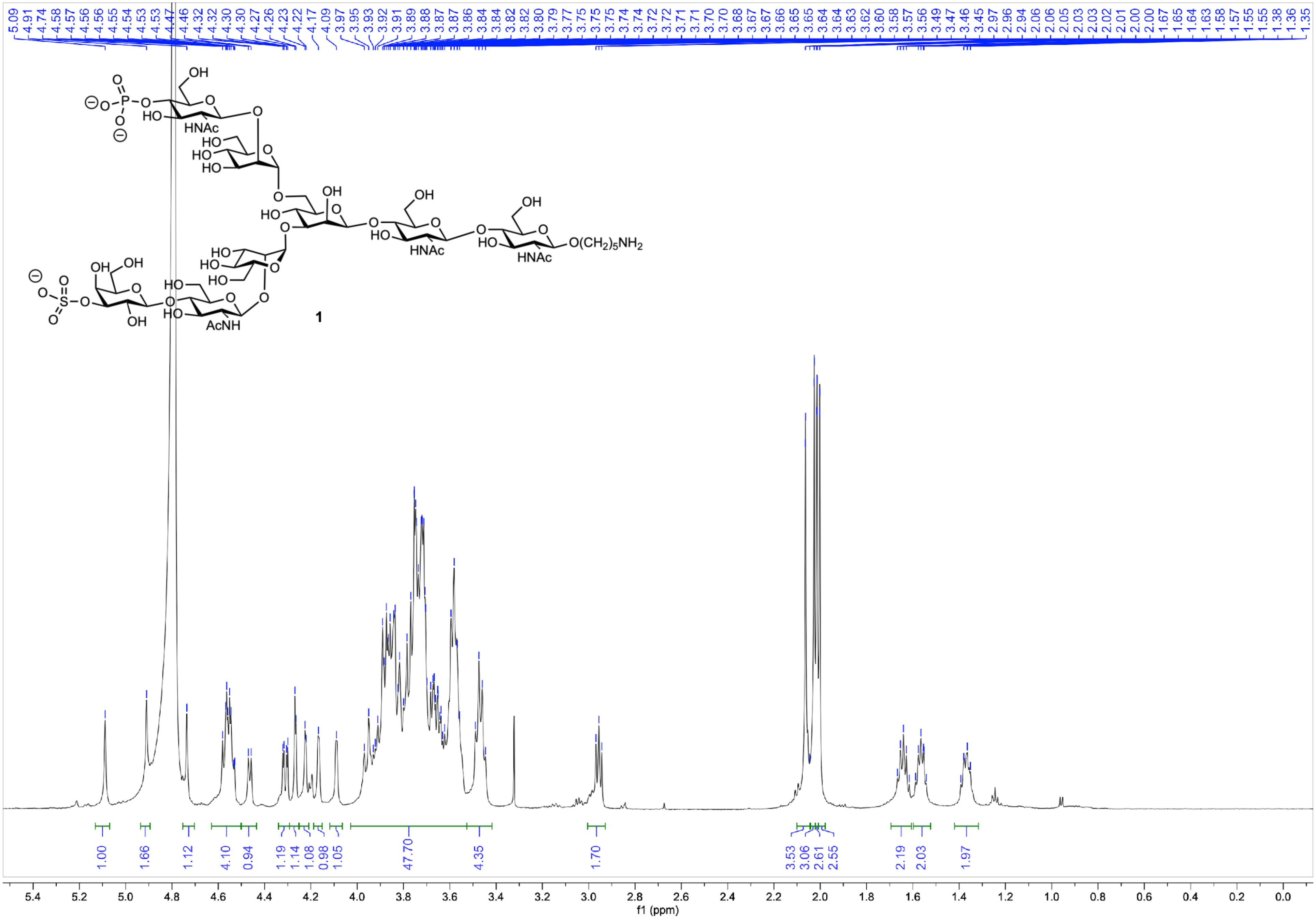

***1** DEPTQ135 ^{13}C NMR spectrum*

151 MHz in D_2O, Pulse Sequence: deptqgpsp.2, NS 293, probe DCH

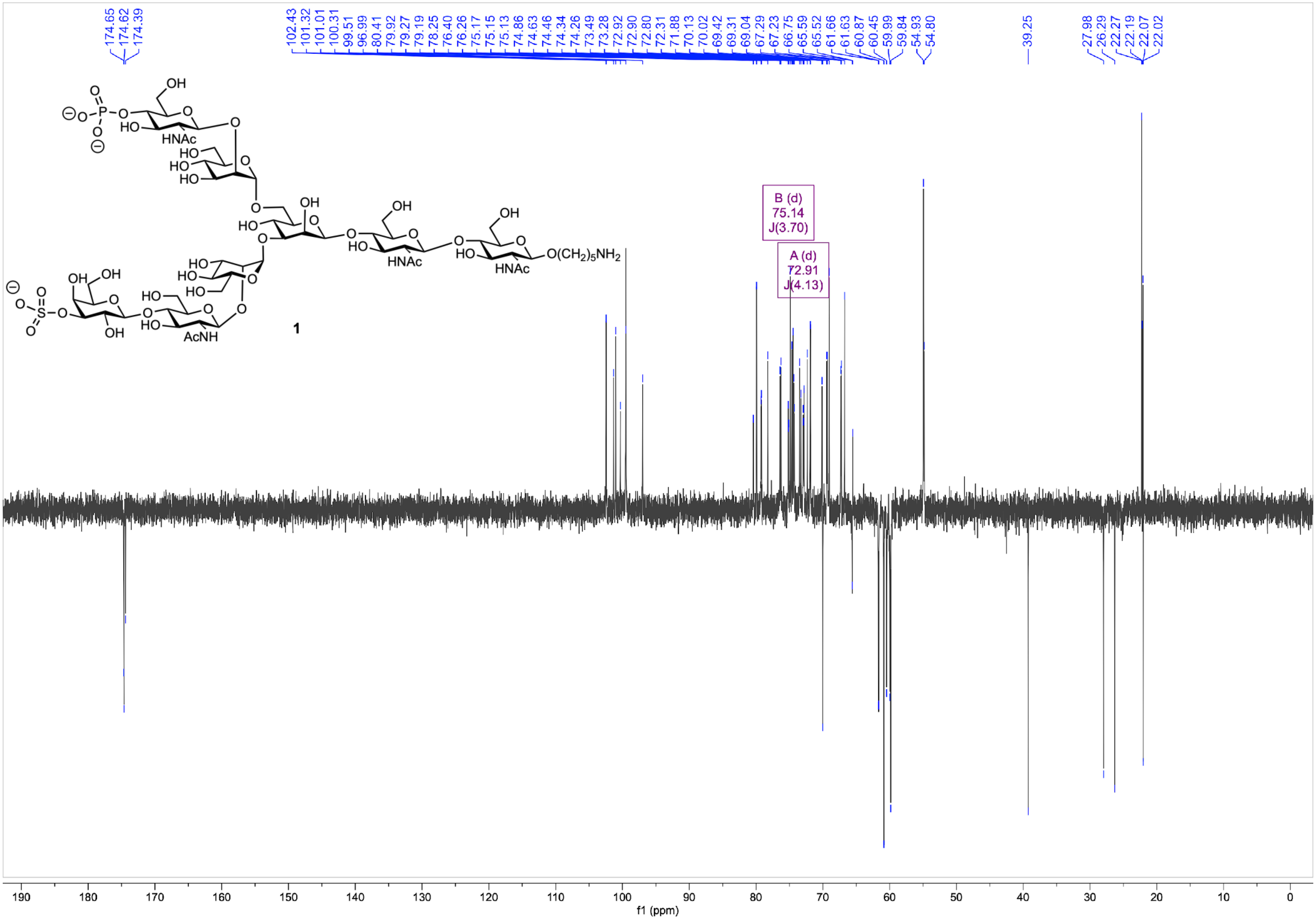

***1** HSQC spectrum*

600 MHz for ^{1}H in D_2O, Pulse Sequence: hsqcedetgpsisp2.3, NS 4, NUS 25%, AV 600, probe DCH

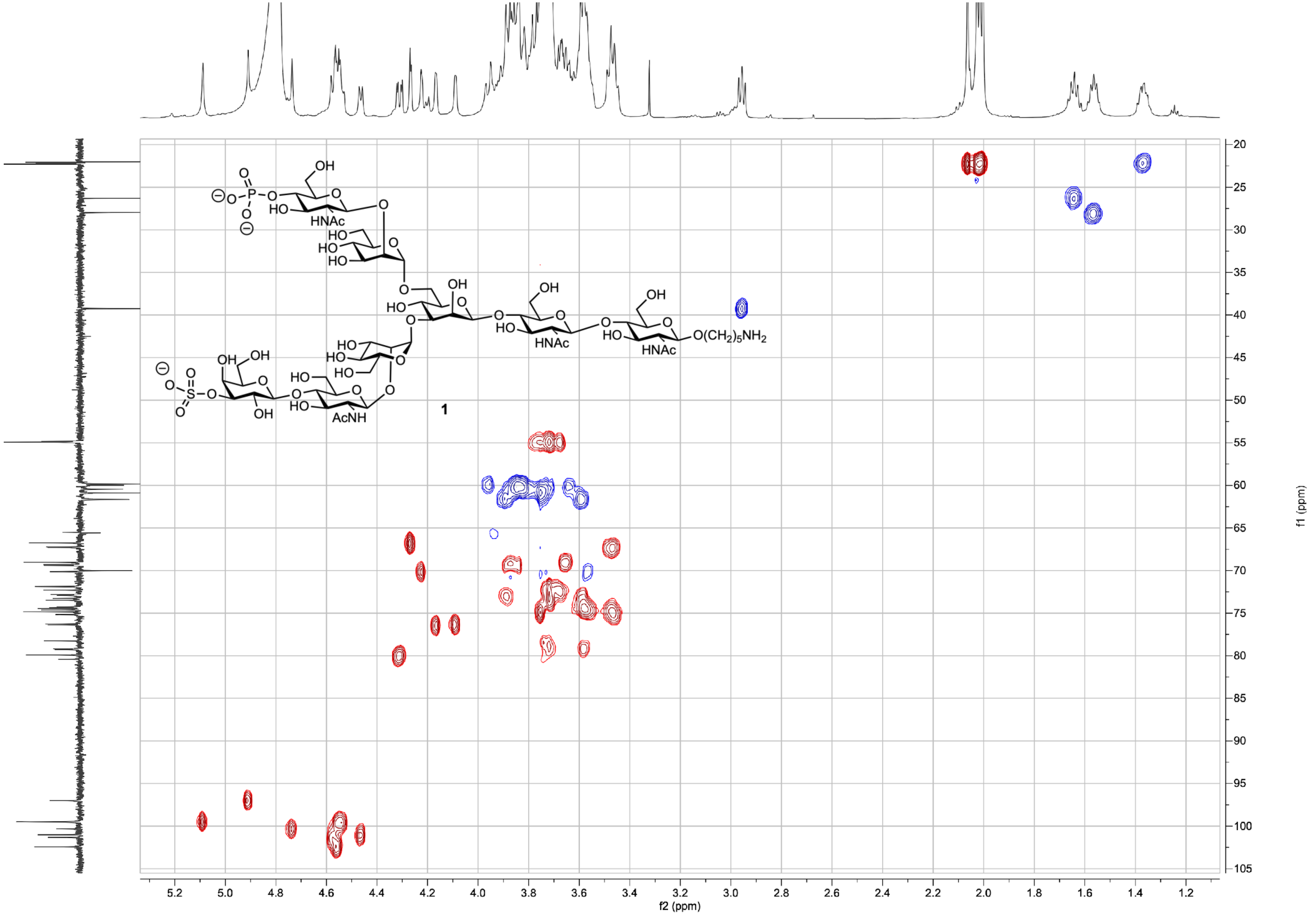

***1** HSQC spectrum with splitting via F2 phase*

600 MHz for ^{1}H in D_2O, Pulse Sequence: hsqcetgpipjcsp, NS 4, NUS 25%, AV 600, probe DCH

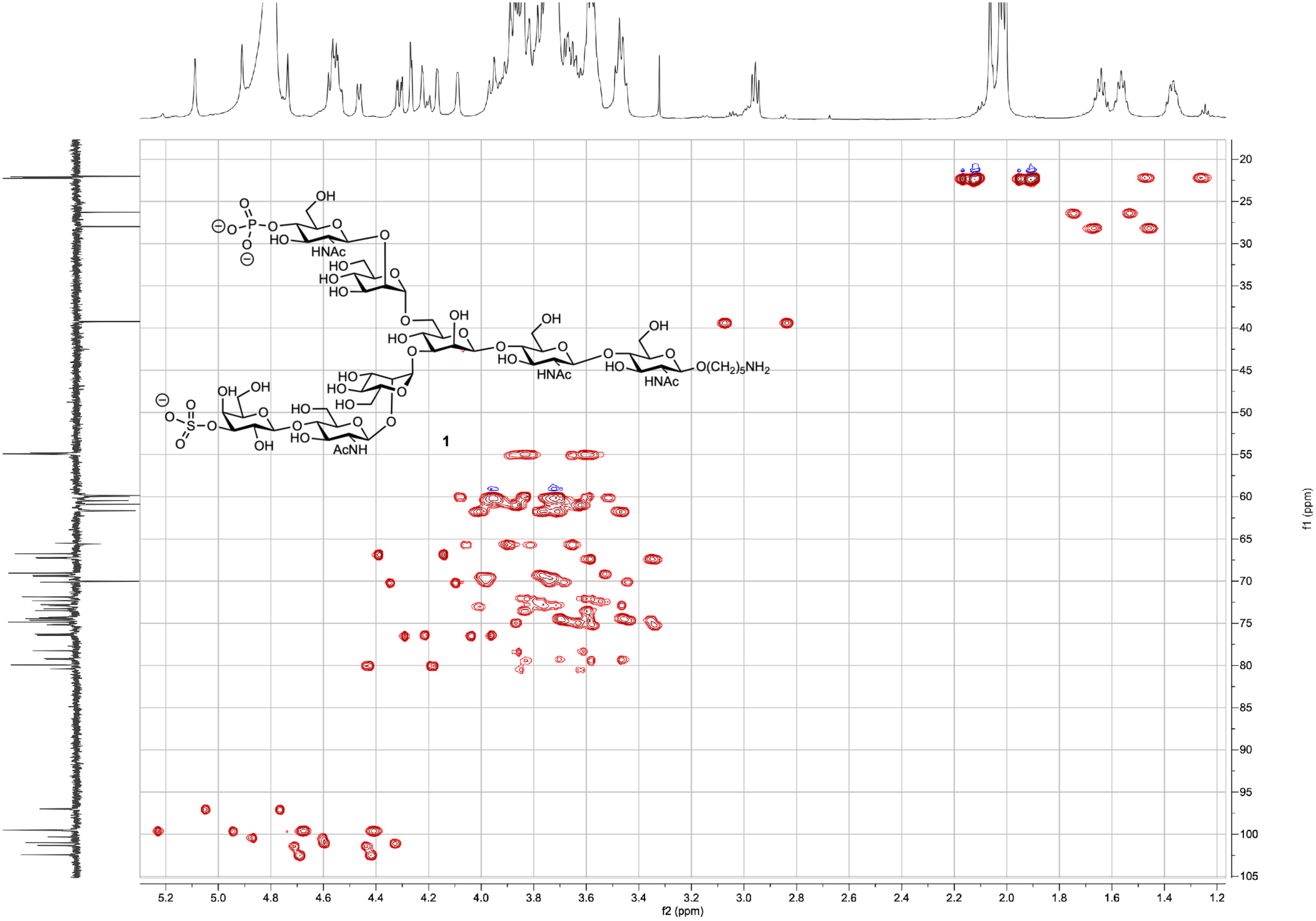

***1** superimposed HSQC spectra*

Cyan: HSQC splitting via F2 phase; Red and Blue: HSQC-edited

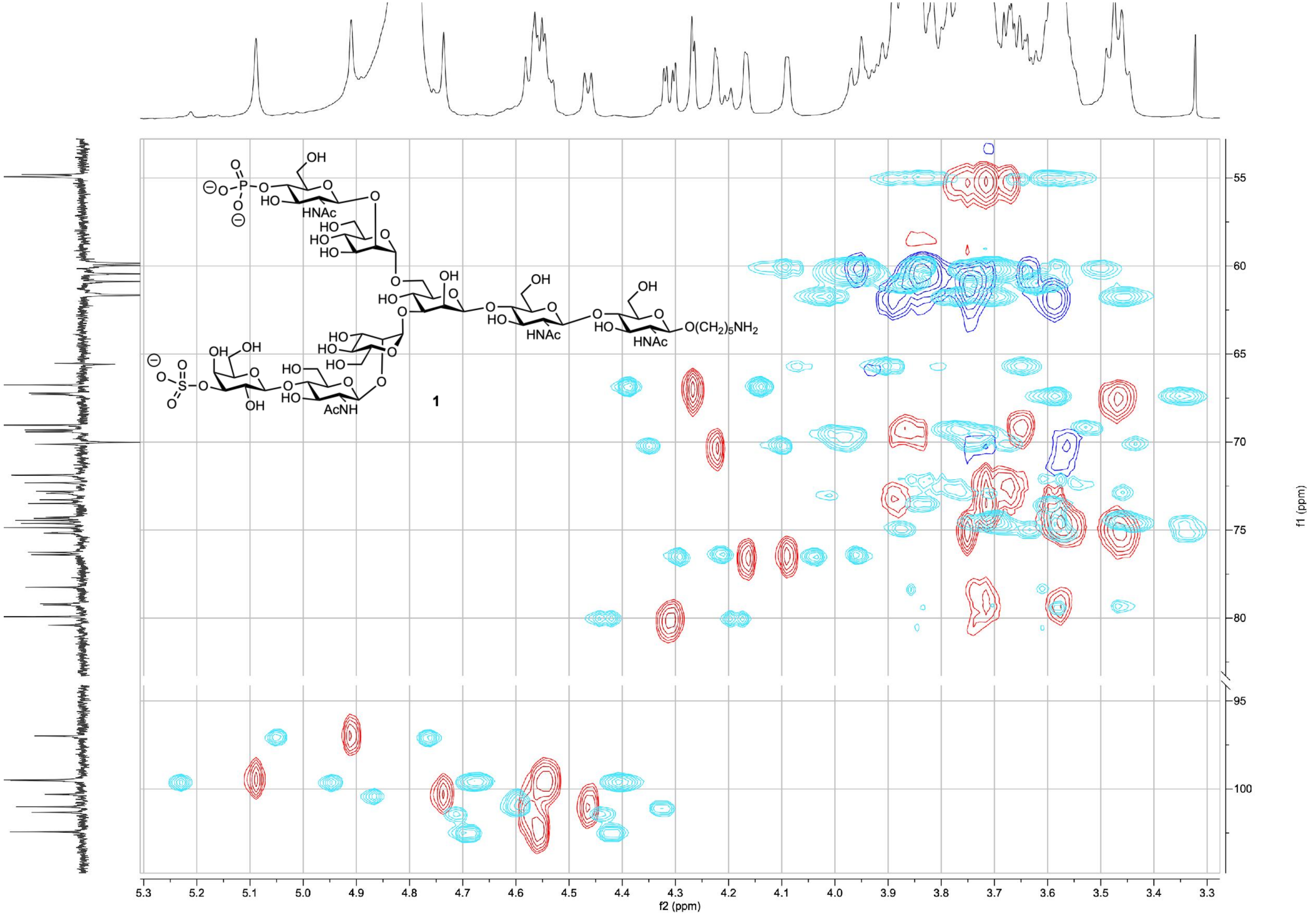

***1** COSY spectrum*

600 MHz for ^{1}H in D_2O

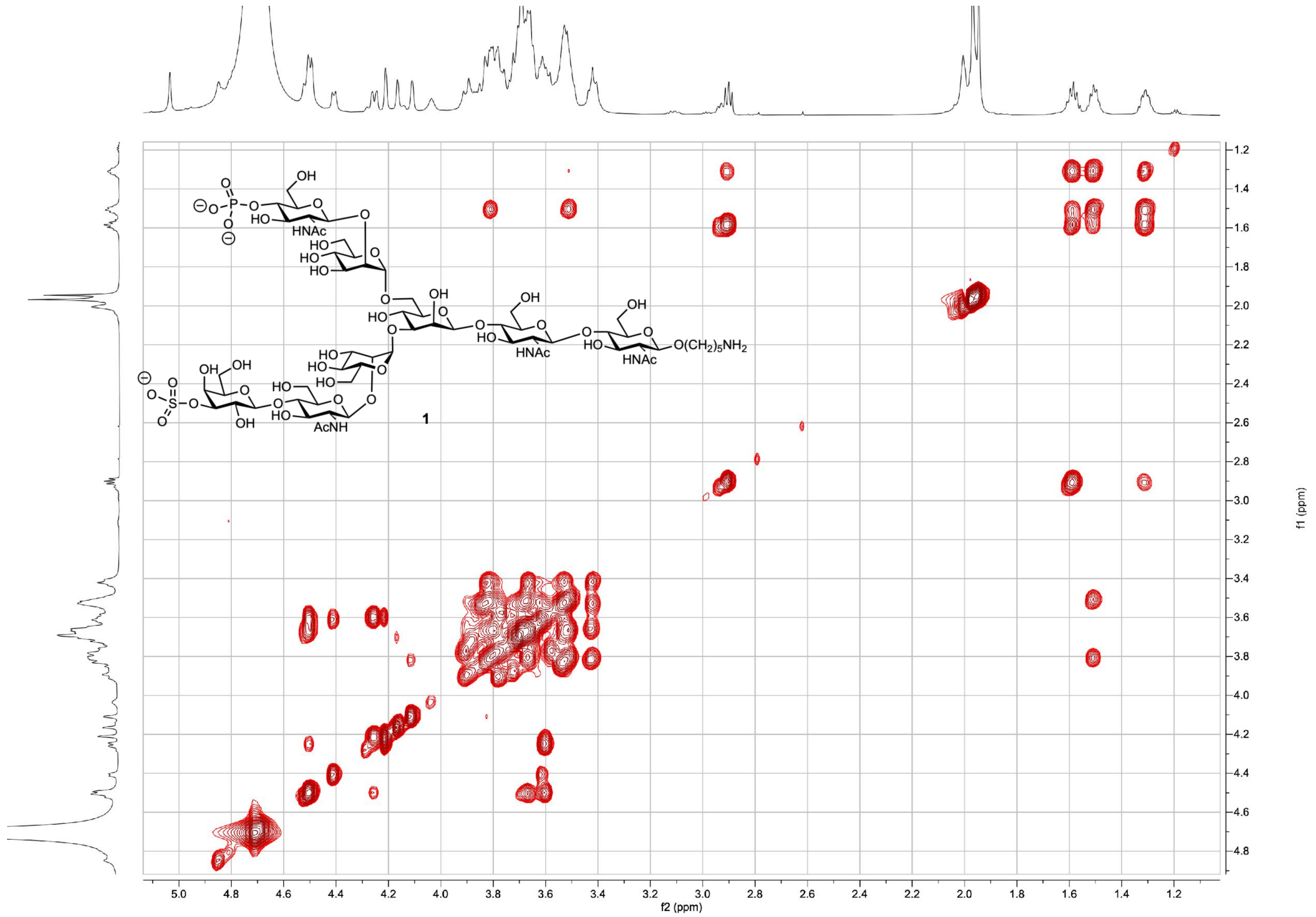

***1** TOCSY-HSQC spectrum*

600 MHz for ^{1}H in D_2O, Pulse Sequence: hsqcgpmlph, NS 16, NUS 25%, AV 600, Probe: QCI

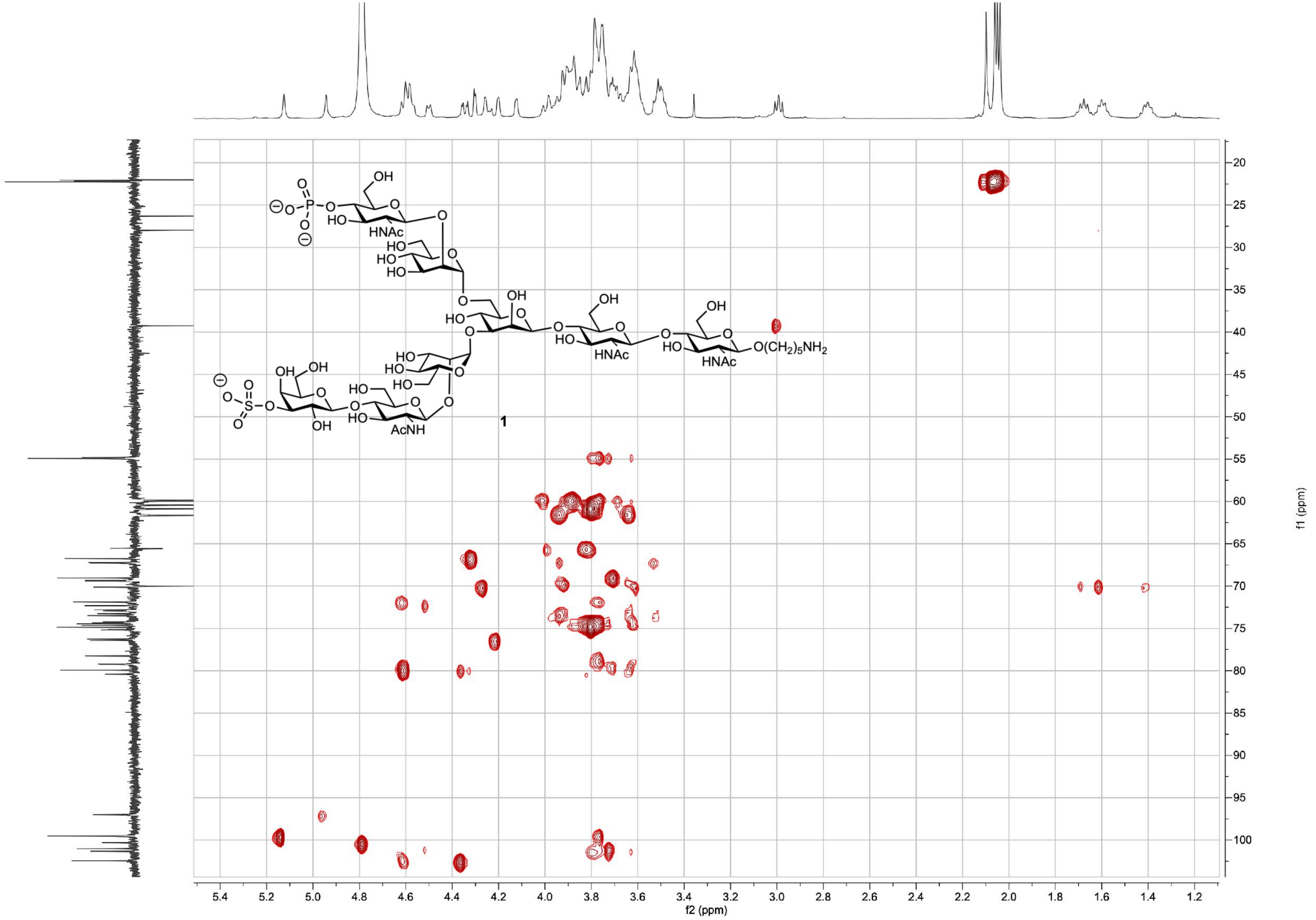

***1** HMBC spectrum*

600 MHz for ^{1}H in D_2O, Pulse Sequence: clhmbcetgpl3nd, NS 32, NUS 50%, AV 600, Probe: QCI

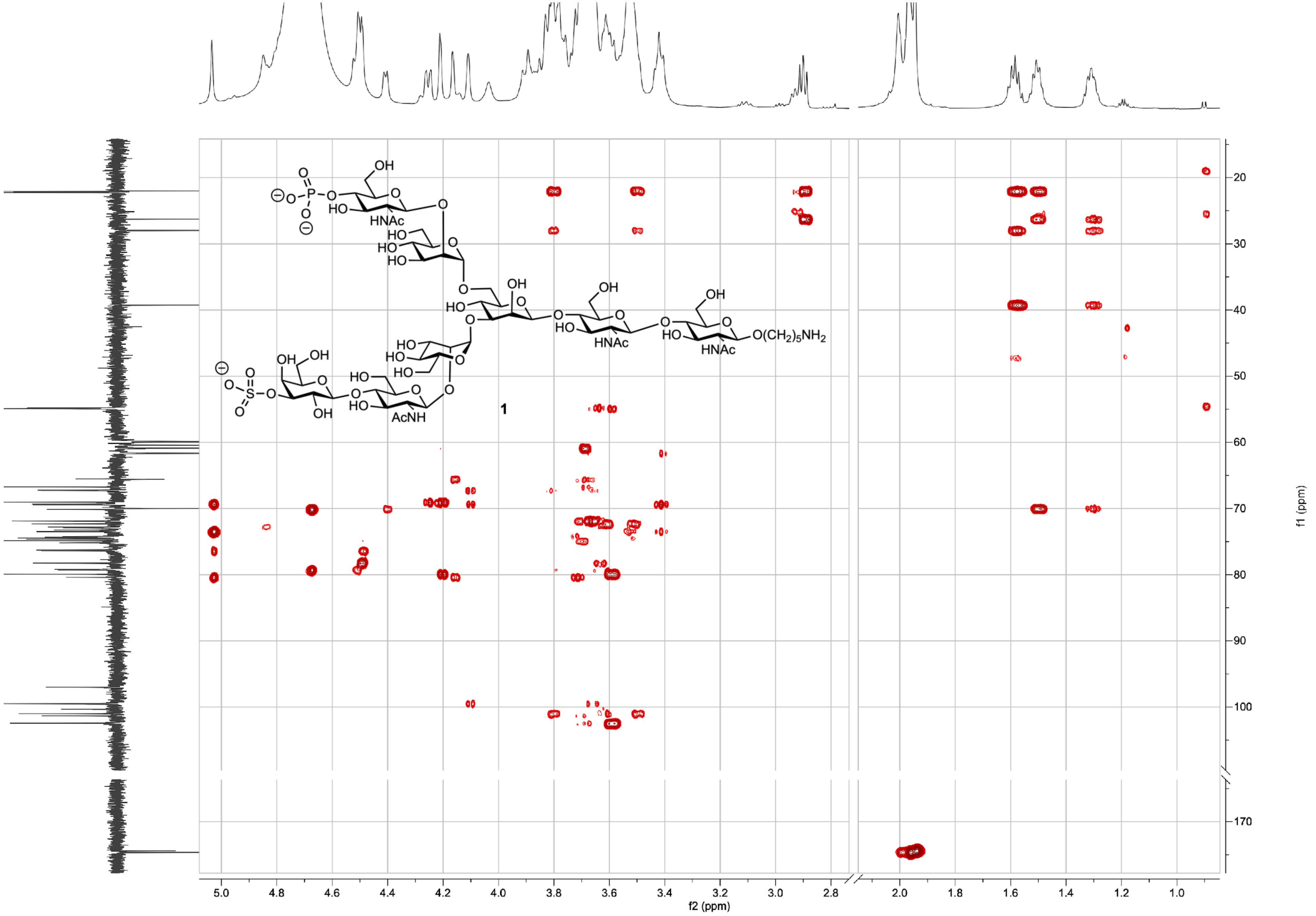

21 *^{1}H spectrum*

600 MHz in D_2O

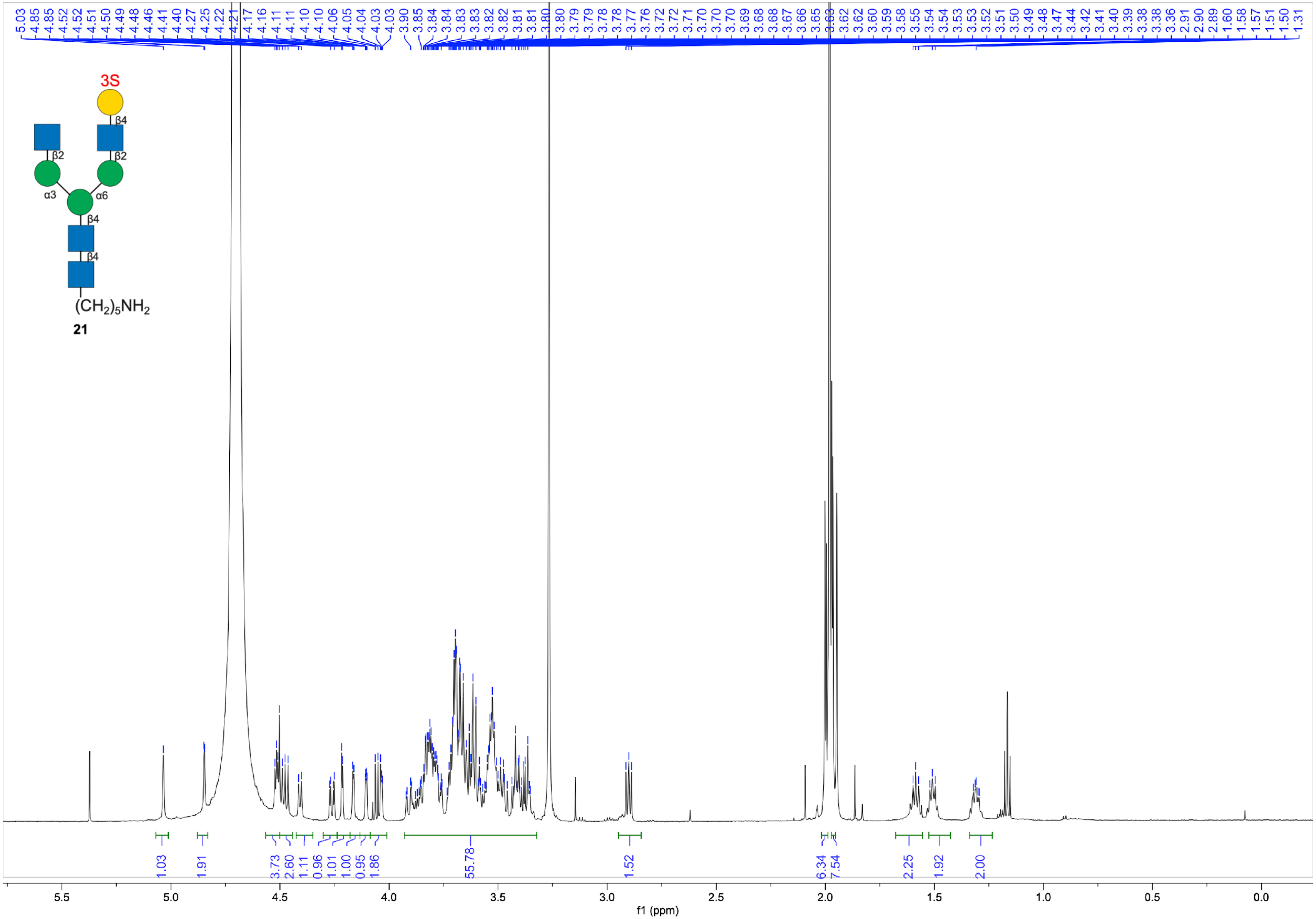

21 *HSQC spectrum*

600 MHz for ^{1}H in D_2O, Pulse Sequence: hsqcedetgpsisp2.3, NS 4, NUS 25%, AV 600, probe QCI

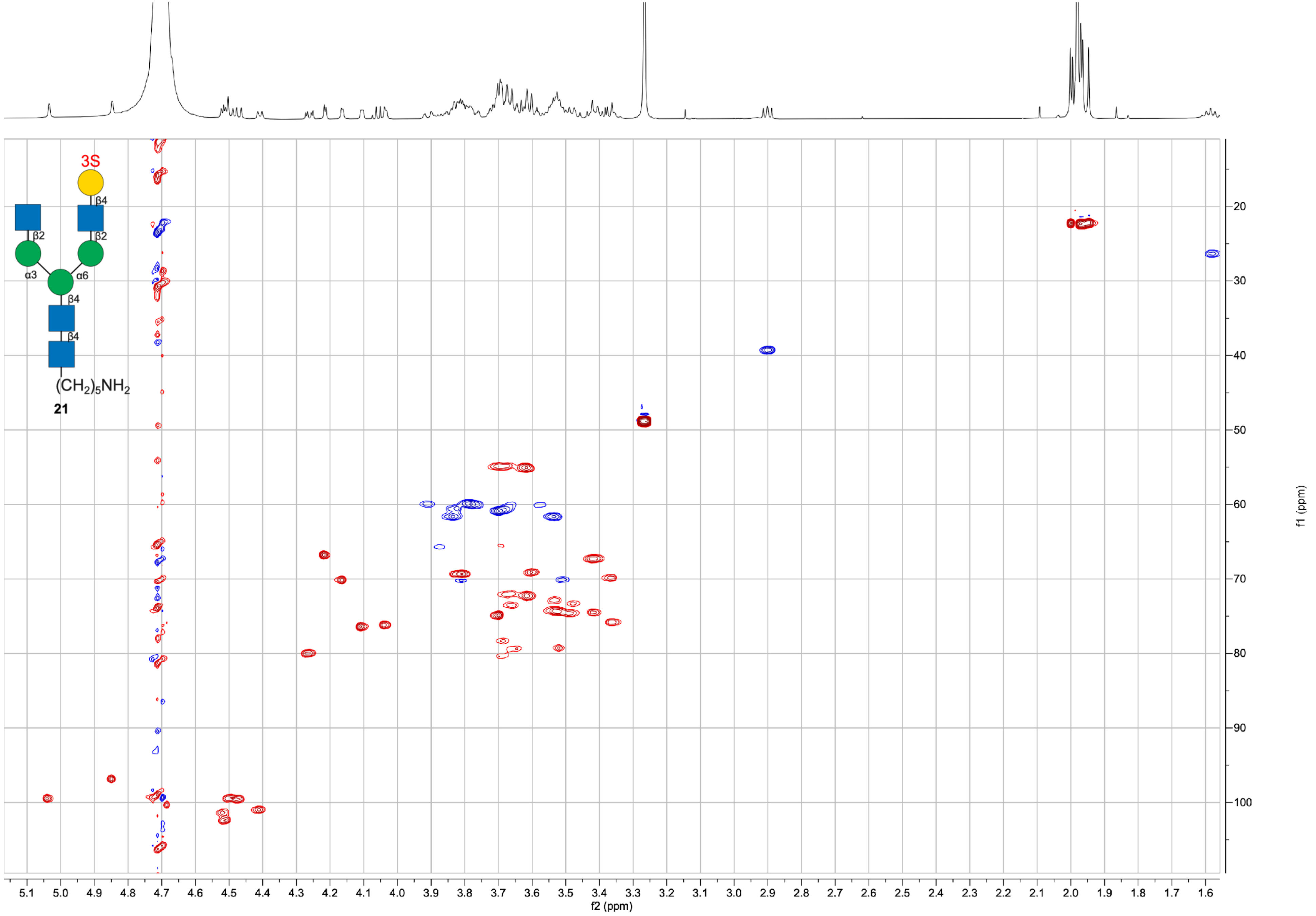

22 *^{1}H NMR spectrum*

600 MHz in D_2O

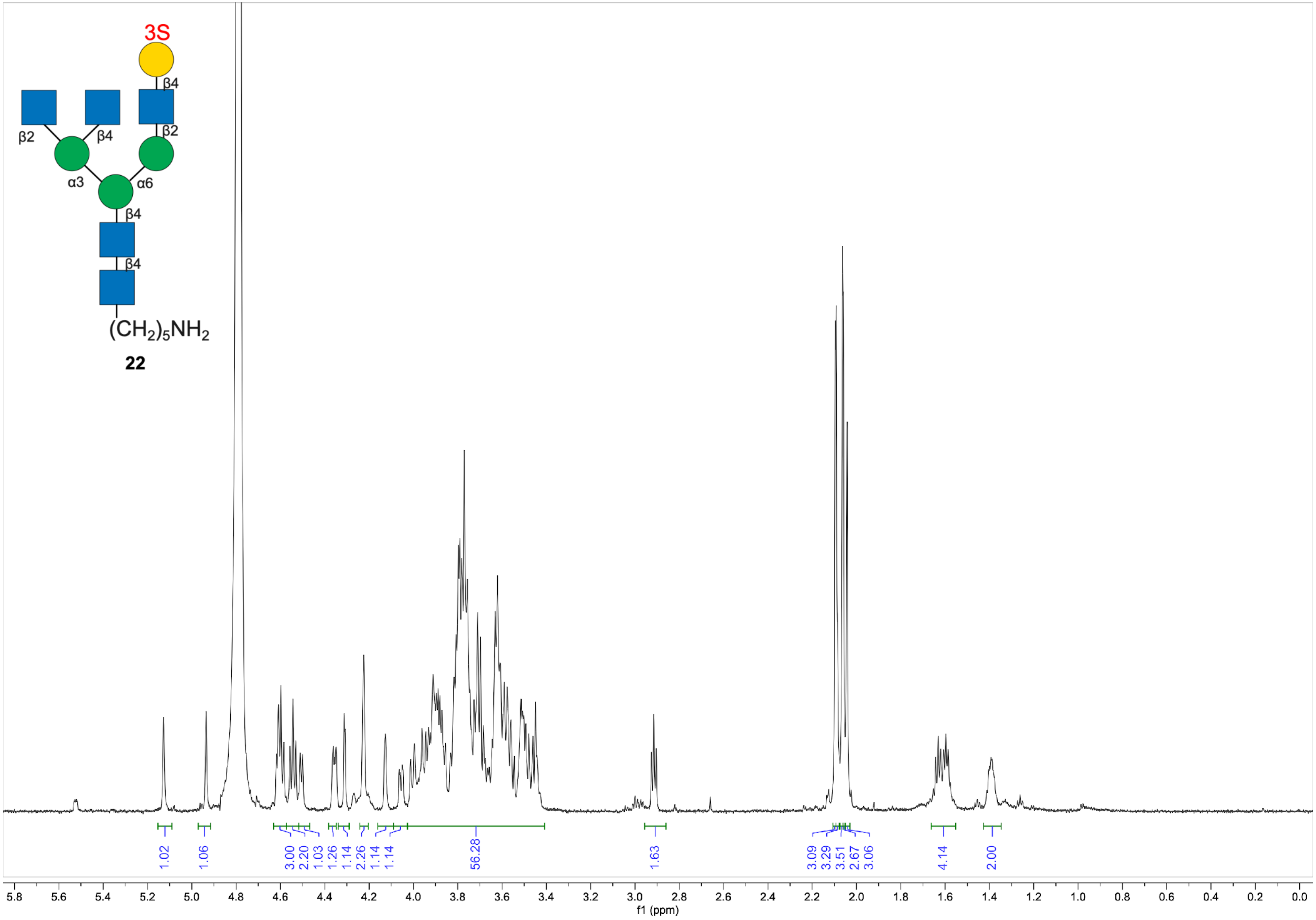

***22** HSQC spectrum*

700 MHz for ^{1}H in D_2O, Pulse Sequence: hsqcedetgpsisp2.2, NS 8, NUS 25%, AV 700, probe CPTCI

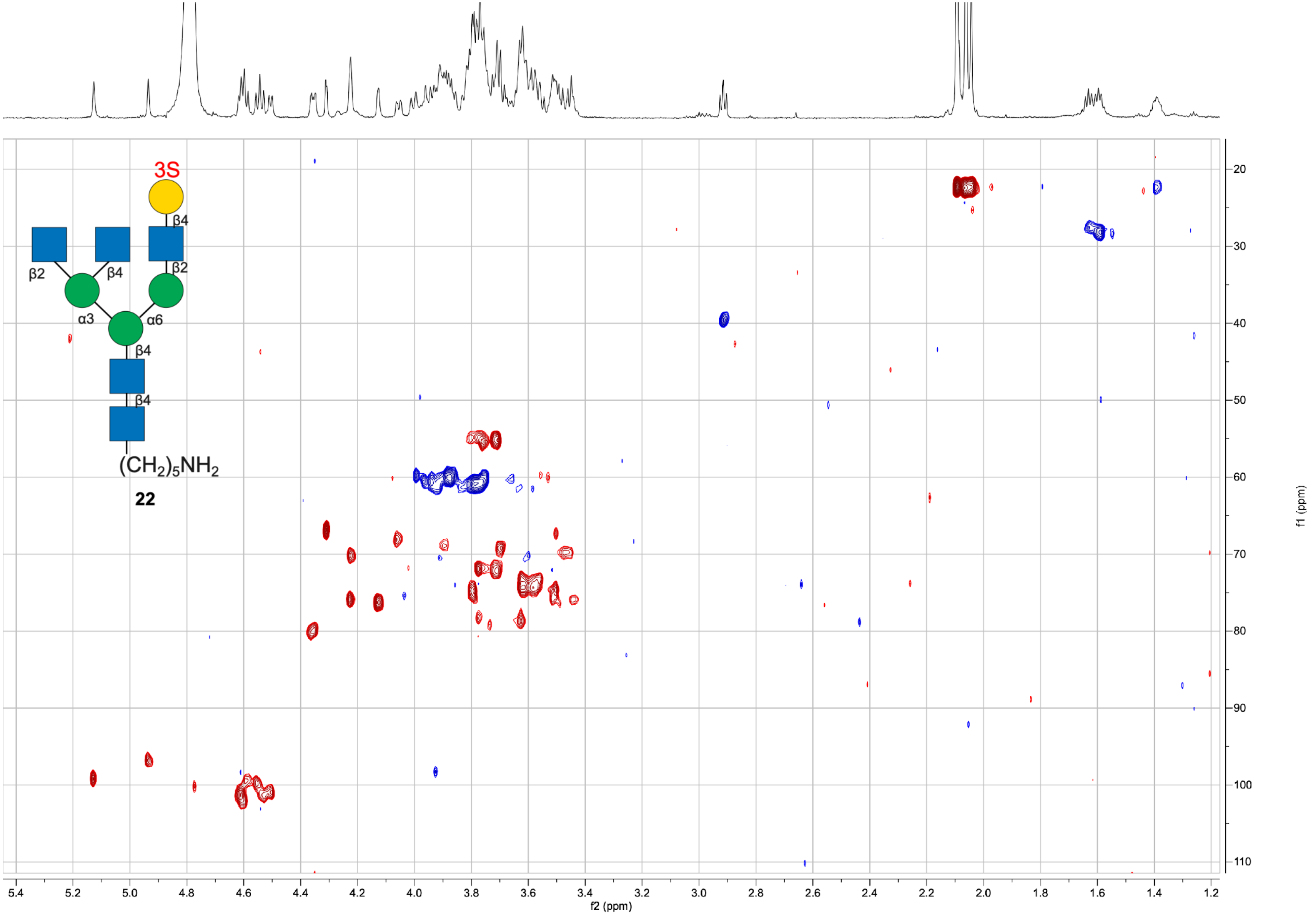

25 *^{1}H NMR spectrum*

600 MHz in D_2O

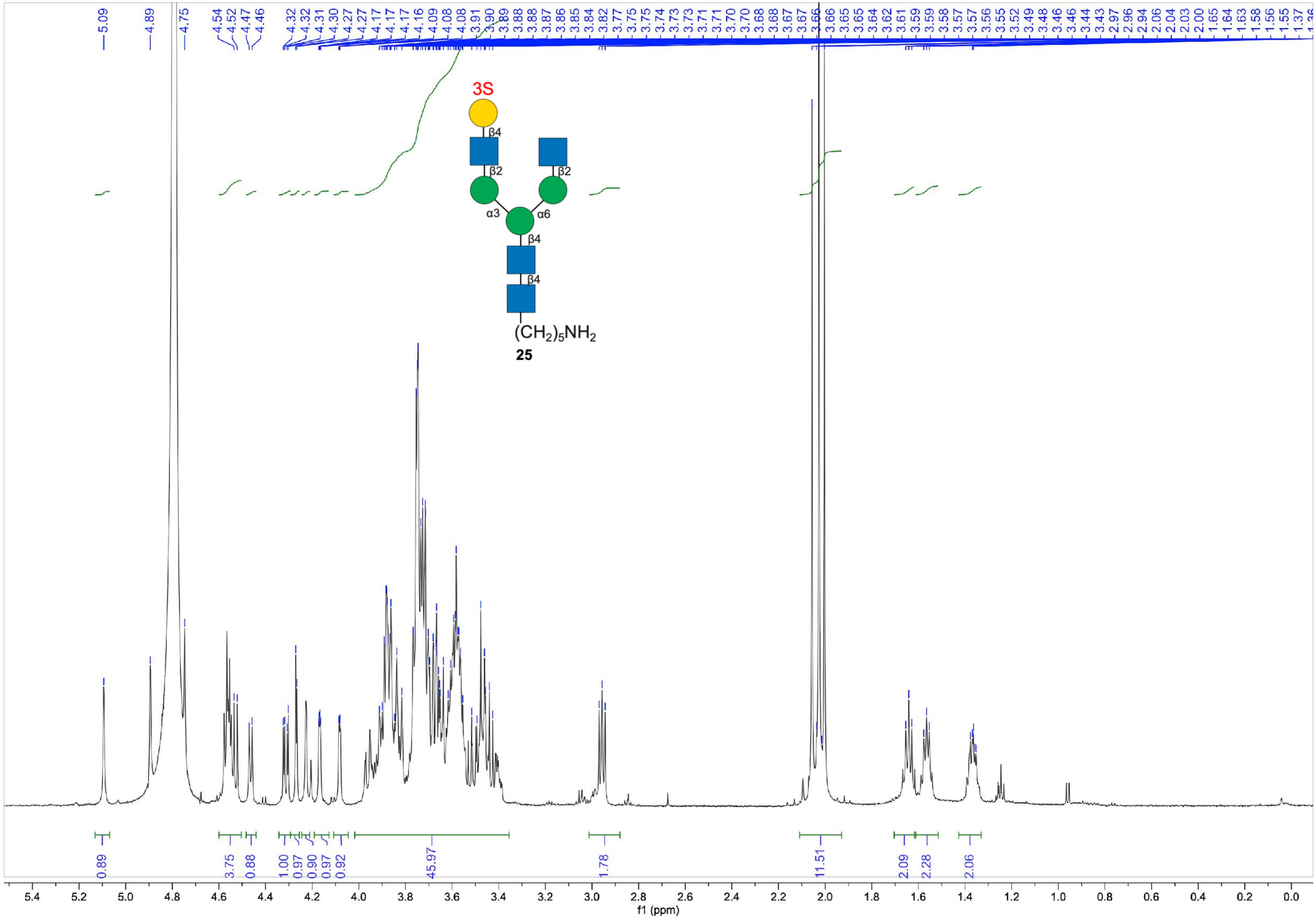

25 *DEPTQ135* ^{13}*C NMR spectrum*

151 MHz in D_2O, Pulse Sequence: deptqgpsp.2, NS 480, probe DCH

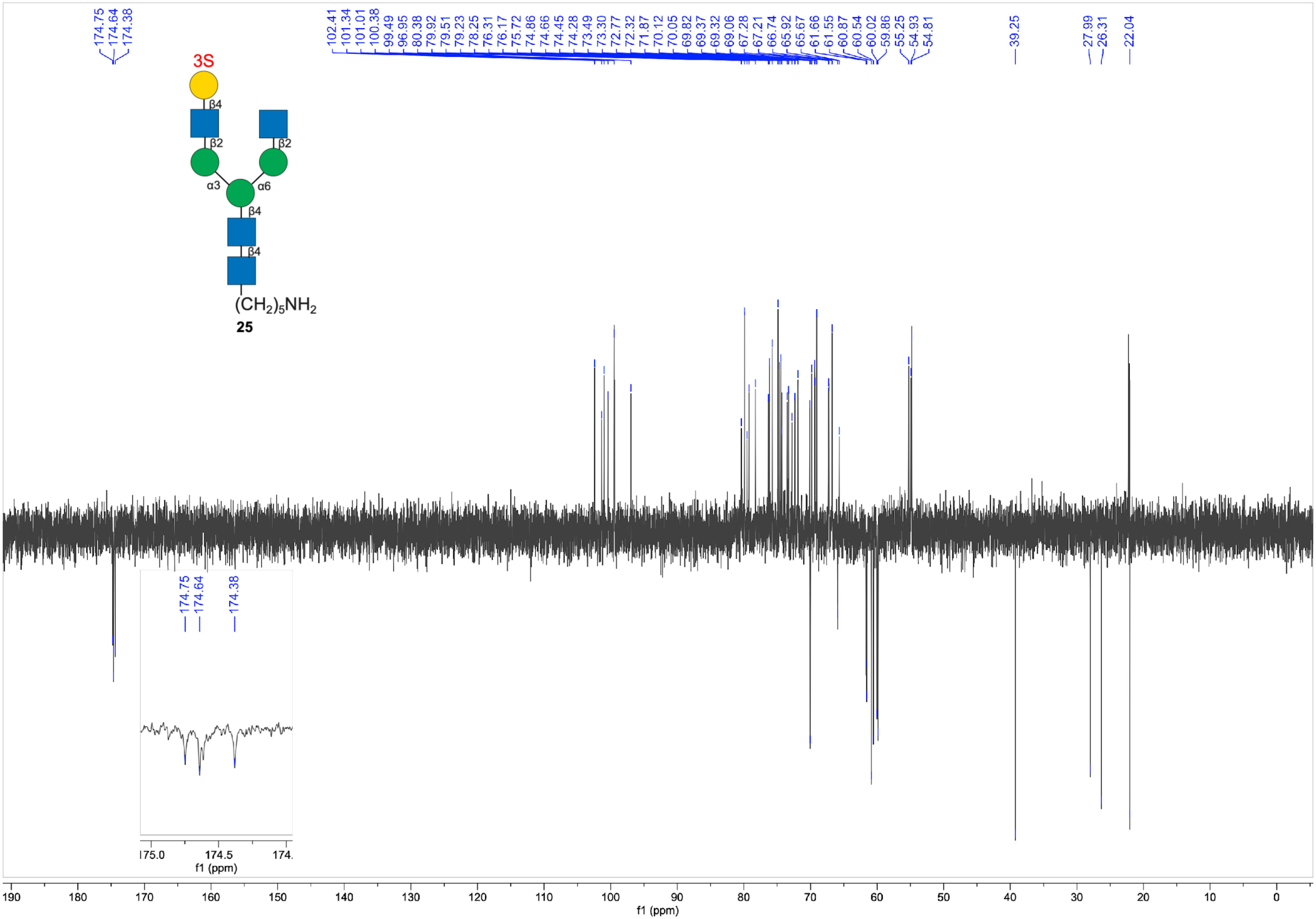

***25** HSQC spectrum*

600 MHz for ^{1}H in D_2O, Pulse Sequence: hsqcedetgpsisp2.3, NS 4, NUS 25%, AV 600, probe DCH

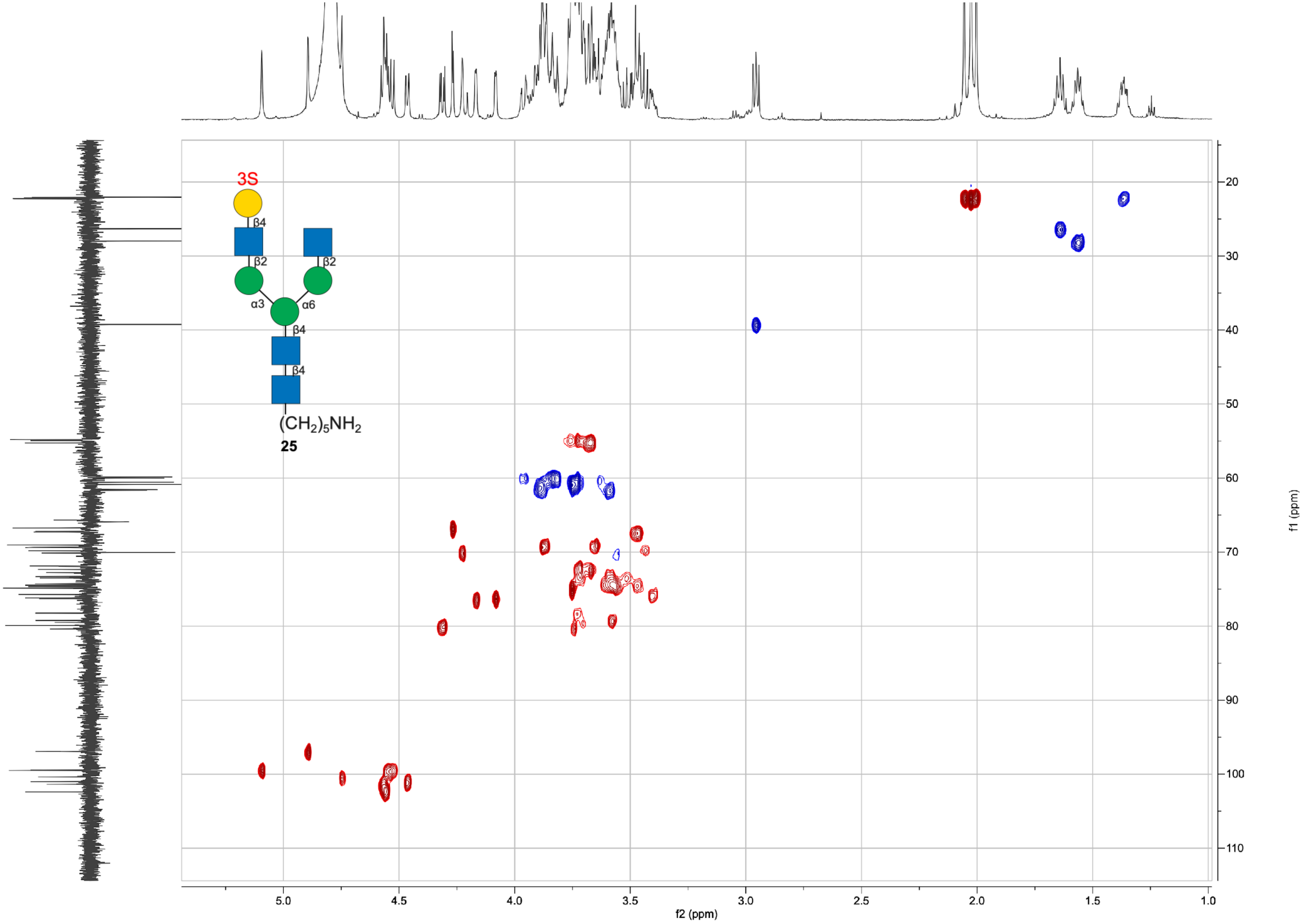

__25__ HSQC spectrum with splitting via F2 phase

600 MHz for ^{1}H in D_2O, Pulse Sequence: hsqcetgpipjcsp.2, NS 4, NUS 25%, AV 600, probe DCH

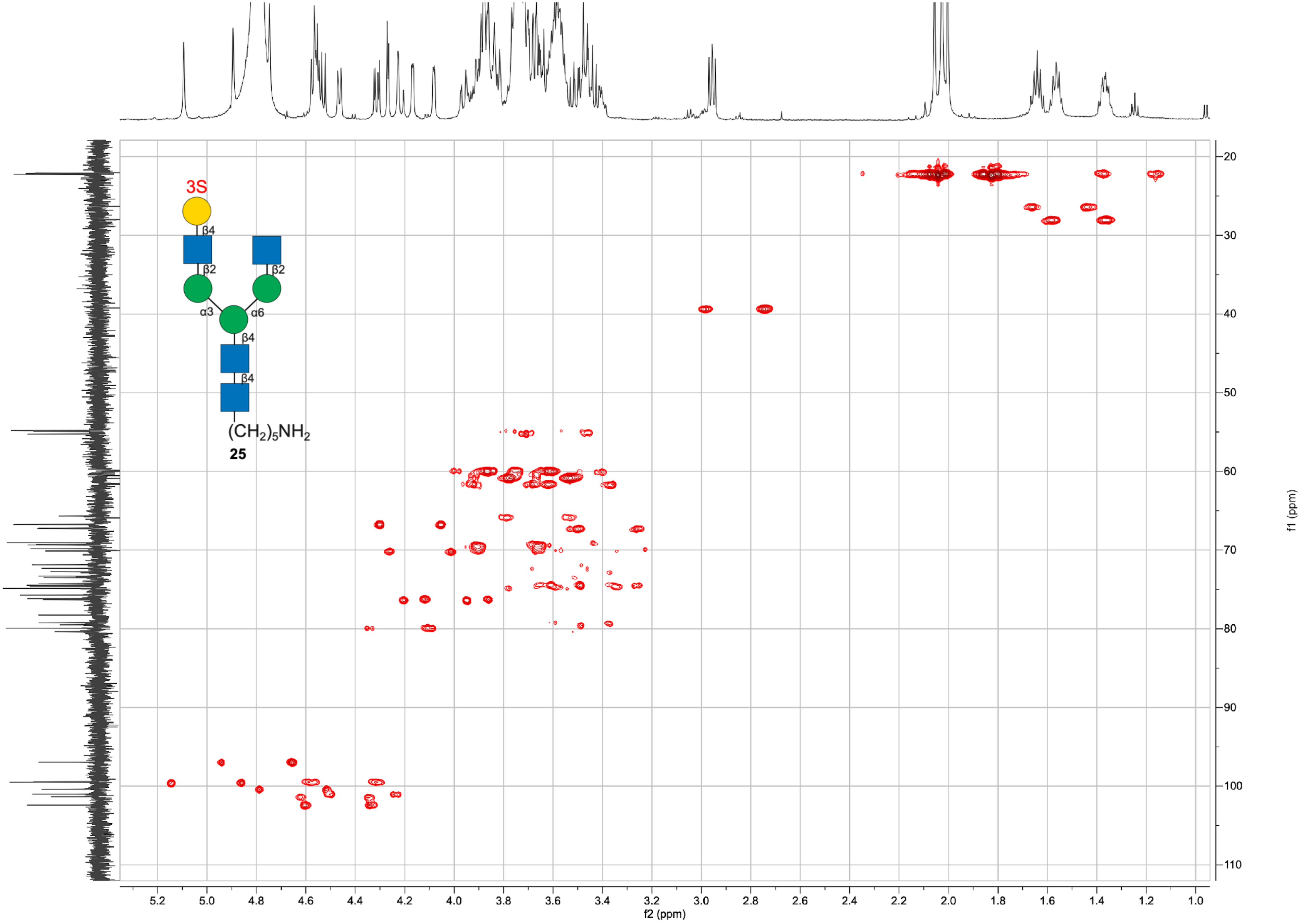

26 *^{1}H NMR spectrum*

700 MHz in D_2O

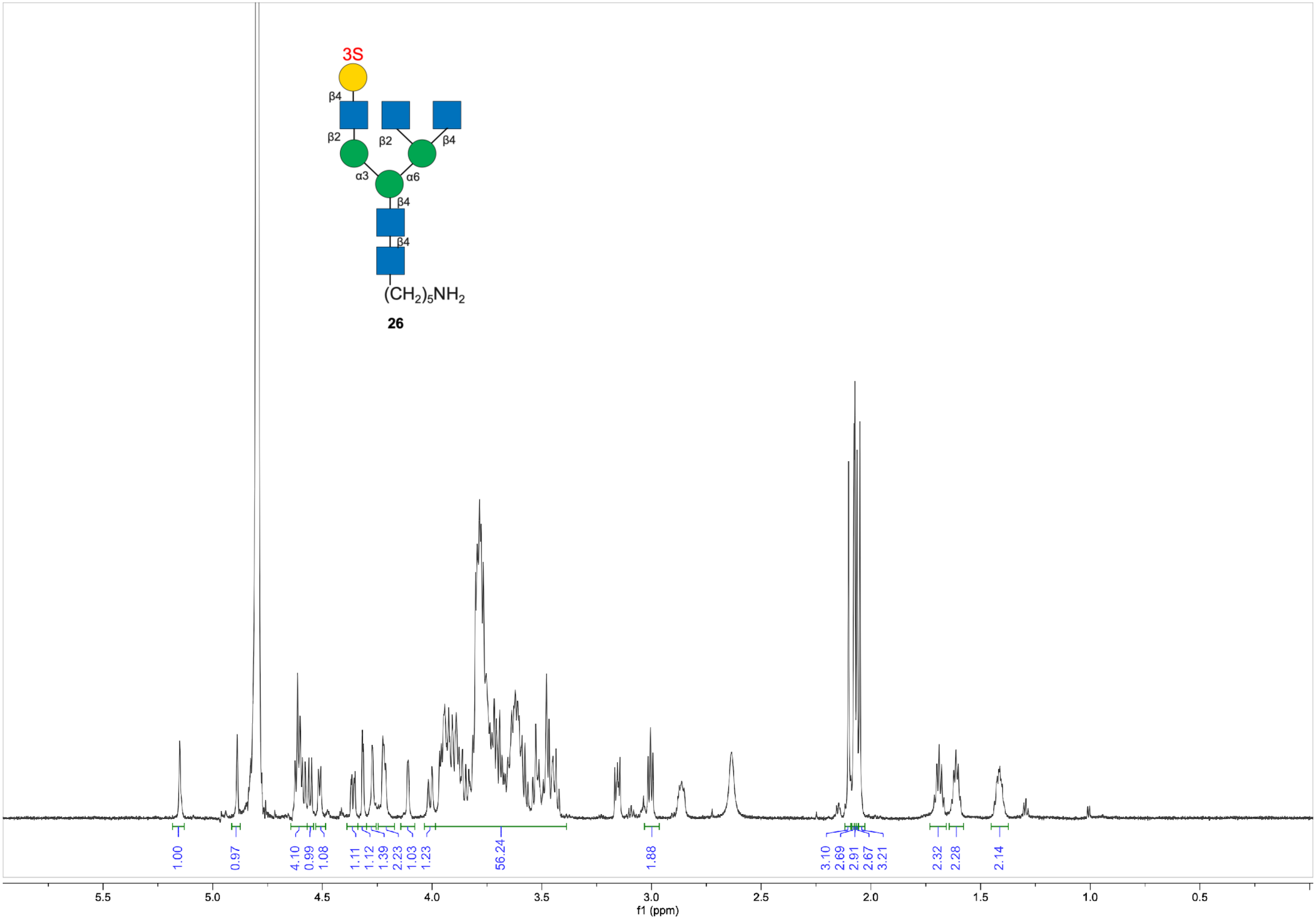

26 *HSQC spectrum*

700 MHz for ^{1}H in D_2O, Pulse Sequence: hsqcedetgpsisp2.3, NS 8, NUS 25%, AV700, probe CPTCI

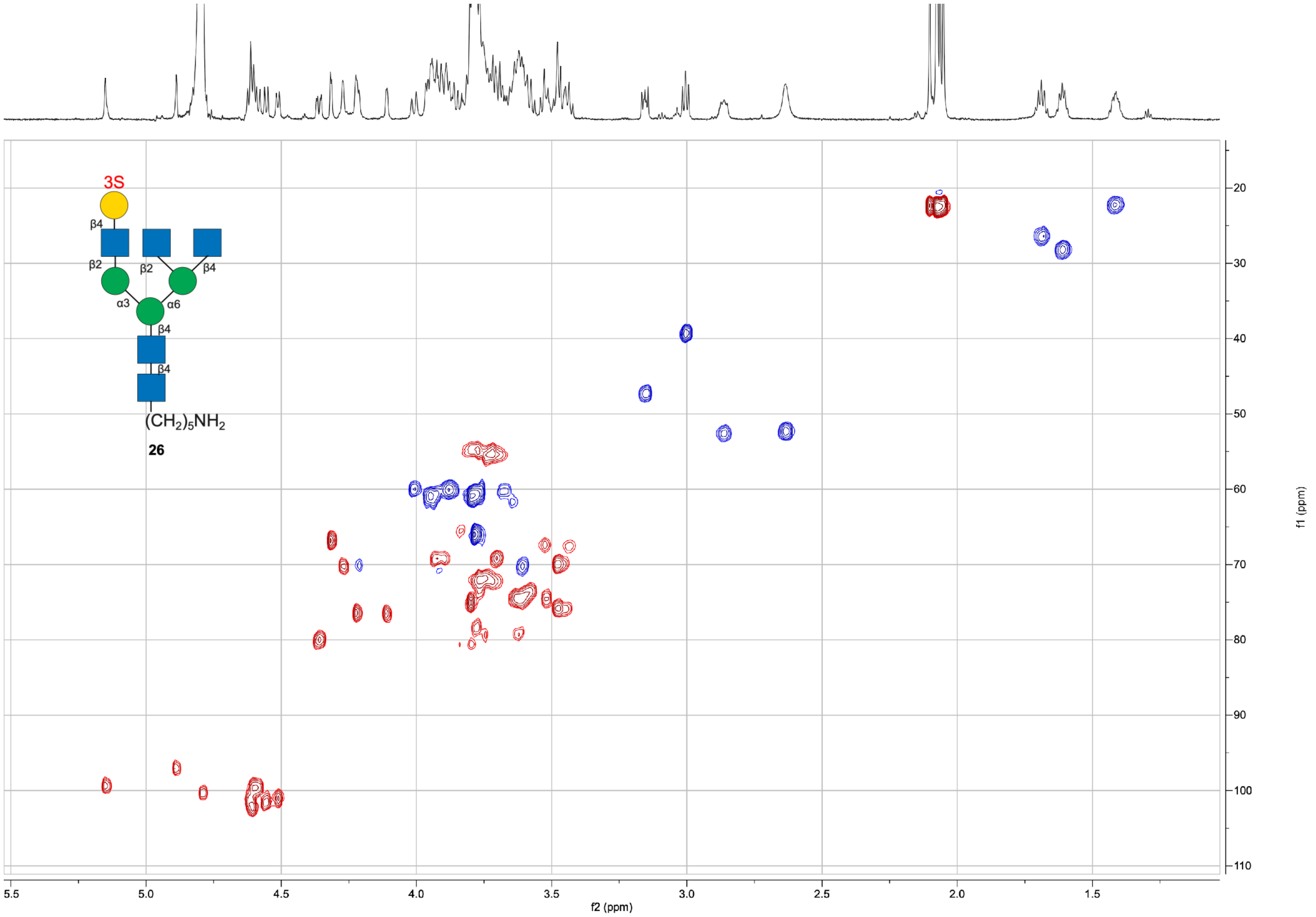

27 *^{1}H NMR spectrum*

700 MHz in D_2O

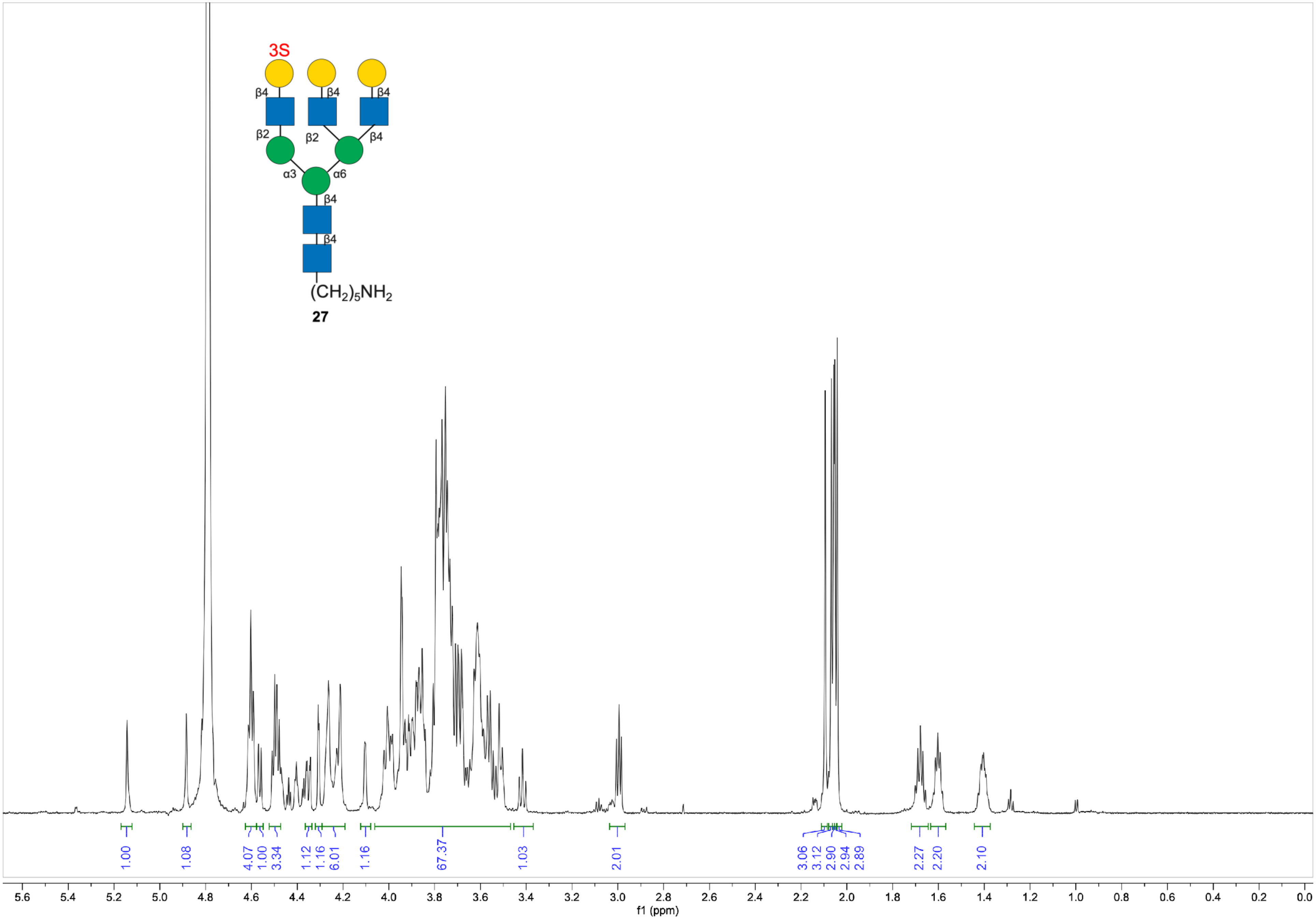

27 *HSQC spectrum*

700 MHz for ^{1}H in D_2O, Pulse Sequence: hsqcedetgpsisp2.3, NS 16, NUS 25%, AV700, probe CPTCI

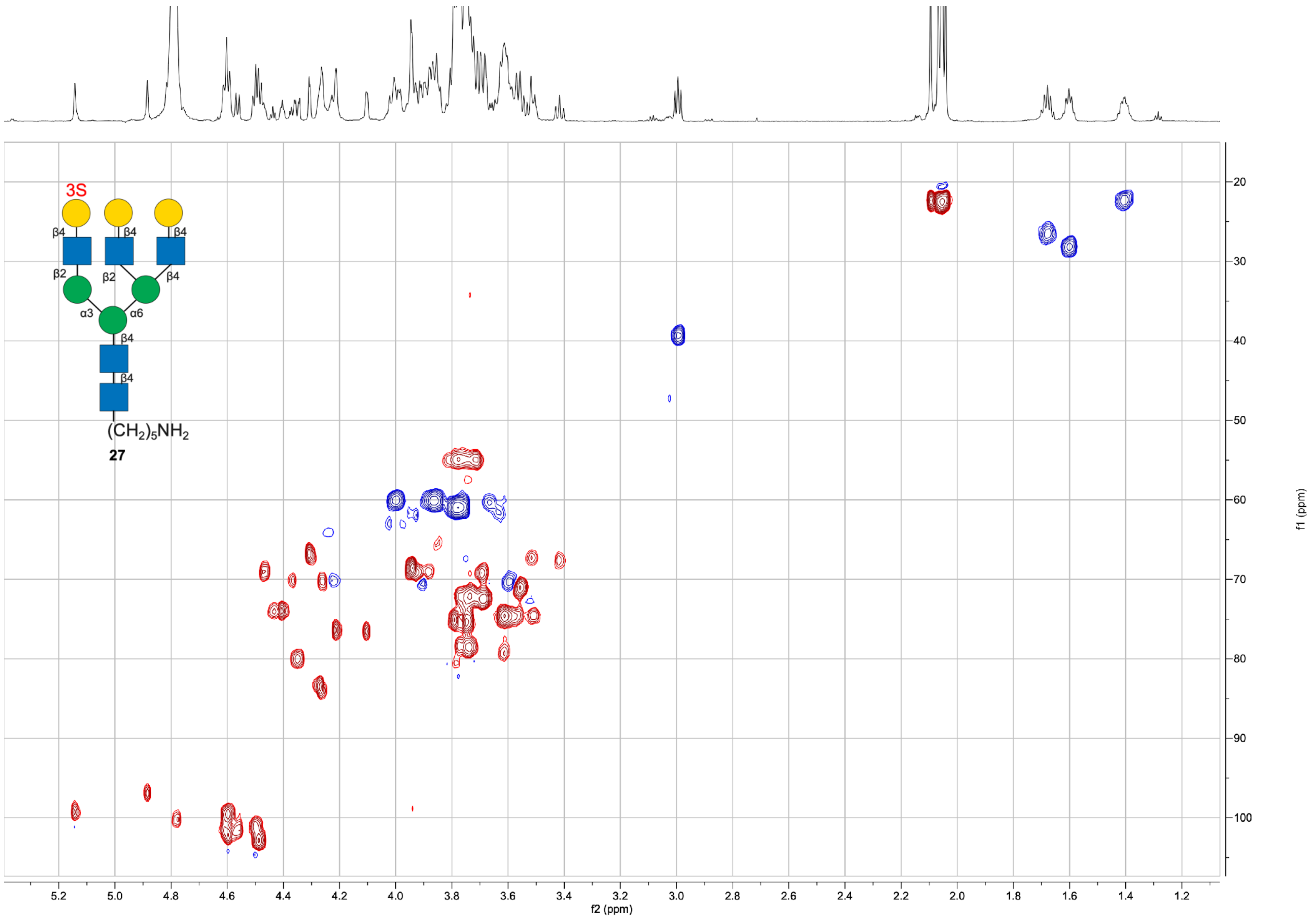

29 *^{1}H NMR spectrum*

600 MHz in D_2O

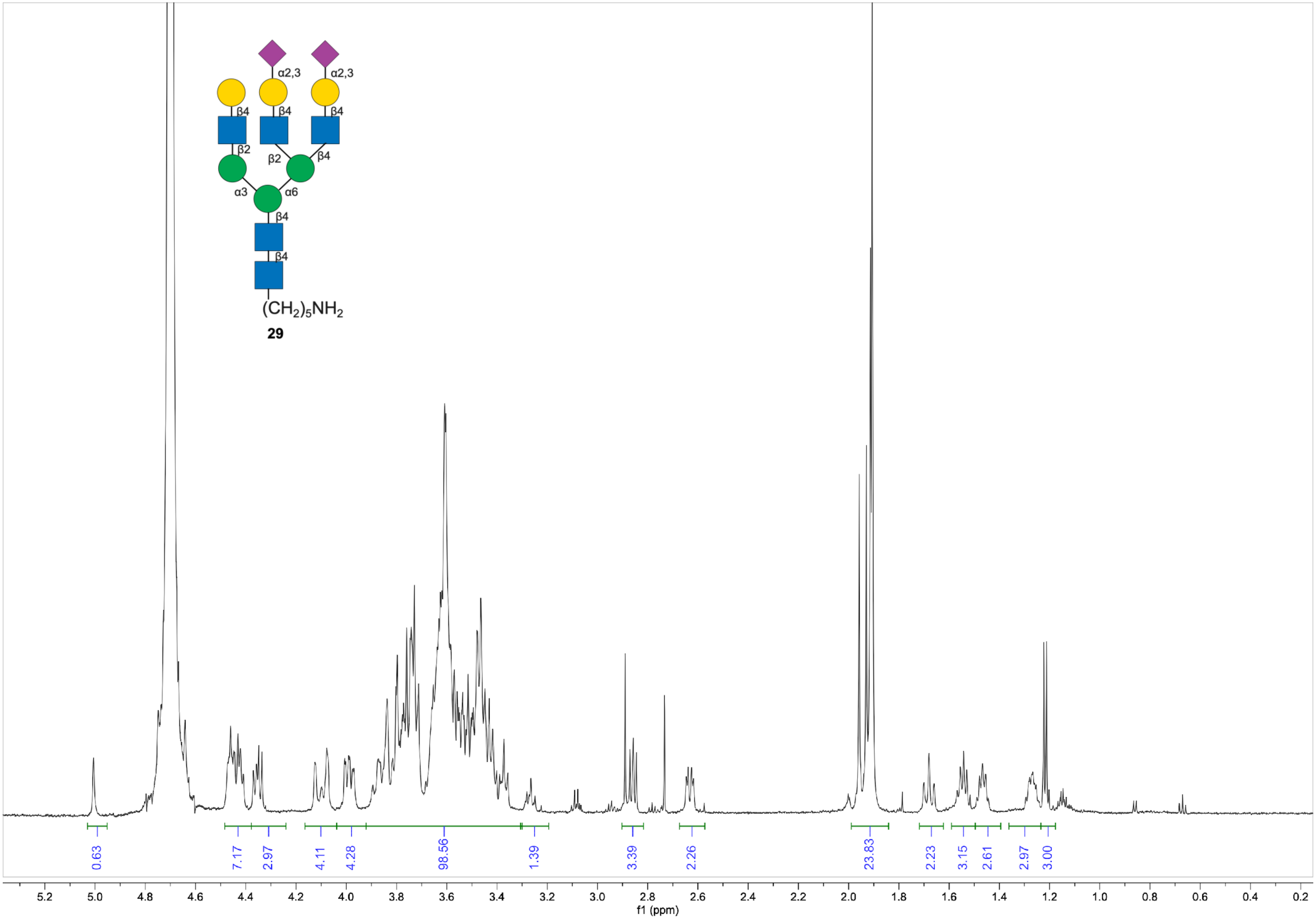

29 *HSQC spectrum*

600 MHz for ^1H in D_2O, Pulse Sequence: hsqcedetgpsisp2.3, NS 32, NUS 25%, AV 600, probe QCI

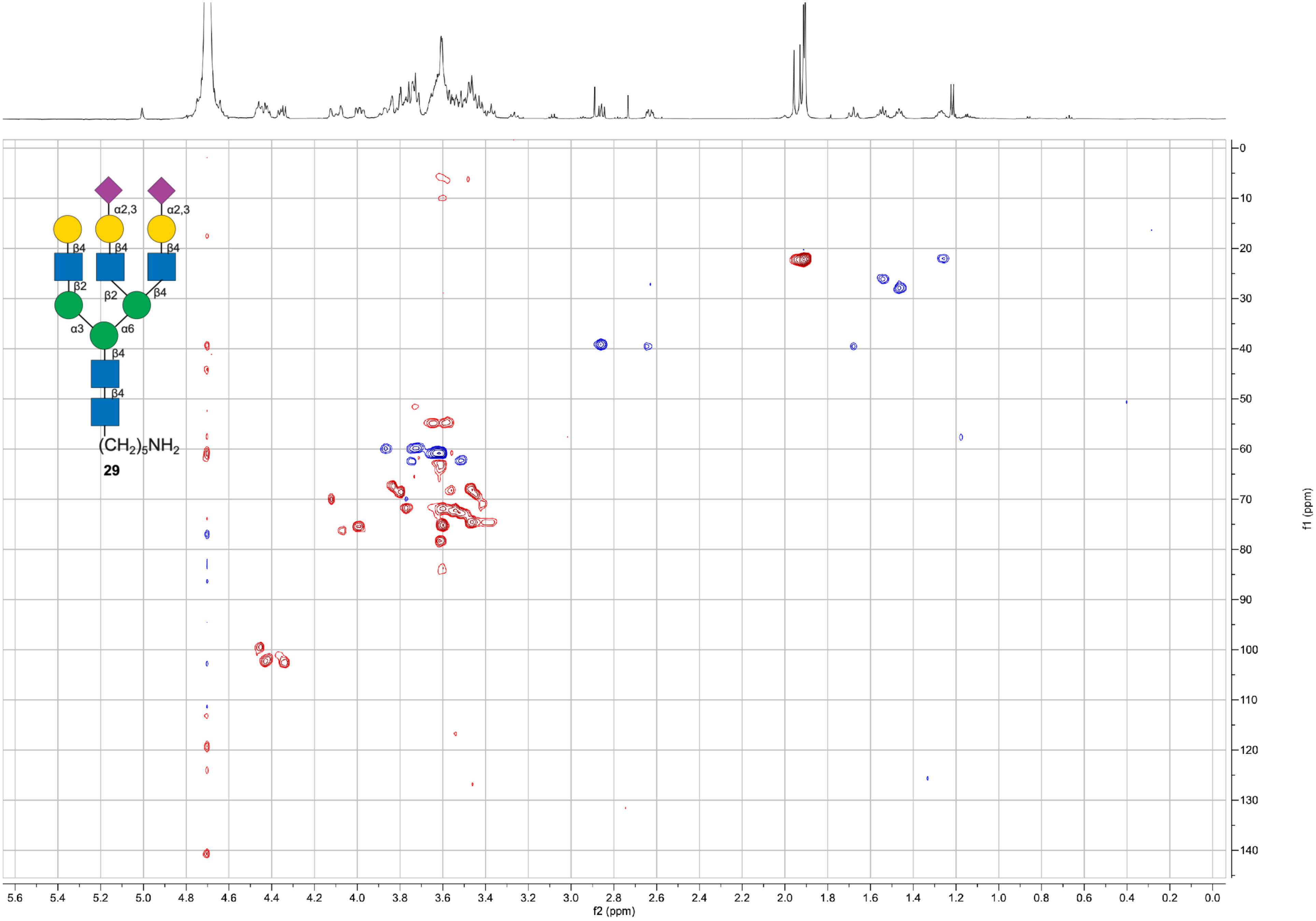

34 *^{1}H NMR spectrum*

600 MHz in D_2O

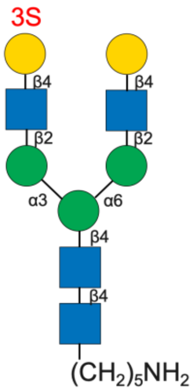

34

34 HSQC spectrum

600 MHz for ^{1}H in D_2O, Pulse Sequence: hsqcedetgpsisp2.3, NS 4, NUS 25%, AV 600, probe DCH

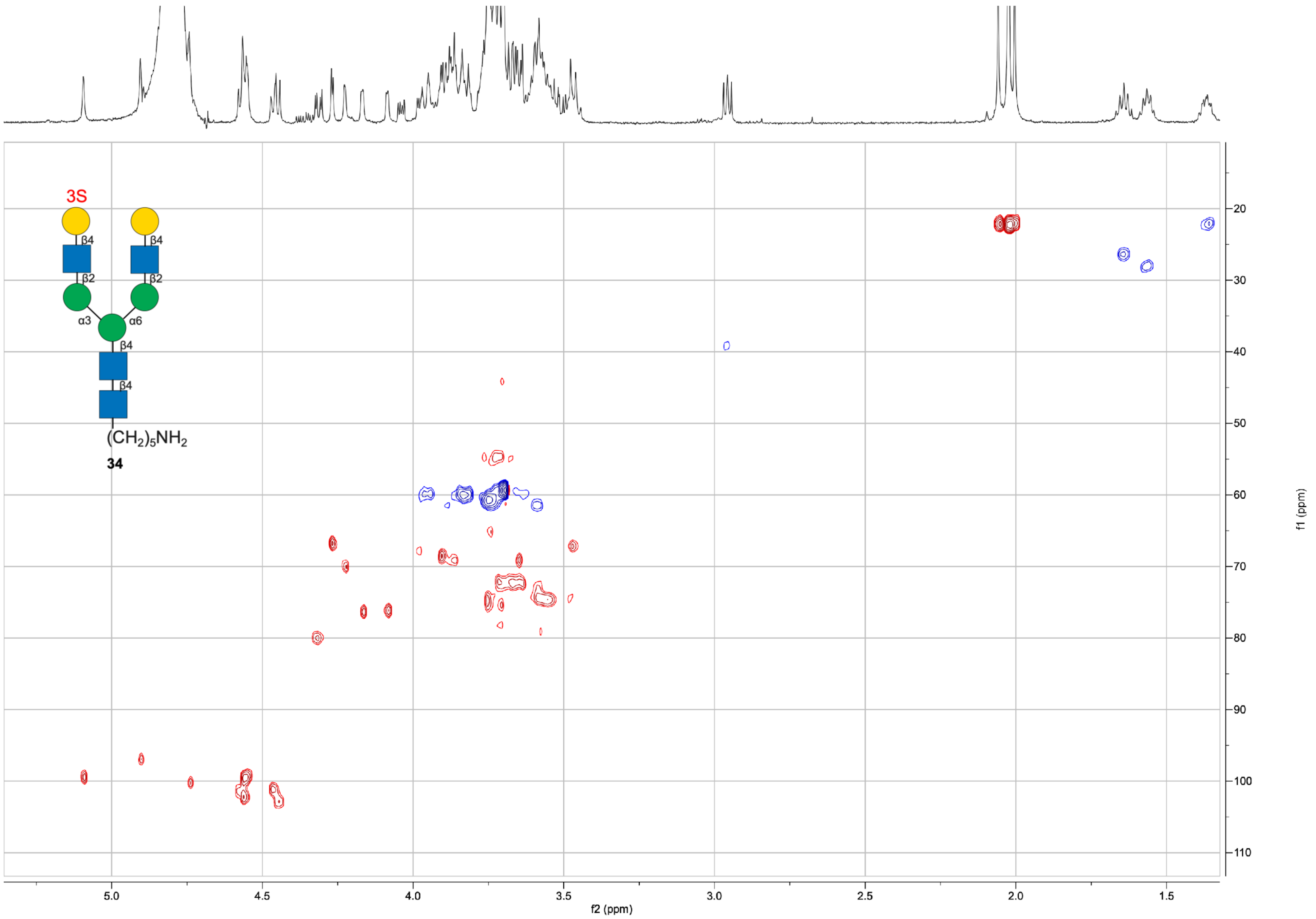

35 ^{1}H NMR spectrum

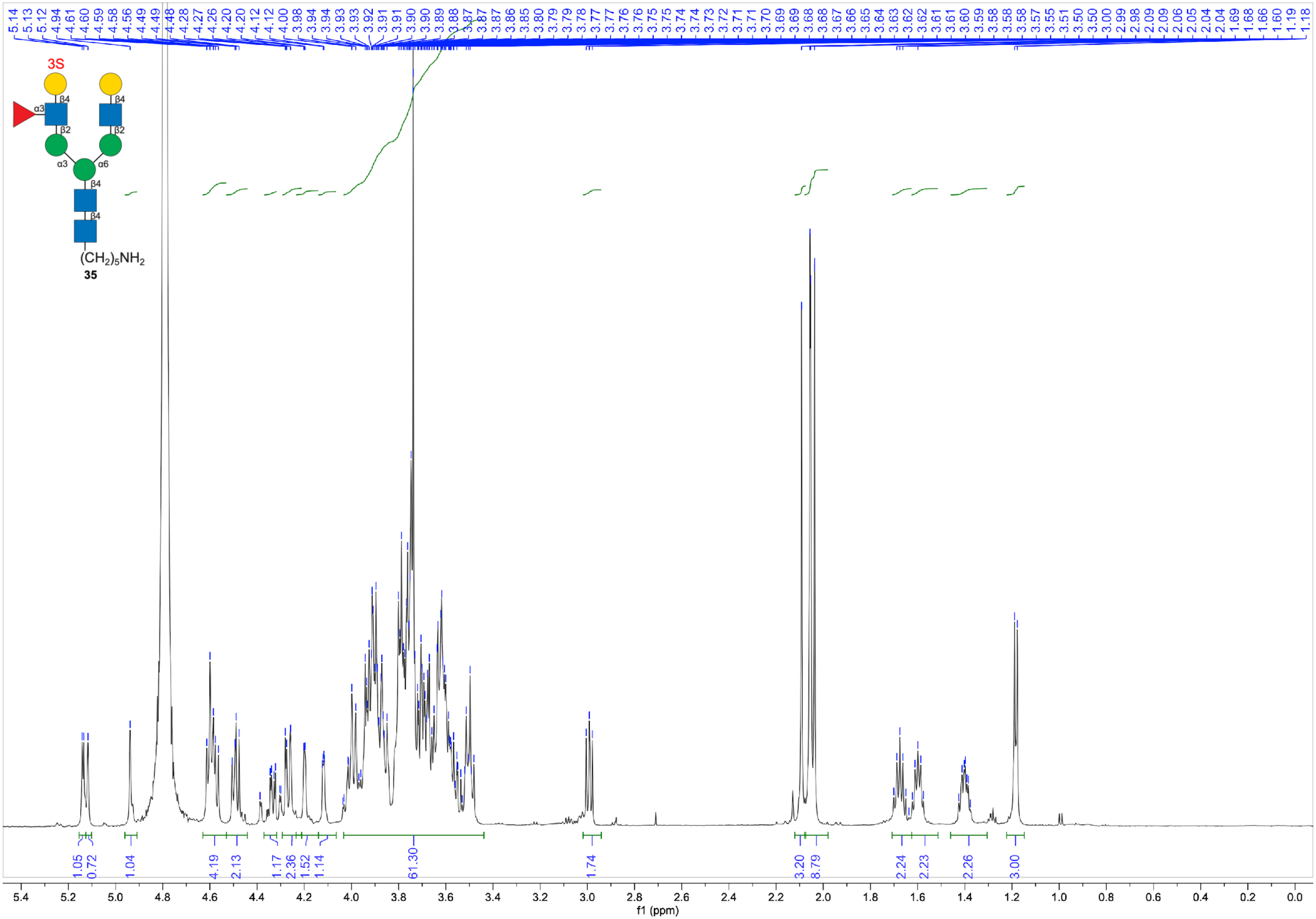

__35__ DEPTQ135 ^{13}C NMR spectrum

151 MHz in D_2O, Pulse Sequence: deptqgpsp.2, NS 1520, probe DCH

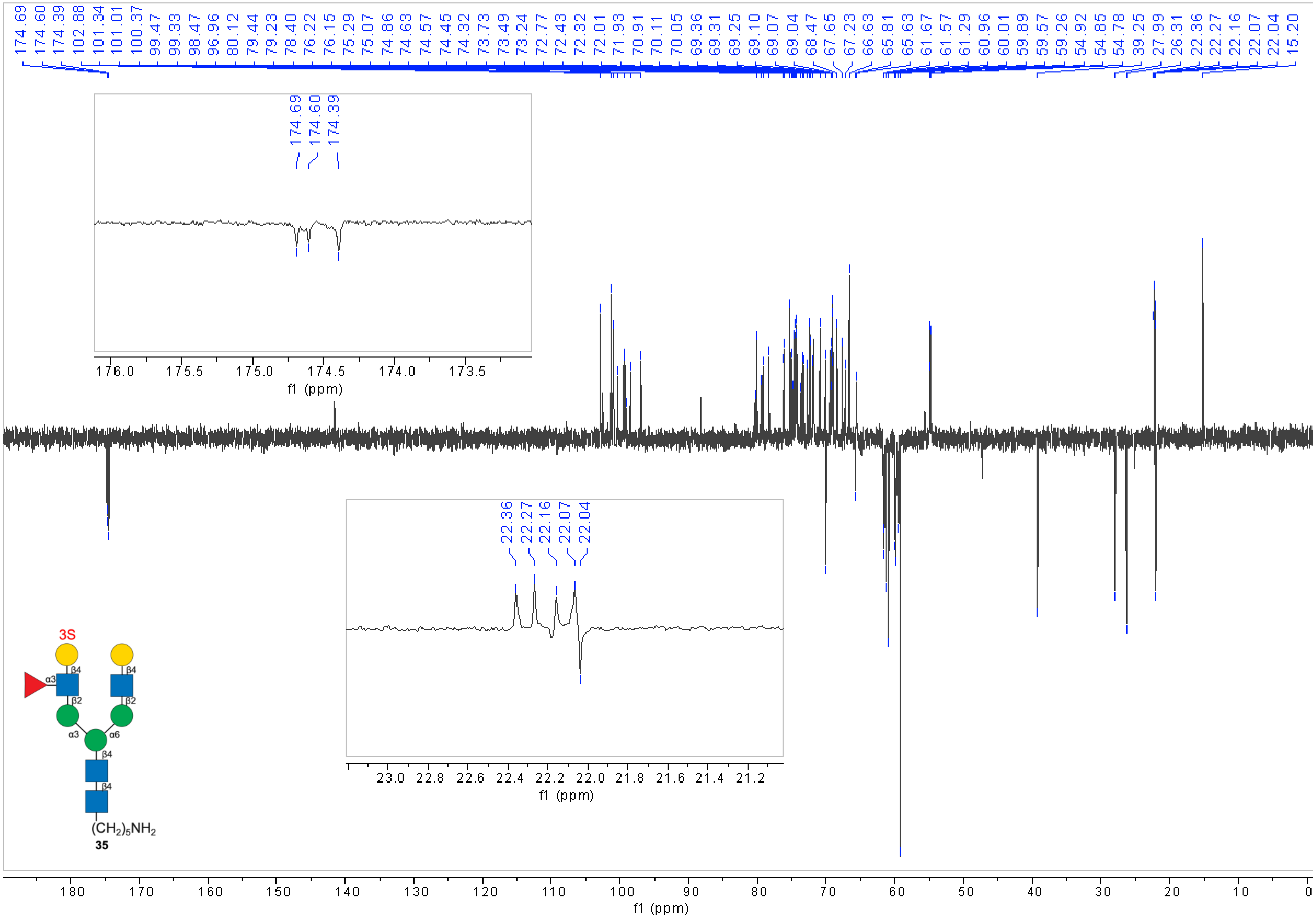

***35** TOCSY spectrum*

600 MHz for ^{1}H in D_2O, Pulse Sequence: mlevetgp, NS 16, AV 600, probe QCI

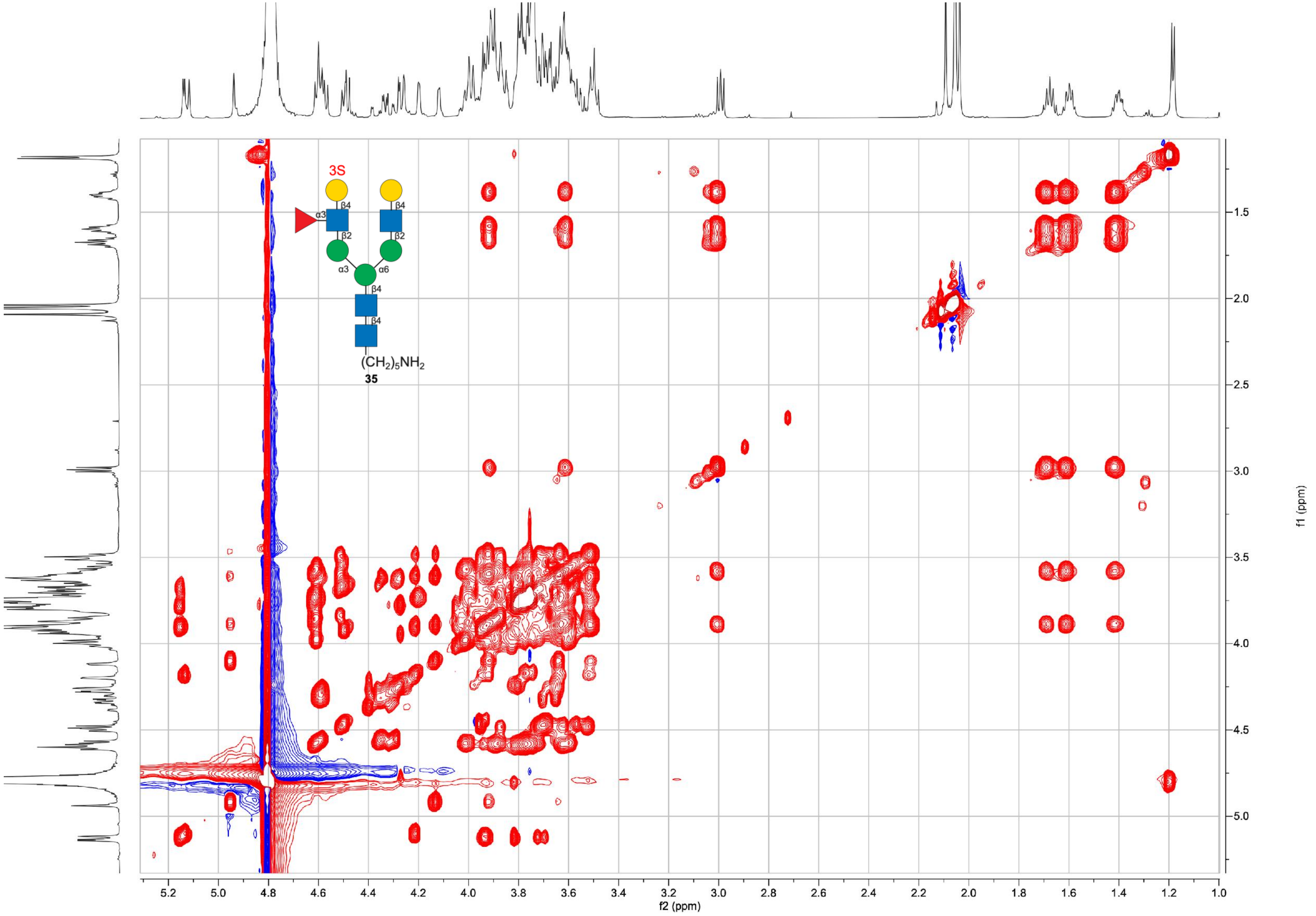

***35** COSY spectrum*

600 MHz for ^{1}H in D_2O, Pulse Sequence: noah4_BSCN, NS 16, AV 600, probe QCI

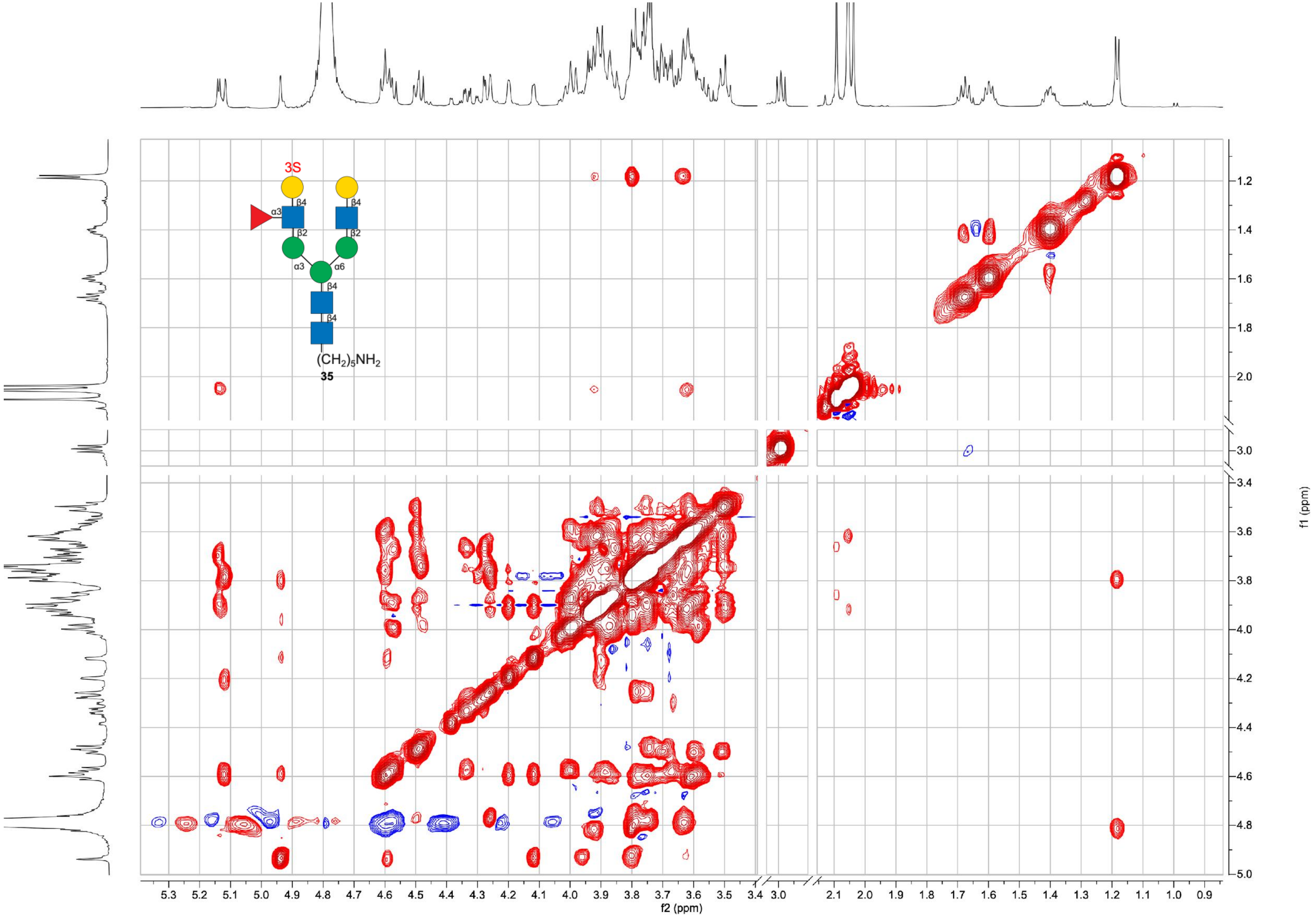

***35** HSQC spectrum*

600 MHz for ^{1}H in D_2O, Pulse Sequence: noah_4BSCN, NS 16, AV 600, probe QCI

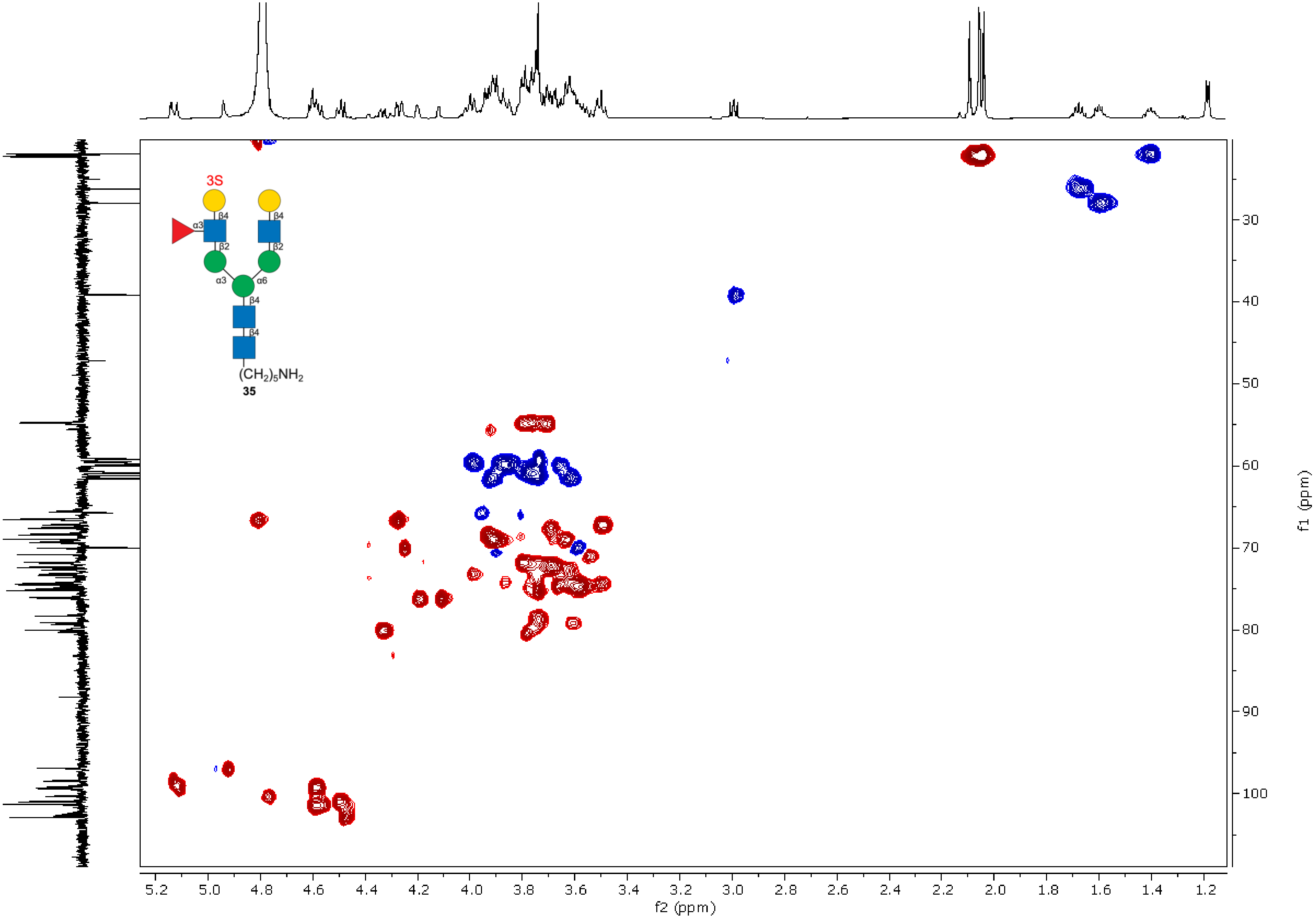

35 HMBC spectrum

600 MHz for ^{1}H in D_2O, Pulse Sequence: clhmbcetgpl3nd, NS 56, AV600, Probe DCH

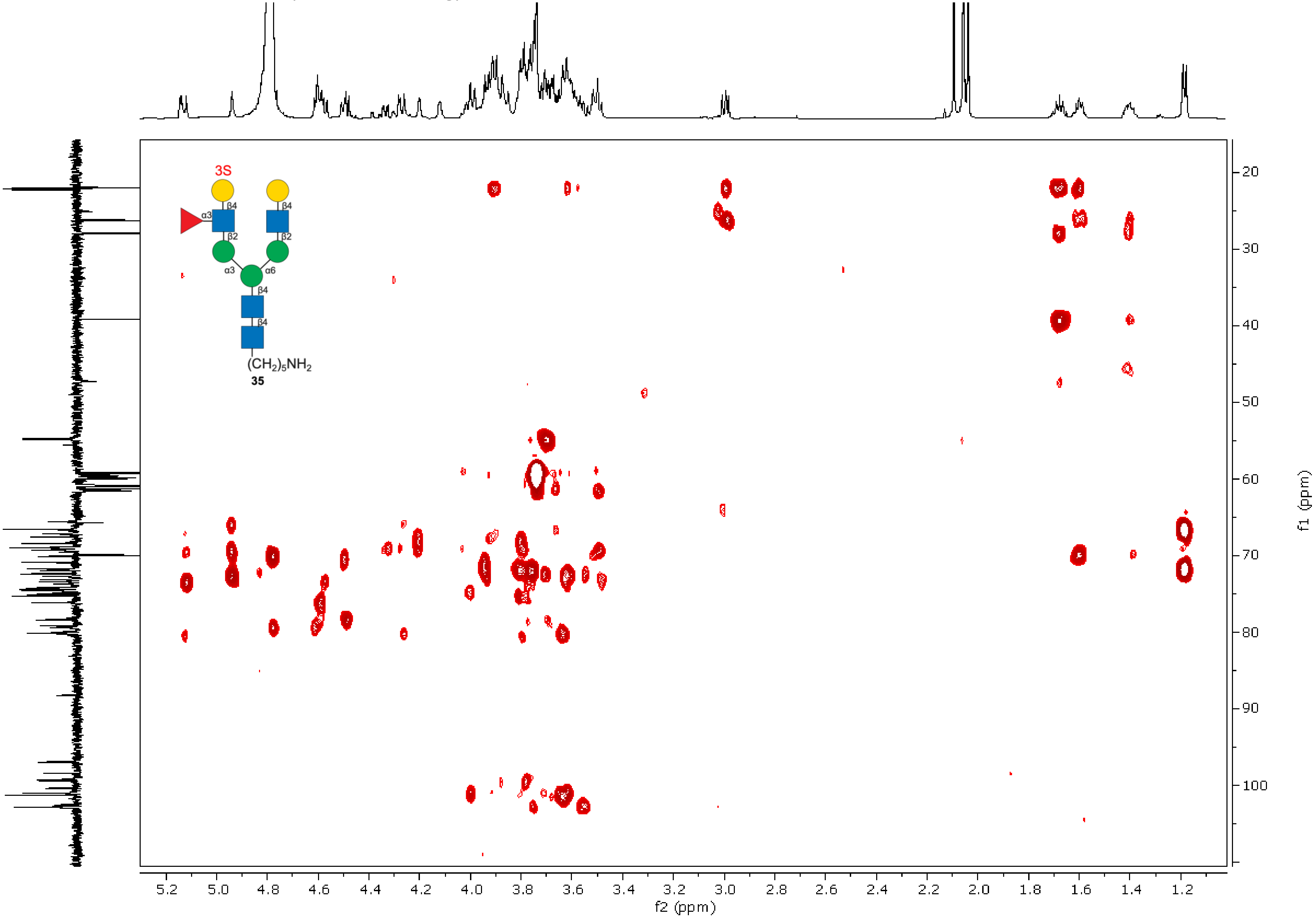

35 H2BC spectrum

600 MHz for ^{1}H in D_2O, Pulse Sequence: h2bcetgpl3, NS 56, AV600, Probe QCI

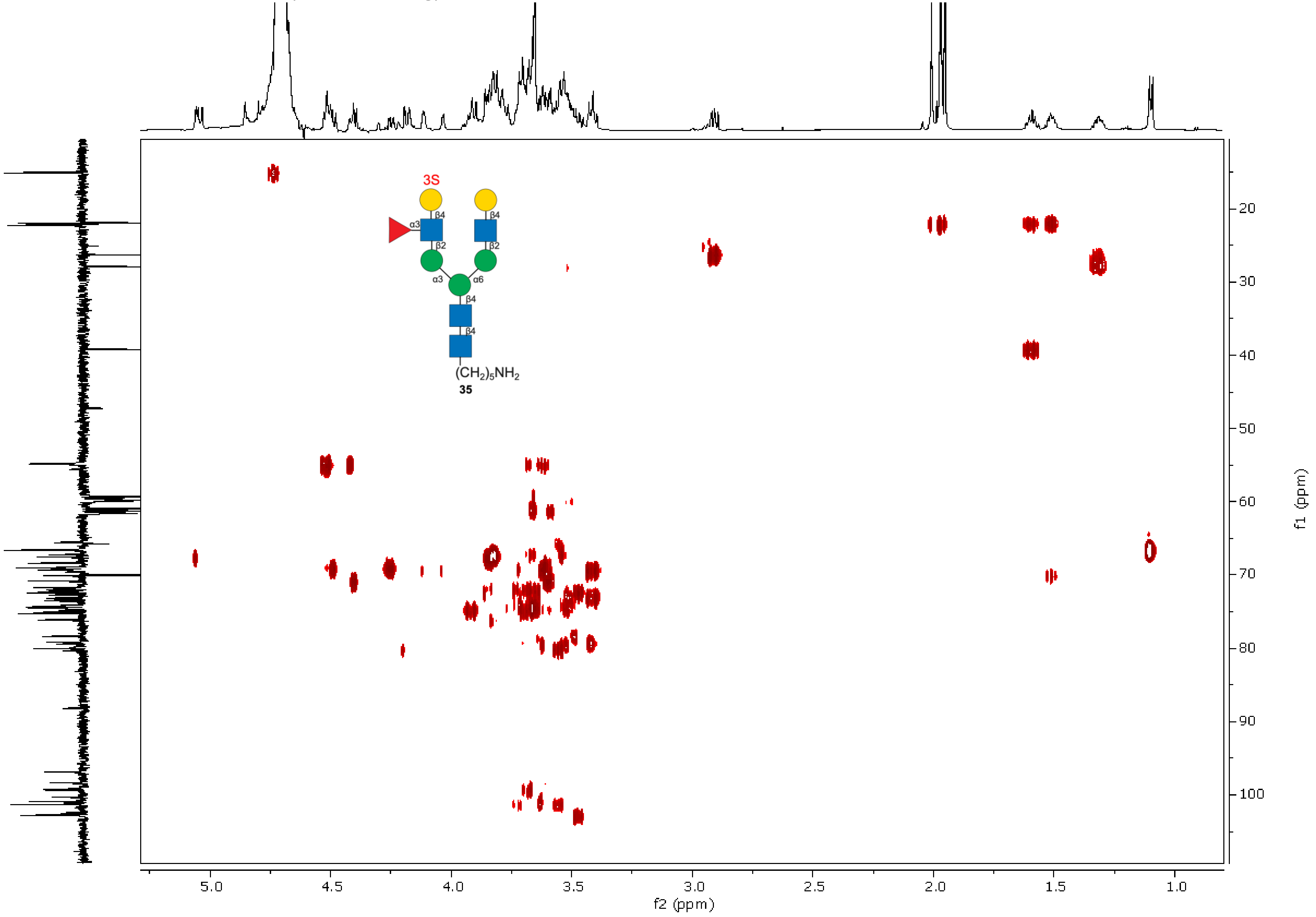

36 *^{1}H NMR spectrum*

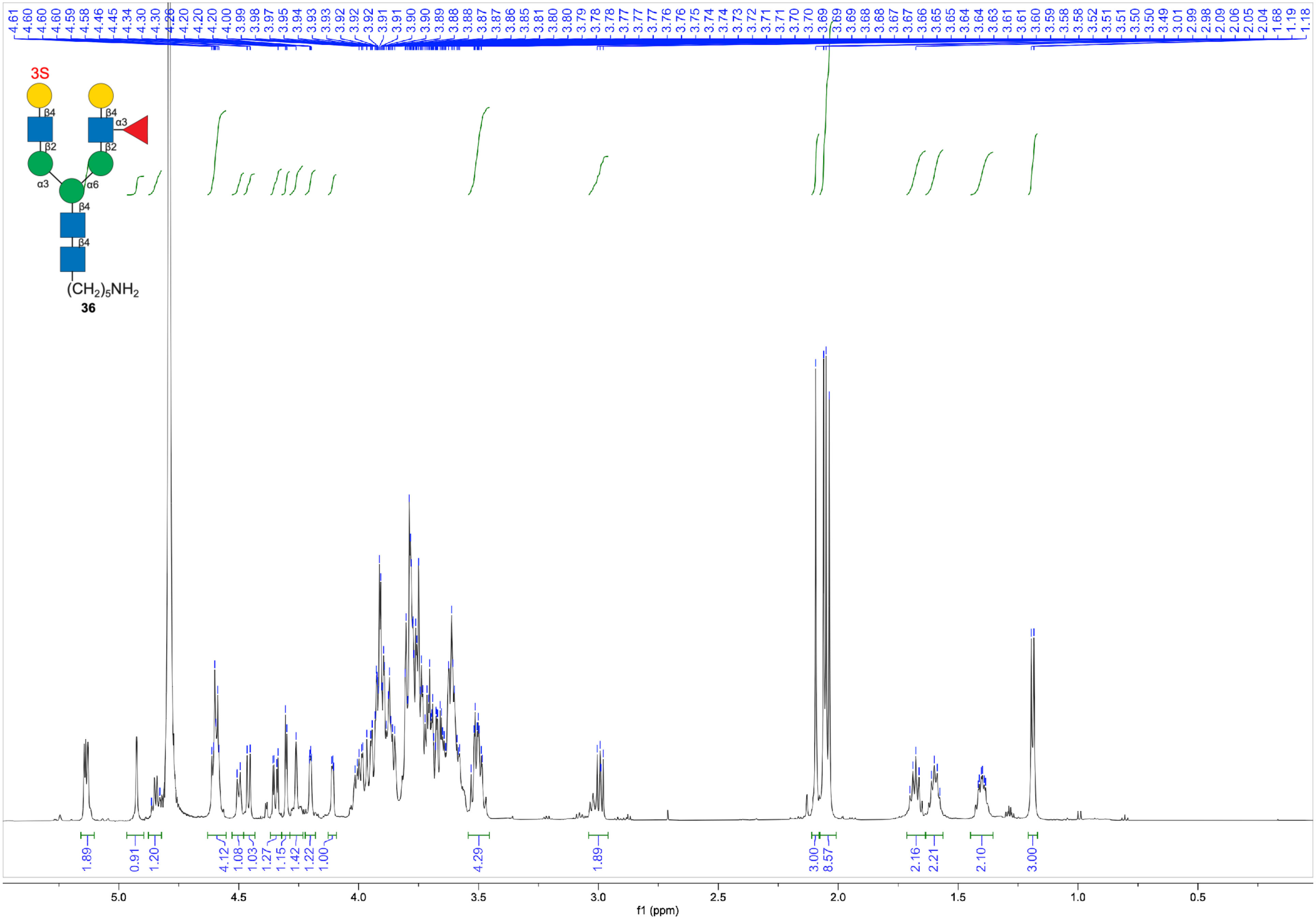

36 DEPTQ135 ^{13}C NMR spectrum

151 MHz in D_2O, Pulse Sequence: deptqgpsp.2, NS 1024, AV 600, probe DCH

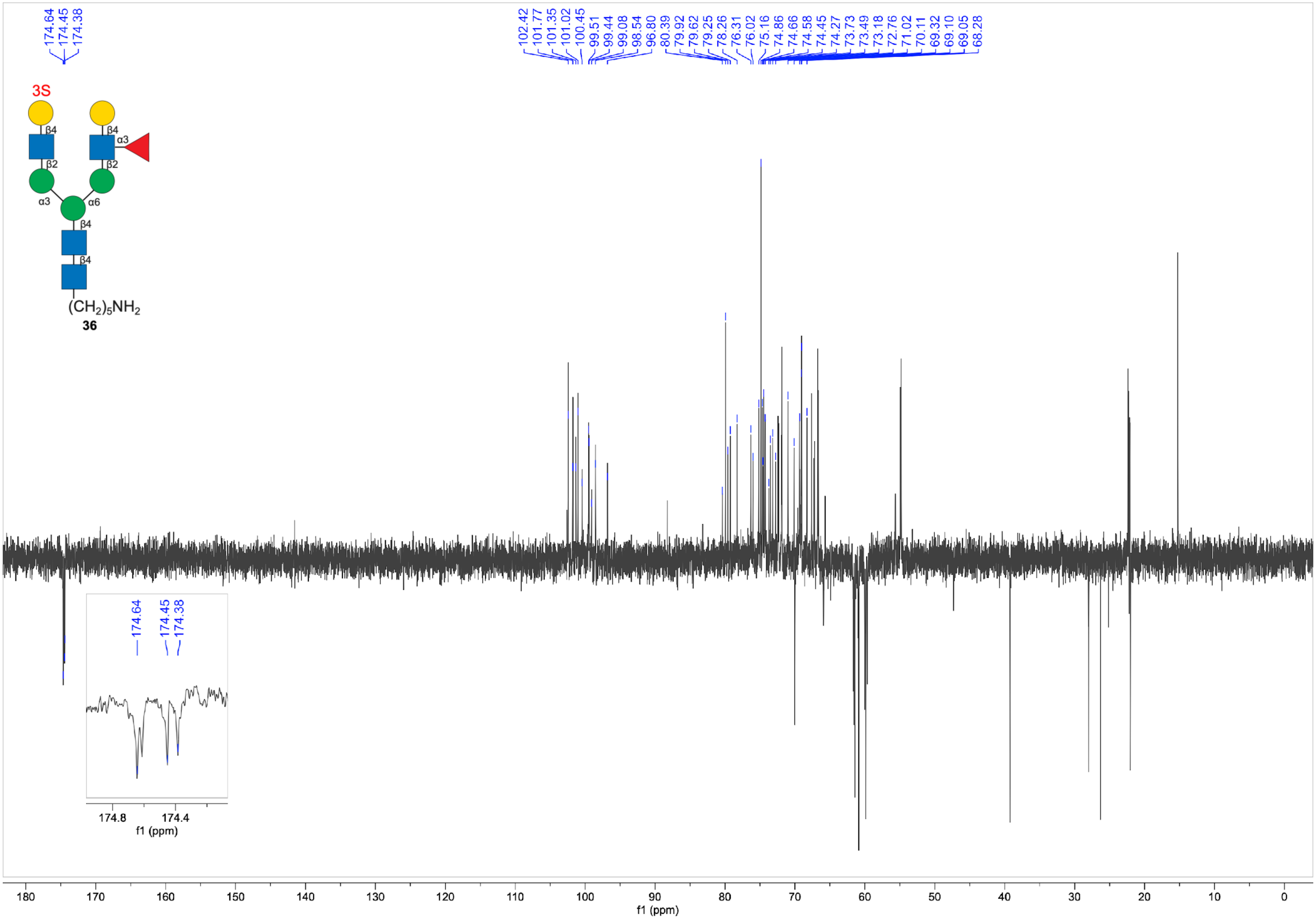

__36__ HSQC spectrum

600 MHz for ^{1}H in D_2O, Pulse Sequence: hsqcedetgpsisp2.3, NS 4, NUS 25%, AV 600, probe DCH

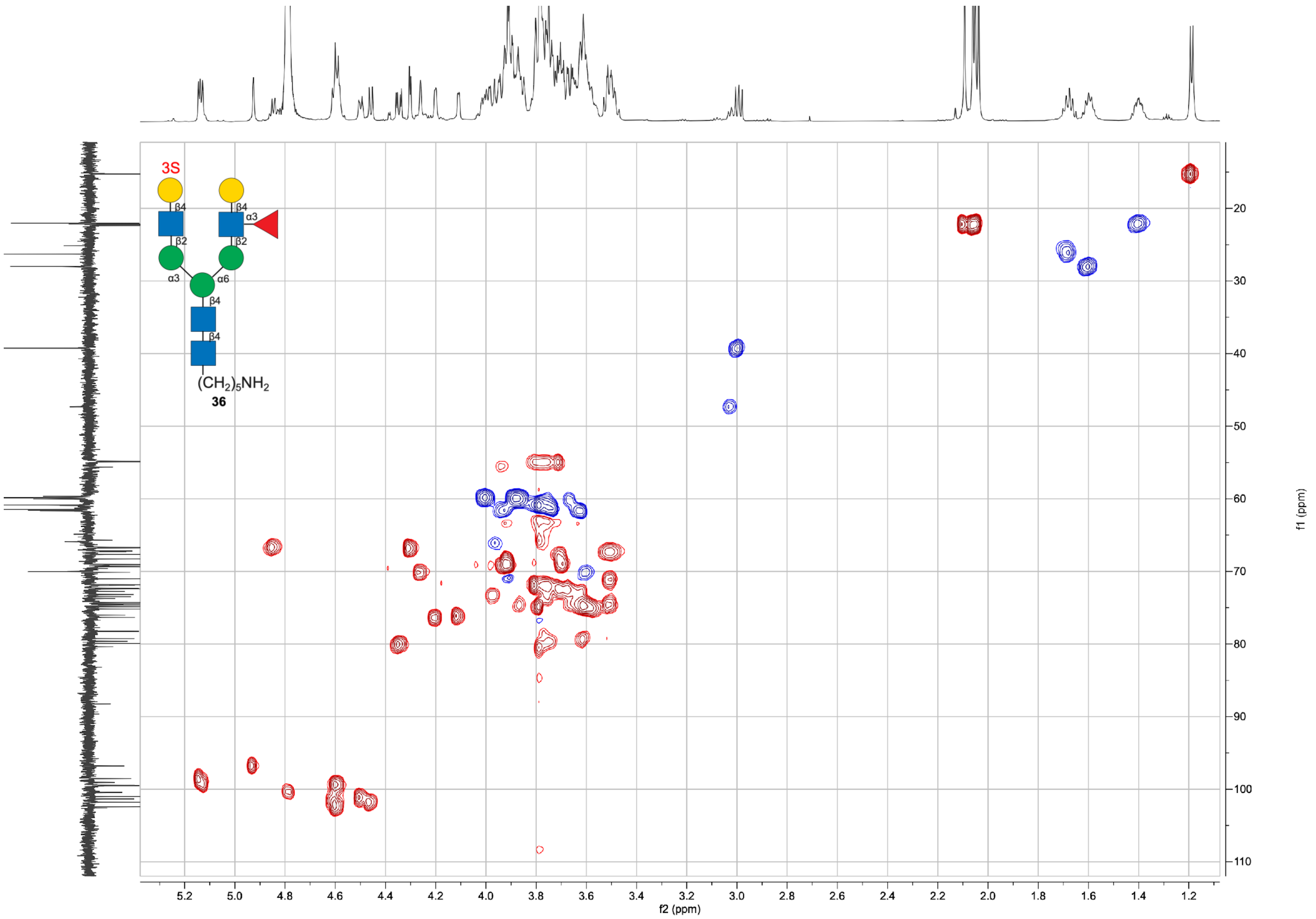

36 *HSQC spectrum with splitting via F2 phase*

600 MHz for ^{1}H in D_2O, Pulse Sequence: hsqcedetgpsisp.2, NS 16, NUS 25%, AV 600, probe DCH

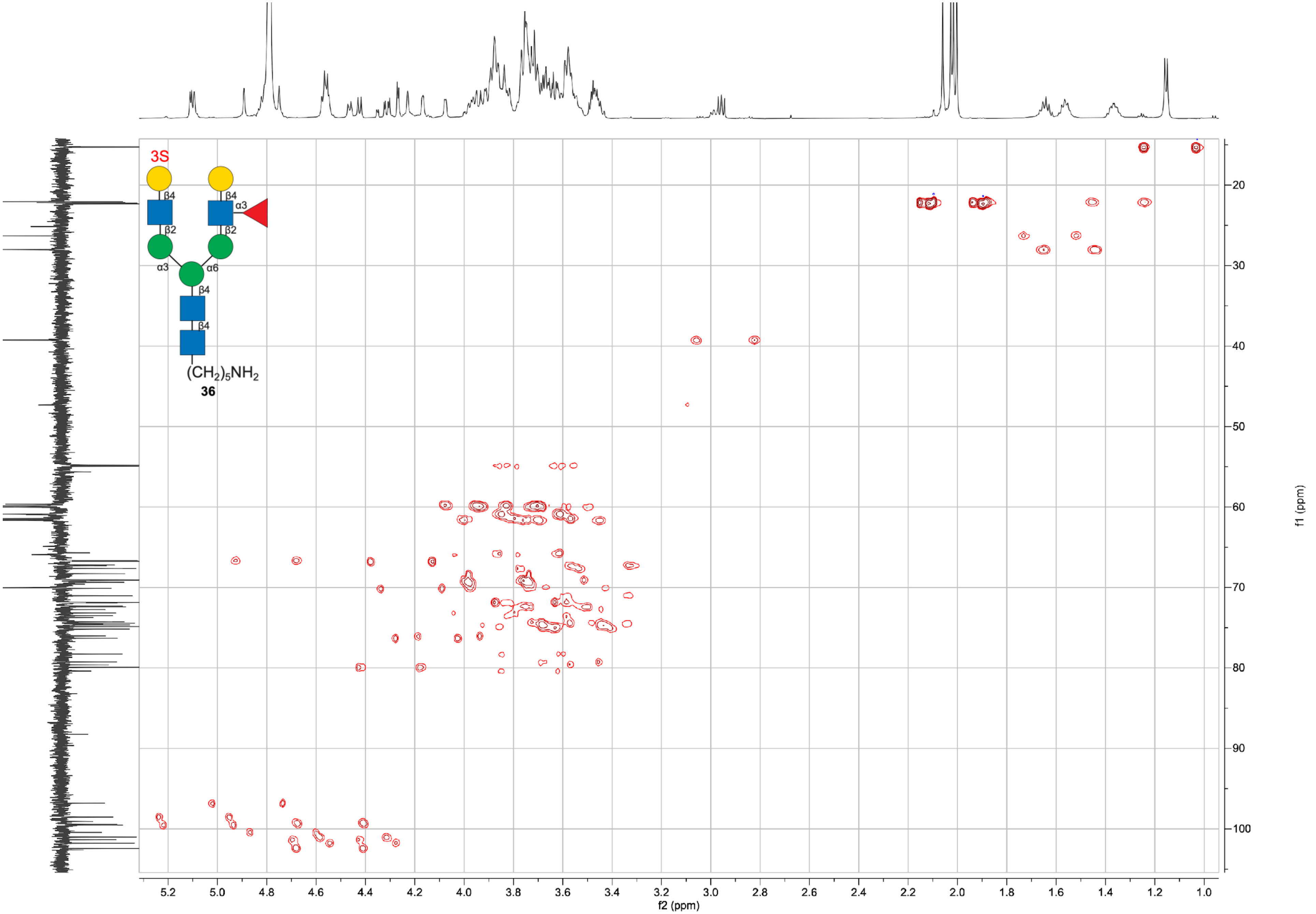

__36__ HMBC spectrum

600 MHz for ^{1}H in D_2O, Pulse Sequence: hmbcetgpl3nd, NS 16, NUS 50%, AV 600, probe DCH

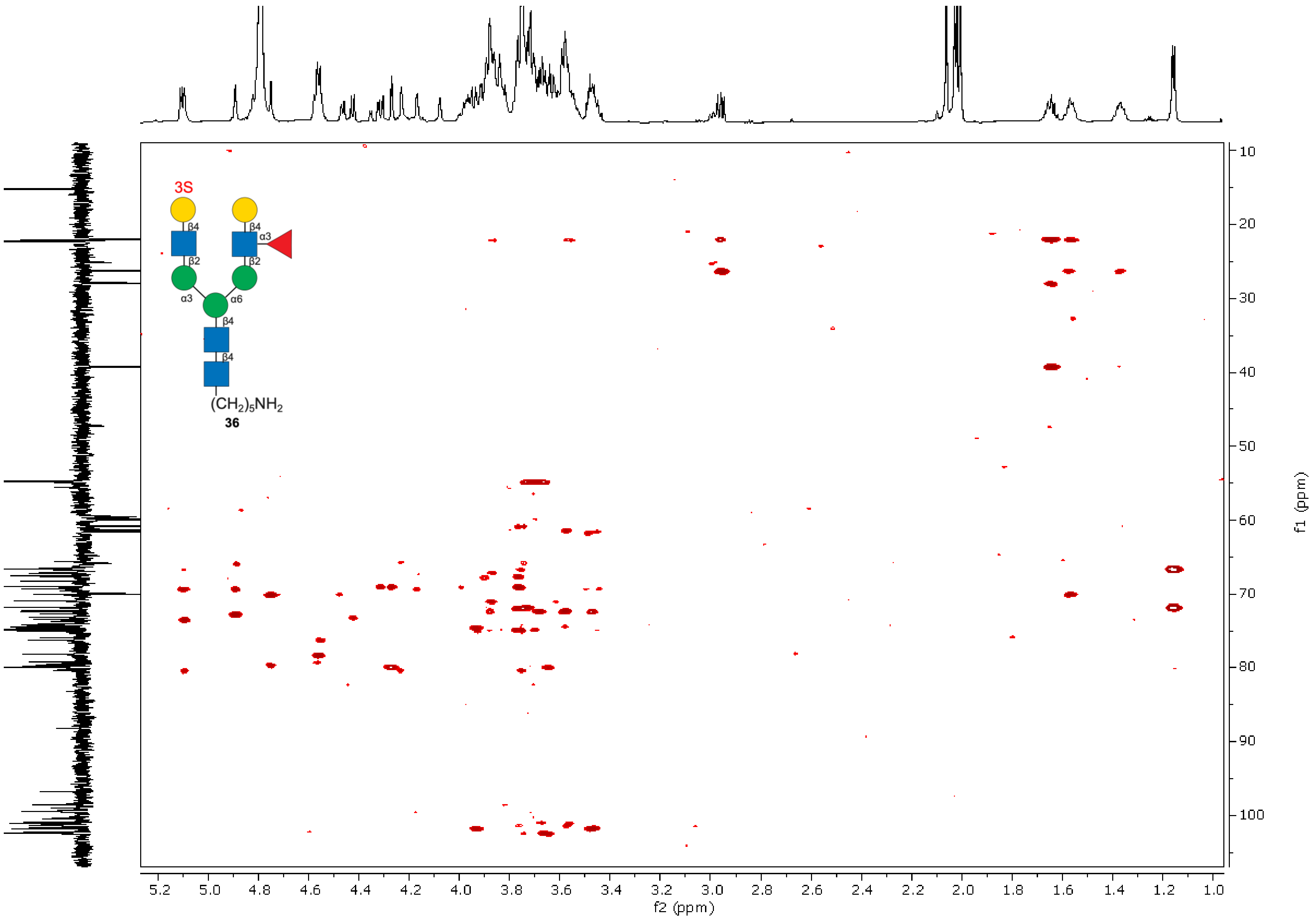

36 TOCSY spectrum

600 MHz for ^{1}H in D_2O, Pulse Sequence: mlevetgp, NS 16, AV 600, probe DCH

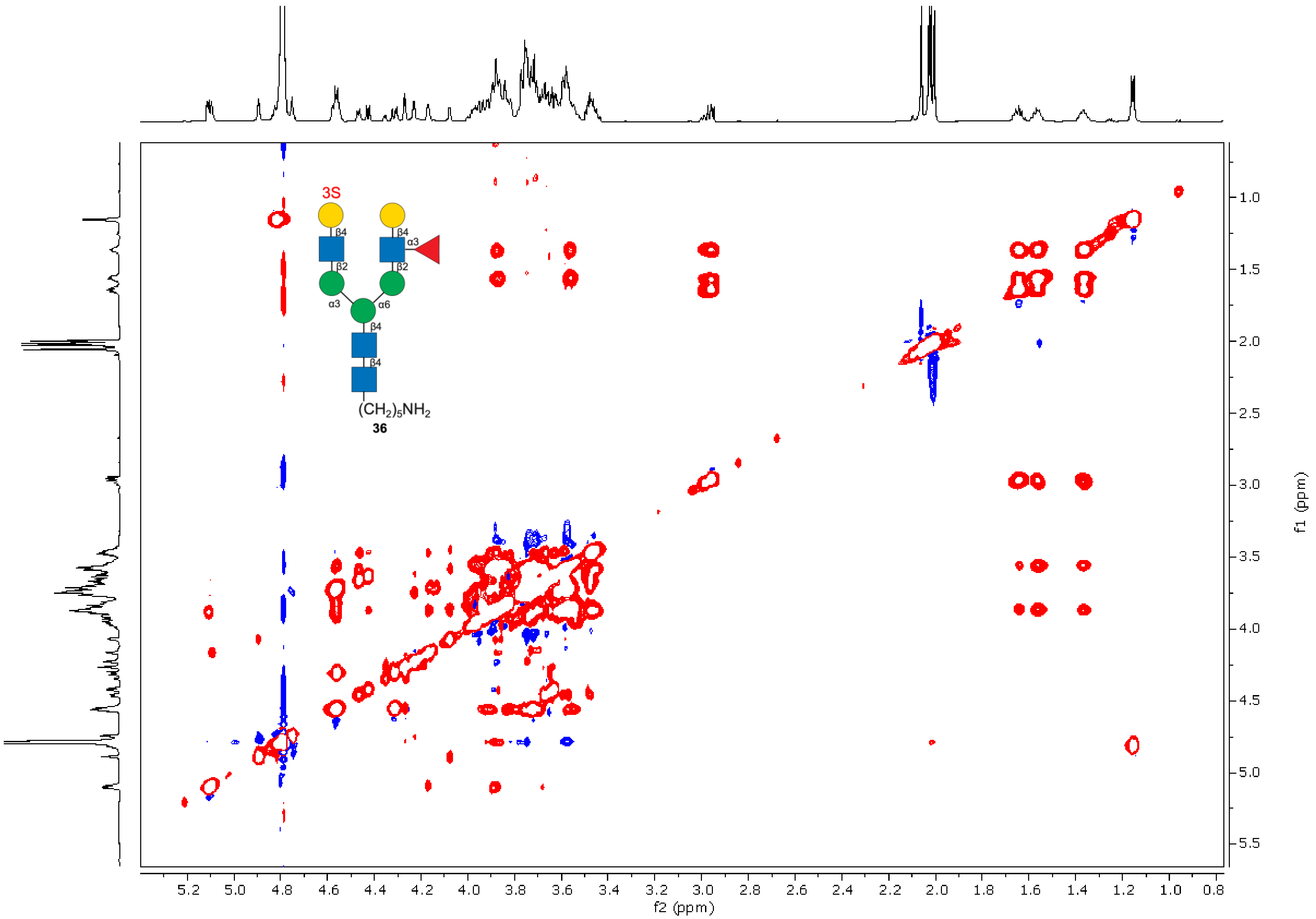

***36** H2BC spectrum*

600 MHz for ^{1}H in D_2O, Pulse Sequence: h2bcetgpl3, NS 128, AV 600, probe QCI

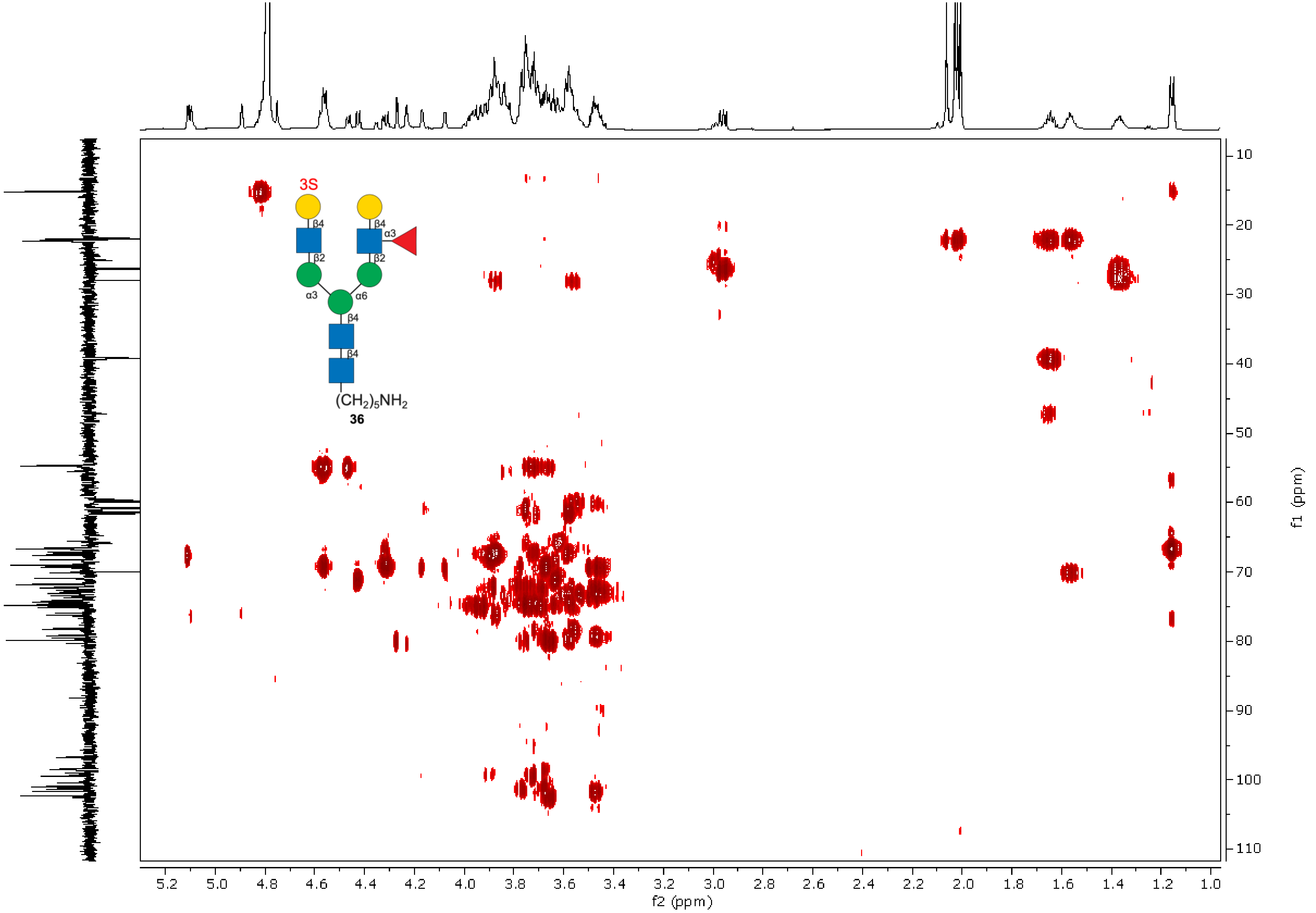

Supplement: Supplementary file 1 [file ja6c06320_si_001.pdf]
